# Supplementary material for: Comparative genomic analysis of SET domain family reveals the origin, expansion, and putative function of the arthropod-specific SmydA genes as histone modifiers in insects
Source: Gigascience. 2017 Apr 22;6(6):1–16. doi: 10.1093/gigascience/gix031 (PMC5459927; doi:10.1093/gigascience/gix031)
Supplement: GIGA-D-16-00127_Original_Submission.pdf [file gix031_GIGA-D-16-00127_Original_Submission.pdf]

Manuscript
Click here to download Manuscript insectsSetDomain_16102701_giga.doc
Click here to view linked References

Comparative genomic analysis of *SET*-domain family reveals the origin, expansion, and putative function of the arthropod-specific *SmydA* genes as histone modifier in insects

Feng Jiang[1, *], Qing Liu[1, 2, *], Yanli Wang[2, 3], Jie Zhang[1], Huimin Wang[1], Tianqi Song[3], Meiling Yang[2], Xianhui Wang[2, #], Le Kang[1, 2, #]

[1] Beijing Institutes of Life Science, Chinese Academy of Sciences, Beijing, China

[2] State Key Laboratory of Integrated Management of Pest Insects and Rodents, Institute of Zoology, Chinese Academy of Sciences, Beijing, China

[3] Institute of Applied Biology, Shanxi University, Taiyuan, Shanxi, China

*These authors contributed equally to this study.

Corresponding authors:

Le Kang, Ph.D. and Professor

Institute of Zoology, Chinese Academy of Sciences

Beijing 100101, China

Tel: 86-10-6480-7219

Fax: 86-10-6480-7099

E-mail: lkang@ioz.ac.cn

OR

Xianhui, Wang Ph.D. and Professor

Institute of Zoology, Chinese Academy of Sciences

Beijing 100101, China

Tel: 86-10-64807220

Fax: 86-10-6480-7099

E-mail: wangxh@ioz.ac.cn

Evolution of *SET* Genes in Insects

Abstract

The *SET* domain is an evolutionarily conserved motif present in histone lysine methyltransferases, which are important in the regulation of chromatin and gene expression in animals. In this study, we searched for *SET* domain-containing genes (*SET* genes) in all of the 147 arthropod genomes sequenced so far to understand the evolutionary history by which *SET* domain have evolved in insects. Phylogenetic and ancestral state reconstruction analysis revealed a arthropod-specific *SET* gene family, named *SmydA*, which is ancestral to arthropod animals and specifically diversified during insect evolution. Considering that pseudogenization is the most probable fate of the new emerging gene copies, we provided experimental and evolutionary evidence to demonstrate their essential functions. Fluorescence *in situ* hybridization analysis and *in vitro* methyltransferase activity assays showed that the *SmydA-2* gene was transcriptionally active and retained the original histone methylation activity. Expression knockdown by RNA interference significantly increased mortality, implying that the *SmydA* genes may be essential for insect survival. We further showed predominantly strong purifying selection on the *SmydA* gene family and a potential association between the regulation of gene expression and insect phenotypic plasticity by transcriptome analysis. Overall, these data suggest that the *SmydA* gene family retains essential functions that may possibly define novel regulatory pathways in insects. This work provides insights into the roles of lineage-specific domain duplication in insect evolution.

Key words: insects, domain, gene duplication, histone modification.

Background

Protein domains are functional and structural units that are evolutionary well conserved across species [1]. Specific protein domains are often linked to discrete biological function; therefore, the frequent duplication, gain, and loss of protein domains play substantial roles in functional novelty [2]. Domain duplication can be achieved via frequent domain-containing gene family expansion. Thus, the member number of a gene family that contains domains can be expanded, representing a common method by which divergence to domain sequences can lead to the evolutionary novelty of domain-containing genes [3]. In taxonomically related species, the expansion of conserved gene families through gene duplication is widespread in metazoan genomes [4]. Gene duplication may increase species fitness by subfunctionalization or neofunctionalization [5, 6]. Subfunctionalization results in the symmetric division of the functional capability of the original gene among the duplicated genes [7]. Neofunctionalization allows the original copy to maintain its function and permits the new copy to diverge under relaxed selective constraints or positive selection for a novel function. Rapid domain diversification followed by gene duplications in particular lineages is important for the adaptation of lineage-specific ecological specializations [8].

Histones are highly alkaline proteins in cell nuclei that package and order the nuclear DNA into nucleosomes, which are the main components of chromatin. Histone modifications are a major epigenetic regulatory mechanism for phenotypic plasticity in insects. Inhibition of histone deacetylation affects developmental plasticity both in ants (*Camponotus floridanus*) and honeybees (*Apis mellifera*) [9, 10]. Genome-wide profiling of histone modifications revealed an important role of histone

H3 lysine 27 acetylation in the caste differentiation of ants [11]. Methylations of histone H3 lysine 27 and histone H3 lysine 36 are more abundant in queen ovaries than in larvae, implying that histone methylation plays a specific role in honey bees [12]. In recent years an increasing number of publications have established histone lysine methylation as a central epigenetic modification in regulation of chromatin and transcription. The *SET* domain, which is observed in many histone lysine methyltransferases, is widely and probably universally distributed in metazoan species. This protein family typically comprises an approximately 130 amino acid-long *SET* domain, which was identified in the strongest PEV suppressor gene Su(var)3-9, in the Pc-G gene Enhancer of zeste [E(z)] and in the activating trx-G gene Trithorax of *Drosophila* [13]. The *SET* domain possesses a catalytic activity that transfers a methyl group to the amino group of lysine residues of nuclear histones from S-adenosyl-L-methionine. Based on their biochemical characteristics, *SET* domain is capable of catalyzing mono-, di- or tri-methylation of their lysine substrates. *SET* domain-dependent methylation has been identified in a wide range of lysine residues in different histones: K4 (K is the abbreviation for lysine), K9, K27, K36, and K79 in histone H3; K20 in histone H4; K59 in the globular domain of histone H4; and K26 in histone H1B [14]. Methylation of lysine residues in histone proteins is an important post-translational epigenetic event that regulates gene expression by serving as an epigenetic marker for the recruitment of complexes that participate in the organization of chromatin structure [15]. The importance of *SET*-domain containing genes is strongly supported by the involvement of this protein family in diverse biological mechanisms, such as transcriptional activation, transcriptional repression, enhancer function, mRNA splicing and DNA replication

Evolution of *SET* Genes in Insects

[16]. Therefore, expectedly, the regulation of various *SET*-domain containing genes are increasing correlated with diverse epigenetic phenomena which, for example, include epigenetic control in plants, centromeric gene silencing in yeasts, repeat-induced point mutations in fungi, DNA elimination in *Tetrahymena*, germline chromatin silencing in worms and heterochromatin formation in flies [17].

Insects constitute a remarkably diverse group of organisms that make up a vast majority of known species with their importance including biodiversity, agricultural, and human health concerns. The insect lineage comprises species that are both cosmopolitan distributed and geographically restricted, showing a broad range of adaptation diversity. The evolutionary history of gene families is not confounded by whole-genome duplication, and the major topology of insect species is well resolved [18]. Therefore, the insect lineage offers an excellent model to study domain/gene evolution in the context of gene family dynamics [19-23]. Insect *SET* domain-containing genes (*SET* genes) have been identified in a limited number of representative insect species without complicated analysis [24-26]. The *Smyd* subfamilies of *SET* genes have expanded in a few insects from Diptera and Hymenoptera, and several members of the *Smyd* subfamilies show significant changes in gene expression in response to phenotypic plasticity in ants [27, 28]. However, the evolutionary history of insect *SET* genes remains largely unknown because the *SET* genes from a broad range of insect species have not been combined in a single evolutionary framework. Therefore, a comprehensive study of the origin and diversification of the *SET* gene family in insects is required. Accurate classification of *SET*-domain containing genes can pave the fundamental way to further understanding the epigenetic basis of gene regulation in insects.

Evolution of *SET* Genes in Insects

In the present study, we aimed to ascertain the origin and diversification of *SET* genes in insects. We searched for *SET* genes in the 130 insect genomes and the 17 arthropod genomes as outgroups. These 130 insect species include both hemimetabolous and holometabolous insects and cover all the insect species for which genome data have been fully available and annotated so far. Our phylogenetic analysis revealed that an important diversification of arthropod-specific *SET* genes, *SmydA*, occurred during insect evolution. Experimental evidence of the important functions of *SmydA* genes in insects was obtained through fluorescence *in situ* hybridization, *in vitro* methyltransferase activity assay, and survival assay after expression knockdown. Furthermore, we compared the gene expression patterns and examined the selection signatures of *SmydA* genes in the four representative insects exhibiting phenotypic plasticity. These results provides insights into the regulatory roles of lineage-specific domain duplication in insect evolution.

Results

Identification and phylogenetic classification of *SET* genes

We comprehensively searched for *SET* genes in a wide range of sequenced insect species, which included 130 insect species from 14 insect orders (Supplementary Table S1). The *SET* genes were defined by the presence of the *SET* domain as predicted by the HMMER search, and their gene models were manually improved. Seventeen non-insect arthropods were also included to achieve ancestral status along with insect evolution. In total, 4,498 *SET* genes were identified in the 147 arthropod genomes (Supplementary Table S2). The genes showing potential pseudogene signals were removed in these identified *SET* genes. A database webserver

(http://159.226.67.242:8080/) has been constructed to select, retrieve, and analyze the data in this study. In insects, the number of *SET* genes found per species ranges from 16 in the scuttle fly *Megaselia scalaris* to 81 in the mosquito *Culex quinquefasciatus* (Table 1 and see Supplementary Table S3 for the full list of summary of *SET* genes in the 147 arthropod genomes). This observation suggests that the size of *SET* genes varies significantly among different insect lineages Although the genome size of the migratory locust *Locusta migratoria* is approximately 30-fold that of the fruit fly *Drosophila melanogaster* [29] , the number of *SET* genes in locusts is comparable with that of flies. Thus, the genome sizes and number of SET genes are not linearly correlated. The specificity of certain substrates is reflected by the classification of *SET* genes, and *SET* genes can be classified into seven major conserved groups, namely: Suv, Ash, Trx, E(z), PRDM, SMYD, and SETD [24]. We performed phylogenetic analysis of the *SET* genes for representative species to obtain insights into the evolution of insect *SET* genes. Multiple sequence alignments of complete proteins could not accurately determine the homologous sites of *SET* genes because of the considerably different sequence lengths and domain architectures of these genes. Thus, alignment-based methods using Bayesian inferences for *SET* domain sequences and alignment-free methods based on feature frequency profiles for complete protein sequences were conducted to infer phylogenetic relationships. The overall tree topologies (Figure 1) inferred using the two methods were generally consistent. Based on the previous nomenclature system [24], the phylogenetic tree topology allows the grouping of insect *SET* genes into seven major conserved groups, generally showing slight fluctuation in the member sizes in each conserved group. The protein domains for each *SET* gene were annotated using the InterProScan package. In general, the

SET genes in the same conserved group exhibited a similar domain composition, suggesting that the domain architectures support the conserved group classification inferred through the phylogenetic analysis. In addition to the *SET* genes in the conserved groups, a large number of *SET* genes could not be classified into known conserved groups on the basis of the phylogenetic analysis. These unclassified genes act as potential “arthropod-specific” genes. The lineage-specificity was further verified through reciprocal BLAST search against known *SET* genes of nematodes and humans.

Ancestral states of the *SET* gene family in insects

A character matrix that represents the present/absent states for each *SET* homologous group was constructed to infer the ancestral states of interior nodes along with the species tree using the Mesquite program. The ancestral states at different nodes could infer the appearances/losses of the *SET* homologous group that occurred at and above the level of orders (Figure 2A). The grouping of *SET* homologous genes for each species was inferred using the OrthoMCL program with the corresponding orthologous *SET* gene in *D. melanogaster,* and the grouping reliability was supported by the phylogenetic analysis (Supplementary Figure S1–S5). The putative ancestral state was composed of 19 *SET* homologous groups present in the last common ancestor (LCA) of the studied arthropod species. Generally the insect species possessed more *SET* homologous groups than the chelicerata species studied, suggesting that *SET* homologous groups considerably expanded during insect evolution. At the interior clades, novel *SET* homologous groups emerged several times. Only few losses of *SET* homologous groups, such as the loss of *SmydA-3*, were

observed at the interior clades. The large fluctuation of *SET* homologous groups in each species indicates that these groups experienced rapid lineage-specific expansion/contraction within insect orders. For example, in Hymenoptera, the number of *SET* homologous groups ranged from 18 (covering 23 *SET* genes) in the jumping ant *Harpegnathos saltator* to 30 (covering 52 *SET* genes) in the parasitoid wasp *Nasonia vitripennis*. In Diptera, 13 *SET* homologous groups (covering 14 *SET* genes) were found in *M. scalaris*, and the oriental fruit fly *Bactrocera dorsalis* possessed only 31 *SET* homologous groups (covering 45 *SET* genes). A large number of arthropod specific *SET* homologous groups cannot be classified into the seven major conserved groups, which revealed their origin after the emergence of main arthropod lineages. Nevertheless, at least six of these groups were present among insect species belonging to different orders, indicating their broad conservation in insects (Figure 2B).

SET domains do not just function as an independent unit, as in many proteins it co-occurs with multiple other protein domains to regulate their target specificity and catalysis [16]. We surveyed the gene ontology (GO) classification of proteins by integrating biological knowledge into three hierarchies, namely, biological process, molecular function, and cellular component, to assess the function innovation of domain acquisition globally. The common GO categories included histone lysine methylation (GO:0034968), regulation of transcription (GO:0006355), protein binding (GO:0005515), nucleic acid binding (GO:0003676), and metal ion binding (GO:0046872) (Figure 3A). Partitioning of *SET* gene families between the conserved and arthropod specific groups revealed that GO categories could be shared between the two groups or be assigned exclusively to one group. The GO categories, which

were only exclusive in the arthropod specific groups, included RNA methyltransferase activity (GO:0008173), metallocarboxypeptidase activity (GO:0004181), lysozyme activity (GO:0003796), homophilic cell adhesion (GO:0007156), sulfotransferase activity (GO:0008146) and so on.

Emergence of arthropod lineage-specific *SET* gene families

Pairwise BLAST search against all the *SET* genes indicated that the arthropod specific *SET* genes showed considerable amino acid similarity to the SMYD groups, which contain a conserved core consisting of a *SET* domain and a MYND (Myeloid translocation protein, Nervy, Deaf) zinc finger domain [30]. The arthropod specific *SET* genes also contain the *SET* and MYND domains and were named *SmydA* [28]. We performed the phylogenetic analysis of the SMYD genes through Bayesian inferences. The majority of the SMYD genes could be classified into 11 monophyletic clades, which exhibited similar high Bayesian posterior probability values (Figure 3B). In a global view, these SMYD genes fell into two distinct branches, which correspond with the conserved SMYD and *SmydA* groups. These results could exclude the possibility that the *SmydA* groups have raised from multiple independent gain events by duplications from deeply diverged SMYD genes of insects. Indeed, *SmydA* genes were absent from in all Chelicerata species investigated but present in the genomes of crustacean species and insect species, suggesting that *SmydA* genes may have originated prior to the divergence of Crustacea and Insecta. *SmydA-1*, *SmydA-2*, *SmydA-3*, and *SmydA-6* were already present before the split of Crustacea with other insects, showing clues for their ancient duplication events. The strong support for distinct individual lineages of paralogous genes implied that multiple

duplications occurred within the order level; the most notable case was the detection of three copies of *SmydA-3* in the red flour beetle *Tribolium castaneum* (Supplementary Table 2). *SmydA-1/SmydA-4* and *SmydA-6* were subjected to additional rounds of duplication in Lepidoptera and Orthoptera, respectively. The genes annotated as *SmydA-8* and *SmydA-9* in *D. melanogaster* previously formed a single clade alone with a high Bayesian posterior probability value (0.99), suggesting a specific duplication event in *Drosophila*. Therefore, the *SmydA* groups differed considerably in the number of genes in each insect order, implying the complexity of their evolutionary histories.

To shed light into the evolutionary history of *SmydA* genes, we determined the location and gene order of *SmydA* genes in the four holometabolous species with available chromosome-level genome assemblies or genome-scale genetic linkage maps (Figure 3C). In Diptera, the syntenic gene orders could be inferred from the four ancient *SmydA* genes, namely, *SmydA-1*, *SmydA-2*, *SmydA-3*, and *SmydA-6*, all of which may have been present in the ancestor of insects and crustaceans. An insect-specific *SmydA-9* could be observed in the majority of insect orders, including both hemimetabolous and holometabolous insects. *SmydA-9* showed syntenic conservation with the four ancient genes. This gene order was also conserved when *SmydA* genes in insects distantly related from other insect orders were examined. Almost all of the five synteny-anchoring genes were maintained in both the coleopteran species *T. castaneum* and hymenoptera species *A. mellifera*, with an exception of *SmydA-2* that was missed in *A. mellifera*. In contrast to those in *T. castaneum* and *A. mellifera*, the reversed order of *SmydA-3* and *SmydA-6* in Dipteran species implies that an intrachromosome transfer event of genomic segments occurred

before the emergence of Diptera. Duplication events could also occur in the early diversification of arthropod species. No orthologous *SmydA-4* gene was detected the chelicerata species, indicating that duplication event contributes to the emergence of *SmydA-4* gene in Pancrustacea species. *SmydA-4* was present all the hemimetabolous insect orders studied, as well as in the holometabolous insect orders Lepidoptera, Coleoptera, and Diptera. The absence of *SmydA-4* in all the 32 hymenopteran species suggested that subsequent loss of *SmydA-4* could be traced back to the ancestor of the hymenopteran lineage before the divergence of wasp, ants, and bees. In the SMYD phylogenetic tree, the Bayesian inferences supported the grouping of *SmydA-3*, *SmydA-4*, and *SmydA-6*. Three of the four species exhibited a accordant location of *SmydA-3*/*SmydA-4/SmydA-6* in the syntenic regions. In addition to the old duplication events that categorized the divergent duplicates into distinct *SmydA* subfamilies (e.g., *SmydA-3* and *SmydA-4*), recent duplications within an insect order were also observed. The three copies of *SmydA-3* in *T. castaneum*, which spanned within a 4.2 kb genomic region, were observed in tandem array between the two syntenic genes *SmydA-1* and *SmydA-6*. The closeness in protein sequence and genomic location implies an evolutionary origin of these three copies of *SmydA-3* via local duplication. Overall, our data suggest that the order of *SmydA* genes was conserved over a remarkable wide range of holometabolous insect orders.

Selective pressures acting on *SmydA* genes

Functional differentiations or mutations leading to pseudogene formation were the two major causes for sequence divergence between new duplicates and their orthologous counterpart. Synonymous substitutions are assumed to accumulate at a

constant rate; hence, the ratios of nonsynonymous substitution per nonsynonymous site (d_N) to synonymous substitution per synonymous site (d_S) are deemed to be an indicator to measure the relative rates of evolution for protein sequences. The four genes (ACYPI26757 and ACYPI55839 in *Acyrthosiphon pisum*; Px015362.1 and Px001029.1 in *Plutella xylostella*) showing signals of recombination were removed from the further selection analysis. We estimated a global d_N/d_S ratio (one ratio, model M0) for these *SET* genes to determine whether the *SmydA* genes have been under different selection pressures than the other conserved *SET* genes. The d_N/d_S ratios ($\omega = d_N/d_S$ ratio) of *SET* genes varied from low (0.0007, Ez, CG6502) to high (0.1627, *Smyd4-1*, *CG1868*), indicating a variance in the rates of protein evolution on different *SET* genes (Table 2). The ω values among the conserved *SET* genes (excluding the SMYD genes) ranged from 0.0007 to 0.0624 (mean ω = 0.0185). The conserved SMYD and *SmydA* groups showed ω values in the ranges of 0.055–0.1627 (mean ω = 0.1020) and 0.0052–0.1623 (mean ω = 0.0884), respectively. Overall, both the conserved SMYD and *SmydA* (P = 0.0003 and P = 0.0178, Wilcoxon signed-rank tests with Bonferroni correction, respectively) groups exhibited significantly higher ω values than the conserved *SET* genes (Figure 3D). However, the distributions of ω values of the conserved SMYD and *SmydA* groups were statistically indistinguishable (P = 1.0000, Wilcoxon signed-rank tests with Bonferroni correction).

Function approval of *SmydA* genes

We attempted to determine whether the *SmydA* genes retained histone methylate activities to approve the non-pseudogenization process of these genes. We expressed *SmydA-2* as a randomly selected representative and performed *in vitro* histone

methylation activity assays using histones as substrates in the migratory locust. As shown in Figure 4A, Western blot analysis detected increased lysine methylation on histone H3 compared with the controls, indicating that *SmydA-2* possesses methyltransferase activity on histones. Similar to that of the other conserved SMYD genes, the methyltransferase activity of *SmydA-2* was also dependent on S-adenosyl methionine. Fluorescence *in situ* hybridization analysis provided further tissue expression evidence to support the reliability of the *SmydA-2* gene function. Obvious fluorescence signals were observed in the brain and epidermal cells of cuticle in the locusts (Figure 4B). These cells did not show any hybridization signal for the negative controls. The origin and evolution of new emerging genes undergo an increased expression breadth of new duplicated genes over evolutionary time [31, 32]. Thus, we determined the expression levels of the *SmydA-2* gene using quantitative real-time polymerase chain reaction (qPCR) analysis in the different tissues. qPCR data showed that the *SmydA-2* gene was expressed in a broad range of tissues, including brains, testes, ovaries, cuticles, and legs (Figure 4C). The broad expression pattern suggests that the *SmydA-2* gene is less tissue specific and may serve as a functional gene in multiple tissues [32].

To determine whether the *SmydA-2* gene plays an essential role during development [33], we knocked its expression down by using RNA interferences in the locusts. Compared with the controls, the relative mRNA level of the *SmydA-2* gene decreased by approximately 70% after injecting double-strand RNAs (Supplementary Figure S6). After injection of *dsSmydA-2*, we observed large numbers of dead locusts, which did not display obvious defect phenotype. As shown in Figure 4D, Kaplan–Meier survival estimates indicate that injection of locusts with *dsSmydA-2*

significantly increased mortality when compared with the controls ($\chi^2 = 6.260$, df = 1, $P = 0.0123$, Chi-square tests).

Expression and selection analysis of *SmydA* genes in response to phenotypic plasticity

Epigenetic reprogramming that modifies chromatin structure through histone modifiers contributes to orchestrate the generation and maintenance of phenotypic plasticity, which is a key trait for the success of insects. Therefore, we compared the expression patterns of histone-modifier *SET* genes in four representative insects exhibiting phenotypic plasticity, namely, locust density-dependent behavior, aphid seasonal morphs, dietary-mediated interactions of bees and ants. Specially, we performed differential expression analysis between gregarious and solitary locusts, between asexual and sexual morphs in *A. pisum*, between queens and workers in *A. mellifera*, and between large workers and queens in *Acromyrmex echinatior*. In all the four species, a number of differentially expressed genes (DEGs) were detected between the two alternative phenotypes using the criteria of a false discovery rate (FDR)-corrected $P < 0.05$. In terms of DEG number, a large portion of *SET* genes showed significant changes in gene expression (16 in 31, 52%, in *A. mellifera*; 25 in 59, 50%, in *A. pisum*;13 in 29, 45%, in *L. migratoria*; and 11 in 27, 41%, in *A. echinatio*r). Compared with those DEGs observed at the genome-wide level (DEGs in total), the sensitivities of DEG number in *SET* genes in the four insects were even prominent, emphasizing the important regulatory role of *SET* genes in phenotypic transition (*Ps* < 0.02, Chi-square tests). Overlapping of the differentially expressed *SET* genes derived from the same ortholog could provide a clue of their convergent function in phenotypic transition. We found three *SET* genes, namely, *Hmt4-20*, *Set2*,

and *SmydA-5*, showed significant changes in gene expression simultaneously in three of the four insect species studied.

Assuming that a non-pseudogene gene should not be randomly expressed, we compared the expression pattern of the duplication-derived *SmydA* genes to their derived ancestral SMYD genes in response to environment-dependent phenotypic plasticity (Figure 5). The majority of *SET* genes from the conserved SMYD (30 in 30 in total, 100%) and *SmydA* (13 in 17 in total, 76%) groups were expressed in at least one insect. No significant differences (P = 0.710, Chi-Square tests) in the number of expressed genes were observed between the two groups. A number of DEGs were detected in both the conserved SMYD and *SmydA* groups in the four insect species. All the two *SmydA* genes in *A. pisum* and all the four *SmydA* genes in *A. echinatio*r were also differentially expressed. We also obtained significant results in three of the six *SmydA* genes of *L. migratoria* and in two of the five *SmydA* genes of *A. mellifera* between the two alternative phenotypes. The DEG number in the *SmydA* groups did not show significant deviation from those in the conserved SMYD group in the four insects (*Ps* > 0.2, Fisher's exact tests). This result suggests that the *SmydA* genes might not be randomly expressed and that they did not represent as pseudogenes or transcriptional byproducts. Thus, the *SmydA* genes may preserve a regulatory role, indicating the function similarity to their ancestral SMYD genes.

The free ratio model of *SmydA* genes fitted the data significantly better than the one model (model M0) using likelihood ratio tests (*Ps* < 0.001), indicating heterogeneous rates of sequence evolution along the gene tree of *SmydA* genes. Therefore, we tested whether the differentially expressed *SmydA* genes between alternative phenotypes (foreground branches) evolved under different selective

pressures than those in the remaining branches (background branch) (Supplementary Figure S7). The branch model was much better supported by the data than the model M0 for *SmydA-5* in *A. mellifera* and *SmydA-1* in *L. migratoria* (Table 3). Fixing $\omega = 1$ for the foreground branch did not result in an improved fit over the branch model with the unconstrained foreground branch (the null neutral model and the alternative model). This result suggests that the ω values in the external branch were smaller than 1 for *SmydA-3* and *SmydA-5* in *A. mellifera*, *SmydA-1* in *L. migratoria*, and *SmydA-3* in *A. echinatio*r. Only *SmydA-1* in *L. migratoria* exhibited elevated ω values, and a branch-site model allowing heterogeneous ω values across sequences and branches identified four sites (5M, 11K, 93P, and 105C) under positive selection.

Discussion

In this study, the phylogenetic analyses allowed the subdivision of the insect *SET* genes into seven major conserved groups and one arthropod-specific *SmydA* group. We inferred many *SmydA* gene duplication events along insect evolution, suggesting an important diversification of the *SmydA* genes occurred during insect evolutionary processes. With the *SmydA-2* genes in locusts as representatives, the maintenance of essential gene function was confirmed from the experimental evidence of *in vitro* methyltransferase activity, *in situ* mRNA expression, and phenotypes after expression knockdown. Based on the examination of distribution pattern and selection signatures across insects, our data indicated that extensive pseudogenization unlikely occurred for the *SmydA* genes. Finally, the transcriptome analyses of the four insects showed that several *SmydA* genes are involved in insect phenotype plasticity, suggesting that

SmydA genes contributed novelties for insect adaptive evolution. This data suggest a role of diverged regulatory functions after their duplication in insects.

SmydA genes represent a class of arthropod-specific genes that are only present in the LCA of insect species and crustacean species, suggesting their origin after the split of chelicerates from Pancrustacea species. Conservation of five ancient *SmydA* genes in a wide range of species suggests they probably originated from duplication events of conserved SMYD genes predating the diversification of insects. Although a few cases of whole-genome duplication have been documented in chelicerates, evidence that whole-genome duplication occurs widely in arthropod evolution remains lacking [34]. Therefore, gene duplication rather than whole-genome duplication possibly leads to the emergence of multiple copies of ancient *SmydA* genes in the LCA of Pancrustacea species. The clear split of conserved SMYD and *SmydA* genes excluded the possibility that multiple independent duplication events from conserved SMYD genes resulted in the current repertoire of *SmydA* genes in insects. This result suggests that the five ancient *SmydA* genes were first produced from a single ancestral gene, which was derived from conserved SMYD genes. The five ancient *SmydA* genes were thus the source from which insect-specific *SmydA* duplications were subsequently produced in insects. Determining the location and order of multiple gene members at the genomic scale sheds light on the evolutionary history of gene family. The closely linked manner in genomic location suggests that homologous recombination and functional differentiation may be a major force to shape the evolution of *SmydA* genes in insects. For instance, in dipteran and lepidopteran insects, homologous recombination may give rise to *SmydA-6* via the duplication events of *SmydA-3* because *SmydA-3* and *SmydA-6* were in close proximity to each other in both genomic

location and phylogenetic trees. The tandem organization of three *SmydA-3* copies in *T. castaneum* may also result from species-specific duplications via homologous recombination. Retrotransposition events may represent another contributing force for generating unlinked *SmydA* genes; these events can also generate intronless retroposed gene copies [35]. However, the retrotransposition events could not be inferred from the presence of signature of intron–exon structure because of the subsequent insertion in deeply diverged duplicates, such as *SmydA-5*. Conserved gene orders between species from Lepidoptera, Coleoptera, and Diptera revealed a high degree of macrosyntenic gene order of the five ancient *SmydA* genes during approximately 348 million years of evolutions splitting these insects [36]. This observation implies strong constraints for preserving the conserved gene order of *SmydA* genes in insects. Currently, whether this macro-syntenic gene order is preserved outside holometabolous insects cannot be determined because chromosome-level genome assemblies or genome-scale genetic linkage maps are not available in hemimetabolous insects. This issue would be addressed when the genome assembly is considerably improved in the future.

Selective pressures were significantly weaker for the SMYD genes than for the six conserved groups (Suv, Ash, Trx, E(z), PRDM, and SETD). Compared with the six conserved groups, SMYD genes were the least conserved gene group and, concordantly, the least constrained one. Nevertheless, the ω values of SMYD genes ranged from 0.0052 for *SmydA-2* to 0.1627 for *Smyd4-1*. $\omega << 1$ was consistent with their broad conservation across insects, implying their essential functional roles. This observation suggests that purifying selection is the main force governing the evolution of SMYD genes. The distributions of ω values of the conserved SMYD and *SmydA*

gens were statistically indistinguishable, indicating a symmetrical rate of sequence evolution. Thus, purifying selection is subject to the conserved SMYD and *SmydA* genes, but their intensity may be relaxed compared with other *SET* genes. Both the GO analysis and the *in vitro* methyltransferase activity assay suggest that *SmydA* genes, similar to their conserved SMYD ancestors, are sufficient to perform the original function relating to histone methylation [37]. GO ontology analysis implied that the *SmydA* genes have developed to acquire novel functions. These functions were absent in the conserved SMYD genes, indicating that the *SmydA* genes may have undergone functional differentiation. Gene duplications that occurred in specific lineages are important in contributing to lineage-specific adaptive processes [38]. After gene duplication, purifying selection is expected in both gene copies if duplication can confer a selective advantage [7]. By contrast, one of the two copies can evolve either under relaxed purifying selection when no immediate advantage is shown from gene duplication or under positive selection when a new function is acquired via advantageous mutations [39]. Overall, these data suggest that the *SmydA* genes may not represent redundant gene copies that are under pseudogenization.

Several members of the SMYD family of histone methyltransferases have undergone a dramatic expansion in the insect lineage [27]. These SMYD genes were identified as caste-specific genes in ants (*Harpegnathos saltator*), suggesting that these histone modifiers play dedicated regulatory roles in insect phenotypic plasticity. However, the biological significance of the differential expressions of these genes remains unknown [40]. Our study further verified the presence of the differential expression patterns of the SMYD genes in the four other insects that also possessed adaptive phenotypic plasticity. Consequently, the understanding of convergent

regulatory roles of the SMYD genes in insect phenotypic plasticity was extended. Histone lysine methyltransferase catalyzes methyl group transfer to the amino group of lysine residues of histones by means of the *SET* domain, a domain presented within many proteins that regulate diverse development processes [41]. Histone lysine methylation on specific residues is associated with distinct signatures of gene expression, thereby serving as a chromatin modulator for epigenetic regulation [42]. Future studies should understand how the expanded SMYD gene family can quickly become essential and identify the roles of the duplicated SMYD genes in insects, despite the expectation of redundant functionality at the beginning of new duplicated gene evolution [33].

Materials and Methods

Identification of insect *SET* genes

Genome assemblies and official gene sets of 130 insect species, including 62 dipteran insects, 33 hymenopteran insects, 10 hemipteran insects, 7 coleopteran species, 9 lepidopteran insects, and representatives from Orthoptera, Phthiraptera, Phasmatoptera, Trichoptera, Thysanoptera, Isoptera, Blattodea, Ephemeroptera and Odonata, were obtained from their respective genome databases (Supplementary Table S1). Among the basal arthropod species, we included 17 arthropod genomes from 10 chelicerate species, five crustacean species and two non-insect hexapod species.

The hidden Markov model-based HMMER program was used to identify the SET domain containing proteins using PF00856 in the Pfam database [43, 44]. The resulting genes with stop codons or frameshift mutations were subsequently manually

checked. The obvious incorrect gene models were improved with transcriptome data through the GeneWise program [45]. The PSILC program was used to identify the potential pseudogenes [46]. Gene Ontology (GO) categories were determined via scanning protein sequences against Interpro member databases using various profile-based and hidden Markov models in the InterProScan package [47].

Phylogenetic analysis, ancestral state reconstructions, and tests for selection

Alignment-based methods using Bayesian inferences for *SET* domain sequences and alignment-free methods based on feature frequency profiles for complete protein sequences were used to infer phylogenetic relationships of *SET* genes across insects. Multiple alignments were generated using the MAFFT alignment software [48]. According to the Akaike information criterion, the model of molecular evolution with the best fit to the data was determined by using the ProtTest software [49]. Bayesian reconstruction of phylogeny was conducted using the MrBayes 3.2.1 software for 10,000,000 generations [50]. The first 25% of the trees were discarded as burn-in. The alignment-free and distance-based methods for phylogenetic tree building were implemented by means of the feature frequency profile method (http://sourceforge.net/projects/ffp-phylogeny/).

We constructed a character matrix that represents present/absent states for each *SET* gene family to reconstruct the ancestral states of interior clades. The grouping of the *SET* gene family was inferred from the OrthoMCL software with the corresponding orthologous *SET* gene in *D. melanogaster*. Ancestral state reconstruction was implemented in the Mesquite program using maximum likelihood approaches (http://mesquiteproject.org/). This process requires a phylogeny tree of all

the species studied. Single-copy orthologous gene families were inferred from the core eukaryote CEGMA gene sets from each species using the OrthoMCL software [51]. The resulting 455 single-copy orthologous gene families were used to construct the species tree, which is consistent with the phylogenomic tree recently inferred from transcriptome data [18].

Expression of SMYD family genes in response to phenotypic plasticity

The transcriptome data for gregarious and solitary locusts in *L. migratoria*, asexual and sexual morphs in *A. pisum*, queens and workers in *A. mellifera*, and minor and major workers in *A. echinatior* were retrieved from the NCBI database under accession numbers PRJNA79681, GSE56830, GSE61253, and GSE51576, respectively. The raw reads were preprocessed to remove adapters and low-quality bases using the Trimmomatic software; these reads were then mapped to the genome assembly using the Tophat2 software [52, 53]. Raw counts of each gene were calculated and annotated using the HT-seq package in Python, and the trimmed mean of M value normalization method was used to normalize raw counts [54]. Differential expression analysis was performed using the edgeR package at an FDR cut-off of 0.05 [55].

Function approval of *SmydA-2* genes via experimental evidence

A fluorescence *in situ* analysis of *SmydA-2* was performed on the brains and integuments of locust nymphs. Biotin-labeled antisense and sense probes of *SmydA-2* were produced from pGEM-T Easy plasmids (Promega) by using the T7/SP6 RNA

transcription system (Roche) following the manufacturer's protocol. The brains and integuments were fixed in 4% paraformaldehyde overnight. The paraffin-embedded slides (5 µm thick) were deparaffinized in xylene, rehydrated with an ethanol gradient, digested with 20 µg/mL proteinase K (Roche) at 37 °C for 15 min, and then incubated with *SmydA-2* probe at 60 °C for 5 min. The slides were hybridized for 7–15 h at 37 °C and washed in 0.2×SSC and 2% BSA at 4 °C for 5 min. The biotin-labeled probes of *SmydA-2* were detected with a streptavidin horseradish peroxidase conjugate and fluorescein tyramide substrate using a TSA kit (Perkin Elmer). Images for fluorescence signals were acquired using an LSM 710 confocal fluorescence microscope (Zeiss).

The recombinant proteins for *SmydA-2* and the negative controls of translation system were produced using the TNT protein expression system (Promega). For *in vitro* methyltransferase assay, 2 mg of unmodified histone H3 peptides (Sino Biological) were incubated with 1 mg of recombinant protein and 0.1 mM S-adenosyl-methionine (SAM, NEB) in a reaction buffer containing 50 mM Tris-HCl (pH 8.0), 10% glycerol, 20 mM KCl, 5 mM $MgCl_2$, 1 mM DTT, and 1 mM PMSF at 30 °C for 2 h. The reaction mixtures were subjected to electrophoresis on SDS-PAGE, and the methylation activities were detected in Western blotting using anti-pan methyl lysine antibody (Abcam). Anti-histone H3 (Abcam) was used as endogenous control for protein samples.

Locusts were reared in large, well-ventilated cages (40 cm × 40 cm × 40 cm) at a density of 500–1000 insects per container. These colonies were reared under a 14:10 light/dark photo regime at 30 °C and were fed fresh wheat seedlings and bran. Double-stranded RNAs of *SmydA-2* and green fluorescent protein (GFP) were

prepared using the T7 RiboMAX Express RNAi system (Promega) in accordance with the manufacturer's protocols. Second-instar locusts were injected with double-stranded RNAs in the second ventral segment of the abdomen. Total RNAs were isolated using TRIzol reagent (Thermo Fisher Scientific) and then reverse-transcribed into cDNA using M-MLV reverse transcriptase (Promega). The mRNA levels were quantified using the SYBR Green expression assays on a LightCycler 480 instrument (Roche). Survival data were analyzed using the Kaplan–Meier method, and survival curves were compared using log-rank testing for the *dsSmydA-2* and *dsGFP* curves.

Signature of selection detected through likelihood ratio tests

Protein sequences of *SET* genes were aligned with the MAFFT alignment software [48] and the back-translated into corresponding nucleotide sequences. Gene conversion was detected using the recombination detection program GENECONV version 1.81a. To assess the contribution of natural selection during the diversification of the *SET* gene family in insects, the ratios of nonsynonymous substitution per nonsynonymous site (d_N) to synonymous substitution per synonymous site (d_S) across the phylogenetic tree of the species were calculated using the software package PAML version 4.48a [56]. The basic model M0 (null model) assumes the ratio $\varpi = d_N/d_S$ is invariable (one-ratio model) among all branches examined, whereas the alternative model allows the ϖ ratio to vary in different tree branches in the phylogenetic tree [57, 58]. Likelihood ratio tests were applied to compare the null and alternative models, which estimated ϖ ratio separately for different branches, assuming a priori and the background branches. A significantly higher likelihood of

the alternative model than the null model indicates a better fit to the data, indicating a variation of selective pressures in different tree branches [57, 58].

Declarations

List of abbreviations

SET genes, *SET* domain-containing genes; E(z), Enhancer of zeste; LCA, last common ancestor; GO, gene ontology; MYND, Myeloid translocation protein; qPCR, quantitative real-time polymerase chain reaction; DEGs, differentially expressed genes; FDR, false discovery rate; SAM, S-adenosyl-methionine; GFP, green fluorescent protein; PP, posterior probability

Ethics approval and consent to participate

All animal procedures were licensed under the Institutional Animal Care and Use Committee of the Institute of Zoology, Chinese Academy of Sciences.

Consent for publication

Not applicable

Competing interests

The authors declare they have no competing interests.

Funding

This research was supported by the following grants: National Natural Science Foundation of China Grants (Nos. 31301915, 31472051, and 31672353) and Strategic Priority Research Program of the Chinese Academy of Sciences (No. XDB11010000).

Authors' contributions

F.J., X.W., and L.K conceived and designed the experiments. F.J. and Q. L analyzed and interpreted the data. F.J., Q. L., Y.W., J.Z., H.W., T.S., and M.Y. performed the experiments. F.J., Q.L., and L.K wrote the paper.

Acknowledgements

The computational resources were provided by the Research Network of Computational Biology and the Supercomputing Center at Beijing Institutes of Life Science, Chinese Academy of Sciences.

Availability of supporting data and materials

The dataset supporting the conclusions of this article is available in http://159.226.67.242:8080/.

References

1. Elofsson A, Sonnhammer EL: **A comparison of sequence and structure protein domain families as a basis for structural genomics**. *Bioinformatics* 1999, **15**(6):480-500.
2. Itoh M, Nacher JC, Kuma K, Goto S, Kanehisa M: **Evolutionary history and functional implications of protein domains and their combinations in eukaryotes**. *Genome Biol* 2007, **8**(6):R121.
3. Sakarya O, Conaco C, Egecioglu O, Solla SA, Oakley TH, Kosik KS: **Evolutionary expansion and specialization of the PDZ domains**. *Mol Biol Evol* 2010, **27**(5):1058-1069.

4. Johnson BR, Tsutsui ND: **Taxonomically restricted genes are associated with the evolution of sociality in the honey bee**. *BMC Genomics* 2011, **12**:164.

5. Qian W, Zhang J: **Genomic evidence for adaptation by gene duplication**. *Genome Res* 2014, **24**(8):1356-1362.

6. Lynch M, Conery JS: **The evolutionary fate and consequences of duplicate genes**. *Science* 2000, **290**(5494):1151-1155.

7. Innan H, Kondrashov F: **The evolution of gene duplications: classifying and distinguishing between models**. *Nat Rev Genet* 2010, **11**(2):97-108.

8. Moore AD, Bornberg-Bauer E: **The dynamics and evolutionary potential of domain loss and emergence**. *Mol Biol Evol* 2012, **29**(2):787-796.

9. Simola DF, Graham RJ, Brady CM, Enzmann BL, Desplan C, Ray A, Zwiebel LJ, Bonasio R, Reinberg D, Liebig J *et al*: **Epigenetic (re)programming of caste-specific behavior in the ant Camponotus floridanus**. *Science* 2016, **351**(6268):aac6633.

10. Spannhoff A, Kim YK, Raynal NJ, Gharibyan V, Su MB, Zhou YY, Li J, Castellano S, Sbardella G, Issa JP *et al*: **Histone deacetylase inhibitor activity in royal jelly might facilitate caste switching in bees**. *EMBO Rep* 2011, **12**(3):238-243.

11. Simola DF, Ye C, Mutti NS, Dolezal K, Bonasio R, Liebig J, Reinberg D, Berger SL: **A chromatin link to caste identity in the carpenter ant Camponotus floridanus**. *Genome Res* 2013, **23**(3):486-496.

12. Dickman MJ, Kucharski R, Maleszka R, Hurd PJ: **Extensive histone post-translational modification in honey bees**. *Insect Biochem Mol Biol* 2013, **43**(2):125-137.

13. Jenuwein T, Laible G, Dorn R, Reuter G: **SET domain proteins modulate chromatin domains in eu- and heterochromatin**. *Cell Mol Life Sci* 1998, **54**(1):80-93.

14. Dillon SC, Zhang X, Trievel RC, Cheng X: **The SET-domain protein superfamily: protein lysine methyltransferases**. *Genome Biol* 2005, **6**(8):227.

15. Boros IM: **Histone modification in Drosophila**. *Brief Funct Genomics* 2012, **11**(4):319-331.

16. Herz HM, Garruss A, Shilatifard A: **SET for life: biochemical activities and biological functions of SET domain-containing proteins**. *Trends Biochem Sci* 2013, **38**(12):621-639.

17. Jenuwein T: **The epigenetic magic of histone lysine methylation**. *FEBS J* 2006, **273**(14):3121-3135.

18. Misof B, Liu S, Meusemann K, Peters RS, Donath A, Mayer C, Frandsen PB, Ware J, Flouri T, Beutel RG *et al*: **Phylogenomics resolves the timing and pattern of insect evolution**. *Science* 2014, **346**(6210):763-767.

19. Ferguson LC, Green J, Surridge A, Jiggins CD: **Evolution of the insect yellow gene family**. *Mol Biol Evol* 2011, **28**(1):257-272.

20. Helmkampf M, Cash E, Gadau J: **Evolution of the insect desaturase gene family with an emphasis on social Hymenoptera**. *Mol Biol Evol* 2015, **32**(2):456-471.

21. Tanaka K, Diekmann Y, Hazbun A, Hijazi A, Vreede B, Roch F, Sucena E: **Multispecies Analysis of Expression Pattern Diversification in the**

Recently Expanded Insect Ly6 Gene Family. *Mol Biol Evol* 2015, **32**(7):1730-1747.

22. Urena E, Pirone L, Chafino S, Perez C, Sutherland JD, Lang V, Rodriguez MS, Lopitz-Otsoa F, Blanco FJ, Barrio R *et al*: **Evolution of SUMO Function and Chain Formation in Insects**. *Mol Biol Evol* 2016, **33**(2):568-584.
23. Benton R: **Multigene Family Evolution: Perspectives from Insect Chemoreceptors**. *Trends Ecol Evol* 2015, **30**(10):590-600.
24. Zhang L, Ma H: **Complex evolutionary history and diverse domain organization of SET proteins suggest divergent regulatory interactions**. *The New phytologist* 2012, **195**(1):248-263.
25. Vidal NM, Grazziotin AL, Iyer LM, Aravind L, Venancio TM: **Transcription factors, chromatin proteins and the diversification of Hemiptera**. *Insect Biochem Mol Biol* 2016, **69**:1-13.
26. Rider SD, Jr., Srinivasan DG, Hilgarth RS: **Chromatin-remodelling proteins of the pea aphid, Acyrthosiphon pisum (Harris)**. *Insect Mol Biol* 2010, **19 Suppl 2**:201-214.
27. Bonasio R, Zhang G, Ye C, Mutti NS, Fang X, Qin N, Donahue G, Yang P, Li Q, Li C *et al*: **Genomic comparison of the ants Camponotus floridanus and Harpegnathos saltator**. *Science* 2010, **329**(5995):1068-1071.
28. Calpena E, Palau F, Espinos C, Galindo MI: **Evolutionary History of the Smyd Gene Family in Metazoans: A Framework to Identify the Orthologs of Human Smyd Genes in Drosophila and Other Animal Species**. *PLoS One* 2015, **10**(7):e0134106.
29. Wang X, Fang X, Yang P, Jiang X, Jiang F, Zhao D, Li B, Cui F, Wei J, Ma C *et al*: **The locust genome provides insight into swarm formation and long-distance flight**. *Nat Commun* 2014, **5**:2957.
30. Thompson EC, Travers AA: **A Drosophila Smyd4 homologue is a muscle-specific transcriptional modulator involved in development**. *PLoS One* 2008, **3**(8):e3008.
31. Kaessmann H: **Origins, evolution, and phenotypic impact of new genes**. *Genome Res* 2010, **20**(10):1313-1326.
32. Assis R, Bachtrog D: **Neofunctionalization of young duplicate genes in Drosophila**. *Proc Natl Acad Sci U S A* 2013, **110**(43):17409-17414.
33. Kemkemer C, Long M: **New genes important for development**. *EMBO Rep* 2014, **15**(5):460-461.
34. Kenny NJ, Chan KW, Nong W, Qu Z, Maeso I, Yip HY, Chan TF, Kwan HS, Holland PW, Chu KH *et al*: **Ancestral whole-genome duplication in the marine chelicerate horseshoe crabs**. *Heredity (Edinb)* 2016, **116**(2):190-199.
35. Kaessmann H, Vinckenbosch N, Long M: **RNA-based gene duplication: mechanistic and evolutionary insights**. *Nat Rev Genet* 2009, **10**(1):19-31.
36. Hedges SB, Dudley J, Kumar S: **TimeTree: a public knowledge-base of divergence times among organisms**. *Bioinformatics* 2006, **22**(23):2971-2972.
37. Tan X, Rotllant J, Li H, De Deyne P, Du SJ: **SmyD1, a histone methyltransferase, is required for myofibril organization and muscle contraction in zebrafish embryos**. *Proc Natl Acad Sci U S A* 2006, **103**(8):2713-2718.

38. Domazet-Loso T, Tautz D: **An evolutionary analysis of orphan genes in Drosophila**. *Genome Res* 2003, **13**(10):2213-2219.
39. Pegueroles C, Laurie S, Alba MM: **Accelerated evolution after gene duplication: a time-dependent process affecting just one copy**. *Mol Biol Evol* 2013, **30**(8):1830-1842.
40. Bonasio R: **The role of chromatin and epigenetics in the polyphenisms of ant castes**. *Brief Funct Genomics* 2014, **13**(3):235-245.
41. Leinhart K, Brown M: **SET/MYND Lysine Methyltransferases Regulate Gene Transcription and Protein Activity**. *Genes (Basel)* 2011, **2**(1):210-218.
42. Sims RJ, 3rd, Nishioka K, Reinberg D: **Histone lysine methylation: a signature for chromatin function**. *Trends Genet* 2003, **19**(11):629-639.
43. Finn RD, Mistry J, Tate J, Coggill P, Heger A, Pollington JE, Gavin OL, Gunasekaran P, Ceric G, Forslund K *et al*: **The Pfam protein families database**. *Nucleic Acids Res* 2010, **38**(Database issue):D211-222.
44. Finn RD, Clements J, Eddy SR: **HMMER web server: interactive sequence similarity searching**. *Nucleic Acids Res* 2011, **39**(Web Server issue):W29-37.
45. Birney E, Clamp M, Durbin R: **GeneWise and Genomewise**. *Genome Res* 2004, **14**(5):988-995.
46. Coin L, Durbin R: **Improved techniques for the identification of pseudogenes**. *Bioinformatics* 2004, **20 Suppl 1**:i94-100.
47. Jones P, Binns D, Chang HY, Fraser M, Li W, McAnulla C, McWilliam H, Maslen J, Mitchell A, Nuka G *et al*: **InterProScan 5: genome-scale protein function classification**. *Bioinformatics* 2014, **30**(9):1236-1240.
48. Katoh K, Asimenos G, Toh H: **Multiple alignment of DNA sequences with MAFFT**. *Methods Mol Biol* 2009, **537**:39-64.
49. Darriba D, Taboada GL, Doallo R, Posada D: **ProtTest 3: fast selection of best-fit models of protein evolution**. *Bioinformatics* 2011, **27**(8):1164-1165.
50. Ronquist F, Teslenko M, van der Mark P, Ayres DL, Darling A, Hohna S, Larget B, Liu L, Suchard MA, Huelsenbeck JP: **MrBayes 3.2: efficient Bayesian phylogenetic inference and model choice across a large model space**. *Syst Biol* 2012, **61**(3):539-542.
51. Li L, Stoeckert CJ, Jr., Roos DS: **OrthoMCL: identification of ortholog groups for eukaryotic genomes**. *Genome Res* 2003, **13**(9):2178-2189.
52. Bolger AM, Lohse M, Usadel B: **Trimmomatic: a flexible trimmer for Illumina sequence data**. *Bioinformatics* 2014, **30**(15):2114-2120.
53. Trapnell C, Pachter L, Salzberg SL: **TopHat: discovering splice junctions with RNA-Seq**. *Bioinformatics* 2009, **25**(9):1105-1111.
54. Robinson MD, Oshlack A: **A scaling normalization method for differential expression analysis of RNA-seq data**. *Genome Biol* 2010, **11**(3):R25.
55. Robinson MD, McCarthy DJ, Smyth GK: **edgeR: a Bioconductor package for differential expression analysis of digital gene expression data**. *Bioinformatics* 2010, **26**(1):139-140.
56. Yang Z: **PAML 4: phylogenetic analysis by maximum likelihood**. *Mol Biol Evol* 2007, **24**(8):1586-1591.
57. Yang Z: **Likelihood ratio tests for detecting positive selection and application to primate lysozyme evolution**. *Mol Biol Evol* 1998, **15**(5):568-573.

58. Yang Z, Nielsen R: **Synonymous and nonsynonymous rate variation in nuclear genes of mammals**. *J Mol Evol* 1998, **46**(4):409-418.

Figures

Figure 1. Phylogenetic analysis of *SET* genes in insects. A phylogeny using Bayesian inference is generated from the domain protein sequence of *SET* genes. One representative is elected for each order. The protein domains, which are labeled with different colors based on the domain type, are shown in the exterior circle of the phylogenetic tree.

Figure 2. Diversification of arthropod-specific *SET* genes. (A) Inference of ancestral sets of *SET* homologous groups along the evolution of insects. The gains and losses of *SET* homologous groups are indicated in the internal nodes of the phylogenetic tree. The number in parentheses indicates the number of species in each order. (B) Distribution pattern of *SET* genes in arthropod orders. One representative is elected for each order. Red color indicates presence of *SET* genes, and blue color indicates absence of *SET* genes.

Figure 3. Evolution of *SmydA* genes in insects. (A) Gene ontology categories of the conserved and arthropod-specific groups of *SET* genes. The gene ontology categories, which are only present in the arthropod-specific group, are highlighted in red. (B) Phylogenetic tree of the SMYD gene family of the representative species selected from each order. The phylogenetic tree is constructed using the Bayesian inference method. The Bayesian posterior probability (PP) values are indicated only for the

internal nodes to improve clarity; consequently, the *SET* genes are grouped into different monophyletic clades (SMYD subfamilies). Red and orange circles indicate PP > 90% and PP > 70%, respectively. (C) Conserved syntenies for *SmydA* genes in four holometabolous species. (D) Distributions of ω ($\omega = d_N/d_S$ ratio) values of the conserved SMYD and *SmydA* groups of *SET* genes.

Figure 4. Function approval of *SmydA-2* genes through experimental evidence. (A) *In vitro* methyltransferase assay of histone H3 of *SmydA-2* in locusts. Anti-pan methyl lysine antibody recognizes histone H3 *in vitro* methylated with *SmydA-2*. Anti-histone H3 serves as endogenous control for protein samples. The analyses were carried out in three replicates. $^{**}P < 0.01$. (B) Expression evidence of *SmydA-2* in the brain and cuticle of locusts via fluorescence *in situ* hybridization analysis. Green signals indicate the expression of *SmydA-2* /control, and blue signals indicate nuclear staining with Hoechst. (C) Relative gene expression of *SmydA-2* in the different tissues. mRNA levels are quantified using the SYBR Green expression assays on a LightCycler 480 instrument. The qPCR data are shown as the mean ± SEM ($n = 6$). (D) Survival analysis of the locusts after *SmydA-2* double-strand RNA injection. Data are analyzed through the Kaplan–Meier survival curve comparison of the *dsSmydA-2* and *dsGFP* groups for three replicates.

Figure 5. Differential expression analysis in insects showing phenotype plasticity. Alternative phenotype includes gregarious and solitary phases in *Locusta migratoria* (LOCMI), asexual and sexual morphs in *Acyrthosiphon pisum* (ACYPI), queens and

workers in *Apis mellifera* (APIME), and minor workers and major workers in *Acromyrmex echinatior* (ACREC).

Tables

Table 1. Summary of *SET* genes in insect genomes.

Table 2. Tests of rate heterogeneity acting on *SET* genes in insects.

Table 3. Signatures of selection acting on differentially expressed *SET* genes in response to phenotypic plasticity.

Supplementary Data

Supplementary Table S1. The arthropod genome data involved in this study.

Supplementary Table S2. *SET* genes in the 147 arthropod genomes.

Supplementary Table S3. Summary of *SET* genes in the 147 arthropod genomes.

Supplementary Figure S1. Phylogenetic analysis of the *SET* genes in Lepidoptera using Bayesian inferences. The *SET* gene families labeled with different colors are shown in the exterior circle of the phylogenetic tree. The insect species involved are represented with different colors of the external branch.

Supplementary Figure S2. Phylogenetic analysis of the *SET* genes in Diptera using Bayesian inferences. The *SET* gene families labeled with different colors are shown in the exterior circle of the phylogenetic tree. The insect species involved are represented with different colors of the external branch. The representative species are selected to improve clarity.

Supplementary Figure S3. Phylogenetic analysis of the *SET* genes in Hemiptera using Bayesian inferences. The *SET* gene families labeled with different colors are

shown in the exterior circle of the phylogenetic tree. The insect species involved are represented with different colors of the external branch.

Supplementary Figure S4. Phylogenetic analysis of the *SET* genes in Hymenoptera using Bayesian inferences. The *SET* gene families labeled with different colors are shown in the exterior circle of the phylogenetic tree. The insect species involved are represented with different colors of the external branch. The representative species are selected to improve clarity.

Supplementary Figure S5. Phylogenetic analysis of the *SET* genes in Coleopteran using Bayesian inferences. The *SET* gene families labeled with different colors are shown in the exterior circle of the phylogenetic tree. The insect species involved are represented with different colors of the external branch.

Supplementary Figure S6. Effects of RNA interference of the mRNA expression levels of *SmydA-2* in locust brains. The locusts are injected with double-stranded RNAs into the second ventral segment of the abdomen. Due to the systemic RNA interference in locusts, the brain, which is spatially distant from the abdomen, is used in qPCR assays to guarantee effective expression knockdown. qPCR data are shown as the mean ± SEM (n = 6). **P < 0.01.

Supplementary Figure S7. Tree topology and branch labeling for tests of selection on *SET* genes. APIME, *Apis mellifera*; ACREC, *Acromyrmex echinatior*; LOCMI, *Locusta migratoria*. Supplementary Table S1 presents the abbreviation of insect species.

Table 1

Click here to download Table Table1.2016102701.xls

Table 1. Summary of *SET* genes in insect genomes.

Order	Genus	SMYD	SETD	PRDM	Ash	Suv	Trx	Ez	AS	Total
Coleoptera	*Agrilus* (1)	4	1	2	3	3	3	1	9	26
Coleoptera	*Anoplophora* (1)	7	1	2	3	3	3	2	7	28
Coleoptera	*Dendroctonus* (1)	5	1	1	3	3	3	1	12	29
Coleoptera	*Leptinotarsa* (1)	10	1	1	2	5	3	1	9	32
Coleoptera	*Onthophagus* (1)	4	1	1	3	4	3	1	10	27
Coleoptera	*Oryctes* (1)	6	1	1	3	3	1	1	9	25
Coleoptera	*Tribolium* (1)	6	2	1	3	3	3	1	15	34
Phthiraptera	*Pediculus* (1)	6	1	1	3	4	3	1	9	28
Blattodea	*Blattella* (1)	4	2	2	4	3	2	1	7	25
Diptera	*Aedes* (2)	11-12	1	2	3-4	2-3	3-4	1-2	11-12	34-38
Diptera	*Anopheles* (19)	6-19	1	1-2	1-3	2-3	2-3	1	4-11	20-37
Diptera	*Bactrocera* (2)	4-5	1	1-2	3-4	4	3-6	1-2	13-22	31-45
Diptera	*Ceratina* (1)	5	1	1	2	4	3	1	11	28
Diptera	*Ceratitis* (1)	5	1	1	3	3	3	1	14	31
Diptera	*Culex* (1)	40	1	1	13	2	9	1	14	81
Diptera	*Drosophila* (22)	4-5	1	1	3-4	3-5	2-4	1	7-14	24-31
Diptera	*Glossina* (6)	4-5	1	1	3-4	2-5	3-4	1	12-15	29-34
Diptera	*Lucilia* (1)	5	1	1	3	3	3	1	12	29
Diptera	*Lutzomyia* (1)	6	1	1	3	3	2	1	10	27
Diptera	*Mayetiola* (1)	13	1	1	9	6	4	1	25	60
Diptera	*Megaselia* (1)	2	1	1	3	2	1	1	5	16
Diptera	*Musca* (1)	5	1	1	3	3	3	1	20	37
Diptera	*Phlebotomus* (1)	5	1	1	4	3	3	1	6	24
Diptera	*Belgica* (1)	27	2	1	3	5	4	1	12	55
Diptera	*Stomoxys* (1)	5	1	1	3	2	3	1	16	32
Ephemeroptera	*Ephemera* (1)	18	1	1	3	3	2	1	12	42
Hemiptera	*Acyrthosiphon* (1)	14	1	0	2	10	4	1	31	63
Hemiptera	*Cimex* (1)	4	1	2	3	5	3	1	5	24
Hemiptera	*Diaphorina* (1)	3	1	1	4	4	3	2	11	29
Hemiptera	*Gerris* (1)	6	1	1	3	3	3	1	8	26
Hemiptera	*Halyomorpha* (1)	5	1	1	2	5	3	1	8	26
Hemiptera	*Homalodisca* (1)	5	2	2	2	5	4	1	8	29
Hemiptera	*Nilaparvata* (1)	4	1	6	2	4	4	1	7	29
Hemiptera	*Oncopeltus* (1)	6	1	1	2	5	4	1	7	27
Hemiptera	*Pachypsylla* (1)	1	1	2	2	3	1	1	9	20
Hemiptera	*Rhodnius* (1)	6	1	1	2	2	2	1	6	21
Hymenoptera	*Acromyrmex* (1)	7	2	1	3	3	3	1	7	27
Hymenoptera	*Apis* (3)	6-7	1	1	3	3-4	1-3	1	7-9	22-29
Hymenoptera	*Athalia* (1)	7	1	2	2	3	2	1	8	26
Hymenoptera	*Atta* (1)	8	1	1	3	4	3	1	7	28
Hymenoptera	*Bombus* (2)	7-8	1	1	3	4	3	1	8-10	29-30
Hymenoptera	*Camponotus* (1)	8	2	1	2	3	2	1	8	27
Hymenoptera	*Cardiocondyla* (1)	7	2	1	3	4	3	1	10	31
Hymenoptera	*Cephus* (1)	6	1	1	2	3	2	1	6	22
Hymenoptera	*Cerapachys* (1)	5	1	1	2	3	3	1	6	22
Hymenoptera	*Ceratosolen* (1)	8	1	1	3	3	2	1	9	28
Hymenoptera	*Copidosoma* (1)	17	1	1	3	4	2	1	16	45
Hymenoptera	*Dufourea* (1)	7	2	1	3	4	3	1	7	28

Hymenoptera	*Eufriesea* (1)	6	2	1	3	4	3	1	8	28
Hymenoptera	*Fopius* (1)	9	1	1	3	4	1	1	9	29
Hymenoptera	*Habropoda* (1)	8	2	1	3	4	3	1	8	30
Hymenoptera	*Harpegnathos* (1)	8	2	0	1	2	1	1	8	23
Hymenoptera	*Linepithema* (1)	7	2	1	3	4	3	1	8	29
Hymenoptera	*Megachile* (1)	7	2	1	3	3	3	1	8	28
Hymenoptera	*Melipona* (1)	7	2	1	3	4	3	1	8	29
Hymenoptera	*Microplitis* (1)	18	1	1	3	4	3	2	8	40
Hymenoptera	*Monomorium* (1)	6	1	1	2	3	2	1	5	21
Hymenoptera	*Nasonia* (1)	17	1	1	3	4	2	1	23	52
Hymenoptera	*Orussus* (1)	11	2	1	2	3	3	1	7	30
Hymenoptera	*Pogonomyrmex* (1)	5	2	1	2	4	3	1	8	26
Hymenoptera	*Polistes* (1)	6	1	1	1	4	2	1	6	22
Hymenoptera	*Solenopsis* (1)	2	1	1	3	3	3	1	7	21
Hymenoptera	*Trichogramma* (1)	15	1	1	3	4	1	1	26	52
Hymenoptera	*Vollenhovia* (1)	6	1	1	3	4	2	1	3	21
Hymenoptera	*Hypothenemus* (1)	9	1	1	3	3	3	1	8	29
Hymenoptera	*Wasmannia* (1)	7	1	1	3	3	3	1	6	25
Isoptera	*Zootermopsis* (2)	6	1	2	2	4	3	1	10	29
Lepidoptera	*Bombyx* (1)	4	2	1	3	4	3	1	8	26
Lepidoptera	*Danaus* (1)	5	1	1	3	5	3	1	10	29
Lepidoptera	*Heliconius* (1)	5	1	1	2	4	3	1	6	23
Lepidoptera	*Papilio* (2)	6	1	1	3	2-4	2	1	9-11	26-27
Lepidoptera	*Lerema* (1)	4	1	2	3	3	3	1	10	27
Lepidoptera	*Melitaea* (1)	5	1	1	3	1	3	1	8	23
Lepidoptera	*Manduca* (1)	6	2	7	7	5	5	2	29	63
Lepidoptera	*Plutella* (1)	5	4	1	4	5	6	0	13	38
Odonata	*Ladona* (1)	3	2	2	3	4	3	1	9	27
Orthoptera	*Locusta* (1)	9	1	1	3	4	3	1	7	29
Phasmatoptera	*Timema* (1)	3	1	1	3	5	3	1	6	23
Thysanoptera	*Frankliniella* (1)	6	2	8	3	5	3	1	21	49
Trichoptera	*Limnephilus* (1)	3	1	0	2	3	2	1	6	18

Note: The numbers in parenthesis indicates the number of the species in each genus. The dash is used to represent the range of *SET* gene number in each genus. AS, arthropod-specific.

Click here to download Table Table2.16100701.xls

Table 2. Tests of rate heterogeneity acting on *SET* genes in insects.

	Gene	One Ratio Likelihood	One Ratio ω	Free Ratio Likelihood	df	*P*
SMYD	*Smyd3*	-4833.870633	0.055	-4833.870633	16	<0.001
	Smyd4-1	-17270.85481	0.1627	-17140.2931	58	<0.001
	Smyd4-2	-13187.36796	0.1125	-13112.10598	44	<0.001
	Smyd4-3	-20488.96316	0.1069	-20364.99139	66	<0.001
	Smyd4-4	-15552.36608	0.1112	-15475.97917	44	<0.001
	Smyd5	-21495.43548	0.0633	-21329.01303	64	<0.001
	upSET(MLL5)	-7286.598116	0.0103	-7247.800191	62	0.087
	Set8	-6450.096636	0.0321	-6386.997507	60	<0.001
	Hmt4-20	-3523.660744	0.0079	-3478.339497	56	<0.001
SETD	*SETD*	-9030.115692	0.033	-9009.972504	34	0.212
PRDM	*Blimp-1*	-2679.981724	0.0051	-2664.129882	52	0.988
Ash	*Mes-4*	-5530.425067	0.0163	-5504.225668	56	0.612
	ash1	-4995.315864	0.0122	-4947.987993	60	<0.001
	Set2	-5636.021533	0.0118	-5570.266003	60	<0.001
Suv	*Su(var)3-9*	-4351.473377	0.0212	-4308.872564	32	<0.001
	egg	-15308.27271	0.0624	-15214.54477	54	<0.001
	CG4565	-7168.675146	0.056	-7114.254055	46	<0.001
	G9a	-4641.585219	0.0091	-4604.810574	54	0.040
Trx	*trx*	-3897.22035	0.0031	-3877.624919	58	0.972
	Set1	-3733.003015	0.0026	-3700.07484	60	0.281
	trr	-4549.712	0.0114	-4471.116449	60	<0.001
E(z)	*Ez*	-3368.302419	0.0007	-3355.922925	61	1.000
SMYDA	*SmydA-1*	-10066.85883	0.0904	-9995.276076	34	<0.001
	SmydA-2	-11858.79656	0.0052	-11812.61641	30	<0.001
	SmydA-3	-13902.68842	0.0817	-13842.81154	56	<0.001
	SmydA-4	-9602.742487	0.0254	-9583.599425	26	0.057
	SmydA-5	-13748.76916	0.1179	-13656.26849	50	<0.001
	SmydA-6	-12142.19779	0.1623	-12043.99319	42	<0.001
	SmydA-9	-13258.40628	0.1357	-13193.53611	52	<0.001

Note: Accounting for the unequal genome sequencing efforts between different insect families, we selected one species within each genus to be representative of the genus.

Click here to download Table Table3.16082501.xls

Table 3. Signatures of selection acting on differential expressed *SET* genes in response to phenotypic plasticity.

Model-Parameters	APIME		LOCMI	ACREC		
	SmydA-3	*SmydA-5*	*SmydA-1*	*SmydA-3*	*SmydA-5*	*SmydA-9*
**Basic models**						
M0: ω	0.082	0.118	0.090	0.082	0.118	0.136
**Branch models**						
B0: lnL	-13914.741	-13749.007	-10088.904	-13905.140	-13749.047	-13259.370
B0: ω_0 ($\omega_1 = 1$)	0.077	0.113	0.090	0.081	0.117	0.135
BA: lnL	-13901.138	-13745.405	-10056.182	-13901.922	-13748.719	-13258.338
BA: ω_0, ω_1	0.080, 0.142	0.115, 0.313	0.095, 0.003	0.081, 0.177	0.118, 0.181	0.135, 0.186
**Branch-site models**						
A0: p_{2a} ($\omega_2 = 1$)	0.078	0.059	0.111	0.082	0.155	0.096
AA: p_{2a}', ω_2	0.078, 1.000	0.025, 3.102	0.109, 8.895	0.082, 1.000	0.155,1.000	0.011, 19.742
Positively selected sites (BEB)			5 M 11 K 93 P 105 C			
**LRT, *P***						
M0 versus BA	0.078	0.009	<0.001	0.216	0.752	0.712
BA versus B0	<0.001	0.007	<0.001	0.011	0.418	0.151
A0 versus AA	1.000	0.802	0.022	1.000	1.000	0.082

ω, the ratios of nonsynonymous substitution per nonsynonymous site to synonymous substitution per synonymous site; ω0, ω1, background and foreground ω values, respectively; APIME, *Apis mellifera* ; ACREC, *Acromyrmex echinator* ; LOCMI, *Locusta migratoria.*

Figure 1

Click here to download Figure figure1_20181006.pdf

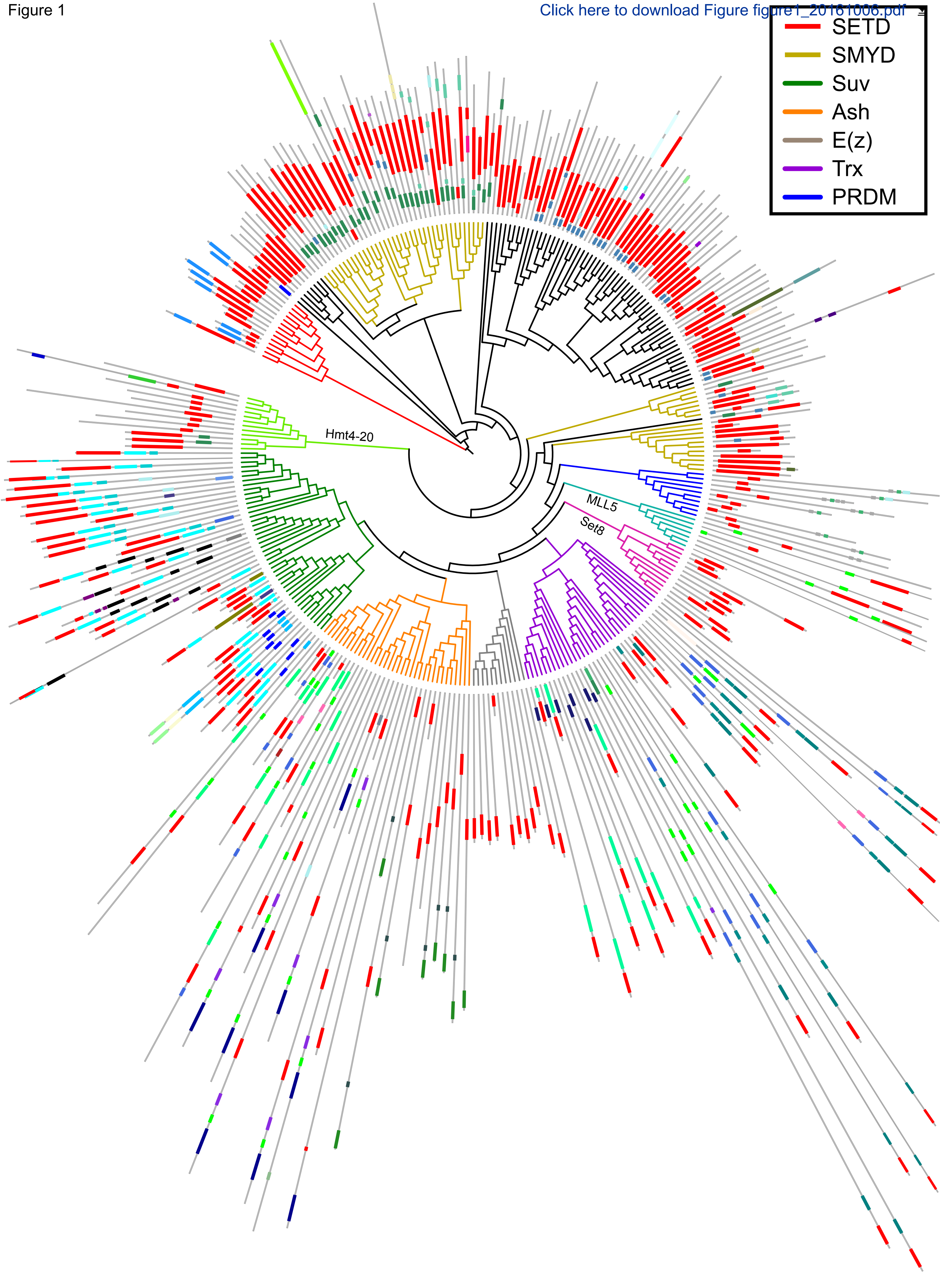

Click here to download Figure figure2_2016100501.pdf

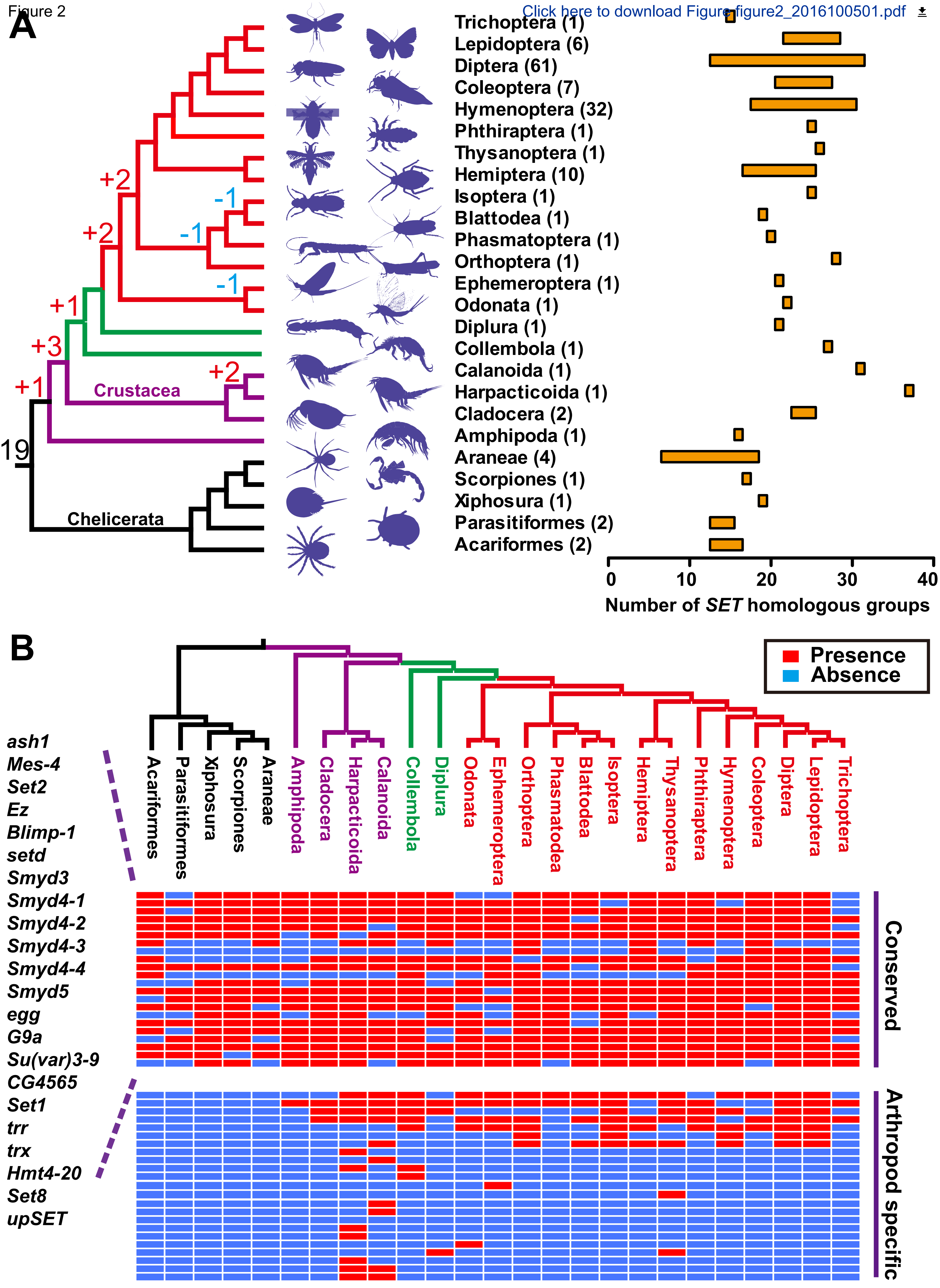

Figure 3

Click here to download Figure figure3_2016102801.pdf

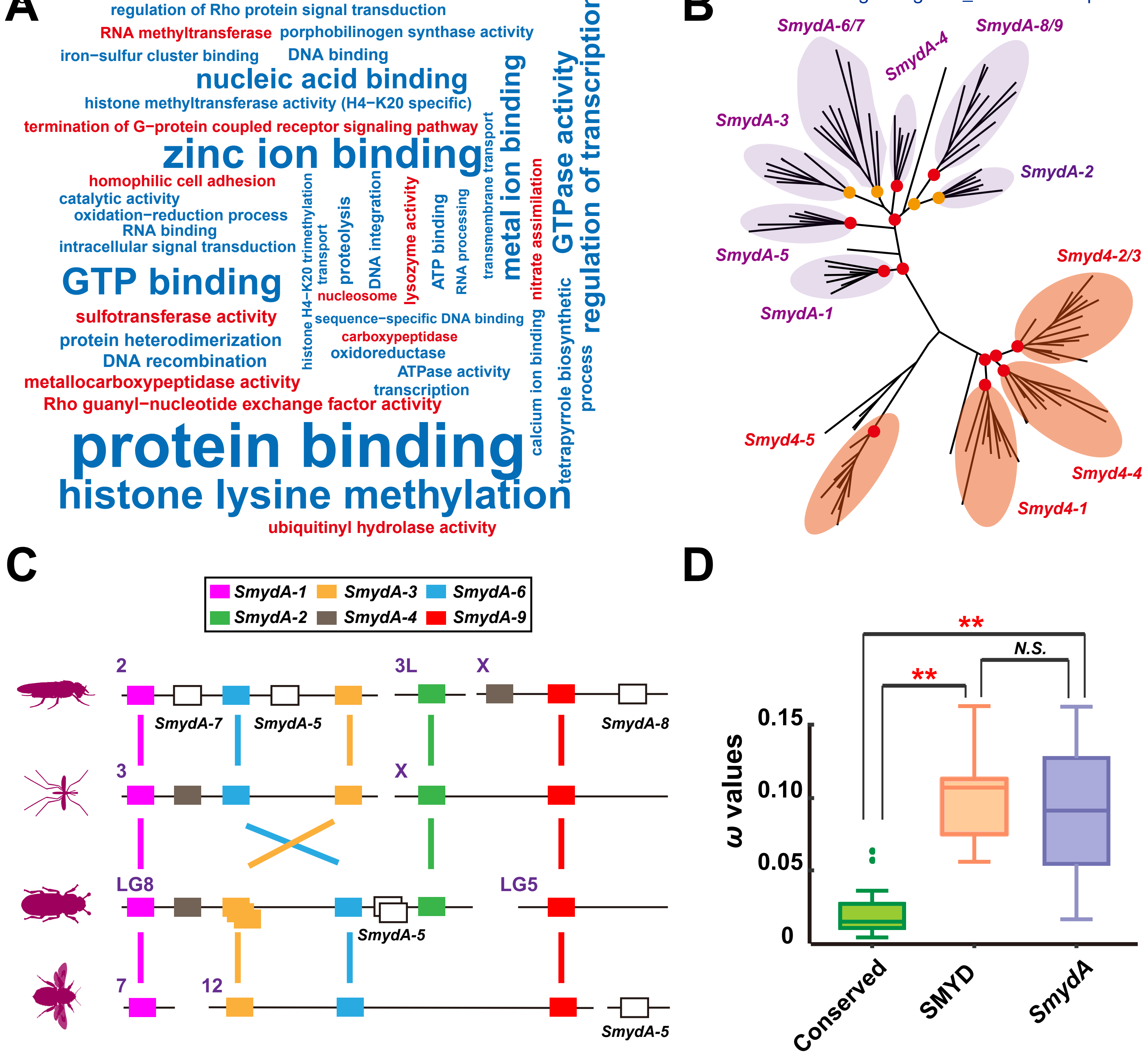

Figure 4 Click here to download Figure figure4_2016102801.pdf

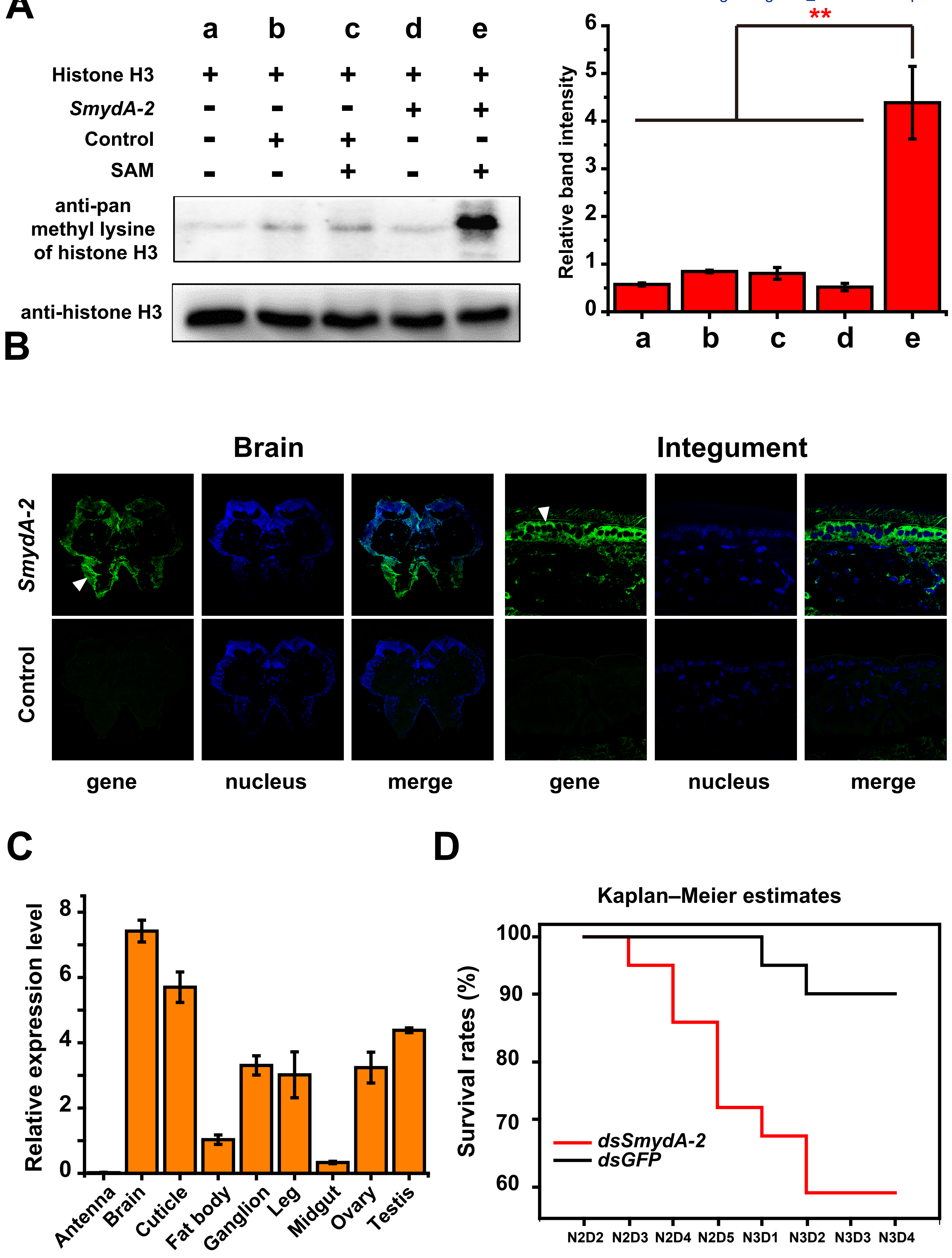

Figure 5

Click here to download Figure figure5_2016101801.pdf

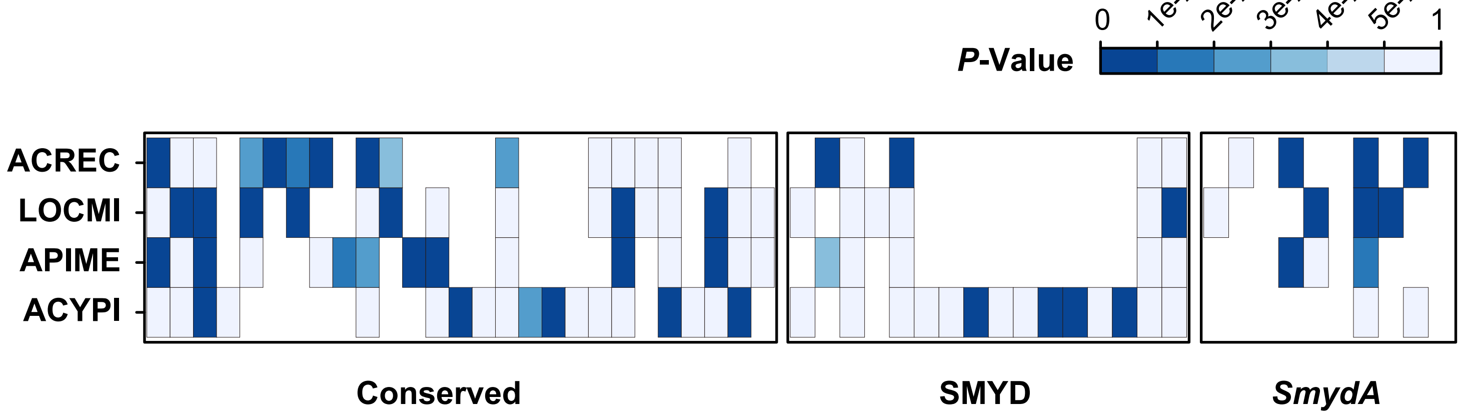

Click here to access/download
Supplementary Material
insectsSetDomain_supply_16102701_giga.pdf
