# Supplementary material for: Comparative genomic analysis of SET domain family reveals the origin, expansion, and putative function of the arthropod-specific SmydA genes as histone modifiers in insects
Source: Gigascience. 2017 Apr 22;6(6):1–16. doi: 10.1093/gigascience/gix031 (PMC5459927; doi:10.1093/gigascience/gix031)
Supplement: Supplemental material — Additional file 1: Table S1: The arthropod genome data involved in this study. Additional file 2: Table S2: SET genes in the 147 arthropod genomes. Additional file 3: Table S3: Summary of SET genes in the 147 arthropod genomes. Additional file 4: Table S4: Primers used in the study. Additional file 5: Figure S1: Phylogenetic analysis of the SET genes in Lepidoptera using maximum-likelihood inferences with PhyML. The SET gene families labeled with different colors are shown in the exterior circle of the phylogenetic tree. The insect species involved are represented with different colors of the external branch. Additional file 6: Figure S2: Phylogenetic analysis of the SET genes in Diptera using maximum-likelihood inferences with PhyML. The SET gene families labeled with different colors are shown in the exterior circle of the phylogenetic tree. The insect species involved are represented with different colors of the external branch. The representative species are selected to improve clarity. Additional file 7: Figure S3: Phylogenetic analysis of the SET genes in Hemiptera using maximum-likelihood inferences with PhyML. The SET gene families labeled with different colors are shown in the exterior circle of the phylogenetic tree. The insect species involved are represented with different colors of the external branch. Additional file 8: Figure S4: Phylogenetic analysis of the SET genes in Hymenoptera using maximum-likelihood inferences with PhyML. The SET gene families labeled with different colors are shown in the exterior circle of the phylogenetic tree. The insect species involved are represented with different colors of the external branch. The representative species are selected to improve clarity. Additional file 9: Figure S5: Phylogenetic analysis of the SET genes in Coleopteran using maximum-likelihood inferences with PhyML. The SET gene families labeled with different colors are shown in the exterior circle of the phylogenetic tree. The insect species involved [file gix031_Supp.pdf]

Supplementary materials

Comparative genomic analysis of *SET*-domain family reveals the origin, expansion, and putative function of the arthropod-specific *SmydA* genes as histone modifier in insects

Feng Jiang[1, *], Qing Liu[1, 2, *], Yanli Wang[2, 3], Jie Zhang[1], Huimin Wang[1], Tianqi Song[3], Meiling Yang[2], Xianhui Wang[2, #], Le Kang[1, 2, #]

[1] Beijing Institutes of Life Science, Chinese Academy of Sciences, Beijing, China

[2] Institute of Zoology, Chinese Academy of Sciences, Beijing, China

[3] Institute of Applied Biology, Shanxi University, Taiyuan, Shanxi, China

*These authors contributed equally to this study.

Running head: Evolution of *SET* Genes in Insects

Supplementary Tables

Supplementary Table S1. The arthropod genome data involved in this study.

Species	Abbreviation	Database	Version
Pediculus humanus	PEDHU	ftp.vectorbase.org/public_data/organism_data/phumanus/Geneset/phumanus.PEPTIDES-PhumU1.2.fa.gz	v1.2
Blattella germanica	BLAGE	ftp://ftp.hgsc.bcm.edu/I5K-pilot/German_cockroach/maker_annotation/version_0.5.3/BGER.faa	v0.5.3
Agrilus planipennis	AGRPL	ftp://ftp.hgsc.bcm.tmc.edu/I5K-pilot/Emerald_ash_borer/maker_annotation/version_0.5.3/APLA.faa	v0.5.3
Anoplophora glabripennis	ANOGL	ftp://ftp.hgsc.bcm.edu/I5K-pilot/Asian_long-horned_beetle/maker_annotation/version_0.5.3/AGLA.faa	v0.5.3
Dendroctonus ponderosae	DENPO	ftp://ftp.ensemblgenomes.org/pub/metazoa/release-32/fasta/dendroctonus_ponderosae/pep/Dendroctonus_ponderosae.GCA_000355655.1.pep.all.fa.gz	ensembl-32
Leptinotarsa decemlineata	LEPDE	ftp://ftp.hgsc.bcm.edu/I5K-pilot/Colorado_Potato_Beetle/maker_annotation/version_0.5.3/LDEC.faa	v0.5.3
Onthophagus taurus	ONTTA	ftp://ftp.hgsc.bcm.edu/I5K-pilot/Bull-headed_Dung_beetle/maker_annotation/version_0.5.3/OTAU.faa	v0.5.3
Oryctes borbonicus	ORYBO	ftp://ftp.ncbi.nlm.nih.gov/genomes/all/GCA_001443705.1_ASM144370v1/GCA_001443705.1_ASM144370v1_protein.faa.gz	v1
Tribolium castaneum	TRICA	http://nosil-lab.group.shef.ac.uk/wp-content/uploads/data/timema_cristinae_draft_2014_gff_prots.tar.bz2	v3.0
Aedes aegypti	AEDAE	ftp.vectorbase.org/public_data/organism_data/aaegypti/Geneset/aaegypti.PEPTIDES-AaegL1.2.fa.gz	v1.2
Aedes albopictus	AEDAL	https://www.vectorbase.org/download/aedes-albopictus-foshanpeptidesaalof11fagz	v1.1

Anopheles albimanus	ANOAL	https://www.vectorbase.org/download/anopheles-albimanus-steclapeptidesaalbs13fagz	v1.3
Anopheles arabiensis	ANOAR	https://www.vectorbase.org/download/anopheles-arabiensis-dongolapeptidesaarad13fagz	v1.3
Anopheles atroparvus	ANOAT	https://www.vectorbase.org/download/anopheles-atroparvus-ebropeptidesaatre13fagz	v1.3
Anopheles christyi	ANOCH	https://www.vectorbase.org/download/anopheles-christyi-achkn1017peptidesachra13fagz	v1.3
Anopheles coluzzii	ANOCO	https://www.vectorbase.org/download/anopheles-coluzzii-mali-nihpeptidesacolm13fagz	v1.3
Anopheles culicifacies	ANOCU	https://www.vectorbase.org/download/anopheles-culicifacies-37peptidesacula13fagz	v1.3
Anopheles darlingi	ANODA	ftp://ftp.ensemblgenomes.org/pub/metazoa/release-32/fasta/anopheles_darlingi/pep/Anopheles_darlingi.AdarC3.pep.all.fa.gz	ensembl-32
Anopheles dirus	ANODI	https://www.vectorbase.org/download/anopheles-dirus-wrair2peptidesadirw13fagz	v1.3
Anopheles epiroticus	ANOEP	https://www.vectorbase.org/download/anopheles-epiroticus-epiroticus2peptidesaepie13fagz	v1.3
Anopheles farauti	ANOFA	https://www.vectorbase.org/download/anopheles-farauti-far1peptidesafarf21fagz	v1.3
Anopheles funestus	ANOFU	https://www.vectorbase.org/download/anopheles-funestus-fumozpeptidesafunf13fagz	v1.3
Anopheles gambiae	ANOGA	ftp://ftp.vectorbase.org/public_data/organism_data/agambiae/Geneset/agambiae.PEPTIDES-AgamP3.6.fa.gz	v3.6

Anopheles maculatus	ANOMA	https://www.vectorbase.org/download/anopheles-maculatus-maculatus3peptidesamacm13fagz	v1.3
Anopheles melas	ANOME	https://www.vectorbase.org/download/anopheles-melas-cm1001059apeptidesamelc21fagz	v2.1
Anopheles merus	ANOMR	https://www.vectorbase.org/download/anopheles-merus-mafpeptidesamerm21fagz	v2.1
Anopheles minimus	ANOMI	https://www.vectorbase.org/download/anopheles-minimus-minimus1peptidesaminm13fagz	v1.3
Anopheles quadriannulatus	ANOQU	https://www.vectorbase.org/download/anopheles-quadriannulatus-sangwepeptidesaquas13fagz	v1.3
Anopheles sinensis	ANOSI	https://www.vectorbase.org/download/anopheles-sinensis-sinensispeptidesasins21fagz	v2.1
Anopheles stephensi	ANOST	https://www.vectorbase.org/download/anopheles-stephensi-indianpeptidesastei22fagz	v2.2
Bactrocera cucurbitae	BACCU	ftp://ftp.ncbi.nlm.nih.gov/genomes/all/GCF_000806345.1_ASM80634v1/GCF_000806345.1_ASM80634v1_protein.faa.gz	v1
Bactrocera dorsalis	BACDO	http://www.insect-genome.com/data/genome_download/Bactrocera_dorsalis/Bactrocera_dorsalis_protein.fasta.gz	v2.7
Ceratina calcarata	CERCA	ftp://ftp.ncbi.nlm.nih.gov/genomes/all/GCF_001652005.1_ASM165200v1/GCF_001652005.1_ASM165200v1_protein.faa.gz	v1.1
Ceratitis capitata	CERCP	ftp://ftp.hgsc.bcm.edu/I5K-pilot/Mediterranean_fruit_fly/maker_annotation/version_0.5.3/CCAP.faa	v0.5.3
Culex quinquefasciatus	CULQU	ftp.vectorbase.org/public_data/organism_data/cquinquefasciatus/Geneset/cquinquefasciatus.BASEFEATURES_Johannesburg-CpipJ1.2.gff3.gz	v1.2

Drosophila ananassae	DROAN	ftp://ftp.ensemblgenomes.org/pub/metazoa/release-32/fasta/drosophila_ananassae/pep/Drosophila_ananassae.GCA_000005115.1.pep.all.fa.gz	ensembl-32
Drosophila biarmipes	DROBI	ftp://ftp.hgsc.bcm.edu/DmodENCODE/maker_annotation/Drosophila_biarmipes/version_0.5.3/DBIA.faa	v0.5.3
Drosophila bipectinata	DROBP	ftp://ftp.hgsc.bcm.edu/DmodENCODE/maker_annotation/Drosophila_bipectinata/version_0.5.3/DBIP.faa	v0.5.3
Drosophila elegans	DROEL	ftp://ftp.hgsc.bcm.edu/DmodENCODE/maker_annotation/Drosophila_elegans/version_0.5.3/DELE.faa	v0.5.3
Drosophila erecta	DROER	ftp://ftp.ensemblgenomes.org/pub/metazoa/release-32/fasta/drosophila_erecta/pep/Drosophila_erecta.GCA_000005135.1.pep.all.fa.gz	ensembl-32
Drosophila eugracilis	DROEU	ftp://ftp.hgsc.bcm.edu/DmodENCODE/maker_annotation/Drosophila_eugracilis/version_0.5.3/DEUG.faa	v0.5.3
Drosophila ficusphila	DROFI	ftp://ftp.hgsc.bcm.edu/DmodENCODE/maker_annotation/Drosophila_ficusphila/version_0.5.3/DFIC.faa	v0.5.3
Drosophila grimshawi	DROGR	ftp://ftp.ensemblgenomes.org/pub/metazoa/release-32/fasta/drosophila_grimshawi/pep/Drosophila_grimshawi.GCA_000005155.1.pep.all.fa.gz	ensembl-32
Drosophila kikkawai	DROKI	ftp://ftp.hgsc.bcm.edu/DmodENCODE/maker_annotation/Drosophila_kikkawai/version_0.5.3/DKIK.faa	v0.5.3
Drosophila melanogaster	DROME	ftp://ftp.flybase.net/genomes/Drosophila_melanogaster/dmel_r6.04_FB2015_01/fasta/dmel-all-translation-r6.04.fasta.gz	r6.04
Drosophila miranda	DROMI	ftp://ftp.ncbi.nih.gov/genomes/Drosophila_miranda/protein/Gnomon_prot.fsa.gz	v2.2
Drosophila mojavensis	DROMO	http://www.insect-genome.com/data/genome_download/Drosophila_mojavensis/Drosophila_mojavensis_protein.fasta.gz	v2

Drosophila persimilis	DROPE	ftp://ftp.ensemblgenomes.org/pub/metazoa/release-32/fasta/drosophila_persimilis/pep/Drosophila_persimilis.GCA_000005195.1.pep.all.fa.gz	ensembl-32
Drosophila pseudoobscura	DROPS	ftp.flybase.net/genomes/Drosophila_pseudoobscura/dpse_r2.10_FB2010_04/fasta/dpse-all-translation-r2.10.fasta.gz	r2.10
Drosophila rhopaloa	DRORH	ftp://ftp.hgsc.bcm.edu/DmodENCODE/maker_annotation/Drosophila_rhopaloa/version_0.5.3/DRHO.faa	v0.5.3
Drosophila sechellia	DROSE	ftp://ftp.ensemblgenomes.org/pub/metazoa/release-32/fasta/drosophila_sechellia/pep/Drosophila_sechellia.GCA_000005215.1.pep.all.fa.gz	ensembl-32
Drosophila simulans	DROSI	ftp://ftp.ensemblgenomes.org/pub/metazoa/release-32/fasta/drosophila_simulans/pep/Drosophila_simulans.GCA_000259055.1.pep.all.fa.gz	ensembl-32
Drosophila suzukii	DROSU	ftp://ftp.ncbi.nih.gov/genomes/Drosophila_suzukii/protein/Gnomon_prot.fsa.gz	v1
Drosophila takahashii	DROTA	ftp://ftp.hgsc.bcm.edu/DmodENCODE/maker_annotation/Drosophila_takahashii/version_0.5.3/DTAK.faa	v0.5.3
Drosophila virilis	DROVI	ftp://ftp.ensemblgenomes.org/pub/metazoa/release-32/fasta/drosophila_virilis/pep/Drosophila_virilis.GCA_000005245.1.pep.all.fa.gz	ensembl-32
Drosophila willistoni	DROWI	ftp://ftp.ensemblgenomes.org/pub/metazoa/release-32/fasta/drosophila_willistoni/pep/Drosophila_willistoni.GCA_000005925.1.pep.all.fa.gz	ensembl-32
Drosophila yakuba	DROYA	ftp://ftp.flybase.net/genomes/Drosophila_yakuba/dyak_r1.3_FB2010_02/fasta/dyak-all-translation-r1.3.fasta.gz	r5.27
Glossina austeni	GLOAU	https://www.vectorbase.org/download/glossina-austeni-ttripeptidesgaust13fagz	v1.3
Glossina brevipalpis	GLOBR	https://www.vectorbase.org/download/glossina-brevipalpis-iaeapeptidesgbrei13fagz	v1.3
Glossina fuscipes	GLOFU	https://www.vectorbase.org/download/glossina-fuscipes-iaeapeptidesgfusi13fagz	v1.3

Glossina morsitans	GLOMO	https://www.vectorbase.org/download/glossina-morsitans-yalepeptidesgmory15fagz	v1.5
Glossina pallidipes	GLOPA	https://www.vectorbase.org/download/glossina-pallidipes-iaeapeptidesgpali13fagz	v1.3
Glossina palpalis	GLOPL	https://www.vectorbase.org/download/glossina-palpalis-iaeapeptidesgpapi11fagz	v1.1
Lucilia cuprina	LUCCU	ftp://ftp.hgsc.bcm.edu/I5K-pilot/Sheep_blowfly/maker_annotation/version_0.5.3/LCUP.faa	v0.5.3
Lutzomyia longipalpis	LUTLO	https://www.vectorbase.org/download/lutzomyia-longipalpis-jacobinapeptidesllonj12fagz	v1.2
Mayetiola destructor	MAYDE	ftp://ftp.ncbi.nlm.nih.gov/genomes/all/GCA/000/149/185/GCA_000149185.1_Mdes_1.0/GCA_000149185.1_Mdes_1.0_genomic.fna.gz	v1.0
Megaselia scalaris	MEGSC	ftp://ftp.ensemblgenomes.org/pub/release-32/metazoa/fasta/megaselia_scalaris/pep/Megaselia_scalaris.Msca1.pep.all.fa.gz	v1
Musca domestica	MUSDO	https://www.vectorbase.org/download/musca-domestica-aabyspeptidesmdoma11fagz	v1.1
Phlebotomus papatasi	PHLPA	https://www.vectorbase.org/download/phlebotomus-papatasi-israelpeptidespapi12fagz	v1.2
Stomoxys calcitrans	STOCA	https://www.vectorbase.org/download/stomoxys-calcitrans-usdapeptidesscalu11fagz	v1.1
Ephemera danica	EPHDA	ftp://ftp.hgsc.bcm.edu/I5K-pilot/Mayfly/maker_annotation/version_0.5.3/EDAN.faa	v0.5.3
Acyrthosiphon pisum	ACYPI	http://bipaa.genouest.org/data/public/a_pisum/ACYPIprot.fa.bz2	v1.0
Cimex lectularius	CIMLE	ftp://ftp.hgsc.bcm.edu/I5K-pilot/Bed_bug/maker_annotation/version_0.5.3/CLEC.faa	v0.5.3

Diaphorina citri	DIACI	ftp://ftp.ncbi.nlm.nih.gov/genomes/all/GCF/000/475/195/GCF_000475195.1_Diaci_psyllid_genome_assembly_version_1.1/GCF_000475195.1_Diaci_psyllid_genome_assembly_version_1.1_protein.faa.gz	v1.1
Gerris buenoi	GERBU	ftp://ftp.hgsc.bcm.edu/I5K-pilot/Water_strider/maker_annotation/version_0.5.3/GBUE.faa	v0.5.3
Halyomorpha halys	HALHA	ftp://ftp.hgsc.bcm.edu/I5K-pilot/Brown_marmorated_stink_bug/maker_annotation/version_0.5.3/HHAL.faa	v0.5.3
Homalodisca vitripennis	HOMVI	ftp://ftp.hgsc.bcm.edu/I5K-pilot/Glassy-winged_sharpshooter/maker_annotation/version_0.5.3/HVIT.faa	v0.5.3
Nilaparvata lugens	NILLU	ftp://climb.genomics.cn/pub/10.5524/100001_101000/100139/Nlug_v1.1.fa1.Nchange.pep.gz	v1.1
Oncopeltus fasciatus	ONCFA	ftp://ftp.hgsc.bcm.edu/I5K-pilot/Milkweed_bug/maker_annotation/version_0.5.3/OFAS.faa	v0.5.3
Pachypsylla venusta	PACVE	ftp://ftp.hgsc.bcm.edu/I5K-pilot/Hackberry_petiole_gall_psyllid/maker_annotation/version_0.5.3/PVEN.faa	v0.5.3
Rhodnius prolixus	RHOPR	https://www.vectorbase.org/download/rhodnius-prolixus-cdcpeptidesrproc31fagz	v3.1
Acromyrmex echinatior	ACREC	http://www.antgenomes.org/downloads/proteins/Acromyrmex_echinatior/Aech_v3.8.pep.fa.gz	v3.8
Apis dorsata	APIDO	ftp://ftp.ncbi.nih.gov/genomes/Apis_dorsata/protein/Gnomon_prot.fsa.gz	v1.3
Apis florea	APIFL	ftp://ftp.ncbi.nlm.nih.gov/genomes/all/GCF/000/184/785/GCF_000184785.2_Aflo_1.0/GCF_000184785.2_Aflo_1.0_protein.faa.gz	v1.0
Apis mellifera	APIME	http://hymenopteragenome.org/drupal/sites/hymenopteragenome.org.beebase/files/data/Amel_pre_release2_OGS_pep.fa.gz	v2.0

Athalia rosae	ATHRO	ftp://ftp.ncbi.nlm.nih.gov/genomes/all/GCF/000/344/095/GCF_000344095.1_Aros_1.0/GCF_000344095.1_Aros_1.0_protein.faa.gz	v1.0
Atta cephalotes	ATTCE	http://www.antgenomes.org/downloads/annotations/Atta_cephalotes/GCA_000143395.2_Acep_genomic.gff.gz	v1.2
Bombus impatiens	BOMIM	http://hymenopteragenome.org/beebase/sites/hymenopteragenome.org.beebase/files/data/consortium_data/Bombus_impatiens_v1.2.pep.fa.gz	v1.2
Bombus terrestris	BOMTE	http://hymenopteragenome.org/beebase/sites/hymenopteragenome.org.beebase/files/data/consortium_data/Bombus_terrestris_v1.3.pep.fa.gz	v1.3
Camponotus floridanus	CAMFL	http://www.antgenomes.org/downloads/annotations/Camponotus_floridanus/GCA_000147175.1_Cflo_genomic.gff.gz	v3.3
Cardiocondyla obscurior	CAROB	http://hymenopteragenome.org/drupal/sites/hymenopteragenome.org.ant_genomes.cardiocondyla/files/data/Cobs_1.4_OGS_proteins.fa.gz	v1.4
Cephus cinctus	CEPCI	ftp://ftp.ncbi.nlm.nih.gov/genomes/all/GCF/000/341/935/GCF_000341935.1_Ccin1/GCF_000341935.1_Ccin1_protein.faa.gz	v1
Cerapachys biroi	CERBI	ftp://ftp.ncbi.nlm.nih.gov/genomes/all/GCF/000/611/835/GCF_000611835.1_CerBir1.0/GCF_000611835.1_CerBir1.0_protein.faa.gz	v1.0
Ceratosolen solmsi	CERSO	http://www.insect-genome.com/data/genome_download/Ceratosolen_solmsi/Ceratosolen_solmsi_protein.fasta.gz	v1.0
Copidosoma floridanum	COPFL	ftp://ftp.hgsc.bcm.edu/I5K-pilot/Copidosoma_floridanum/maker_annotation/version_0.5.3/CFLO.faa	v0.5.3
Dufourea novaeangliae	DUFNO	http://hymenopteragenome.org/beebase/sites/hymenopteragenome.org.beebase/files/data/consortium_data/Dufourea_novaeangliae_v1.1.pep.fa	v1.1
Eufriesea mexicana	EUFME	http://hymenopteragenome.org/beebase/sites/hymenopteragenome.org.beebase/files/data/consortium_data/Eufriesea_mexicana_v1.1.pep.fa.gz	v1.1

Fopius arisanus	FOPAR	ftp://ftp.ncbi.nlm.nih.gov/genomes/all/GCF/000/806/365/GCF_000806365.1_ASM80636v1/GCF_000806365.1_ASM80636v1_protein.faa.gz	v1.0
Habropoda laboriosa	HABLA	http://hymenopteragenome.org/beebase/sites/hymenopteragenome.org.beebase/files/data/consortium_data/Habropoda_laboriosa_v1.2.pep.fa.gz	v1.2
Harpegnathos saltator	HARSA	http://www.antgenomes.org/downloads/annotations/Harpegnathos_saltator/GCA_000147195.1_Hsal_genomic.gff.gz	v3.3
Linepithema humile	LINHU	http://www.antgenomes.org/downloads/annotations/Linepithema_humile/GCA_000217595.1_Lhum_genomic.gff.gz	v1.2
Megachile rotundata	MEGRO	http://hymenopteragenome.org/beebase/sites/hymenopteragenome.org.beebase/files/data/consortium_data/Megachile_rotundata_v1.1.pep.fa.gz	v1.1
Melipona quadrifasciata	MELQU	http://hymenopteragenome.org/beebase/sites/hymenopteragenome.org.beebase/files/data/consortium_data/Melipona_quadrifasciata_v1.1.pep.fa.gz	v1.1
Microplitis demolitor	MICDE	ftp://ftp.ncbi.nlm.nih.gov/genomes/all/GCF/000/572/035/GCF_000572035.2_Mdem2/GCF_000572035.2_Mdem2_protein.faa.gz	v2.0
Monomorium pharaonis	MONPH	ftp://ftp.ncbi.nlm.nih.gov/genomes/all/GCF/000/980/195/GCF_000980195.1_M.pharaonis_V2.0/GCF_000980195.1_M.pharaonis_V2.0_protein.faa.gz	v2.0
Nasonia vitripennis	NASVI	http://genomes.arc.georgetown.edu/nasonia/nasonia_genome_consortium/data/Nvit_OGSv1.2_pep.fa.gz	v1.2
Orussus abietinus	ORUAB	ftp://ftp.hgsc.bcm.edu/I5K-pilot/Parasitic_wood_wasp/maker_annotation/version_0.5.3/OABI.faa	v0.5.3
Pogonomyrmex barbatus	POGBA	http://www.antgenomes.org/downloads/annotations/Pogonomyrmex_barbatus/GCA_000187915.1_Pbar_genomic.gff.gz	v1.2
Polistes dominula	POLDO	ftp://ftp.ncbi.nlm.nih.gov/genomes/all/GCF_001465965.1_Pdom_r1.2/GCF_001465965.1_Pdom_r1.2_protein.faa.gz	v1.2

Solenopsis invicta	SOLIN	http://www.antgenomes.org/downloads/genome/Solenopsis_invicta/GCA_000188075.1_Sinv_genomic.fna.gz	v2.2.3
Trichogramma pretiosum	TRIPR	ftp://ftp.hgsc.bcm.edu/I5K-pilot/Trichogramma_pretiosum/maker_annotation/version_0.5.3/TPRE.faa	v0.5.3
Vollenhovia emeryi	VOLEM	ftp://ftp.ncbi.nlm.nih.gov/genomes/all/GCF/000/949/405/GCF_000949405.1_V.emery_V1.0/GCF_000949405.1_V.emery_V1.0_protein.faa.gz	v1.0
Wasmannia auropunctata	WASAU	ftp://ftp.ncbi.nlm.nih.gov/genomes/all/GCF/000/956/235/GCF_000956235.1_wasmannia.A_1.0/GCF_000956235.1_wasmannia.A_1.0_protein.faa.gz	v1.0
Zootermopsis nevadensis	ZOONE	http://termitegenome.org/sites/www.termitegenome.org/files/data/Znev/Znev.OGS.v2.2.pep.gz	v2.2
Bombyx mori	BOMMO	http://silkworm.genomics.org.cn/silkdb/genome/	v2.0
Danaus plexippus	DANPL	ftp.jgi-psf.org/pub/JGI_data/Daphnia_pulex/v1.0/Daphnia_pulex.fasta.gz	v1
Heliconius melpomene	HELME	http://butterflygenome.org/sites/default/files/HelEraLat10.tar	v1.1
Papilio polytes	PAPPO	http://papilio.bio.titech.ac.jp/genome-browser//download/pp/Ppolytes.v1.0.0.pep.faa.gz	v1.0.0
Papilio xuthus	PAPXU	http://papilio.bio.titech.ac.jp/genome-browser//download/px/Pxuthus.v1.0.0.pep.faa.gz	v1.0.0
Plutella xylostella	PLUXY	http://iae.fafu.edu.cn/DBM/data/DBM-DB.v1.0/Gene/P.xylostella.pep.fasta.tar.gz	v1.0
Ladona fulva	LADFU	ftp://ftp.hgsc.bcm.edu/I5K-pilot/Scarce_Chaser/maker_annotation/version_0.5.3/LFUL.faa	v0.5.3
Locusta migratoria	LOCMI	http://159.226.67.243/index.html	v2.4

Timema cristinae	TIMCR	http://nosil-lab.group.shef.ac.uk/wp-content/uploads/data/timema_cristinae_draft_2014_gff_prots.tar.bz2	v1.0
Frankliniella occidentalis	FRAOC	ftp://ftp.hgsc.bcm.edu/I5K-pilot/Western_flower_thrips/maker_annotation/version_0.5.3/FOCC.faa	v0.5.3
Limnephilus lunatus	LIMLU	ftp://ftp.hgsc.bcm.edu/I5K-pilot/Caddisfly/maker_annotation/version_0.5.3/LLUN.faa	v0.5.3
Manduca sexta	MANSE	ftp://ftp.bioinformatics.ksu.edu/pub/Manduca/OGS2/OGS2_20140407_proteins.fa	v1.0
belgica ntarctica	BELNT	ftp://ftp.ensemblgenomes.org/pub/release-32/metazoa/fasta/belgica_antarctica/pep/	release-32
Lasioglossum albipes	LASAL	http://hymenopteragenome.org/beebase/sites/hymenopteragenome.org.beebase/files/data/consortium_data/Lalb_OGS_v5.42.pep.fa.gz	v5.42
Lerema accius	LERAC	http://prodata.swmed.edu/LepDB/lac/annotation_V1.1.tar.gz	V1.1
Melitaea cinxia	MELCI	ftp://ftp.ensemblgenomes.org/pub/metazoa/release-32/fasta/melitaea_cinxia/pep/	release-32
Centruroides exilicauda	CENCE	ftp://ftp.hgsc.bcm.edu/I5K-pilot/Bark_scorpion/maker_annotation/version_0.5.3/CSCU.faa	v0.5.3
Ixodes scapularis	IXOSC	ftp://ftp.ncbi.nlm.nih.gov/genomes/all/GCF_000208615.1_JCVI_ISG_i3_1.0/GCF_000208615.1_JCVI_ISG_i3_1.0_protein.faa.gz	v1.0
Latrodectus hesperus	LATHE	ftp://ftp.hgsc.bcm.edu/I5K-pilot/Western_black_widow_spider/maker_annotation/version_0.5.3/LHES.faa	v0.5.3
Loxosceles reclusa	LOCRE	ftp://ftp.hgsc.bcm.edu/I5K-pilot/Brown_recluse_spider/maker_annotation/version_0.5.3/LREC.faa	v0.5.3
Metaseiulus occidentalis	METOC	ftp://ftp.ncbi.nlm.nih.gov/genomes/all/GCF_000255335.1_Mocc_1.0/GCF_000255335.1_Mocc_1.0_protein.faa.gz	v1.0

Parasteatoda tepidariorum	PARTE	ftp://ftp.hgsc.bcm.edu/I5K-pilot/Common_house_spider/maker_annotation/version_0.5.3/PTEP.faa	v0.5.3
Sarcoptes scabiei	SARSC	ftp://ftp.ncbi.nlm.nih.gov/genomes/all/GCA_000828355.1_SarSca1.0/GCA_000828355.1_SarSca1.0_protein.faa.gz	v1.0
Stegodyphus mimosarum	STEMI	ftp://ftp.ncbi.nlm.nih.gov/genomes/all/GCA_000611955.2_Stegodyphus_mimosarum_v1/GCA_000611955.2_Stegodyphus_mimosarum_v1_protein.faa.gz	v1.0
Tetranychus urticae	TETUR	ftp://ftp.ncbi.nlm.nih.gov/genomes/all/GCF_000239435.1_ASM23943v1/GCF_000239435.1_ASM23943v1_protein.faa.gz	v1.0
Daphnia magna	DAPMA	ftp://ftp.ncbi.nlm.nih.gov/genomes/all/GCA_001632505.1_daphmag2.4/GCA_001632505.1_daphmag2.4_protein.faa.gz	v2.4
Daphnia pulex	DAPPU	ftp://ftp.ncbi.nlm.nih.gov/genomes/all/GCA_001632505.1_daphmag2.4/GCA_001632505.1_daphmag2.4_protein.faa.gz	v1.0
Orchesella cincta	ORCCI	ftp://ftp.ncbi.nlm.nih.gov/genomes/all/GCA_001718145.1_ASM171814v1/GCA_001718145.1_ASM171814v1_protein.faa.gz	v1.0
Catajapyx aquilonaris	CATAQ	ftp://ftp.hgsc.bcm.edu/I5K-pilot/Silvestris_Northern_Forcepstail/maker_annotation/version_0.5.3/CAQU.faa	v0.5.3
Hyalella azteca	HYAAZ	ftp://ftp.hgsc.bcm.edu/I5K-pilot/Hyalella_azteca/maker_annotation/version_0.5.3/HAZT.faa	v0.5.3
Eurytemora affinis	EURAF	ftp://ftp.hgsc.bcm.edu/I5K-pilot/Eurytemora_affinis/maker_annotation/version_0.5.3/EAFF.faa	v0.5.3
Tigriopus californicus	TIGCA	https://i5k.nal.usda.gov/sites/default/files/data/Arthropoda/tigcal-%28Tigriopus_californicus%29/Current%20Genome%20Assembly/2.Official%20or%20Primary%20Gene%20Set/TCALIF_v1.0/TCALIF_proteins_v1.0.fasta	v1.0
Limulus polyphemus	LIMPO	ftp://ftp.ncbi.nlm.nih.gov/genomes/all/GCF_000517525.1_Limulus_polyphemus-2.1.2/GCF_000517525.1_Limulus_polyphemus-2.1.2_protein.faa.gz	v2.1.2

Supplementary Table S2. *SET* genes in the 147 arthropod genomes.

Accession	Gene	Group	Species
Aech_10783	*ash1*	Ash	*Acromyrmex echinatior*
Aech_04466	*Blimp-1*	PRDM	*Acromyrmex echinatior*
Aech_15148	*egg*	Suv	*Acromyrmex echinatior*
Aech_00633	*Ez*	Ez	*Acromyrmex echinatior*
Aech_09941	*G9a*	Suv	*Acromyrmex echinatior*
Aech_03970	*Hmt4-20*	-	*Acromyrmex echinatior*
Aech_09491	*Mes-4*	Ash	*Acromyrmex echinatior*
Aech_01065	*Set1*	Trx	*Acromyrmex echinatior*
Aech_11841	*Set2*	Ash	*Acromyrmex echinatior*
Aech_17307	*Set8*	-	*Acromyrmex echinatior*
Aech_05094	*setd*	SETD	*Acromyrmex echinatior*
Aech_05096	*setd*	SETD	*Acromyrmex echinatior*
Aech_06259	*Smyd4-2*	SMYD	*Acromyrmex echinatior*
Aech_04646	*Smyd4-2*	SMYD	*Acromyrmex echinatior*
Aech_03712	*Smyd4-3*	SMYD	*Acromyrmex echinatior*
Aech_06416	*Smyd4-3*	SMYD	*Acromyrmex echinatior*
Aech_10190	*Smyd4-4*	SMYD	*Acromyrmex echinatior*
Aech_07361	*Smyd4-4*	SMYD	*Acromyrmex echinatior*
Aech_08327	*Smyd5*	SMYD	*Acromyrmex echinatior*
Aech_01678	*SmydA-3*	SmydA	*Acromyrmex echinatior*
Aech_01677	*SmydA-4*	SmydA	*Acromyrmex echinatior*
Aech_14789	*SmydA-5*	SmydA	*Acromyrmex echinatior*
Aech_02287	*SmydA-8*	SmydA	*Acromyrmex echinatior*
Aech_15245	*suv*	Suv	*Acromyrmex echinatior*
Aech_05334	*trr*	Trx	*Acromyrmex echinatior*
Aech_04399	*trx*	Trx	*Acromyrmex echinatior*
Aech_02461	*upSET(MLL5)*	-	*Acromyrmex echinatior*
ACYPI20513	-	-	*Acyrthosiphon pisum*
ACYPI23756	-	-	*Acyrthosiphon pisum*
ACYPI23949	-	-	*Acyrthosiphon pisum*
ACYPI25437	-	-	*Acyrthosiphon pisum*
ACYPI26657	-	-	*Acyrthosiphon pisum*
ACYPI27009	-	-	*Acyrthosiphon pisum*
ACYPI27763	-	-	*Acyrthosiphon pisum*
ACYPI28709	-	-	*Acyrthosiphon pisum*
ACYPI29273	-	-	*Acyrthosiphon pisum*
ACYPI29294	-	-	*Acyrthosiphon pisum*
ACYPI31177	-	-	*Acyrthosiphon pisum*
ACYPI36619	-	-	*Acyrthosiphon pisum*
ACYPI38782	-	-	*Acyrthosiphon pisum*
ACYPI40728	-	-	*Acyrthosiphon pisum*
ACYPI41724	-	-	*Acyrthosiphon pisum*

ACYPI41861	-	-	*Acyrthosiphon pisum*
ACYPI45428	-	-	*Acyrthosiphon pisum*
ACYPI45529	-	-	*Acyrthosiphon pisum*
ACYPI45729	-	-	*Acyrthosiphon pisum*
ACYPI45935	-	-	*Acyrthosiphon pisum*
ACYPI45936	-	-	*Acyrthosiphon pisum*
ACYPI55972	-	-	*Acyrthosiphon pisum*
ACYPI006399	*ash1*	Ash	*Acyrthosiphon pisum*
ACYPI006306	*egg*	Suv	*Acyrthosiphon pisum*
ACYPI003603	*egg*	Suv	*Acyrthosiphon pisum*
ACYPI003919	*egg*	Suv	*Acyrthosiphon pisum*
ACYPI006182	*egg*	Suv	*Acyrthosiphon pisum*
ACYPI22783	*egg*	Suv	*Acyrthosiphon pisum*
ACYPI009110	*Ez*	Ez	*Acyrthosiphon pisum*
ACYPI010108	*G9a*	Suv	*Acyrthosiphon pisum*
ACYPI000868	*Hmt4-20*	-	*Acyrthosiphon pisum*
ACYPI008948	*Hmt4-20*	-	*Acyrthosiphon pisum*
ACYPI27385	*Hmt4-20*	-	*Acyrthosiphon pisum*
ACYPI28025	*Hmt4-20*	-	*Acyrthosiphon pisum*
ACYPI49460	*Hmt4-20*	-	*Acyrthosiphon pisum*
ACYPI000557	*Set1*	Trx	*Acyrthosiphon pisum*
ACYPI26224	*Set1*	Trx	*Acyrthosiphon pisum*
ACYPI008644	*Set2*	Ash	*Acyrthosiphon pisum*
ACYPI002722	*Set8*	-	*Acyrthosiphon pisum*
ACYPI004578	*Smyd3*	SMYD	*Acyrthosiphon pisum*
ACYPI000531	*Smyd4-3*	SMYD	*Acyrthosiphon pisum*
ACYPI001574	*Smyd4-3*	SMYD	*Acyrthosiphon pisum*
ACYPI004606	*Smyd4-3*	SMYD	*Acyrthosiphon pisum*
ACYPI23878	*Smyd4-3*	SMYD	*Acyrthosiphon pisum*
ACYPI25513	*Smyd4-3*	SMYD	*Acyrthosiphon pisum*
ACYPI26757	*Smyd4-3*	SMYD	*Acyrthosiphon pisum*
ACYPI27467	*Smyd4-3*	SMYD	*Acyrthosiphon pisum*
ACYPI33312	*Smyd4-3*	SMYD	*Acyrthosiphon pisum*
ACYPI48770	*Smyd4-3*	SMYD	*Acyrthosiphon pisum*
ACYPI52471	*Smyd4-3*	SMYD	*Acyrthosiphon pisum*
ACYPI52915	*Smyd4-3*	SMYD	*Acyrthosiphon pisum*
ACYPI55839	*Smyd4-3*	SMYD	*Acyrthosiphon pisum*
ACYPI001927	*Smyd5*	SMYD	*Acyrthosiphon pisum*
ACYPI001902	*SmydA-5*	SmydA	*Acyrthosiphon pisum*
ACYPI44442	*SmydA-8*	SmydA	*Acyrthosiphon pisum*
ACYPI002814	*Su(var)3-9*	Suv	*Acyrthosiphon pisum*
ACYPI003098	*Su(var)3-9*	Suv	*Acyrthosiphon pisum*
ACYPI006256	*Su(var)3-9*	Suv	*Acyrthosiphon pisum*
ACYPI56420	*suv*	Suv	*Acyrthosiphon pisum*

ACYPI001043	*trr*	Trx	*Acyrthosiphon pisum*
ACYPI007793	*trx*	Trx	*Acyrthosiphon pisum*
ACYPI002785	*upSET(MLL5)*	-	*Acyrthosiphon pisum*
AAEL009666	*ash1*	Ash	*Aedes aegypti*
AAEL004255	*Blimp-1*	PRDM	*Aedes aegypti*
AAEL013770	*Blimp-1*	PRDM	*Aedes aegypti*
AAEL004290	*egg*	Suv	*Aedes aegypti*
AAEL013213	*Ez*	Ez	*Aedes aegypti*
AAEL012995	*Ez*	Ez	*Aedes aegypti*
AAEL008027	*Hmt4-20*	-	*Aedes aegypti*
AAEL010414	*Mes-4*	Ash	*Aedes aegypti*
AAEL010807	*Set1*	Trx	*Aedes aegypti*
AAEL006013	*Set2*	Ash	*Aedes aegypti*
AAEL000193	*Set8*	-	*Aedes aegypti*
AAEL007348	*Smyd4-1*	SMYD	*Aedes aegypti*
AAEL007346	*Smyd4-1*	SMYD	*Aedes aegypti*
AAEL001050	*Smyd4-2*	SMYD	*Aedes aegypti*
AAEL003516	*Smyd4-3*	SMYD	*Aedes aegypti*
AAEL003527	*Smyd4-3*	SMYD	*Aedes aegypti*
AAEL003992	*Smyd4-4*	SMYD	*Aedes aegypti*
AAEL003447	*Smyd4-4*	SMYD	*Aedes aegypti*
AAEL009454	*Smyd4-4*	SMYD	*Aedes aegypti*
AAEL011836	*Smyd4-4*	SMYD	*Aedes aegypti*
AAEL011838	*Smyd4-4*	SMYD	*Aedes aegypti*
AAEL000436	*Smyd5*	SMYD	*Aedes aegypti*
AAEL001936	*SmydA-1*	SmydA	*Aedes aegypti*
AAEL003788	*SmydA-2*	SmydA	*Aedes aegypti*
AAEL001857	*SmydA-3*	SmydA	*Aedes aegypti*
AAEL001920	*SmydA-4*	SmydA	*Aedes aegypti*
AAEL003295	*SmydA-5*	SmydA	*Aedes aegypti*
AAEL009751	*SmydA-6*	SmydA	*Aedes aegypti*
AAEL009757	*SmydA-6*	SmydA	*Aedes aegypti*
AAEL008032	*SmydA-8*	SmydA	*Aedes aegypti*
AAEL010826	*Su(var)3-9*	Suv	*Aedes aegypti*
AAEL005380	*trr*	Trx	*Aedes aegypti*
AAEL010578	*trx*	Trx	*Aedes aegypti*
AAEL001147	*upSET(MLL5)*	-	*Aedes aegypti*
AALF019942	-	-	*Aedes albopictus*
AALF028009	*ash1*	Ash	*Aedes albopictus*
AALF005153	*ash1*	Ash	*Aedes albopictus*
AALF013866	*Blimp-1*	PRDM	*Aedes albopictus*
AALF019168	*Blimp-1*	PRDM	*Aedes albopictus*
AALF001369	*egg*	Suv	*Aedes albopictus*
AALF015398	*egg*	Suv	*Aedes albopictus*

AALF024803	*Ez*	Ez	*Aedes albopictus*
AALF014060	*Hmt4-20*	-	*Aedes albopictus*
AALF017377	*Mes-4*	Ash	*Aedes albopictus*
AALF005355	*Set1*	Trx	*Aedes albopictus*
AALF012083	*Set2*	Ash	*Aedes albopictus*
AALF020875	*Set8*	-	*Aedes albopictus*
AALF026593	*Smyd4-1*	SMYD	*Aedes albopictus*
AALF027384	*Smyd4-2*	SMYD	*Aedes albopictus*
AALF021455	*Smyd4-3*	SMYD	*Aedes albopictus*
AALF018911	*Smyd4-4*	SMYD	*Aedes albopictus*
AALF004458	*Smyd4-4*	SMYD	*Aedes albopictus*
AALF006249	*Smyd4-4*	SMYD	*Aedes albopictus*
AALF015442	*Smyd4-4*	SMYD	*Aedes albopictus*
AALF015443	*Smyd4-4*	SMYD	*Aedes albopictus*
AALF025730	*Smyd4-4*	SMYD	*Aedes albopictus*
AALF026554	*Smyd4-4*	SMYD	*Aedes albopictus*
AALF016668	*Smyd5*	SMYD	*Aedes albopictus*
AALF022548	*Smyd5*	SMYD	*Aedes albopictus*
AALF008460	*SmydA-1*	SmydA	*Aedes albopictus*
AALF003791	*SmydA-1*	SmydA	*Aedes albopictus*
AALF003405	*SmydA-2*	SmydA	*Aedes albopictus*
AALF003793	*SmydA-4*	SmydA	*Aedes albopictus*
AALF008462	*SmydA-4*	SmydA	*Aedes albopictus*
AALF018336	*SmydA-5*	SmydA	*Aedes albopictus*
AALF025243	*SmydA-6*	SmydA	*Aedes albopictus*
AALF014059	*SmydA-8*	SmydA	*Aedes albopictus*
AALF025991	*Su(var)3-9*	Suv	*Aedes albopictus*
AALF017609	*trr*	Trx	*Aedes albopictus*
AALF015214	*trx*	Trx	*Aedes albopictus*
AALF009731	*trx*	Trx	*Aedes albopictus*
AALF014731	*upSET(MLL5)*	-	*Aedes albopictus*
APLA000918	-	-	*Agrilus planipennis*
APLA001309	*ash1*	Ash	*Agrilus planipennis*
APLA008065	*Blimp-1*	PRDM	*Agrilus planipennis*
APLA013892	*Blimp-1*	PRDM	*Agrilus planipennis*
APLA000603	*egg*	Suv	*Agrilus planipennis*
APLA013399	*Ez*	Ez	*Agrilus planipennis*
APLA012795	*Hmt4-20*	-	*Agrilus planipennis*
APLA003315	*Mes-4*	Ash	*Agrilus planipennis*
APLA009436	*Set1*	Trx	*Agrilus planipennis*
APLA003597	*Set2*	Ash	*Agrilus planipennis*
APLA008756	*Set8*	-	*Agrilus planipennis*
APLA014062	*setd*	SETD	*Agrilus planipennis*
APLA012316	*Smyd4-2*	SMYD	*Agrilus planipennis*

APLA006512	*Smyd4-3*	SMYD	*Agrilus planipennis*
APLA001020	*Smyd4-4*	SMYD	*Agrilus planipennis*
APLA010567	*Smyd5*	SMYD	*Agrilus planipennis*
APLA007169	*SmydA-1*	SmydA	*Agrilus planipennis*
APLA012086	*SmydA-3*	SmydA	*Agrilus planipennis*
APLA000387	*SmydA-4*	SmydA	*Agrilus planipennis*
APLA005739	*SmydA-5*	SmydA	*Agrilus planipennis*
APLA005059	*SmydA-6*	SmydA	*Agrilus planipennis*
APLA007967	*Su(var)3-9*	Suv	*Agrilus planipennis*
APLA011917	*suv*	Suv	*Agrilus planipennis*
APLA009473	*trr*	Trx	*Agrilus planipennis*
APLA006438	*trx*	Trx	*Agrilus planipennis*
APLA012611	*upSET(MLL5)*	-	*Agrilus planipennis*
AALB004147	-	-	*Anopheles albimanus*
AALB009144	*ash1*	Ash	*Anopheles albimanus*
AALB005061	*Blimp-1*	PRDM	*Anopheles albimanus*
AALB000320	*egg*	Suv	*Anopheles albimanus*
AALB007836	*Ez*	Ez	*Anopheles albimanus*
AALB009123	*G9a*	Suv	*Anopheles albimanus*
AALB007777	*Hmt4-20*	-	*Anopheles albimanus*
AALB002890	*Mes-4*	Ash	*Anopheles albimanus*
AALB009646	*Set1*	Trx	*Anopheles albimanus*
AALB010325	*Set2*	Ash	*Anopheles albimanus*
AALB007539	*Set8*	-	*Anopheles albimanus*
AALB007130	*setd*	SETD	*Anopheles albimanus*
AALB000850	*Smyd4-1*	SMYD	*Anopheles albimanus*
AALB009107	*Smyd4-2*	SMYD	*Anopheles albimanus*
AALB010491	*Smyd4-2*	SMYD	*Anopheles albimanus*
AALB000467	*Smyd4-3*	SMYD	*Anopheles albimanus*
AALB010380	*Smyd4-4*	SMYD	*Anopheles albimanus*
AALB010372	*Smyd4-4*	SMYD	*Anopheles albimanus*
AALB002270	*Smyd5*	SMYD	*Anopheles albimanus*
AALB010154	*SmydA-1*	SmydA	*Anopheles albimanus*
AALB007436	*SmydA-1*	SmydA	*Anopheles albimanus*
AALB003310	*SmydA-2*	SmydA	*Anopheles albimanus*
AALB010147	*SmydA-4*	SmydA	*Anopheles albimanus*
AALB001224	*SmydA-5*	SmydA	*Anopheles albimanus*
AALB001227	*SmydA-6*	SmydA	*Anopheles albimanus*
AALB007778	*SmydA-8*	SmydA	*Anopheles albimanus*
AALB008184	*Su(var)3-9*	Suv	*Anopheles albimanus*
AALB010678	*trr*	Trx	*Anopheles albimanus*
AALB009472	*trx*	Trx	*Anopheles albimanus*
AALB010198	*upSET(MLL5)*	-	*Anopheles albimanus*
AARA001981	*ash1*	Ash	*Anopheles arabiensis*

AARA008466	*Blimp-1*	PRDM	*Anopheles arabiensis*
AARA005146	*egg*	Suv	*Anopheles arabiensis*
AARA009882	*Ez*	Ez	*Anopheles arabiensis*
AARA004840	*G9a*	Suv	*Anopheles arabiensis*
AARA001140	*Hmt4-20*	-	*Anopheles arabiensis*
AARA010201	*Mes-4*	Ash	*Anopheles arabiensis*
AARA006692	*Set1*	Trx	*Anopheles arabiensis*
AARA000494	*Set2*	Ash	*Anopheles arabiensis*
AARA012095	*Set8*	-	*Anopheles arabiensis*
AARA000873	*setd*	SETD	*Anopheles arabiensis*
AARA009514	*Smyd3*	SMYD	*Anopheles arabiensis*
AARA009535	*Smyd4-1*	SMYD	*Anopheles arabiensis*
AARA004823	*Smyd4-2*	SMYD	*Anopheles arabiensis*
AARA009380	*Smyd4-3*	SMYD	*Anopheles arabiensis*
AARA000650	*Smyd4-4*	SMYD	*Anopheles arabiensis*
AARA000156	*Smyd4-4*	SMYD	*Anopheles arabiensis*
AARA000157	*Smyd4-4*	SMYD	*Anopheles arabiensis*
AARA000158	*Smyd4-4*	SMYD	*Anopheles arabiensis*
AARA000159	*Smyd4-4*	SMYD	*Anopheles arabiensis*
AARA000160	*Smyd4-4*	SMYD	*Anopheles arabiensis*
AARA000189	*Smyd4-4*	SMYD	*Anopheles arabiensis*
AARA002920	*Smyd4-4*	SMYD	*Anopheles arabiensis*
AARA006296	*Smyd4-4*	SMYD	*Anopheles arabiensis*
AARA010547	*Smyd4-4*	SMYD	*Anopheles arabiensis*
AARA006612	*Smyd5*	SMYD	*Anopheles arabiensis*
AARA002766	*SmydA-1*	SmydA	*Anopheles arabiensis*
AARA011762	*SmydA-2*	SmydA	*Anopheles arabiensis*
AARA011975	*SmydA-3*	SmydA	*Anopheles arabiensis*
AARA002772	*SmydA-4*	SmydA	*Anopheles arabiensis*
AARA005867	*SmydA-5*	SmydA	*Anopheles arabiensis*
AARA005864	*SmydA-6*	SmydA	*Anopheles arabiensis*
AARA001141	*SmydA-8*	SmydA	*Anopheles arabiensis*
AARA006655	*Su(var)3-9*	Suv	*Anopheles arabiensis*
AARA000113	*trr*	Trx	*Anopheles arabiensis*
AARA004103	*trx*	Trx	*Anopheles arabiensis*
AARA003322	*upSET(MLL5)*	-	*Anopheles arabiensis*
AATE001986	*ash1*	Ash	*Anopheles atroparvus*
AATE010559	*Blimp-1*	PRDM	*Anopheles atroparvus*
AATE010215	*Blimp-1*	PRDM	*Anopheles atroparvus*
AATE010754	*egg*	Suv	*Anopheles atroparvus*
AATE012517	*Ez*	Ez	*Anopheles atroparvus*
AATE018312	*G9a*	Suv	*Anopheles atroparvus*
AATE021144	*Hmt4-20*	-	*Anopheles atroparvus*
AATE002579	*Hmt4-20*	-	*Anopheles atroparvus*

AATE009362	*Mes-4*	Ash	*Anopheles atroparvus*
AATE011750	*Set1*	Trx	*Anopheles atroparvus*
AATE013785	*Set2*	Ash	*Anopheles atroparvus*
AATE001322	*Set8*	-	*Anopheles atroparvus*
AATE007609	*setd*	SETD	*Anopheles atroparvus*
AATE012285	*Smyd4-2*	SMYD	*Anopheles atroparvus*
AATE018332	*Smyd4-3*	SMYD	*Anopheles atroparvus*
AATE018076	*Smyd4-3*	SMYD	*Anopheles atroparvus*
AATE008287	*Smyd4-4*	SMYD	*Anopheles atroparvus*
AATE010823	*Smyd4-4*	SMYD	*Anopheles atroparvus*
AATE017046	*Smyd5*	SMYD	*Anopheles atroparvus*
AATE014042	*SmydA-1*	SmydA	*Anopheles atroparvus*
AATE000280	*SmydA-2*	SmydA	*Anopheles atroparvus*
AATE020573	*SmydA-4*	SmydA	*Anopheles atroparvus*
AATE008651	*SmydA-8*	SmydA	*Anopheles atroparvus*
AATE007975	*trr*	Trx	*Anopheles atroparvus*
AATE019914	*trx*	Trx	*Anopheles atroparvus*
AATE015475	*upSET(MLL5)*	-	*Anopheles atroparvus*
ACHR004150	*ash1*	Ash	*Anopheles christyi*
ACHR005333	*Blimp-1*	PRDM	*Anopheles christyi*
ACHR003313	*egg*	Suv	*Anopheles christyi*
ACHR006274	*Ez*	Ez	*Anopheles christyi*
ACHR004732	*G9a*	Suv	*Anopheles christyi*
ACHR005184	*Hmt4-20*	-	*Anopheles christyi*
ACHR003025	*Mes-4*	Ash	*Anopheles christyi*
ACHR001688	*Set1*	Trx	*Anopheles christyi*
ACHR002893	*Set2*	Ash	*Anopheles christyi*
ACHR006197	*Set8*	-	*Anopheles christyi*
ACHR004534	*setd*	SETD	*Anopheles christyi*
ACHR009346	*Smyd4-1*	SMYD	*Anopheles christyi*
ACHR003333	*Smyd4-2*	SMYD	*Anopheles christyi*
ACHR003962	*Smyd4-3*	SMYD	*Anopheles christyi*
ACHR003961	*Smyd4-3*	SMYD	*Anopheles christyi*
ACHR003375	*Smyd4-4*	SMYD	*Anopheles christyi*
ACHR006505	*Smyd4-4*	SMYD	*Anopheles christyi*
ACHR008883	*Smyd4-4*	SMYD	*Anopheles christyi*
ACHR001476	*Smyd5*	SMYD	*Anopheles christyi*
ACHR002501	*SmydA-1*	SmydA	*Anopheles christyi*
ACHR009347	*SmydA-2*	SmydA	*Anopheles christyi*
ACHR006880	*SmydA-3*	SmydA	*Anopheles christyi*
ACHR005153	*SmydA-4*	SmydA	*Anopheles christyi*
ACHR000441	*SmydA-5*	SmydA	*Anopheles christyi*
ACHR000444	*SmydA-6*	SmydA	*Anopheles christyi*
ACHR001130	*Su(var)3-9*	Suv	*Anopheles christyi*

ACHR000033	*trr*	Trx	*Anopheles christyi*
ACHR000512	*trx*	Trx	*Anopheles christyi*
ACHR002873	*upSET(MLL5)*	-	*Anopheles christyi*
ACOM022824	-	-	*Anopheles coluzzii*
ACOM039862	-	-	*Anopheles coluzzii*
ACOM034264	*ash1*	Ash	*Anopheles coluzzii*
ACOM039487	*Blimp-1*	PRDM	*Anopheles coluzzii*
ACOM030721	*egg*	Suv	*Anopheles coluzzii*
ACOM036272	*Ez*	Ez	*Anopheles coluzzii*
ACOM037293	*G9a*	Suv	*Anopheles coluzzii*
ACOM041819	*Mes-4*	Ash	*Anopheles coluzzii*
ACOM031842	*Mes-4*	Ash	*Anopheles coluzzii*
ACOM041980	*Set1*	Trx	*Anopheles coluzzii*
ACOM038701	*Set8*	-	*Anopheles coluzzii*
ACOM037868	*setd*	SETD	*Anopheles coluzzii*
ACOM040598	*Smyd3*	SMYD	*Anopheles coluzzii*
ACOM040545	*Smyd4-1*	SMYD	*Anopheles coluzzii*
ACOM037316	*Smyd4-2*	SMYD	*Anopheles coluzzii*
ACOM024367	*Smyd4-3*	SMYD	*Anopheles coluzzii*
ACOM024363	*Smyd4-3*	SMYD	*Anopheles coluzzii*
ACOM035881	*Smyd4-3*	SMYD	*Anopheles coluzzii*
ACOM032081	*Smyd4-4*	SMYD	*Anopheles coluzzii*
ACOM026706	*Smyd4-4*	SMYD	*Anopheles coluzzii*
ACOM026707	*Smyd4-4*	SMYD	*Anopheles coluzzii*
ACOM032704	*Smyd4-4*	SMYD	*Anopheles coluzzii*
ACOM034648	*Smyd4-4*	SMYD	*Anopheles coluzzii*
ACOM038104	*Smyd4-4*	SMYD	*Anopheles coluzzii*
ACOM038110	*Smyd4-4*	SMYD	*Anopheles coluzzii*
ACOM038111	*Smyd4-4*	SMYD	*Anopheles coluzzii*
ACOM028568	*Smyd5*	SMYD	*Anopheles coluzzii*
ACOM041370	*SmydA-1*	SmydA	*Anopheles coluzzii*
ACOM038485	*SmydA-2*	SmydA	*Anopheles coluzzii*
ACOM031027	*SmydA-3*	SmydA	*Anopheles coluzzii*
ACOM041390	*SmydA-4*	SmydA	*Anopheles coluzzii*
ACOM028644	*Su(var)3-9*	Suv	*Anopheles coluzzii*
ACOM038199	*trr*	Trx	*Anopheles coluzzii*
ACOM036432	*trx*	Trx	*Anopheles coluzzii*
ACOM026402	*upSET(MLL5)*	-	*Anopheles coluzzii*
ACUA005703	*ash1*	Ash	*Anopheles culicifacies*
ACUA020082	*Blimp-1*	PRDM	*Anopheles culicifacies*
ACUA006607	*egg*	Suv	*Anopheles culicifacies*
ACUA021465	*Ez*	Ez	*Anopheles culicifacies*
ACUA002190	*G9a*	Suv	*Anopheles culicifacies*
ACUA027774	*Hmt4-20*	-	*Anopheles culicifacies*

ACUA005751	*Mes-4*	Ash	*Anopheles culicifacies*
ACUA004163	*Set1*	Trx	*Anopheles culicifacies*
ACUA013249	*Set2*	Ash	*Anopheles culicifacies*
ACUA026085	*Set8*	-	*Anopheles culicifacies*
ACUA024866	*setd*	SETD	*Anopheles culicifacies*
ACUA001952	*Smyd3*	SMYD	*Anopheles culicifacies*
ACUA002159	*Smyd4-1*	SMYD	*Anopheles culicifacies*
ACUA005214	*Smyd4-2*	SMYD	*Anopheles culicifacies*
ACUA004563	*Smyd4-3*	SMYD	*Anopheles culicifacies*
ACUA002830	*Smyd4-4*	SMYD	*Anopheles culicifacies*
ACUA001970	*Smyd5*	SMYD	*Anopheles culicifacies*
ACUA015444	*SmydA-1*	SmydA	*Anopheles culicifacies*
ACUA012854	*SmydA-2*	SmydA	*Anopheles culicifacies*
ACUA016822	*SmydA-3*	SmydA	*Anopheles culicifacies*
ACUA007419	*SmydA-4*	SmydA	*Anopheles culicifacies*
ACUA010941	*SmydA-5*	SmydA	*Anopheles culicifacies*
ACUA017098	*SmydA-6*	SmydA	*Anopheles culicifacies*
ACUA019898	*SmydA-8*	SmydA	*Anopheles culicifacies*
ACUA002140	*Su(var)3-9*	Suv	*Anopheles culicifacies*
ACUA015706	*trr*	Trx	*Anopheles culicifacies*
ACUA018535	*trx*	Trx	*Anopheles culicifacies*
ACUA010722	*upSET(MLL5)*	-	*Anopheles culicifacies*
ADAC005679	-	-	*Anopheles darlingi*
ADAC005034	*ash1*	Ash	*Anopheles darlingi*
ADAC005262	*Blimp-1*	PRDM	*Anopheles darlingi*
ADAC006910	*egg*	Suv	*Anopheles darlingi*
ADAC009901	*G9a*	Suv	*Anopheles darlingi*
ADAC000673	*Hmt4-20*	-	*Anopheles darlingi*
ADAC004648	*Mes-4*	Ash	*Anopheles darlingi*
ADAC009677	*Set1*	Trx	*Anopheles darlingi*
ADAC001196	*Set2*	Ash	*Anopheles darlingi*
ADAC006491	*Set8*	-	*Anopheles darlingi*
ADAC000960	*Smyd4-1*	SMYD	*Anopheles darlingi*
ADAC009912	*Smyd4-2*	SMYD	*Anopheles darlingi*
ADAC002530	*Smyd4-3*	SMYD	*Anopheles darlingi*
ADAC002531	*Smyd4-3*	SMYD	*Anopheles darlingi*
ADAC000007	*Smyd4-4*	SMYD	*Anopheles darlingi*
ADAC002843	*Smyd4-4*	SMYD	*Anopheles darlingi*
ADAC000301	*Smyd5*	SMYD	*Anopheles darlingi*
ADAC007277	*SmydA-1*	SmydA	*Anopheles darlingi*
ADAC000885	*SmydA-1*	SmydA	*Anopheles darlingi*
ADAC008279	*SmydA-2*	SmydA	*Anopheles darlingi*
ADAC003680	*SmydA-4*	SmydA	*Anopheles darlingi*
ADAC006043	*SmydA-5*	SmydA	*Anopheles darlingi*

ADAC006045	*SmydA-6*	SmydA	*Anopheles darlingi*
ADAC000669	*SmydA-8*	SmydA	*Anopheles darlingi*
ADAC003826	*Su(var)3-9*	Suv	*Anopheles darlingi*
ADAC008315	*trr*	Trx	*Anopheles darlingi*
ADAC008354	*trx*	Trx	*Anopheles darlingi*
ADAC003698	*upSET(MLL5)*	-	*Anopheles darlingi*
ADIR009886	*ash1*	Ash	*Anopheles dirus*
ADIR007386	*Blimp-1*	PRDM	*Anopheles dirus*
ADIR010998	*egg*	Suv	*Anopheles dirus*
ADIR009828	*Ez*	Ez	*Anopheles dirus*
ADIR003112	*G9a*	Suv	*Anopheles dirus*
ADIR001253	*Hmt4-20*	-	*Anopheles dirus*
ADIR009088	*Mes-4*	Ash	*Anopheles dirus*
ADIR008928	*Set1*	Trx	*Anopheles dirus*
ADIR001842	*Set2*	Ash	*Anopheles dirus*
ADIR003903	*Set8*	-	*Anopheles dirus*
ADIR003127	*Smyd4-2*	SMYD	*Anopheles dirus*
ADIR002333	*Smyd4-2*	SMYD	*Anopheles dirus*
ADIR011385	*Smyd4-3*	SMYD	*Anopheles dirus*
ADIR002334	*Smyd4-4*	SMYD	*Anopheles dirus*
ADIR000001	*Smyd4-4*	SMYD	*Anopheles dirus*
ADIR000003	*Smyd4-4*	SMYD	*Anopheles dirus*
ADIR000068	*Smyd4-4*	SMYD	*Anopheles dirus*
ADIR001442	*Smyd4-4*	SMYD	*Anopheles dirus*
ADIR002342	*Smyd4-4*	SMYD	*Anopheles dirus*
ADIR011543	*Smyd4-4*	SMYD	*Anopheles dirus*
ADIR010677	*Smyd5*	SMYD	*Anopheles dirus*
ADIR000317	*SmydA-1*	SmydA	*Anopheles dirus*
ADIR002369	*SmydA-2*	SmydA	*Anopheles dirus*
ADIR000311	*SmydA-4*	SmydA	*Anopheles dirus*
ADIR005591	*SmydA-5*	SmydA	*Anopheles dirus*
ADIR005594	*SmydA-6*	SmydA	*Anopheles dirus*
ADIR001254	*SmydA-8*	SmydA	*Anopheles dirus*
ADIR002622	*Su(var)3-9*	Suv	*Anopheles dirus*
ADIR000099	*trr*	Trx	*Anopheles dirus*
ADIR009916	*trx*	Trx	*Anopheles dirus*
ADIR009538	*upSET(MLL5)*	-	*Anopheles dirus*
AEPI006170	*ash1*	Ash	*Anopheles epiroticus*
AEPI008254	*Blimp-1*	PRDM	*Anopheles epiroticus*
AEPI004820	*egg*	Suv	*Anopheles epiroticus*
AEPI010708	*Ez*	Ez	*Anopheles epiroticus*
AEPI002277	*G9a*	Suv	*Anopheles epiroticus*
AEPI008601	*Hmt4-20*	-	*Anopheles epiroticus*
AEPI010018	*Mes-4*	Ash	*Anopheles epiroticus*

AEPI004718	*Set1*	Trx	*Anopheles epiroticus*
AEPI004533	*Set2*	Ash	*Anopheles epiroticus*
AEPI004984	*Set8*	-	*Anopheles epiroticus*
AEPI005413	*setd*	SETD	*Anopheles epiroticus*
AEPI008133	*Smyd3*	SMYD	*Anopheles epiroticus*
AEPI011149	*Smyd4-1*	SMYD	*Anopheles epiroticus*
AEPI007491	*Smyd4-2*	SMYD	*Anopheles epiroticus*
AEPI008125	*Smyd4-3*	SMYD	*Anopheles epiroticus*
AEPI011500	*Smyd4-4*	SMYD	*Anopheles epiroticus*
AEPI001344	*Smyd4-4*	SMYD	*Anopheles epiroticus*
AEPI006916	*Smyd4-4*	SMYD	*Anopheles epiroticus*
AEPI006256	*Smyd5*	SMYD	*Anopheles epiroticus*
AEPI010529	*SmydA-1*	SmydA	*Anopheles epiroticus*
AEPI006589	*SmydA-2*	SmydA	*Anopheles epiroticus*
AEPI007725	*SmydA-4*	SmydA	*Anopheles epiroticus*
AEPI010927	*SmydA-5*	SmydA	*Anopheles epiroticus*
AEPI010929	*SmydA-6*	SmydA	*Anopheles epiroticus*
AEPI008602	*SmydA-8*	SmydA	*Anopheles epiroticus*
AEPI003432	*Su(var)3-9*	Suv	*Anopheles epiroticus*
AEPI000069	*trr*	Trx	*Anopheles epiroticus*
AEPI010594	*trx*	Trx	*Anopheles epiroticus*
AEPI004325	*upSET(MLL5)*	-	*Anopheles epiroticus*
AFAF004540	-	-	*Anopheles farauti*
AFAF012871	*ash1*	Ash	*Anopheles farauti*
AFAF006673	*Blimp-1*	PRDM	*Anopheles farauti*
AFAF000218	*egg*	Suv	*Anopheles farauti*
AFAF007019	*Ez*	Ez	*Anopheles farauti*
AFAF014974	*G9a*	Suv	*Anopheles farauti*
AFAF003732	*Hmt4-20*	-	*Anopheles farauti*
AFAF010905	*Mes-4*	Ash	*Anopheles farauti*
AFAF014140	*Set1*	Trx	*Anopheles farauti*
AFAF010271	*Set2*	Ash	*Anopheles farauti*
AFAF005239	*Set8*	-	*Anopheles farauti*
AFAF003061	*setd*	SETD	*Anopheles farauti*
AFAF016704	*Smyd4-2*	SMYD	*Anopheles farauti*
AFAF016027	*Smyd4-3*	SMYD	*Anopheles farauti*
AFAF020148	*Smyd4-4*	SMYD	*Anopheles farauti*
AFAF000640	*Smyd4-4*	SMYD	*Anopheles farauti*
AFAF002619	*Smyd5*	SMYD	*Anopheles farauti*
AFAF016575	*SmydA-1*	SmydA	*Anopheles farauti*
AFAF014816	*SmydA-2*	SmydA	*Anopheles farauti*
AFAF018626	*SmydA-4*	SmydA	*Anopheles farauti*
AFAF005036	*SmydA-8*	SmydA	*Anopheles farauti*
AFAF013256	*Su(var)3-9*	Suv	*Anopheles farauti*

AFAF010066	*trr*	Trx	*Anopheles farauti*
AFAF016439	*trx*	Trx	*Anopheles farauti*
AFUN001211	*ash1*	Ash	*Anopheles funestus*
AFUN007598	*Blimp-1*	PRDM	*Anopheles funestus*
AFUN006312	*egg*	Suv	*Anopheles funestus*
AFUN006519	*Ez*	Ez	*Anopheles funestus*
AFUN007895	*G9a*	Suv	*Anopheles funestus*
AFUN000188	*Hmt4-20*	-	*Anopheles funestus*
AFUN004497	*Mes-4*	Ash	*Anopheles funestus*
AFUN005553	*Set1*	Trx	*Anopheles funestus*
AFUN001027	*Set2*	Ash	*Anopheles funestus*
AFUN009140	*Set8*	-	*Anopheles funestus*
AFUN006555	*Smyd3*	SMYD	*Anopheles funestus*
AFUN011643	*Smyd4-1*	SMYD	*Anopheles funestus*
AFUN007912	*Smyd4-2*	SMYD	*Anopheles funestus*
AFUN000998	*Smyd4-3*	SMYD	*Anopheles funestus*
AFUN002591	*Smyd4-4*	SMYD	*Anopheles funestus*
AFUN002599	*Smyd4-4*	SMYD	*Anopheles funestus*
AFUN011040	*Smyd4-4*	SMYD	*Anopheles funestus*
AFUN011934	*Smyd4-4*	SMYD	*Anopheles funestus*
AFUN008689	*SmydA-1*	SmydA	*Anopheles funestus*
AFUN008074	*SmydA-2*	SmydA	*Anopheles funestus*
AFUN002498	*SmydA-4*	SmydA	*Anopheles funestus*
AFUN002646	*SmydA-5*	SmydA	*Anopheles funestus*
AFUN002649	*SmydA-6*	SmydA	*Anopheles funestus*
AFUN000189	*SmydA-8*	SmydA	*Anopheles funestus*
AFUN008215	*Su(var)3-9*	Suv	*Anopheles funestus*
AFUN009566	*trr*	Trx	*Anopheles funestus*
AFUN009401	*trx*	Trx	*Anopheles funestus*
AFUN005366	*upSET(MLL5)*	-	*Anopheles funestus*
AGAP010931	-	-	*Anopheles gambiae*
AGAP001535	*ash1*	Ash	*Anopheles gambiae*
AGAP006592	*Blimp-1*	PRDM	*Anopheles gambiae*
AGAP007978	*egg*	Suv	*Anopheles gambiae*
AGAP012516	*Ez*	Ez	*Anopheles gambiae*
AGAP003013	*G9a*	Suv	*Anopheles gambiae*
AGAP000042	*Hmt4-20*	-	*Anopheles gambiae*
AGAP004656	*Mes-4*	Ash	*Anopheles gambiae*
AGAP002246	*Set1*	Trx	*Anopheles gambiae*
AGAP011688	*Set2*	Ash	*Anopheles gambiae*
AGAP012610	*Set8*	-	*Anopheles gambiae*
AGAP013401	*setd*	SETD	*Anopheles gambiae*
AGAP008954	*Smyd3*	SMYD	*Anopheles gambiae*
AGAP008973	*Smyd4-1*	SMYD	*Anopheles gambiae*

AGAP002999	*Smyd4-2*	SMYD	*Anopheles gambiae*
AGAP008839	*Smyd4-3*	SMYD	*Anopheles gambiae*
AGAP008840	*Smyd4-3*	SMYD	*Anopheles gambiae*
AGAP011835	*Smyd4-4*	SMYD	*Anopheles gambiae*
AGAP000216	*Smyd4-4*	SMYD	*Anopheles gambiae*
AGAP005253	*Smyd4-4*	SMYD	*Anopheles gambiae*
AGAP009448	*Smyd4-4*	SMYD	*Anopheles gambiae*
AGAP009449	*Smyd4-4*	SMYD	*Anopheles gambiae*
AGAP011232	*Smyd4-4*	SMYD	*Anopheles gambiae*
AGAP011234	*Smyd4-4*	SMYD	*Anopheles gambiae*
AGAP011235	*Smyd4-4*	SMYD	*Anopheles gambiae*
AGAP011237	*Smyd4-4*	SMYD	*Anopheles gambiae*
AGAP011238	*Smyd4-4*	SMYD	*Anopheles gambiae*
AGAP011257	*Smyd4-4*	SMYD	*Anopheles gambiae*
AGAP011267	*Smyd4-4*	SMYD	*Anopheles gambiae*
AGAP012638	*Smyd4-4*	SMYD	*Anopheles gambiae*
AGAP003552	*Smyd5*	SMYD	*Anopheles gambiae*
AGAP011530	*SmydA-1*	SmydA	*Anopheles gambiae*
AGAP001025	*SmydA-2*	SmydA	*Anopheles gambiae*
AGAP007779	*SmydA-3*	SmydA	*Anopheles gambiae*
AGAP011523	*SmydA-4*	SmydA	*Anopheles gambiae*
AGAP010377	*SmydA-6*	SmydA	*Anopheles gambiae*
AGAP000041	*SmydA-8*	SmydA	*Anopheles gambiae*
AGAP003597	*Su(var)3-9*	Suv	*Anopheles gambiae*
AGAP011192	*trr*	Trx	*Anopheles gambiae*
AGAP002741	*trx*	Trx	*Anopheles gambiae*
AGAP012236	*upSET(MLL5)*	-	*Anopheles gambiae*
AMAM002900	-	-	*Anopheles maculatus*
AMAM020763	*ash1*	Ash	*Anopheles maculatus*
AMAM018700	*Blimp-1*	PRDM	*Anopheles maculatus*
AMAM008031	*Blimp-1*	PRDM	*Anopheles maculatus*
AMAM007096	*egg*	Suv	*Anopheles maculatus*
AMAM005984	*Ez*	Ez	*Anopheles maculatus*
AMAM015900	*G9a*	Suv	*Anopheles maculatus*
AMAM006905	*Hmt4-20*	-	*Anopheles maculatus*
AMAM017586	*Mes-4*	Ash	*Anopheles maculatus*
AMAM021419	*Set1*	Trx	*Anopheles maculatus*
AMAM001199	*Set2*	Ash	*Anopheles maculatus*
AMAM020524	*Set8*	-	*Anopheles maculatus*
AMAM001066	*Smyd4-1*	SMYD	*Anopheles maculatus*
AMAM001162	*Smyd4-2*	SMYD	*Anopheles maculatus*
AMAM018902	*Smyd4-3*	SMYD	*Anopheles maculatus*
AMAM020244	*Smyd4-3*	SMYD	*Anopheles maculatus*
AMAM007489	*Smyd4-4*	SMYD	*Anopheles maculatus*

AMAM022944	*Smyd4-4*	SMYD	*Anopheles maculatus*
AMAM023423	*Smyd5*	SMYD	*Anopheles maculatus*
AMAM005241	*SmydA-1*	SmydA	*Anopheles maculatus*
AMAM002801	*SmydA-4*	SmydA	*Anopheles maculatus*
AMAM017767	*SmydA-5*	SmydA	*Anopheles maculatus*
AMAM009817	*SmydA-6*	SmydA	*Anopheles maculatus*
AMAM020034	*SmydA-8*	SmydA	*Anopheles maculatus*
AMAM020583	*Su(var)3-9*	Suv	*Anopheles maculatus*
AMAM007797	*trr*	Trx	*Anopheles maculatus*
AMAM004679	*trx*	Trx	*Anopheles maculatus*
AMAM000855	*upSET(MLL5)*	-	*Anopheles maculatus*
AMEC013710	-	-	*Anopheles melas*
AMEC000956	*ash1*	Ash	*Anopheles melas*
AMEC005588	*Blimp-1*	PRDM	*Anopheles melas*
AMEC011999	*egg*	Suv	*Anopheles melas*
AMEC009964	*Ez*	Ez	*Anopheles melas*
AMEC018244	*G9a*	Suv	*Anopheles melas*
AMEC012967	*Hmt4-20*	-	*Anopheles melas*
AMEC010344	*Set1*	Trx	*Anopheles melas*
AMEC021762	*Set8*	-	*Anopheles melas*
AMEC002500	*setd*	SETD	*Anopheles melas*
AMEC010777	*Smyd3*	SMYD	*Anopheles melas*
AMEC002271	*Smyd4-1*	SMYD	*Anopheles melas*
AMEC008223	*Smyd4-2*	SMYD	*Anopheles melas*
AMEC006734	*Smyd4-3*	SMYD	*Anopheles melas*
AMEC015873	*Smyd4-3*	SMYD	*Anopheles melas*
AMEC020580	*Smyd4-3*	SMYD	*Anopheles melas*
AMEC018820	*Smyd4-4*	SMYD	*Anopheles melas*
AMEC002177	*Smyd4-4*	SMYD	*Anopheles melas*
AMEC003887	*Smyd4-4*	SMYD	*Anopheles melas*
AMEC007489	*Smyd4-4*	SMYD	*Anopheles melas*
AMEC009251	*Smyd4-4*	SMYD	*Anopheles melas*
AMEC010448	*Smyd4-4*	SMYD	*Anopheles melas*
AMEC018563	*Smyd4-4*	SMYD	*Anopheles melas*
AMEC019207	*Smyd4-4*	SMYD	*Anopheles melas*
AMEC011282	*Smyd5*	SMYD	*Anopheles melas*
AMEC014136	*SmydA-1*	SmydA	*Anopheles melas*
AMEC001953	*SmydA-2*	SmydA	*Anopheles melas*
AMEC017226	*SmydA-3*	SmydA	*Anopheles melas*
AMEC007461	*SmydA-4*	SmydA	*Anopheles melas*
AMEC017763	*SmydA-8*	SmydA	*Anopheles melas*
AMEC005849	*Su(var)3-9*	Suv	*Anopheles melas*
AMEC016519	*trr*	Trx	*Anopheles melas*
AMEC012661	*trx*	Trx	*Anopheles melas*

AMEC010292	*upSET(MLL5)*	-	*Anopheles melas*
AMEM001699	-	-	*Anopheles merus*
AMEM011814	-	-	*Anopheles merus*
AMEM016754	-	-	*Anopheles merus*
AMEM017977	-	-	*Anopheles merus*
AMEM011678	*ash1*	Ash	*Anopheles merus*
AMEM000073	*Blimp-1*	PRDM	*Anopheles merus*
AMEM015377	*egg*	Suv	*Anopheles merus*
AMEM002929	*G9a*	Suv	*Anopheles merus*
AMEM008445	*Hmt4-20*	-	*Anopheles merus*
AMEM012160	*Mes-4*	Ash	*Anopheles merus*
AMEM010011	*Set1*	Trx	*Anopheles merus*
AMEM016351	*Set2*	Ash	*Anopheles merus*
AMEM001840	*Set8*	-	*Anopheles merus*
AMEM016838	*setd*	SETD	*Anopheles merus*
AMEM017677	*Smyd3*	SMYD	*Anopheles merus*
AMEM006609	*Smyd4-1*	SMYD	*Anopheles merus*
AMEM017156	*Smyd4-2*	SMYD	*Anopheles merus*
AMEM003006	*Smyd4-3*	SMYD	*Anopheles merus*
AMEM016185	*Smyd4-3*	SMYD	*Anopheles merus*
AMEM007139	*Smyd4-4*	SMYD	*Anopheles merus*
AMEM000956	*Smyd4-4*	SMYD	*Anopheles merus*
AMEM001976	*Smyd4-4*	SMYD	*Anopheles merus*
AMEM004974	*Smyd4-4*	SMYD	*Anopheles merus*
AMEM006937	*Smyd4-4*	SMYD	*Anopheles merus*
AMEM007787	*Smyd4-4*	SMYD	*Anopheles merus*
AMEM009150	*Smyd4-4*	SMYD	*Anopheles merus*
AMEM011685	*Smyd4-4*	SMYD	*Anopheles merus*
AMEM016083	*Smyd4-4*	SMYD	*Anopheles merus*
AMEM004690	*Smyd5*	SMYD	*Anopheles merus*
AMEM017633	*SmydA-1*	SmydA	*Anopheles merus*
AMEM005030	*SmydA-2*	SmydA	*Anopheles merus*
AMEM004977	*SmydA-3*	SmydA	*Anopheles merus*
AMEM008766	*SmydA-4*	SmydA	*Anopheles merus*
AMEM005433	*SmydA-8*	SmydA	*Anopheles merus*
AMEM015759	*Su(var)3-9*	Suv	*Anopheles merus*
AMEM009860	*trr*	Trx	*Anopheles merus*
AMIN002426	*ash1*	Ash	*Anopheles minimus*
AMIN007801	*Blimp-1*	PRDM	*Anopheles minimus*
AMIN006287	*egg*	Suv	*Anopheles minimus*
AMIN007508	*Ez*	Ez	*Anopheles minimus*
AMIN003720	*G9a*	Suv	*Anopheles minimus*
AMIN002103	*Hmt4-20*	-	*Anopheles minimus*
AMIN000101	*Mes-4*	Ash	*Anopheles minimus*

AMIN000158	*Set1*	Trx	*Anopheles minimus*
AMIN008770	*Set2*	Ash	*Anopheles minimus*
AMIN006460	*Set8*	-	*Anopheles minimus*
AMIN008001	*setd*	SETD	*Anopheles minimus*
AMIN009400	*Smyd3*	SMYD	*Anopheles minimus*
AMIN009414	*Smyd4-1*	SMYD	*Anopheles minimus*
AMIN003703	*Smyd4-2*	SMYD	*Anopheles minimus*
AMIN001595	*Smyd4-3*	SMYD	*Anopheles minimus*
AMIN004343	*Smyd4-4*	SMYD	*Anopheles minimus*
AMIN006792	*Smyd4-4*	SMYD	*Anopheles minimus*
AMIN007057	*Smyd4-4*	SMYD	*Anopheles minimus*
AMIN008359	*Smyd4-4*	SMYD	*Anopheles minimus*
AMIN000845	*Smyd5*	SMYD	*Anopheles minimus*
AMIN004523	*SmydA-1*	SmydA	*Anopheles minimus*
AMIN005380	*SmydA-2*	SmydA	*Anopheles minimus*
AMIN009066	*SmydA-4*	SmydA	*Anopheles minimus*
AMIN006575	*SmydA-5*	SmydA	*Anopheles minimus*
AMIN006572	*SmydA-6*	SmydA	*Anopheles minimus*
AMIN002102	*SmydA-8*	SmydA	*Anopheles minimus*
AMIN000200	*Su(var)3-9*	Suv	*Anopheles minimus*
AMIN008387	*trr*	Trx	*Anopheles minimus*
AMIN003572	*trx*	Trx	*Anopheles minimus*
AMIN006931	*upSET(MLL5)*	-	*Anopheles minimus*
AQUA000404	*ash1*	Ash	*Anopheles quadriannulatus*
AQUA004699	*Blimp-1*	PRDM	*Anopheles quadriannulatus*
AQUA000686	*egg*	Suv	*Anopheles quadriannulatus*
AQUA008909	*Ez*	Ez	*Anopheles quadriannulatus*
AQUA000150	*G9a*	Suv	*Anopheles quadriannulatus*
AQUA003948	*Hmt4-20*	-	*Anopheles quadriannulatus*
AQUA004809	*Mes-4*	Ash	*Anopheles quadriannulatus*
AQUA004272	*Set1*	Trx	*Anopheles quadriannulatus*
AQUA010424	*Set2*	Ash	*Anopheles quadriannulatus*
AQUA011894	*Set8*	-	*Anopheles quadriannulatus*
AQUA011555	*Smyd3*	SMYD	*Anopheles quadriannulatus*
AQUA002276	*Smyd4-1*	SMYD	*Anopheles quadriannulatus*
AQUA000168	*Smyd4-2*	SMYD	*Anopheles quadriannulatus*
AQUA007881	*Smyd4-3*	SMYD	*Anopheles quadriannulatus*
AQUA009048	*Smyd4-4*	SMYD	*Anopheles quadriannulatus*
AQUA006091	*Smyd4-4*	SMYD	*Anopheles quadriannulatus*
AQUA006092	*Smyd4-4*	SMYD	*Anopheles quadriannulatus*
AQUA006117	*Smyd4-4*	SMYD	*Anopheles quadriannulatus*
AQUA007251	*Smyd4-4*	SMYD	*Anopheles quadriannulatus*
AQUA009729	*Smyd4-4*	SMYD	*Anopheles quadriannulatus*
AQUA011953	*Smyd4-4*	SMYD	*Anopheles quadriannulatus*

AQUA011962	*Smyd4-4*	SMYD	*Anopheles quadriannulatus*
AQUA011963	*Smyd4-4*	SMYD	*Anopheles quadriannulatus*
AQUA011967	*Smyd4-4*	SMYD	*Anopheles quadriannulatus*
AQUA001660	*Smyd5*	SMYD	*Anopheles quadriannulatus*
AQUA007380	*SmydA-1*	SmydA	*Anopheles quadriannulatus*
AQUA010849	*SmydA-2*	SmydA	*Anopheles quadriannulatus*
AQUA003637	*SmydA-3*	SmydA	*Anopheles quadriannulatus*
AQUA007386	*SmydA-4*	SmydA	*Anopheles quadriannulatus*
AQUA006709	*SmydA-5*	SmydA	*Anopheles quadriannulatus*
AQUA006712	*SmydA-6*	SmydA	*Anopheles quadriannulatus*
AQUA003949	*SmydA-8*	SmydA	*Anopheles quadriannulatus*
AQUA001617	*Su(var)3-9*	Suv	*Anopheles quadriannulatus*
AQUA005076	*trr*	Trx	*Anopheles quadriannulatus*
AQUA006747	*trx*	Trx	*Anopheles quadriannulatus*
AQUA002891	*upSET(MLL5)*	-	*Anopheles quadriannulatus*
ASIS001853	*ash1*	Ash	*Anopheles sinensis*
ASIS005657	*Blimp-1*	PRDM	*Anopheles sinensis*
ASIS000823	*egg*	Suv	*Anopheles sinensis*
ASIS004256	*Ez*	Ez	*Anopheles sinensis*
ASIS008043	*G9a*	Suv	*Anopheles sinensis*
ASIS002039	*Mes-4*	Ash	*Anopheles sinensis*
ASIS019895	*Set1*	Trx	*Anopheles sinensis*
ASIS014568	*Set8*	-	*Anopheles sinensis*
ASIS003741	*setd*	SETD	*Anopheles sinensis*
ASIS016123	*Smyd4-1*	SMYD	*Anopheles sinensis*
ASIS023491	*Smyd4-2*	SMYD	*Anopheles sinensis*
ASIS011359	*Smyd4-2*	SMYD	*Anopheles sinensis*
ASIS014752	*Smyd4-3*	SMYD	*Anopheles sinensis*
ASIS005116	*Smyd4-4*	SMYD	*Anopheles sinensis*
ASIS003292	*Smyd5*	SMYD	*Anopheles sinensis*
ASIS007463	*SmydA-1*	SmydA	*Anopheles sinensis*
ASIS008940	*SmydA-5*	SmydA	*Anopheles sinensis*
ASIS012008	*SmydA-8*	SmydA	*Anopheles sinensis*
ASIS023794	*trr*	Trx	*Anopheles sinensis*
ASIS003703	*trx*	Trx	*Anopheles sinensis*
ASTEI06781	-	-	*Anopheles stephensi*
ASTEI10920	-	-	*Anopheles stephensi*
ASTEI05450	*ash1*	Ash	*Anopheles stephensi*
ASTEI02700	*Blimp-1*	PRDM	*Anopheles stephensi*
ASTEI01744	*egg*	Suv	*Anopheles stephensi*
ASTEI11490	*Ez*	Ez	*Anopheles stephensi*
ASTEI03292	*G9a*	Suv	*Anopheles stephensi*
ASTEI01247	*Hmt4-20*	-	*Anopheles stephensi*
ASTEI06936	*Mes-4*	Ash	*Anopheles stephensi*

ASTEI03436	*Set1*	Trx	*Anopheles stephensi*
ASTEI08574	*Set2*	Ash	*Anopheles stephensi*
ASTEI06658	*Set8*	-	*Anopheles stephensi*
ASTEI00369	*setd*	SETD	*Anopheles stephensi*
ASTEI01947	*Smyd3*	SMYD	*Anopheles stephensi*
ASTEI01959	*Smyd4-1*	SMYD	*Anopheles stephensi*
ASTEI03277	*Smyd4-2*	SMYD	*Anopheles stephensi*
ASTEI05014	*Smyd4-3*	SMYD	*Anopheles stephensi*
ASTEI05013	*Smyd4-3*	SMYD	*Anopheles stephensi*
ASTEI05052	*Smyd4-4*	SMYD	*Anopheles stephensi*
ASTEI11197	*Smyd4-4*	SMYD	*Anopheles stephensi*
ASTEI04392	*Smyd5*	SMYD	*Anopheles stephensi*
ASTEI03856	*SmydA-1*	SmydA	*Anopheles stephensi*
ASTEI03536	*SmydA-2*	SmydA	*Anopheles stephensi*
ASTEI10159	*SmydA-4*	SmydA	*Anopheles stephensi*
ASTEI06743	*SmydA-5*	SmydA	*Anopheles stephensi*
ASTEI06740	*SmydA-6*	SmydA	*Anopheles stephensi*
ASTEI01245	*SmydA-8*	SmydA	*Anopheles stephensi*
ASTEI04355	*Su(var)3-9*	Suv	*Anopheles stephensi*
ASTEI06814	*trr*	Trx	*Anopheles stephensi*
ASTEI03164	*trx*	Trx	*Anopheles stephensi*
ASTEI02464	*upSET(MLL5)*	-	*Anopheles stephensi*
AGLA002233	-	-	*Anoplophora glabripennis*
AGLA006133	*ash1*	Ash	*Anoplophora glabripennis*
AGLA005605	*Blimp-1*	PRDM	*Anoplophora glabripennis*
AGLA021199	*Blimp-1*	PRDM	*Anoplophora glabripennis*
AGLA015578	*egg*	Suv	*Anoplophora glabripennis*
AGLA010746	*Ez*	Ez	*Anoplophora glabripennis*
AGLA007097	*Ez*	Ez	*Anoplophora glabripennis*
AGLA011365	*G9a*	Suv	*Anoplophora glabripennis*
AGLA005255	*Hmt4-20*	-	*Anoplophora glabripennis*
AGLA005108	*Mes-4*	Ash	*Anoplophora glabripennis*
AGLA012456	*Set1*	Trx	*Anoplophora glabripennis*
AGLA007361	*Set2*	Ash	*Anoplophora glabripennis*
AGLA013595	*Set8*	-	*Anoplophora glabripennis*
AGLA013594	*Set8*	-	*Anoplophora glabripennis*
AGLA000708	*setd*	SETD	*Anoplophora glabripennis*
AGLA005231	*Smyd3*	SMYD	*Anoplophora glabripennis*
AGLA012467	*Smyd4-1*	SMYD	*Anoplophora glabripennis*
AGLA008638	*Smyd4-2*	SMYD	*Anoplophora glabripennis*
AGLA021486	*Smyd4-2*	SMYD	*Anoplophora glabripennis*
AGLA000223	*Smyd4-3*	SMYD	*Anoplophora glabripennis*
AGLA005552	*Smyd4-4*	SMYD	*Anoplophora glabripennis*
AGLA021705	*Smyd5*	SMYD	*Anoplophora glabripennis*

AGLA001654	*SmydA-1*	SmydA	*Anoplophora glabripennis*
AGLA001655	*SmydA-4*	SmydA	*Anoplophora glabripennis*
AGLA001231	*SmydA-5*	SmydA	*Anoplophora glabripennis*
AGLA001385	*suv*	Suv	*Anoplophora glabripennis*
AGLA005070	*trr*	Trx	*Anoplophora glabripennis*
AGLA000269	*trx*	Trx	*Anoplophora glabripennis*
XP_006612780.1	*ash1*	Ash	*Apis dorsata*
XP_006621322.1	*Blimp-1*	PRDM	*Apis dorsata*
XP_006608998.1	*egg*	Suv	*Apis dorsata*
XP_006607899.1	*Ez*	Ez	*Apis dorsata*
XP_006614809.1	*G9a*	Suv	*Apis dorsata*
XP_006622888.1	*Hmt4-20*	-	*Apis dorsata*
XP_006607919.1	*Mes-4*	Ash	*Apis dorsata*
XP_006613374.1	*Set1*	Trx	*Apis dorsata*
XP_006608686.1	*Set2*	Ash	*Apis dorsata*
XP_006617682.1	*Set8*	-	*Apis dorsata*
XP_006610749.1	*Smyd4-1*	SMYD	*Apis dorsata*
XP_006622927.1	*Smyd4-2*	SMYD	*Apis dorsata*
XP_006622175.1	*Smyd4-3*	SMYD	*Apis dorsata*
XP_006622829.1	*Smyd4-3*	SMYD	*Apis dorsata*
XP_006619877.1	*Smyd4-4*	SMYD	*Apis dorsata*
XP_006608248.1	*Smyd5*	SMYD	*Apis dorsata*
XP_006615710.1	*SmydA-3*	SmydA	*Apis dorsata*
XP_006615709.1	*SmydA-4*	SmydA	*Apis dorsata*
XP_006618220.1	*SmydA-5*	SmydA	*Apis dorsata*
XP_006612041.1	*SmydA-8*	SmydA	*Apis dorsata*
XP_006620530.1	*Su(var)3-9*	Suv	*Apis dorsata*
XP_006609855.1	*upSET(MLL5)*	-	*Apis dorsata*
XP_012343597.1	*ash1*	Ash	*Apis florea*
XP_012346985.1	*Blimp-1*	PRDM	*Apis florea*
XP_012348733.1	*egg*	Suv	*Apis florea*
XP_003698603.1	*G9a*	Suv	*Apis florea*
XP_003696886.1	*Hmt4-20*	-	*Apis florea*
XP_012348612.1	*Mes-4*	Ash	*Apis florea*
XP_003690574.1	*Set1*	Trx	*Apis florea*
XP_012341488.1	*Set2*	Ash	*Apis florea*
XP_012344335.1	*Set8*	-	*Apis florea*
XP_003691619.1	*setd*	SETD	*Apis florea*
XP_012350926.1	*Smyd4-2*	SMYD	*Apis florea*
XP_012348137.1	*Smyd4-2*	SMYD	*Apis florea*
XP_003691021.1	*Smyd4-3*	SMYD	*Apis florea*
XP_012349534.1	*Smyd4-3*	SMYD	*Apis florea*
XP_003694793.1	*Smyd4-4*	SMYD	*Apis florea*
XP_012342066.1	*Smyd4-4*	SMYD	*Apis florea*

XP_003692172.1	*Smyd5*	SMYD	*Apis florea*
XP_003693098.2	*SmydA-1*	SmydA	*Apis florea*
XP_003695189.1	*SmydA-3*	SmydA	*Apis florea*
XP_003695192.1	*SmydA-4*	SmydA	*Apis florea*
XP_003697129.1	*SmydA-5*	SmydA	*Apis florea*
XP_003695246.1	*SmydA-8*	SmydA	*Apis florea*
XP_012347907.1	*Su(var)3-9*	Suv	*Apis florea*
XP_012347592.1	*trx*	Trx	*Apis florea*
XP_012342729.1	*upSET(MLL5)*	-	*Apis florea*
GB11034	-	-	*Apis mellifera*
GB12658	*ash1*	Ash	*Apis mellifera*
GB11750	*Blimp-1*	PRDM	*Apis mellifera*
GB17661	*egg*	Suv	*Apis mellifera*
GB15558	*Ez*	Ez	*Apis mellifera*
GB18236	*G9a*	Suv	*Apis mellifera*
GB19385	*Hmt4-20*	-	*Apis mellifera*
GB15744	*Set1*	Trx	*Apis mellifera*
GB10605	*Set2*	Ash	*Apis mellifera*
GB15534	*Set2*	Ash	*Apis mellifera*
GB15777	*Set8*	-	*Apis mellifera*
GB14294	*setd*	SETD	*Apis mellifera*
GB16596	*Smyd4-2*	SMYD	*Apis mellifera*
GB19743	*Smyd4-2*	SMYD	*Apis mellifera*
GB11612	*Smyd4-3*	SMYD	*Apis mellifera*
GB17686	*Smyd4-4*	SMYD	*Apis mellifera*
GB15540	*Smyd4-4*	SMYD	*Apis mellifera*
GB15657	*Smyd4-4*	SMYD	*Apis mellifera*
GB13751	*Smyd5*	SMYD	*Apis mellifera*
GB14592	*SmydA-1*	SmydA	*Apis mellifera*
GB12331	*SmydA-3*	SmydA	*Apis mellifera*
GB17689	*SmydA-4*	SmydA	*Apis mellifera*
GB10954	*SmydA-5*	SmydA	*Apis mellifera*
GB17649	*SmydA-8*	SmydA	*Apis mellifera*
GB30073	*Su(var)3-9*	Suv	*Apis mellifera*
GB13240	*suv*	Suv	*Apis mellifera*
GB19436	*trr*	Trx	*Apis mellifera*
GB16330	*trx*	Trx	*Apis mellifera*
GB16847	*upSET(MLL5)*	-	*Apis mellifera*
XP_012267274.1	-	-	*Athalia rosae*
XP_012250907.1	*Blimp-1*	PRDM	*Athalia rosae*
XP_012262309.1	*Blimp-1*	PRDM	*Athalia rosae*
XP_012269296.1	*Ez*	Ez	*Athalia rosae*
XP_012253973.1	*G9a*	Suv	*Athalia rosae*
XP_012264648.1	*Hmt4-20*	-	*Athalia rosae*

XP_012266705.1	*Mes-4*	Ash	*Athalia rosae*
XP_012266861.1	*Set1*	Trx	*Athalia rosae*
XP_012256884.1	*Set2*	Ash	*Athalia rosae*
XP_012254429.1	*Set8*	-	*Athalia rosae*
XP_012256557.1	*setd*	SETD	*Athalia rosae*
XP_012251505.1	*Smyd3*	SMYD	*Athalia rosae*
XP_012251654.1	*Smyd4-2*	SMYD	*Athalia rosae*
XP_012268663.1	*Smyd4-2*	SMYD	*Athalia rosae*
XP_012257200.1	*Smyd4-3*	SMYD	*Athalia rosae*
XP_012267297.1	*Smyd4-3*	SMYD	*Athalia rosae*
XP_012267599.1	*Smyd4-4*	SMYD	*Athalia rosae*
XP_012261874.1	*Smyd5*	SMYD	*Athalia rosae*
XP_012267502.1	*SmydA-1*	SmydA	*Athalia rosae*
XP_012268035.1	*SmydA-3*	SmydA	*Athalia rosae*
XP_012258552.1	*SmydA-5*	SmydA	*Athalia rosae*
XP_012268041.1	*SmydA-6*	SmydA	*Athalia rosae*
XP_012254696.1	*SmydA-8*	SmydA	*Athalia rosae*
XP_012258464.1	*Su(var)3-9*	Suv	*Athalia rosae*
XP_012251946.1	*suv*	Suv	*Athalia rosae*
XP_012260877.1	*trx*	Trx	*Athalia rosae*
ACEP_00014871	-	-	*Atta cephalotes*
ACEP_00015005	*ash1*	Ash	*Atta cephalotes*
ACEP_00011370	*Blimp-1*	PRDM	*Atta cephalotes*
ACEP_00010437	*egg*	Suv	*Atta cephalotes*
ACEP_00006353	*Ez*	Ez	*Atta cephalotes*
ACEP_00008842	*G9a*	Suv	*Atta cephalotes*
ACEP_00000745	*Hmt4-20*	-	*Atta cephalotes*
ACEP_00011229	*Mes-4*	Ash	*Atta cephalotes*
ACEP_00007263	*Set1*	Trx	*Atta cephalotes*
ACEP_00012429	*Set2*	Ash	*Atta cephalotes*
ACEP_00000026	*Set8*	-	*Atta cephalotes*
ACEP_00007464	*setd*	SETD	*Atta cephalotes*
ACEP_00008566	*Smyd3*	SMYD	*Atta cephalotes*
ACEP_00014186	*Smyd4-2*	SMYD	*Atta cephalotes*
ACEP_00013996	*Smyd4-2*	SMYD	*Atta cephalotes*
ACEP_00007748	*Smyd4-3*	SMYD	*Atta cephalotes*
ACEP_00007892	*Smyd4-3*	SMYD	*Atta cephalotes*
ACEP_00014643	*Smyd4-4*	SMYD	*Atta cephalotes*
ACEP_00015233	*Smyd4-4*	SMYD	*Atta cephalotes*
ACEP_00016065	*Smyd5*	SMYD	*Atta cephalotes*
ACEP_00002782	*SmydA-3*	SmydA	*Atta cephalotes*
ACEP_00010580	*SmydA-5*	SmydA	*Atta cephalotes*
ACEP_00006444	*SmydA-8*	SmydA	*Atta cephalotes*
ACEP_00014833	*Su(var)3-9*	Suv	*Atta cephalotes*

ACEP_00012166	*suv*	Suv	*Atta cephalotes*
ACEP_00016352	*trr*	Trx	*Atta cephalotes*
ACEP_00002886	*trx*	Trx	*Atta cephalotes*
ACEP_00011025	*upSET(MLL5)*	-	*Atta cephalotes*
XP_011188148.1	-	-	*Bactrocera cucurbitae*
XP_011176622.1	*ash1*	Ash	*Bactrocera cucurbitae*
XP_011195704.1	*Blimp-1*	PRDM	*Bactrocera cucurbitae*
XP_011196290.1	*egg*	Suv	*Bactrocera cucurbitae*
XP_011183791.1	*Ez*	Ez	*Bactrocera cucurbitae*
XP_011176759.1	*G9a*	Suv	*Bactrocera cucurbitae*
XP_011192804.1	*Mes-4*	Ash	*Bactrocera cucurbitae*
XP_011188172.1	*Set1*	Trx	*Bactrocera cucurbitae*
XP_011185204.1	*Set2*	Ash	*Bactrocera cucurbitae*
XP_011181958.1	*Set8*	-	*Bactrocera cucurbitae*
XP_011177109.1	*setd*	SETD	*Bactrocera cucurbitae*
XP_011178847.1	*Smyd4-1*	SMYD	*Bactrocera cucurbitae*
XP_011179663.1	*Smyd4-2*	SMYD	*Bactrocera cucurbitae*
XP_011192734.1	*Smyd4-3*	SMYD	*Bactrocera cucurbitae*
XP_011180406.1	*Smyd4-4*	SMYD	*Bactrocera cucurbitae*
XP_011179116.1	*Smyd5*	SMYD	*Bactrocera cucurbitae*
XP_011190850.1	*SmydA-1*	SmydA	*Bactrocera cucurbitae*
XP_011186389.1	*SmydA-2*	SmydA	*Bactrocera cucurbitae*
XP_011192453.1	*SmydA-3*	SmydA	*Bactrocera cucurbitae*
XP_011190535.1	*SmydA-4*	SmydA	*Bactrocera cucurbitae*
XP_011184464.1	*SmydA-5*	SmydA	*Bactrocera cucurbitae*
XP_011179434.1	*SmydA-6*	SmydA	*Bactrocera cucurbitae*
XP_011179432.1	*SmydA-7*	SmydA	*Bactrocera cucurbitae*
XP_011177534.1	*SmydA-8*	SmydA	*Bactrocera cucurbitae*
XP_011177535.1	*SmydA-8*	SmydA	*Bactrocera cucurbitae*
XP_011177548.1	*SmydA-9*	SmydA	*Bactrocera cucurbitae*
XP_011197020.1	*Su(var)3-9*	Suv	*Bactrocera cucurbitae*
XP_011194965.1	*suv*	Suv	*Bactrocera cucurbitae*
XP_011184577.1	*trr*	Trx	*Bactrocera cucurbitae*
XP_011191289.1	*trx*	Trx	*Bactrocera cucurbitae*
XP_011180383.1	*upSET(MLL5)*	-	*Bactrocera cucurbitae*
XP_011200269.1	-	-	*Bactrocera dorsalis*
XP_011200270.1	-	-	*Bactrocera dorsalis*
XP_011206237.1	-	-	*Bactrocera dorsalis*
XP_011208048.1	*ash1*	Ash	*Bactrocera dorsalis*
XP_011208049.1	*ash1*	Ash	*Bactrocera dorsalis*
XP_011214596.1	*Blimp-1*	PRDM	*Bactrocera dorsalis*
XP_011214597.1	*Blimp-1*	PRDM	*Bactrocera dorsalis*
XP_011202233.1	*egg*	Suv	*Bactrocera dorsalis*
XP_011212967.1	*Ez*	Ez	*Bactrocera dorsalis*

XP_011212966.1	*Ez*	Ez	*Bactrocera dorsalis*
XP_011211948.1	*G9a*	Suv	*Bactrocera dorsalis*
XP_011213500.1	*Hmt4-20*	-	*Bactrocera dorsalis*
XP_011213501.1	*Hmt4-20*	-	*Bactrocera dorsalis*
XP_011213502.1	*Hmt4-20*	-	*Bactrocera dorsalis*
XP_011213503.1	*Hmt4-20*	-	*Bactrocera dorsalis*
XP_011213504.1	*Hmt4-20*	-	*Bactrocera dorsalis*
XP_011213505.1	*Hmt4-20*	-	*Bactrocera dorsalis*
XP_011198612.1	*Mes-4*	Ash	*Bactrocera dorsalis*
XP_011200659.1	*Set1*	Trx	*Bactrocera dorsalis*
XP_011201871.1	*Set2*	Ash	*Bactrocera dorsalis*
XP_011208713.1	*Set8*	-	*Bactrocera dorsalis*
XP_011212148.1	*setd*	SETD	*Bactrocera dorsalis*
XP_011214100.1	*Smyd4-1*	SMYD	*Bactrocera dorsalis*
XP_011213895.1	*Smyd4-3*	SMYD	*Bactrocera dorsalis*
XP_011213000.1	*Smyd4-4*	SMYD	*Bactrocera dorsalis*
XP_011203140.1	*Smyd5*	SMYD	*Bactrocera dorsalis*
XP_011202123.1	*SmydA-1*	SmydA	*Bactrocera dorsalis*
XP_011199497.1	*SmydA-2*	SmydA	*Bactrocera dorsalis*
XP_011206159.1	*SmydA-3*	SmydA	*Bactrocera dorsalis*
XP_011206149.1	*SmydA-3*	SmydA	*Bactrocera dorsalis*
XP_011204895.1	*SmydA-4*	SmydA	*Bactrocera dorsalis*
XP_011206042.1	*SmydA-5*	SmydA	*Bactrocera dorsalis*
XP_011206043.1	*SmydA-5*	SmydA	*Bactrocera dorsalis*
XP_011204315.1	*SmydA-6*	SmydA	*Bactrocera dorsalis*
XP_011204314.1	*SmydA-7*	SmydA	*Bactrocera dorsalis*
XP_011207120.1	*SmydA-8*	SmydA	*Bactrocera dorsalis*
XP_011207121.1	*SmydA-9*	SmydA	*Bactrocera dorsalis*
XP_011203153.1	*Su(var)3-9*	Suv	*Bactrocera dorsalis*
XP_011203390.1	*suv*	Suv	*Bactrocera dorsalis*
XP_011206134.1	*trr*	Trx	*Bactrocera dorsalis*
XP_011206135.1	*trr*	Trx	*Bactrocera dorsalis*
XP_011206137.1	*trr*	Trx	*Bactrocera dorsalis*
XP_011206138.1	*trr*	Trx	*Bactrocera dorsalis*
XP_011199371.1	*trx*	Trx	*Bactrocera dorsalis*
XP_011200268.1	*upSET(MLL5)*	-	*Bactrocera dorsalis*
IU25_04690-mRNA-1	-	-	*Belgica antarctica*
IU25_10565-mRNA-1	-	-	*Belgica antarctica*
IU25_00528-mRNA-1	*ash1*	Ash	*Belgica antarctica*
IU25_08656-mRNA-1	*Blimp-1*	PRDM	*Belgica antarctica*
IU25_07936-mRNA-1	*egg*	Suv	*Belgica antarctica*
IU25_02089-mRNA-1	*G9a*	Suv	*Belgica antarctica*
IU25_05679-mRNA-1	*Hmt4-20*	-	*Belgica antarctica*
IU25_00891-mRNA-1	*Mes-4*	Ash	*Belgica antarctica*

IU25_05451-mRNA-1	*Set1*	Trx	*Belgica antarctica*
IU25_10271-mRNA-1	*Set1*	Trx	*Belgica antarctica*
IU25_10678-mRNA-1	*Set2*	Ash	*Belgica antarctica*
IU25_01859-mRNA-1	*Set8*	-	*Belgica antarctica*
IU25_12091-mRNA-1	*setd*	SETD	*Belgica antarctica*
IU25_11943-mRNA-1	*setd*	SETD	*Belgica antarctica*
IU25_00642-mRNA-1	*Smyd4-1*	SMYD	*Belgica antarctica*
IU25_08766-mRNA-1	*Smyd4-2*	SMYD	*Belgica antarctica*
IU25_08031-mRNA-1	*Smyd4-3*	SMYD	*Belgica antarctica*
IU25_03721-mRNA-1	*Smyd4-4*	SMYD	*Belgica antarctica*
IU25_00723-mRNA-1	*Smyd4-4*	SMYD	*Belgica antarctica*
IU25_00904-mRNA-1	*Smyd4-4*	SMYD	*Belgica antarctica*
IU25_01068-mRNA-1	*Smyd4-4*	SMYD	*Belgica antarctica*
IU25_01069-mRNA-1	*Smyd4-4*	SMYD	*Belgica antarctica*
IU25_01260-mRNA-1	*Smyd4-4*	SMYD	*Belgica antarctica*
IU25_01871-mRNA-1	*Smyd4-4*	SMYD	*Belgica antarctica*
IU25_03606-mRNA-1	*Smyd4-4*	SMYD	*Belgica antarctica*
IU25_05375-mRNA-1	*Smyd4-4*	SMYD	*Belgica antarctica*
IU25_06006-mRNA-1	*Smyd4-4*	SMYD	*Belgica antarctica*
IU25_06668-mRNA-1	*Smyd4-4*	SMYD	*Belgica antarctica*
IU25_07270-mRNA-1	*Smyd4-4*	SMYD	*Belgica antarctica*
IU25_07274-mRNA-1	*Smyd4-4*	SMYD	*Belgica antarctica*
IU25_08842-mRNA-1	*Smyd4-4*	SMYD	*Belgica antarctica*
IU25_10111-mRNA-1	*Smyd4-4*	SMYD	*Belgica antarctica*
IU25_10113-mRNA-1	*Smyd4-4*	SMYD	*Belgica antarctica*
IU25_10152-mRNA-1	*Smyd4-4*	SMYD	*Belgica antarctica*
IU25_11387-mRNA-1	*Smyd4-4*	SMYD	*Belgica antarctica*
IU25_11765-mRNA-1	*Smyd4-4*	SMYD	*Belgica antarctica*
IU25_12237-mRNA-1	*Smyd4-4*	SMYD	*Belgica antarctica*
IU25_12452-mRNA-1	*Smyd4-4*	SMYD	*Belgica antarctica*
IU25_12491-mRNA-1	*Smyd4-4*	SMYD	*Belgica antarctica*
IU25_12675-mRNA-1	*Smyd4-4*	SMYD	*Belgica antarctica*
IU25_10269-mRNA-1	*Smyd5*	SMYD	*Belgica antarctica*
IU25_09331-mRNA-1	*SmydA-1*	SmydA	*Belgica antarctica*
IU25_01008-mRNA-1	*SmydA-2*	SmydA	*Belgica antarctica*
IU25_07109-mRNA-1	*SmydA-3*	SmydA	*Belgica antarctica*
IU25_03751-mRNA-1	*SmydA-4*	SmydA	*Belgica antarctica*
IU25_02565-mRNA-1	*SmydA-4*	SmydA	*Belgica antarctica*
IU25_06201-mRNA-1	*SmydA-5*	SmydA	*Belgica antarctica*
IU25_12890-mRNA-1	*SmydA-5*	SmydA	*Belgica antarctica*
IU25_02010-mRNA-1	*SmydA-8*	SmydA	*Belgica antarctica*
IU25_06490-mRNA-1	*Su(var)3-9*	Suv	*Belgica antarctica*
IU25_01555-mRNA-1	*Su(var)3-9*	Suv	*Belgica antarctica*
IU25_09435-mRNA-1	*suv*	Suv	*Belgica antarctica*

IU25_11118-mRNA-1	*trr*	Trx	*Belgica antarctica*
IU25_03226-mRNA-1	*trx*	Trx	*Belgica antarctica*
BGER006344	*ash1*	Ash	*Blattella germanica*
BGER020182	*Blimp-1*	PRDM	*Blattella germanica*
BGER002073	*Blimp-1*	PRDM	*Blattella germanica*
BGER011023	*egg*	Suv	*Blattella germanica*
BGER007156	*G9a*	Suv	*Blattella germanica*
BGER002755	*Hmt4-20*	-	*Blattella germanica*
BGER017642	*Mes-4*	Ash	*Blattella germanica*
BGER027636	*Mes-4*	Ash	*Blattella germanica*
BGER015052	*Set2*	Ash	*Blattella germanica*
BGER016165	*Set8*	-	*Blattella germanica*
BGER022530	*setd*	SETD	*Blattella germanica*
BGER022529	*setd*	SETD	*Blattella germanica*
BGER011879	*Smyd4-2*	SMYD	*Blattella germanica*
BGER015689	*Smyd4-2*	SMYD	*Blattella germanica*
BGER005639	*Smyd4-2*	SMYD	*Blattella germanica*
BGER011191	*Smyd5*	SMYD	*Blattella germanica*
BGER004943	*SmydA-1*	SmydA	*Blattella germanica*
BGER006802	*SmydA-2*	SmydA	*Blattella germanica*
BGER004944	*SmydA-4*	SmydA	*Blattella germanica*
BGER008636	*SmydA-8*	SmydA	*Blattella germanica*
BGER028006	*Su(var)3-9*	Suv	*Blattella germanica*
BGER005717	*trr*	Trx	*Blattella germanica*
BGER025951	*trx*	Trx	*Blattella germanica*
BGER019382	*upSET(MLL5)*	-	*Blattella germanica*
Bimp04847	-	-	*Bombus impatiens*
Bimp12520	-	-	*Bombus impatiens*
Bimp11516	*ash1*	Ash	*Bombus impatiens*
Bimp08899	*Blimp-1*	PRDM	*Bombus impatiens*
Bimp02612	*egg*	Suv	*Bombus impatiens*
Bimp01437	*Ez*	Ez	*Bombus impatiens*
Bimp06854	*G9a*	Suv	*Bombus impatiens*
Bimp12779	*Hmt4-20*	-	*Bombus impatiens*
Bimp13077	*Mes-4*	Ash	*Bombus impatiens*
Bimp12329	*Set1*	Trx	*Bombus impatiens*
Bimp09037	*Set2*	Ash	*Bombus impatiens*
Bimp03899	*Set8*	-	*Bombus impatiens*
Bimp04211	*setd*	SETD	*Bombus impatiens*
Bimp08276	*Smyd4-2*	SMYD	*Bombus impatiens*
Bimp03415	*Smyd4-3*	SMYD	*Bombus impatiens*
Bimp08047	*Smyd4-3*	SMYD	*Bombus impatiens*
Bimp13566	*Smyd4-3*	SMYD	*Bombus impatiens*
Bimp10275	*Smyd4-4*	SMYD	*Bombus impatiens*

Bimp02797	*Smyd4-4*	SMYD	*Bombus impatiens*
Bimp02745	*Smyd5*	SMYD	*Bombus impatiens*
Bimp14104	*SmydA-1*	SmydA	*Bombus impatiens*
Bimp02590	*SmydA-3*	SmydA	*Bombus impatiens*
Bimp12013	*SmydA-4*	SmydA	*Bombus impatiens*
Bimp12651	*SmydA-5*	SmydA	*Bombus impatiens*
Bimp09996	*SmydA-8*	SmydA	*Bombus impatiens*
Bimp05474	*Su(var)3-9*	Suv	*Bombus impatiens*
Bimp10158	*suv*	Suv	*Bombus impatiens*
Bimp12146	*trr*	Trx	*Bombus impatiens*
Bimp05951	*trx*	Trx	*Bombus impatiens*
Bimp09131	*upSET(MLL5)*	-	*Bombus impatiens*
Bter05546	-	-	*Bombus terrestris*
Bter07245	*ash1*	Ash	*Bombus terrestris*
Bter05279	*Blimp-1*	PRDM	*Bombus terrestris*
Bter06631	*egg*	Suv	*Bombus terrestris*
Bter04454	*Ez*	Ez	*Bombus terrestris*
Bter01253	*G9a*	Suv	*Bombus terrestris*
Bter09373	*Hmt4-20*	-	*Bombus terrestris*
Bter10757	*Mes-4*	Ash	*Bombus terrestris*
Bter10939	*Set1*	Trx	*Bombus terrestris*
Bter08592	*Set2*	Ash	*Bombus terrestris*
Bter04090	*Set8*	-	*Bombus terrestris*
Bter05137	*setd*	SETD	*Bombus terrestris*
Bter00208	*Smyd4-2*	SMYD	*Bombus terrestris*
Bter09116	*Smyd4-2*	SMYD	*Bombus terrestris*
Bter01460	*Smyd4-3*	SMYD	*Bombus terrestris*
Bter05601	*Smyd4-3*	SMYD	*Bombus terrestris*
Bter06829	*Smyd4-3*	SMYD	*Bombus terrestris*
Bter07499	*Smyd4-4*	SMYD	*Bombus terrestris*
Bter03698	*Smyd4-4*	SMYD	*Bombus terrestris*
Bter07912	*Smyd5*	SMYD	*Bombus terrestris*
Bter06604	*SmydA-3*	SmydA	*Bombus terrestris*
Bter06605	*SmydA-4*	SmydA	*Bombus terrestris*
Bter00806	*SmydA-5*	SmydA	*Bombus terrestris*
Bter12980	*SmydA-8*	SmydA	*Bombus terrestris*
Bter08904	*Su(var)3-9*	Suv	*Bombus terrestris*
Bter08526	*suv*	Suv	*Bombus terrestris*
Bter11235	*trr*	Trx	*Bombus terrestris*
Bter00752	*trx*	Trx	*Bombus terrestris*
Bter08547	*upSET(MLL5)*	-	*Bombus terrestris*
BGIBMGA001497	*ash1*	Ash	*Bombyx mori*
BGIBMGA000319	*Blimp-1*	PRDM	*Bombyx mori*
BGIBMGA011407	*egg*	Suv	*Bombyx mori*

BGIBMGA014476	*Ez*	Ez	*Bombyx mori*
BGIBMGA007949	*G9a*	Suv	*Bombyx mori*
BGIBMGA000919	*Hmt4-20*	-	*Bombyx mori*
BGIBMGA002246	*Mes-4*	Ash	*Bombyx mori*
BGIBMGA012978	*Set1*	Trx	*Bombyx mori*
BGIBMGA003106	*Set2*	Ash	*Bombyx mori*
BGIBMGA012853	*Set8*	-	*Bombyx mori*
BGIBMGA008518	*setd*	SETD	*Bombyx mori*
BGIBMGA008517	*setd*	SETD	*Bombyx mori*
BGIBMGA002076	*Smyd4-2*	SMYD	*Bombyx mori*
BGIBMGA007907	*Smyd4-3*	SMYD	*Bombyx mori*
BGIBMGA014048	*Smyd4-4*	SMYD	*Bombyx mori*
BGIBMGA011073	*Smyd5*	SMYD	*Bombyx mori*
BGIBMGA008839	*SmydA-1*	SmydA	*Bombyx mori*
BGIBMGA008939	*SmydA-2*	SmydA	*Bombyx mori*
BGIBMGA008923	*SmydA-3*	SmydA	*Bombyx mori*
BGIBMGA008838	*SmydA-4*	SmydA	*Bombyx mori*
BGIBMGA003139	*SmydA-8*	SmydA	*Bombyx mori*
BGIBMGA011500	*Su(var)3-9*	Suv	*Bombyx mori*
BGIBMGA007557	*suv*	Suv	*Bombyx mori*
BGIBMGA002503	*trr*	Trx	*Bombyx mori*
BGIBMGA010221	*trx*	Trx	*Bombyx mori*
BGIBMGA004310	*upSET(MLL5)*	-	*Bombyx mori*
Cflo_06609	-	-	*Camponotus floridanus*
Cflo_10932	*ash1*	Ash	*Camponotus floridanus*
Cflo_04377	*Blimp-1*	PRDM	*Camponotus floridanus*
Cflo_09617	*egg*	Suv	*Camponotus floridanus*
Cflo_10335	*Ez*	Ez	*Camponotus floridanus*
Cflo_12188	*G9a*	Suv	*Camponotus floridanus*
Cflo_05373	*Hmt4-20*	-	*Camponotus floridanus*
Cflo_01290	*Mes-4*	Ash	*Camponotus floridanus*
Cflo_12528	*Set1*	Trx	*Camponotus floridanus*
Cflo_04573	*Set8*	-	*Camponotus floridanus*
Cflo_09803	*setd*	SETD	*Camponotus floridanus*
Cflo_09801	*setd*	SETD	*Camponotus floridanus*
Cflo_08673	*Smyd3*	SMYD	*Camponotus floridanus*
Cflo_06032	*Smyd4-2*	SMYD	*Camponotus floridanus*
Cflo_06938	*Smyd4-2*	SMYD	*Camponotus floridanus*
Cflo_12246	*Smyd4-2*	SMYD	*Camponotus floridanus*
Cflo_09007	*Smyd4-3*	SMYD	*Camponotus floridanus*
Cflo_03939	*Smyd4-4*	SMYD	*Camponotus floridanus*
Cflo_15030	*Smyd4-4*	SMYD	*Camponotus floridanus*
Cflo_05356	*Smyd5*	SMYD	*Camponotus floridanus*
Cflo_08953	*SmydA-3*	SmydA	*Camponotus floridanus*

Cflo_10149	*SmydA-5*	SmydA	*Camponotus floridanus*
Cflo_08954	*SmydA-6*	SmydA	*Camponotus floridanus*
Cflo_06803	*SmydA-8*	SmydA	*Camponotus floridanus*
Cflo_12501	*suv*	Suv	*Camponotus floridanus*
Cflo_01543	*trx*	Trx	*Camponotus floridanus*
Cflo_12296	*upSET(MLL5)*	-	*Camponotus floridanus*
Cobs_02028	-	-	*Cardiocondyla obscurior*
Cobs_13062	-	-	*Cardiocondyla obscurior*
Cobs_02243	*ash1*	Ash	*Cardiocondyla obscurior*
Cobs_03004	*Blimp-1*	PRDM	*Cardiocondyla obscurior*
Cobs_06230	*egg*	Suv	*Cardiocondyla obscurior*
Cobs_09623	*Ez*	Ez	*Cardiocondyla obscurior*
Cobs_07320	*G9a*	Suv	*Cardiocondyla obscurior*
Cobs_11998	*Hmt4-20*	-	*Cardiocondyla obscurior*
Cobs_15648	*Mes-4*	Ash	*Cardiocondyla obscurior*
Cobs_03353	*Set1*	Trx	*Cardiocondyla obscurior*
Cobs_15746	*Set2*	Ash	*Cardiocondyla obscurior*
Cobs_12217	*Set8*	-	*Cardiocondyla obscurior*
Cobs_12222	*Set8*	-	*Cardiocondyla obscurior*
Cobs_00379	*setd*	SETD	*Cardiocondyla obscurior*
Cobs_00400	*setd*	SETD	*Cardiocondyla obscurior*
Cobs_11117	*Smyd4-2*	SMYD	*Cardiocondyla obscurior*
Cobs_09466	*Smyd4-2*	SMYD	*Cardiocondyla obscurior*
Cobs_03894	*Smyd4-3*	SMYD	*Cardiocondyla obscurior*
Cobs_03955	*Smyd4-3*	SMYD	*Cardiocondyla obscurior*
Cobs_13160	*Smyd4-4*	SMYD	*Cardiocondyla obscurior*
Cobs_04265	*Smyd4-4*	SMYD	*Cardiocondyla obscurior*
Cobs_02856	*Smyd5*	SMYD	*Cardiocondyla obscurior*
Cobs_05676	*SmydA-3*	SmydA	*Cardiocondyla obscurior*
Cobs_14589	*SmydA-4*	SmydA	*Cardiocondyla obscurior*
Cobs_06124	*SmydA-5*	SmydA	*Cardiocondyla obscurior*
Cobs_06906	*SmydA-8*	SmydA	*Cardiocondyla obscurior*
Cobs_08762	*Su(var)3-9*	Suv	*Cardiocondyla obscurior*
Cobs_05777	*suv*	Suv	*Cardiocondyla obscurior*
Cobs_17490	*trr*	Trx	*Cardiocondyla obscurior*
Cobs_02821	*trx*	Trx	*Cardiocondyla obscurior*
Cobs_01120	*upSET(MLL5)*	-	*Cardiocondyla obscurior*
CAQU000974	-	-	*Catajapyx aquilonaris*
CAQU003948	-	-	*Catajapyx aquilonaris*
CAQU008317	*ash1*	Ash	*Catajapyx aquilonaris*
CAQU006987	*Blimp-1*	PRDM	*Catajapyx aquilonaris*
CAQU009584	*egg*	Suv	*Catajapyx aquilonaris*
CAQU006575	*egg*	Suv	*Catajapyx aquilonaris*
CAQU008672	*egg*	Suv	*Catajapyx aquilonaris*

CAQU009251	*egg*	Suv	*Catajapyx aquilonaris*
CAQU010052	*egg*	Suv	*Catajapyx aquilonaris*
CAQU003673	*Ez*	Ez	*Catajapyx aquilonaris*
CAQU006666	*G9a*	Suv	*Catajapyx aquilonaris*
CAQU004348	*Hmt4-20*	-	*Catajapyx aquilonaris*
CAQU003309	*Mes-4*	Ash	*Catajapyx aquilonaris*
CAQU006724	*Mes-4*	Ash	*Catajapyx aquilonaris*
CAQU007463	*Mes-4*	Ash	*Catajapyx aquilonaris*
CAQU005130	*Set1*	Trx	*Catajapyx aquilonaris*
CAQU002743	*Set2*	Ash	*Catajapyx aquilonaris*
CAQU004916	*Set8*	-	*Catajapyx aquilonaris*
CAQU001133	*setd*	SETD	*Catajapyx aquilonaris*
CAQU002086	*Smyd3*	SMYD	*Catajapyx aquilonaris*
CAQU002583	*Smyd4-2*	SMYD	*Catajapyx aquilonaris*
CAQU000020	*Smyd4-3*	SMYD	*Catajapyx aquilonaris*
CAQU007067	*Smyd4-3*	SMYD	*Catajapyx aquilonaris*
CAQU007929	*Smyd4-3*	SMYD	*Catajapyx aquilonaris*
CAQU008111	*SmydA-2*	SmydA	*Catajapyx aquilonaris*
CAQU002468	*SmydA-2*	SmydA	*Catajapyx aquilonaris*
CAQU006587	*SmydA-3*	SmydA	*Catajapyx aquilonaris*
CAQU000051	*Su(var)3-9*	Suv	*Catajapyx aquilonaris*
CAQU008933	*suv*	Suv	*Catajapyx aquilonaris*
CAQU009228	*upSET(MLL5)*	-	*Catajapyx aquilonaris*
CSCU016626	*ash1*	Ash	*Centruroides exilicauda*
CSCU017888	*Blimp-1*	PRDM	*Centruroides exilicauda*
CSCU012540	*Blimp-1*	PRDM	*Centruroides exilicauda*
CSCU027036	*Blimp-1*	PRDM	*Centruroides exilicauda*
CSCU019674	*egg*	Suv	*Centruroides exilicauda*
CSCU027935	*egg*	Suv	*Centruroides exilicauda*
CSCU003019	*Ez*	Ez	*Centruroides exilicauda*
CSCU010330	*G9a*	Suv	*Centruroides exilicauda*
CSCU006684	*G9a*	Suv	*Centruroides exilicauda*
CSCU021696	*Hmt4-20*	-	*Centruroides exilicauda*
CSCU016417	*Mes-4*	Ash	*Centruroides exilicauda*
CSCU007994	*Set1*	Trx	*Centruroides exilicauda*
CSCU003748	*Set2*	Ash	*Centruroides exilicauda*
CSCU023248	*setd*	SETD	*Centruroides exilicauda*
CSCU027284	*Smyd4-3*	SMYD	*Centruroides exilicauda*
CSCU004980	*Smyd5*	SMYD	*Centruroides exilicauda*
CSCU028343	*Su(var)3-9*	Suv	*Centruroides exilicauda*
CSCU009512	*suv*	Suv	*Centruroides exilicauda*
CSCU015241	*trr*	Trx	*Centruroides exilicauda*
CSCU016579	*trx*	Trx	*Centruroides exilicauda*
CSCU000650	*upSET(MLL5)*	-	*Centruroides exilicauda*

XP_015591864.1	*Blimp-1*	PRDM	*Cephus cinctus*
XP_015588809.1	*egg*	Suv	*Cephus cinctus*
XP_015608564.1	*G9a*	Suv	*Cephus cinctus*
XP_015593020.1	*Set1*	Trx	*Cephus cinctus*
XP_015606617.1	*Set8*	-	*Cephus cinctus*
XP_015587046.1	*setd*	SETD	*Cephus cinctus*
XP_015595002.1	*Smyd4-1*	SMYD	*Cephus cinctus*
XP_015585699.1	*Smyd4-2*	SMYD	*Cephus cinctus*
XP_015599275.1	*Smyd4-3*	SMYD	*Cephus cinctus*
XP_015598490.1	*Smyd4-3*	SMYD	*Cephus cinctus*
XP_015610301.1	*Smyd4-4*	SMYD	*Cephus cinctus*
XP_015597696.1	*Smyd5*	SMYD	*Cephus cinctus*
XP_015588255.1	*SmydA-3*	SmydA	*Cephus cinctus*
XP_015588254.1	*SmydA-4*	SmydA	*Cephus cinctus*
XP_015595619.1	*SmydA-5*	SmydA	*Cephus cinctus*
XP_015607741.1	*SmydA-8*	SmydA	*Cephus cinctus*
XP_015585211.1	*Su(var)3-9*	Suv	*Cephus cinctus*
XP_015609433.1	*trx*	Trx	*Cephus cinctus*
XP_015601121.1	*upSET(MLL5)*	-	*Cephus cinctus*
XP_011351441.1	*Blimp-1*	PRDM	*Cerapachys biroi*
XP_011347842.1	*Ez*	Ez	*Cerapachys biroi*
XP_011347415.1	*G9a*	Suv	*Cerapachys biroi*
XP_011344661.1	*Set1*	Trx	*Cerapachys biroi*
XP_011341269.1	*Set8*	-	*Cerapachys biroi*
XP_011338544.1	*setd*	SETD	*Cerapachys biroi*
XP_011338883.1	*Smyd4-3*	SMYD	*Cerapachys biroi*
XP_011351984.1	*Smyd4-3*	SMYD	*Cerapachys biroi*
XP_011347284.1	*Smyd4-4*	SMYD	*Cerapachys biroi*
XP_011342209.1	*Smyd4-4*	SMYD	*Cerapachys biroi*
XP_011329713.1	*Smyd5*	SMYD	*Cerapachys biroi*
XP_011333272.1	*SmydA-3*	SmydA	*Cerapachys biroi*
XP_011333274.1	*SmydA-4*	SmydA	*Cerapachys biroi*
XP_011333700.1	*SmydA-5*	SmydA	*Cerapachys biroi*
XP_011336875.1	*SmydA-8*	SmydA	*Cerapachys biroi*
XP_011336738.1	*Su(var)3-9*	Suv	*Cerapachys biroi*
XP_011339538.1	*suv*	Suv	*Cerapachys biroi*
XP_011331544.1	*trr*	Trx	*Cerapachys biroi*
XP_011333299.1	*trx*	Trx	*Cerapachys biroi*
XP_011332728.1	*upSET(MLL5)*	-	*Cerapachys biroi*
XP_012157287.1	-	-	*Ceratina calcarata*
XP_004537513.1	*egg*	Suv	*Ceratina calcarata*
XP_004523769.1	*Ez*	Ez	*Ceratina calcarata*
XP_012156247.1	*G9a*	Suv	*Ceratina calcarata*
XP_004530722.1	*Mes-4*	Ash	*Ceratina calcarata*

XP_004524249.1	*Set1*	Trx	*Ceratina calcarata*
XP_004536421.1	*Set2*	Ash	*Ceratina calcarata*
XP_004520416.1	*setd*	SETD	*Ceratina calcarata*
XP_012160266.1	*Smyd4-1*	SMYD	*Ceratina calcarata*
XP_004525269.1	*Smyd4-2*	SMYD	*Ceratina calcarata*
XP_004535900.2	*Smyd4-3*	SMYD	*Ceratina calcarata*
XP_004529963.1	*Smyd4-4*	SMYD	*Ceratina calcarata*
XP_004519541.1	*Smyd5*	SMYD	*Ceratina calcarata*
XP_004526552.1	*SmydA-1*	SmydA	*Ceratina calcarata*
XP_004530579.1	*SmydA-2*	SmydA	*Ceratina calcarata*
XP_004524764.1	*SmydA-3*	SmydA	*Ceratina calcarata*
XP_004535903.2	*SmydA-4*	SmydA	*Ceratina calcarata*
XP_004534872.1	*SmydA-5*	SmydA	*Ceratina calcarata*
XP_004517861.1	*SmydA-6*	SmydA	*Ceratina calcarata*
XP_004517860.1	*SmydA-7*	SmydA	*Ceratina calcarata*
XP_004529471.1	*SmydA-8*	SmydA	*Ceratina calcarata*
XP_012158904.1	*SmydA-8*	SmydA	*Ceratina calcarata*
XP_004529472.1	*SmydA-9*	SmydA	*Ceratina calcarata*
XP_004537028.1	*Su(var)3-9*	Suv	*Ceratina calcarata*
XP_004524323.1	*suv*	Suv	*Ceratina calcarata*
XP_004534758.1	*trr*	Trx	*Ceratina calcarata*
XP_004517984.2	*trx*	Trx	*Ceratina calcarata*
CCAP005187	-	-	*Ceratitis capitata*
CCAP000782	*ash1*	Ash	*Ceratitis capitata*
CCAP012803	*Blimp-1*	PRDM	*Ceratitis capitata*
CCAP003621	*egg*	Suv	*Ceratitis capitata*
CCAP012789	*Ez*	Ez	*Ceratitis capitata*
CCAP013011	*G9a*	Suv	*Ceratitis capitata*
CCAP004663	*Hmt4-20*	-	*Ceratitis capitata*
CCAP008153	*Mes-4*	Ash	*Ceratitis capitata*
CCAP014583	*Set1*	Trx	*Ceratitis capitata*
CCAP004601	*Set2*	Ash	*Ceratitis capitata*
CCAP007602	*Set8*	-	*Ceratitis capitata*
CCAP014162	*setd*	SETD	*Ceratitis capitata*
CCAP010479	*Smyd4-1*	SMYD	*Ceratitis capitata*
CCAP000083	*Smyd4-2*	SMYD	*Ceratitis capitata*
CCAP007274	*Smyd4-3*	SMYD	*Ceratitis capitata*
CCAP007526	*Smyd4-4*	SMYD	*Ceratitis capitata*
CCAP003276	*Smyd5*	SMYD	*Ceratitis capitata*
CCAP006624	*SmydA-1*	SmydA	*Ceratitis capitata*
CCAP011991	*SmydA-2*	SmydA	*Ceratitis capitata*
CCAP006471	*SmydA-3*	SmydA	*Ceratitis capitata*
CCAP006469	*SmydA-3*	SmydA	*Ceratitis capitata*
CCAP007277	*SmydA-4*	SmydA	*Ceratitis capitata*

CCAP008432	*SmydA-5*	SmydA	*Ceratitis capitata*
CCAP002915	*SmydA-7*	SmydA	*Ceratitis capitata*
CCAP001315	*SmydA-8*	SmydA	*Ceratitis capitata*
CCAP001316	*SmydA-8*	SmydA	*Ceratitis capitata*
CCAP001317	*SmydA-9*	SmydA	*Ceratitis capitata*
CCAP004182	*suv*	Suv	*Ceratitis capitata*
CCAP008476	*trr*	Trx	*Ceratitis capitata*
CCAP001677	*trx*	Trx	*Ceratitis capitata*
CCAP000593	*upSET(MLL5)*	-	*Ceratitis capitata*
XP_011502952.1	-	-	*Ceratosolen solmsi*
XP_011502301.1	*ash1*	Ash	*Ceratosolen solmsi*
XP_011498682.1	*Blimp-1*	PRDM	*Ceratosolen solmsi*
XP_011495749.1	*egg*	Suv	*Ceratosolen solmsi*
XP_011505015.1	*Ez*	Ez	*Ceratosolen solmsi*
XP_011498291.1	*Hmt4-20*	-	*Ceratosolen solmsi*
XP_011503024.1	*Mes-4*	Ash	*Ceratosolen solmsi*
XP_011500896.1	*Set1*	Trx	*Ceratosolen solmsi*
XP_011506148.1	*Set2*	Ash	*Ceratosolen solmsi*
XP_011506141.1	*Set8*	-	*Ceratosolen solmsi*
XP_011493824.1	*Set8*	-	*Ceratosolen solmsi*
XP_011503823.1	*setd*	SETD	*Ceratosolen solmsi*
XP_011497960.1	*Smyd4-2*	SMYD	*Ceratosolen solmsi*
XP_011498185.1	*Smyd4-3*	SMYD	*Ceratosolen solmsi*
XP_011502289.1	*Smyd4-3*	SMYD	*Ceratosolen solmsi*
XP_011502335.1	*Smyd4-3*	SMYD	*Ceratosolen solmsi*
XP_011504410.1	*Smyd4-3*	SMYD	*Ceratosolen solmsi*
XP_011493877.1	*Smyd4-4*	SMYD	*Ceratosolen solmsi*
XP_011502672.1	*Smyd4-4*	SMYD	*Ceratosolen solmsi*
XP_011496156.1	*Smyd5*	SMYD	*Ceratosolen solmsi*
XP_011500086.1	*SmydA-1*	SmydA	*Ceratosolen solmsi*
XP_011506599.1	*SmydA-3*	SmydA	*Ceratosolen solmsi*
XP_011494674.1	*SmydA-5*	SmydA	*Ceratosolen solmsi*
XP_011506121.1	*SmydA-8*	SmydA	*Ceratosolen solmsi*
XP_011502402.1	*Su(var)3-9*	Suv	*Ceratosolen solmsi*
XP_011495370.1	*suv*	Suv	*Ceratosolen solmsi*
XP_011498084.1	*trr*	Trx	*Ceratosolen solmsi*
XP_011495095.1	*upSET(MLL5)*	-	*Ceratosolen solmsi*
CLEC001585	*ash1*	Ash	*Cimex lectularius*
CLEC009125	*Blimp-1*	PRDM	*Cimex lectularius*
CLEC004308	*Blimp-1*	PRDM	*Cimex lectularius*
CLEC011550	*egg*	Suv	*Cimex lectularius*
CLEC007642	*egg*	Suv	*Cimex lectularius*
CLEC007569	*Ez*	Ez	*Cimex lectularius*
CLEC007326	*G9a*	Suv	*Cimex lectularius*

CLEC009946	*Hmt4-20*	-	*Cimex lectularius*
CLEC000702	*Mes-4*	Ash	*Cimex lectularius*
CLEC007543	*Set1*	Trx	*Cimex lectularius*
CLEC005584	*Set2*	Ash	*Cimex lectularius*
CLEC003862	*Set8*	-	*Cimex lectularius*
CLEC011375	*setd*	SETD	*Cimex lectularius*
CLEC012489	*Smyd4-2*	SMYD	*Cimex lectularius*
CLEC007359	*Smyd4-3*	SMYD	*Cimex lectularius*
CLEC006827	*Smyd4-3*	SMYD	*Cimex lectularius*
CLEC002426	*Smyd5*	SMYD	*Cimex lectularius*
CLEC002375	*SmydA-1*	SmydA	*Cimex lectularius*
CLEC006315	*SmydA-5*	SmydA	*Cimex lectularius*
CLEC002827	*Su(var)3-9*	Suv	*Cimex lectularius*
CLEC013338	*suv*	Suv	*Cimex lectularius*
CLEC009783	*trr*	Trx	*Cimex lectularius*
CLEC009544	*trx*	Trx	*Cimex lectularius*
CLEC008463	*upSET(MLL5)*	-	*Cimex lectularius*
CFLO002450	-	-	*Copidosoma floridanum*
CFLO006696	-	-	*Copidosoma floridanum*
CFLO013435	-	-	*Copidosoma floridanum*
CFLO014028	-	-	*Copidosoma floridanum*
CFLO014497	-	-	*Copidosoma floridanum*
CFLO016633	-	-	*Copidosoma floridanum*
CFLO019278	-	-	*Copidosoma floridanum*
CFLO013589	-	-	*Copidosoma floridanum*
CFLO018848	*ash1*	Ash	*Copidosoma floridanum*
CFLO011457	*Blimp-1*	PRDM	*Copidosoma floridanum*
CFLO000021	*egg*	Suv	*Copidosoma floridanum*
CFLO001773	*Ez*	Ez	*Copidosoma floridanum*
CFLO004788	*G9a*	Suv	*Copidosoma floridanum*
CFLO002689	*Hmt4-20*	-	*Copidosoma floridanum*
CFLO003741	*Hmt4-20*	-	*Copidosoma floridanum*
CFLO013278	*Mes-4*	Ash	*Copidosoma floridanum*
CFLO004334	*Set1*	Trx	*Copidosoma floridanum*
CFLO010558	*Set2*	Ash	*Copidosoma floridanum*
CFLO018760	*Set8*	-	*Copidosoma floridanum*
CFLO018761	*Set8*	-	*Copidosoma floridanum*
CFLO018087	*setd*	SETD	*Copidosoma floridanum*
CFLO014410	*Smyd3*	SMYD	*Copidosoma floridanum*
CFLO011986	*Smyd4-2*	SMYD	*Copidosoma floridanum*
CFLO012996	*Smyd4-2*	SMYD	*Copidosoma floridanum*
CFLO012031	*Smyd4-3*	SMYD	*Copidosoma floridanum*
CFLO015820	*Smyd4-3*	SMYD	*Copidosoma floridanum*
CFLO019277	*Smyd4-3*	SMYD	*Copidosoma floridanum*

CFLO000957	*Smyd4-4*	SMYD	*Copidosoma floridanum*
CFLO000342	*Smyd4-4*	SMYD	*Copidosoma floridanum*
CFLO003138	*Smyd4-4*	SMYD	*Copidosoma floridanum*
CFLO003687	*Smyd4-4*	SMYD	*Copidosoma floridanum*
CFLO005082	*Smyd4-4*	SMYD	*Copidosoma floridanum*
CFLO009065	*Smyd4-4*	SMYD	*Copidosoma floridanum*
CFLO012998	*Smyd4-4*	SMYD	*Copidosoma floridanum*
CFLO016574	*Smyd4-4*	SMYD	*Copidosoma floridanum*
CFLO016948	*Smyd4-4*	SMYD	*Copidosoma floridanum*
CFLO019783	*Smyd4-4*	SMYD	*Copidosoma floridanum*
CFLO015317	*Smyd5*	SMYD	*Copidosoma floridanum*
CFLO006650	*SmydA-1*	SmydA	*Copidosoma floridanum*
CFLO004895	*SmydA-3*	SmydA	*Copidosoma floridanum*
CFLO007813	*SmydA-5*	SmydA	*Copidosoma floridanum*
CFLO004833	*SmydA-8*	SmydA	*Copidosoma floridanum*
CFLO018747	*Su(var)3-9*	Suv	*Copidosoma floridanum*
CFLO007195	*suv*	Suv	*Copidosoma floridanum*
CFLO003342	*trr*	Trx	*Copidosoma floridanum*
CPIJ000813	-	-	*Culex quinquefasciatus*
CPIJ000814	-	-	*Culex quinquefasciatus*
CPIJ010019	-	-	*Culex quinquefasciatus*
CPIJ006423	-	-	*Culex quinquefasciatus*
CPIJ007342	-	-	*Culex quinquefasciatus*
CPIJ001202	*ash1*	Ash	*Culex quinquefasciatus*
CPIJ007425	*Blimp-1*	PRDM	*Culex quinquefasciatus*
CPIJ016652	*egg*	Suv	*Culex quinquefasciatus*
CPIJ006484	*Ez*	Ez	*Culex quinquefasciatus*
CPIJ018501	*Hmt4-20*	-	*Culex quinquefasciatus*
CPIJ011088	*Mes-4*	Ash	*Culex quinquefasciatus*
CPIJ004932	*Mes-4*	Ash	*Culex quinquefasciatus*
CPIJ006629	*Mes-4*	Ash	*Culex quinquefasciatus*
CPIJ013265	*Mes-4*	Ash	*Culex quinquefasciatus*
CPIJ016171	*Mes-4*	Ash	*Culex quinquefasciatus*
CPIJ016632	*Mes-4*	Ash	*Culex quinquefasciatus*
CPIJ016840	*Mes-4*	Ash	*Culex quinquefasciatus*
CPIJ017018	*Mes-4*	Ash	*Culex quinquefasciatus*
CPIJ018290	*Mes-4*	Ash	*Culex quinquefasciatus*
CPIJ019579	*Mes-4*	Ash	*Culex quinquefasciatus*
CPIJ019697	*Mes-4*	Ash	*Culex quinquefasciatus*
CPIJ013516	*Set1*	Trx	*Culex quinquefasciatus*
CPIJ000435	*Set1*	Trx	*Culex quinquefasciatus*
CPIJ009561	*Set1*	Trx	*Culex quinquefasciatus*
CPIJ009853	*Set1*	Trx	*Culex quinquefasciatus*
CPIJ011937	*Set1*	Trx	*Culex quinquefasciatus*

CPIJ013517	*Set1*	Trx	*Culex quinquefasciatus*
CPIJ018240	*Set1*	Trx	*Culex quinquefasciatus*
CPIJ005876	*Set2*	Ash	*Culex quinquefasciatus*
CPIJ000732	*Set8*	-	*Culex quinquefasciatus*
CPIJ009666	*setd*	SETD	*Culex quinquefasciatus*
CPIJ009352	*Smyd4-1*	SMYD	*Culex quinquefasciatus*
CPIJ007239	*Smyd4-2*	SMYD	*Culex quinquefasciatus*
CPIJ015708	*Smyd4-3*	SMYD	*Culex quinquefasciatus*
CPIJ015707	*Smyd4-3*	SMYD	*Culex quinquefasciatus*
CPIJ006452	*Smyd4-4*	SMYD	*Culex quinquefasciatus*
CPIJ000744	*Smyd4-4*	SMYD	*Culex quinquefasciatus*
CPIJ000812	*Smyd4-4*	SMYD	*Culex quinquefasciatus*
CPIJ000815	*Smyd4-4*	SMYD	*Culex quinquefasciatus*
CPIJ002821	*Smyd4-4*	SMYD	*Culex quinquefasciatus*
CPIJ002828	*Smyd4-4*	SMYD	*Culex quinquefasciatus*
CPIJ004426	*Smyd4-4*	SMYD	*Culex quinquefasciatus*
CPIJ004427	*Smyd4-4*	SMYD	*Culex quinquefasciatus*
CPIJ004428	*Smyd4-4*	SMYD	*Culex quinquefasciatus*
CPIJ004429	*Smyd4-4*	SMYD	*Culex quinquefasciatus*
CPIJ004431	*Smyd4-4*	SMYD	*Culex quinquefasciatus*
CPIJ006454	*Smyd4-4*	SMYD	*Culex quinquefasciatus*
CPIJ006490	*Smyd4-4*	SMYD	*Culex quinquefasciatus*
CPIJ006632	*Smyd4-4*	SMYD	*Culex quinquefasciatus*
CPIJ006635	*Smyd4-4*	SMYD	*Culex quinquefasciatus*
CPIJ006636	*Smyd4-4*	SMYD	*Culex quinquefasciatus*
CPIJ006637	*Smyd4-4*	SMYD	*Culex quinquefasciatus*
CPIJ006638	*Smyd4-4*	SMYD	*Culex quinquefasciatus*
CPIJ006641	*Smyd4-4*	SMYD	*Culex quinquefasciatus*
CPIJ007544	*Smyd4-4*	SMYD	*Culex quinquefasciatus*
CPIJ008045	*Smyd4-4*	SMYD	*Culex quinquefasciatus*
CPIJ009784	*Smyd4-4*	SMYD	*Culex quinquefasciatus*
CPIJ010143	*Smyd4-4*	SMYD	*Culex quinquefasciatus*
CPIJ010371	*Smyd4-4*	SMYD	*Culex quinquefasciatus*
CPIJ010372	*Smyd4-4*	SMYD	*Culex quinquefasciatus*
CPIJ011021	*Smyd4-4*	SMYD	*Culex quinquefasciatus*
CPIJ011686	*Smyd4-4*	SMYD	*Culex quinquefasciatus*
CPIJ012096	*Smyd4-4*	SMYD	*Culex quinquefasciatus*
CPIJ014755	*Smyd4-4*	SMYD	*Culex quinquefasciatus*
CPIJ014756	*Smyd4-4*	SMYD	*Culex quinquefasciatus*
CPIJ014757	*Smyd4-4*	SMYD	*Culex quinquefasciatus*
CPIJ014758	*Smyd4-4*	SMYD	*Culex quinquefasciatus*
CPIJ015565	*Smyd4-4*	SMYD	*Culex quinquefasciatus*
CPIJ017838	*Smyd4-4*	SMYD	*Culex quinquefasciatus*
CPIJ019668	*Smyd4-4*	SMYD	*Culex quinquefasciatus*

CPIJ007481	*Smyd5*	SMYD	*Culex quinquefasciatus*
CPIJ015254	*SmydA-2*	SmydA	*Culex quinquefasciatus*
CPIJ000970	*SmydA-3*	SmydA	*Culex quinquefasciatus*
CPIJ013580	*SmydA-4*	SmydA	*Culex quinquefasciatus*
CPIJ014160	*SmydA-6*	SmydA	*Culex quinquefasciatus*
CPIJ018500	*SmydA-8*	SmydA	*Culex quinquefasciatus*
CPIJ008357	*SmydA-8*	SmydA	*Culex quinquefasciatus*
CPIJ019323	*Su(var)3-9*	Suv	*Culex quinquefasciatus*
CPIJ011192	*trr*	Trx	*Culex quinquefasciatus*
CPIJ013971	*trx*	Trx	*Culex quinquefasciatus*
CPIJ005719	*upSET(MLL5)*	-	*Culex quinquefasciatus*
DPGLEAN13830	*ash1*	Ash	*Danaus plexippus*
DPGLEAN16647	*Blimp-1*	PRDM	*Danaus plexippus*
DPGLEAN01117	*egg*	Suv	*Danaus plexippus*
DPGLEAN22333	*egg*	Suv	*Danaus plexippus*
DPGLEAN10295	*Ez*	Ez	*Danaus plexippus*
DPGLEAN13671	*G9a*	Suv	*Danaus plexippus*
DPGLEAN05424	*Hmt4-20*	-	*Danaus plexippus*
DPGLEAN14526	*Mes-4*	Ash	*Danaus plexippus*
DPGLEAN15585	*Set1*	Trx	*Danaus plexippus*
DPGLEAN00591	*Set2*	Ash	*Danaus plexippus*
DPGLEAN15467	*Set8*	-	*Danaus plexippus*
DPGLEAN04058	*setd*	SETD	*Danaus plexippus*
DPGLEAN08794	*Smyd4-1*	SMYD	*Danaus plexippus*
DPGLEAN14035	*Smyd4-2*	SMYD	*Danaus plexippus*
DPGLEAN11275	*Smyd4-3*	SMYD	*Danaus plexippus*
DPGLEAN11009	*Smyd4-4*	SMYD	*Danaus plexippus*
DPGLEAN16822	*Smyd5*	SMYD	*Danaus plexippus*
DPGLEAN20849	*SmydA-1*	SmydA	*Danaus plexippus*
DPGLEAN13875	*SmydA-2*	SmydA	*Danaus plexippus*
DPGLEAN22194	*SmydA-3*	SmydA	*Danaus plexippus*
DPGLEAN04190	*SmydA-4*	SmydA	*Danaus plexippus*
DPGLEAN13877	*SmydA-5*	SmydA	*Danaus plexippus*
DPGLEAN22198	*SmydA-6*	SmydA	*Danaus plexippus*
DPGLEAN20152	*SmydA-8*	SmydA	*Danaus plexippus*
DPGLEAN18698	*Su(var)3-9*	Suv	*Danaus plexippus*
DPGLEAN21423	*suv*	Suv	*Danaus plexippus*
DPGLEAN14792	*trr*	Trx	*Danaus plexippus*
DPGLEAN21663	*trx*	Trx	*Danaus plexippus*
DPGLEAN21857	*upSET(MLL5)*	-	*Danaus plexippus*
KZS21850.1	-	-	*Daphnia magna*
KZS04467.1	-	-	*Daphnia magna*
KZS17141.1	*ash1*	Ash	*Daphnia magna*
KZS14461.1	*Blimp-1*	PRDM	*Daphnia magna*

KZS16476.1	*egg*	Suv	*Daphnia magna*
KZS19503.1	*egg*	Suv	*Daphnia magna*
KZS17283.1	*Ez*	Ez	*Daphnia magna*
KZS14880.1	*G9a*	Suv	*Daphnia magna*
KZS07084.1	*Hmt4-20*	-	*Daphnia magna*
KZS12602.1	*Mes-4*	Ash	*Daphnia magna*
KZS16419.1	*Set1*	Trx	*Daphnia magna*
KZS07868.1	*Set2*	Ash	*Daphnia magna*
KZS15065.1	*Set8*	-	*Daphnia magna*
KZS12928.1	*setd*	SETD	*Daphnia magna*
KZS21738.1	*Smyd3*	SMYD	*Daphnia magna*
KZS11370.1	*Smyd4-1*	SMYD	*Daphnia magna*
KZS12439.1	*Smyd4-2*	SMYD	*Daphnia magna*
KZS18458.1	*Smyd4-3*	SMYD	*Daphnia magna*
KZS08827.1	*Smyd4-3*	SMYD	*Daphnia magna*
KZS12493.1	*Smyd4-3*	SMYD	*Daphnia magna*
KZS15207.1	*Smyd5*	SMYD	*Daphnia magna*
KZS05857.1	*SmydA-2*	SmydA	*Daphnia magna*
KZS13858.1	*SmydA-2*	SmydA	*Daphnia magna*
KZS13857.1	*SmydA-3*	SmydA	*Daphnia magna*
KZS11712.1	*SmydA-4*	SmydA	*Daphnia magna*
KZS11713.1	*SmydA-4*	SmydA	*Daphnia magna*
KZS18976.1	*SmydA-4*	SmydA	*Daphnia magna*
KZS16965.1	*Su(var)3-9*	Suv	*Daphnia magna*
KZS17710.1	*trr*	Trx	*Daphnia magna*
KZS15953.1	*trx*	Trx	*Daphnia magna*
KZS05732.1	*upSET(MLL5)*	-	*Daphnia magna*
58066	-	-	*Daphnia pulex*
39415	*ash1*	Ash	*Daphnia pulex*
52261	*Blimp-1*	PRDM	*Daphnia pulex*
6489	*Blimp-1*	PRDM	*Daphnia pulex*
304216	*egg*	Suv	*Daphnia pulex*
303556	*egg*	Suv	*Daphnia pulex*
300071	*Ez*	Ez	*Daphnia pulex*
67185	*Ez*	Ez	*Daphnia pulex*
96728	*G9a*	Suv	*Daphnia pulex*
329141	*Hmt4-20*	-	*Daphnia pulex*
308851	*Mes-4*	Ash	*Daphnia pulex*
301653	*Set1*	Trx	*Daphnia pulex*
319776	*Set2*	Ash	*Daphnia pulex*
311914	*Set8*	-	*Daphnia pulex*
317395	*setd*	SETD	*Daphnia pulex*
309882	*Smyd4-2*	SMYD	*Daphnia pulex*
68494	*Smyd4-3*	SMYD	*Daphnia pulex*

	221129	*Smyd4-3*	SMYD	*Daphnia pulex*
	305694	*Smyd4-3*	SMYD	*Daphnia pulex*
	312722	*Smyd4-3*	SMYD	*Daphnia pulex*
	300033	*Smyd5*	SMYD	*Daphnia pulex*
	2393	*SmydA-2*	SmydA	*Daphnia pulex*
	194440	*SmydA-2*	SmydA	*Daphnia pulex*
	98275	*SmydA-2*	SmydA	*Daphnia pulex*
	237516	*SmydA-3*	SmydA	*Daphnia pulex*
	120473	*SmydA-4*	SmydA	*Daphnia pulex*
	314752	*SmydA-4*	SmydA	*Daphnia pulex*
	306854	*Su(var)3-9*	Suv	*Daphnia pulex*
	41537	*suv*	Suv	*Daphnia pulex*
	51465	*trr*	Trx	*Daphnia pulex*
	105888	*trx*	Trx	*Daphnia pulex*
	219571	*upSET(MLL5)*	-	*Daphnia pulex*
ENN71551		-	-	*Dendroctonus ponderosae*
ENN74965		*ash1*	Ash	*Dendroctonus ponderosae*
ENN77198		*Blimp-1*	PRDM	*Dendroctonus ponderosae*
ENN78407		*Ez*	Ez	*Dendroctonus ponderosae*
ENN73908		*G9a*	Suv	*Dendroctonus ponderosae*
ENN80962		*Hmt4-20*	-	*Dendroctonus ponderosae*
ENN80424		*Mes-4*	Ash	*Dendroctonus ponderosae*
ENN77633		*Set1*	Trx	*Dendroctonus ponderosae*
ENN76193		*Set2*	Ash	*Dendroctonus ponderosae*
ENN71873		*Set8*	-	*Dendroctonus ponderosae*
ENN71869		*Set8*	-	*Dendroctonus ponderosae*
ENN78865		*setd*	SETD	*Dendroctonus ponderosae*
ENN79834		*Smyd4-1*	SMYD	*Dendroctonus ponderosae*
ENN76352		*Smyd4-3*	SMYD	*Dendroctonus ponderosae*
ENN71187		*Smyd4-4*	SMYD	*Dendroctonus ponderosae*
ENN70296		*Smyd4-4*	SMYD	*Dendroctonus ponderosae*
ENN81878		*Smyd5*	SMYD	*Dendroctonus ponderosae*
ENN77956		*SmydA-1*	SmydA	*Dendroctonus ponderosae*
ENN70601		*SmydA-1*	SmydA	*Dendroctonus ponderosae*
ENN76494		*SmydA-3*	SmydA	*Dendroctonus ponderosae*
ENN81700		*SmydA-4*	SmydA	*Dendroctonus ponderosae*
ENN70591		*SmydA-4*	SmydA	*Dendroctonus ponderosae*
ENN76549		*SmydA-5*	SmydA	*Dendroctonus ponderosae*
ENN76550		*SmydA-5*	SmydA	*Dendroctonus ponderosae*
ENN75282		*Su(var)3-9*	Suv	*Dendroctonus ponderosae*
ENN71731		*suv*	Suv	*Dendroctonus ponderosae*
ENN73813		*trr*	Trx	*Dendroctonus ponderosae*
ENN74118		*trx*	Trx	*Dendroctonus ponderosae*
ENN75450		*upSET(MLL5)*	-	*Dendroctonus ponderosae*

XP_008482060.1	-	-	*Diaphorina citri*
XP_017298542.1	*Blimp-1*	PRDM	*Diaphorina citri*
XP_008485751.1	*egg*	Suv	*Diaphorina citri*
XP_017304681.1	*Ez*	Ez	*Diaphorina citri*
XP_008486547.1	*Ez*	Ez	*Diaphorina citri*
XP_008472704.1	*Hmt4-20*	-	*Diaphorina citri*
XP_017305347.1	*Mes-4*	Ash	*Diaphorina citri*
XP_017305346.1	*Mes-4*	Ash	*Diaphorina citri*
XP_008476254.1	*Set1*	Trx	*Diaphorina citri*
XP_008472211.1	*Set2*	Ash	*Diaphorina citri*
XP_008486199.1	*Set2*	Ash	*Diaphorina citri*
XP_008471055.1	*Set8*	-	*Diaphorina citri*
XP_008473256.1	*Smyd4-2*	SMYD	*Diaphorina citri*
XP_017300041.1	*Smyd4-3*	SMYD	*Diaphorina citri*
XP_008478651.1	*Smyd4-3*	SMYD	*Diaphorina citri*
XP_017300620.1	*SmydA-1*	SmydA	*Diaphorina citri*
XP_017302778.1	*SmydA-2*	SmydA	*Diaphorina citri*
XP_017303780.1	*SmydA-3*	SmydA	*Diaphorina citri*
XP_008480211.1	*SmydA-4*	SmydA	*Diaphorina citri*
XP_008476173.1	*SmydA-5*	SmydA	*Diaphorina citri*
XP_017303757.1	*SmydA-6*	SmydA	*Diaphorina citri*
XP_008467522.1	*SmydA-8*	SmydA	*Diaphorina citri*
XP_017297848.1	*Su(var)3-9*	Suv	*Diaphorina citri*
XP_017304938.1	*Su(var)3-9*	Suv	*Diaphorina citri*
XP_017300763.1	*suv*	Suv	*Diaphorina citri*
XP_008474986.1	*trr*	Trx	*Diaphorina citri*
XP_008467635.1	*trx*	Trx	*Diaphorina citri*
XP_017305099.1	*upSET(MLL5)*	-	*Diaphorina citri*
GF10724	*ash1*	Ash	*Drosophila ananassae*
GF23985	*Blimp-1*	PRDM	*Drosophila ananassae*
GF11546	*egg*	Suv	*Drosophila ananassae*
GF23825	*Ez*	Ez	*Drosophila ananassae*
GF21998	*G9a*	Suv	*Drosophila ananassae*
GF21991	*Hmt4-20*	-	*Drosophila ananassae*
GF16138	*Mes-4*	Ash	*Drosophila ananassae*
GF23123	*Set1*	Trx	*Drosophila ananassae*
GF22528	*Set2*	Ash	*Drosophila ananassae*
GF17507	*Set8*	-	*Drosophila ananassae*
GF12496	*Smyd4-1*	SMYD	*Drosophila ananassae*
GF24983	*Smyd4-2*	SMYD	*Drosophila ananassae*
GF12554	*Smyd4-3*	SMYD	*Drosophila ananassae*
GF12292	*Smyd4-4*	SMYD	*Drosophila ananassae*
GF23162	*Smyd5*	SMYD	*Drosophila ananassae*
GF13486	*SmydA-1*	SmydA	*Drosophila ananassae*

GF10789	*SmydA-2*	SmydA	*Drosophila ananassae*
GF15829	*SmydA-3*	SmydA	*Drosophila ananassae*
GF20287	*SmydA-4*	SmydA	*Drosophila ananassae*
GF16861	*SmydA-5*	SmydA	*Drosophila ananassae*
GF11433	*SmydA-6*	SmydA	*Drosophila ananassae*
GF21975	*SmydA-8*	SmydA	*Drosophila ananassae*
GF21974	*SmydA-8*	SmydA	*Drosophila ananassae*
GF19100	*SmydA-9*	SmydA	*Drosophila ananassae*
GF11769	*Su(var)3-9*	Suv	*Drosophila ananassae*
GF17909	*suv*	Suv	*Drosophila ananassae*
GF19475	*trr*	Trx	*Drosophila ananassae*
GF23177	*trx*	Trx	*Drosophila ananassae*
GF24057	*upSET(MLL5)*	-	*Drosophila ananassae*
DBIA011526	*ash1*	Ash	*Drosophila biarmipes*
DBIA003686	*Blimp-1*	PRDM	*Drosophila biarmipes*
DBIA004550	*egg*	Suv	*Drosophila biarmipes*
DBIA000786	*Ez*	Ez	*Drosophila biarmipes*
DBIA005583	*G9a*	Suv	*Drosophila biarmipes*
DBIA005574	*Hmt4-20*	-	*Drosophila biarmipes*
DBIA005413	*Mes-4*	Ash	*Drosophila biarmipes*
DBIA014121	*Set1*	Trx	*Drosophila biarmipes*
DBIA010304	*Set2*	Ash	*Drosophila biarmipes*
DBIA015038	*Set2*	Ash	*Drosophila biarmipes*
DBIA005443	*Set8*	-	*Drosophila biarmipes*
DBIA005649	*setd*	SETD	*Drosophila biarmipes*
DBIA009626	*Smyd4-1*	SMYD	*Drosophila biarmipes*
DBIA000577	*Smyd4-2*	SMYD	*Drosophila biarmipes*
DBIA004220	*Smyd4-3*	SMYD	*Drosophila biarmipes*
DBIA006152	*Smyd4-4*	SMYD	*Drosophila biarmipes*
DBIA008708	*Smyd5*	SMYD	*Drosophila biarmipes*
DBIA012884	*SmydA-2*	SmydA	*Drosophila biarmipes*
DBIA002575	*SmydA-3*	SmydA	*Drosophila biarmipes*
DBIA007374	*SmydA-4*	SmydA	*Drosophila biarmipes*
DBIA015092	*SmydA-5*	SmydA	*Drosophila biarmipes*
DBIA004825	*SmydA-6*	SmydA	*Drosophila biarmipes*
DBIA005690	*SmydA-8*	SmydA	*Drosophila biarmipes*
DBIA001618	*Su(var)3-9*	Suv	*Drosophila biarmipes*
DBIA002203	*suv*	Suv	*Drosophila biarmipes*
DBIA005923	*trr*	Trx	*Drosophila biarmipes*
DBIA001478	*trx*	Trx	*Drosophila biarmipes*
DBIA000688	*upSET(MLL5)*	-	*Drosophila biarmipes*
DBIP005101	*ash1*	Ash	*Drosophila bipectinata*
DBIP003330	*Blimp-1*	PRDM	*Drosophila bipectinata*
DBIP005298	*egg*	Suv	*Drosophila bipectinata*

DBIP007894	*Ez*	Ez	*Drosophila bipectinata*
DBIP010902	*G9a*	Suv	*Drosophila bipectinata*
DBIP010914	*Hmt4-20*	-	*Drosophila bipectinata*
DBIP009384	*Mes-4*	Ash	*Drosophila bipectinata*
DBIP013590	*Set1*	Trx	*Drosophila bipectinata*
DBIP011040	*Set2*	Ash	*Drosophila bipectinata*
DBIP008532	*Set8*	-	*Drosophila bipectinata*
DBIP010359	*setd*	SETD	*Drosophila bipectinata*
DBIP002315	*Smyd4-1*	SMYD	*Drosophila bipectinata*
DBIP005673	*Smyd4-2*	SMYD	*Drosophila bipectinata*
DBIP002434	*Smyd4-3*	SMYD	*Drosophila bipectinata*
DBIP003016	*Smyd4-4*	SMYD	*Drosophila bipectinata*
DBIP007466	*Smyd5*	SMYD	*Drosophila bipectinata*
DBIP006513	*SmydA-1*	SmydA	*Drosophila bipectinata*
DBIP005205	*SmydA-2*	SmydA	*Drosophila bipectinata*
DBIP009899	*SmydA-4*	SmydA	*Drosophila bipectinata*
DBIP010612	*SmydA-5*	SmydA	*Drosophila bipectinata*
DBIP002871	*SmydA-6*	SmydA	*Drosophila bipectinata*
DBIP010941	*SmydA-8*	SmydA	*Drosophila bipectinata*
DBIP006373	*SmydA-9*	SmydA	*Drosophila bipectinata*
DBIP001170	*Su(var)3-9*	Suv	*Drosophila bipectinata*
DBIP000248	*suv*	Suv	*Drosophila bipectinata*
DBIP011959	*trr*	Trx	*Drosophila bipectinata*
DBIP007441	*trx*	Trx	*Drosophila bipectinata*
DBIP003443	*upSET(MLL5)*	-	*Drosophila bipectinata*
DELE000710	*ash1*	Ash	*Drosophila elegans*
DELE010093	*Blimp-1*	PRDM	*Drosophila elegans*
DELE001080	*egg*	Suv	*Drosophila elegans*
DELE006110	*Ez*	Ez	*Drosophila elegans*
DELE006766	*G9a*	Suv	*Drosophila elegans*
DELE006756	*Hmt4-20*	-	*Drosophila elegans*
DELE002522	*Mes-4*	Ash	*Drosophila elegans*
DELE014611	*Set1*	Trx	*Drosophila elegans*
DELE005122	*Set2*	Ash	*Drosophila elegans*
DELE007689	*Set8*	-	*Drosophila elegans*
DELE008681	*setd*	SETD	*Drosophila elegans*
DELE007991	*Smyd4-1*	SMYD	*Drosophila elegans*
DELE003489	*Smyd4-2*	SMYD	*Drosophila elegans*
DELE001156	*Smyd4-3*	SMYD	*Drosophila elegans*
DELE001241	*Smyd4-4*	SMYD	*Drosophila elegans*
DELE004218	*Smyd5*	SMYD	*Drosophila elegans*
DELE012077	*SmydA-3*	SmydA	*Drosophila elegans*
DELE009131	*SmydA-4*	SmydA	*Drosophila elegans*
DELE013895	*SmydA-5*	SmydA	*Drosophila elegans*

DELE000984	*SmydA-6*	SmydA	*Drosophila elegans*
DELE008544	*SmydA-8*	SmydA	*Drosophila elegans*
DELE013607	*SmydA-9*	SmydA	*Drosophila elegans*
DELE007522	*Su(var)3-9*	Suv	*Drosophila elegans*
DELE008495	*trr*	Trx	*Drosophila elegans*
DELE007695	*trx*	Trx	*Drosophila elegans*
DELE003651	*upSET(MLL5)*	-	*Drosophila elegans*
GG13392	*ash1*	Ash	*Drosophila erecta*
GG15278	*Blimp-1*	PRDM	*Drosophila erecta*
GG19893	*egg*	Suv	*Drosophila erecta*
GG13968	*Ez*	Ez	*Drosophila erecta*
GG12774	*G9a*	Suv	*Drosophila erecta*
GG12748	*Hmt4-20*	-	*Drosophila erecta*
GG12082	*Mes-4*	Ash	*Drosophila erecta*
GG11901	*Set1*	Trx	*Drosophila erecta*
GG17783	*Set2*	Ash	*Drosophila erecta*
GG16850	*Set8*	-	*Drosophila erecta*
GG17647	*setd*	SETD	*Drosophila erecta*
GG25277	*Smyd4-1*	SMYD	*Drosophila erecta*
GG13835	*Smyd4-2*	SMYD	*Drosophila erecta*
GG20202	*Smyd4-3*	SMYD	*Drosophila erecta*
GG20247	*Smyd4-4*	SMYD	*Drosophila erecta*
GG14794	*Smyd5*	SMYD	*Drosophila erecta*
GG20436	*SmydA-1*	SmydA	*Drosophila erecta*
GG13432	*SmydA-2*	SmydA	*Drosophila erecta*
GG23681	*SmydA-3*	SmydA	*Drosophila erecta*
GG18383	*SmydA-4*	SmydA	*Drosophila erecta*
GG10833	*SmydA-5*	SmydA	*Drosophila erecta*
GG20655	*SmydA-6*	SmydA	*Drosophila erecta*
GG20654	*SmydA-7*	SmydA	*Drosophila erecta*
GG12639	*SmydA-8*	SmydA	*Drosophila erecta*
GG12637	*SmydA-8*	SmydA	*Drosophila erecta*
GG18992	*SmydA-9*	SmydA	*Drosophila erecta*
GG17759	*suv*	Suv	*Drosophila erecta*
GG12672	*trr*	Trx	*Drosophila erecta*
GG21380	*trx*	Trx	*Drosophila erecta*
GG15666	*upSET(MLL5)*	-	*Drosophila erecta*
DEUG006429	*ash1*	Ash	*Drosophila eugracilis*
DEUG002757	*Blimp-1*	PRDM	*Drosophila eugracilis*
DEUG005809	*egg*	Suv	*Drosophila eugracilis*
DEUG001284	*Ez*	Ez	*Drosophila eugracilis*
DEUG006760	*G9a*	Suv	*Drosophila eugracilis*
DEUG006748	*Hmt4-20*	-	*Drosophila eugracilis*
DEUG005091	*Mes-4*	Ash	*Drosophila eugracilis*

DEUG013778	*Set1*	Trx	*Drosophila eugracilis*
DEUG011891	*Set2*	Ash	*Drosophila eugracilis*
DEUG010911	*Set8*	-	*Drosophila eugracilis*
DEUG009864	*Smyd4-1*	SMYD	*Drosophila eugracilis*
DEUG002891	*Smyd4-2*	SMYD	*Drosophila eugracilis*
DEUG003145	*Smyd4-3*	SMYD	*Drosophila eugracilis*
DEUG003059	*Smyd4-4*	SMYD	*Drosophila eugracilis*
DEUG003355	*SmydA-1*	SmydA	*Drosophila eugracilis*
DEUG002172	*SmydA-3*	SmydA	*Drosophila eugracilis*
DEUG011681	*SmydA-4*	SmydA	*Drosophila eugracilis*
DEUG011558	*SmydA-5*	SmydA	*Drosophila eugracilis*
DEUG000277	*SmydA-7*	SmydA	*Drosophila eugracilis*
DEUG013190	*SmydA-8*	SmydA	*Drosophila eugracilis*
DEUG012784	*SmydA-8*	SmydA	*Drosophila eugracilis*
DEUG008906	*SmydA-9*	SmydA	*Drosophila eugracilis*
DEUG013066	*Su(var)3-9*	Suv	*Drosophila eugracilis*
DEUG011271	*trr*	Trx	*Drosophila eugracilis*
DEUG010918	*trx*	Trx	*Drosophila eugracilis*
DEUG007119	*upSET(MLL5)*	-	*Drosophila eugracilis*
DFIC010692	*ash1*	Ash	*Drosophila ficusphila*
DFIC008679	*Blimp-1*	PRDM	*Drosophila ficusphila*
DFIC005281	*egg*	Suv	*Drosophila ficusphila*
DFIC008708	*Ez*	Ez	*Drosophila ficusphila*
DFIC006626	*G9a*	Suv	*Drosophila ficusphila*
DFIC006613	*Hmt4-20*	-	*Drosophila ficusphila*
DFIC007323	*Mes-4*	Ash	*Drosophila ficusphila*
DFIC013431	*Set1*	Trx	*Drosophila ficusphila*
DFIC005874	*Set2*	Ash	*Drosophila ficusphila*
DFIC001678	*Set8*	-	*Drosophila ficusphila*
DFIC006683	*setd*	SETD	*Drosophila ficusphila*
DFIC005344	*Smyd4-1*	SMYD	*Drosophila ficusphila*
DFIC004048	*Smyd4-2*	SMYD	*Drosophila ficusphila*
DFIC011548	*Smyd4-3*	SMYD	*Drosophila ficusphila*
DFIC008914	*Smyd4-4*	SMYD	*Drosophila ficusphila*
DFIC006261	*Smyd5*	SMYD	*Drosophila ficusphila*
DFIC010240	*SmydA-2*	SmydA	*Drosophila ficusphila*
DFIC009417	*SmydA-3*	SmydA	*Drosophila ficusphila*
DFIC007675	*SmydA-4*	SmydA	*Drosophila ficusphila*
DFIC001193	*SmydA-8*	SmydA	*Drosophila ficusphila*
DFIC005735	*SmydA-9*	SmydA	*Drosophila ficusphila*
DFIC006784	*Su(var)3-9*	Suv	*Drosophila ficusphila*
DFIC001143	*trr*	Trx	*Drosophila ficusphila*
DFIC001671	*trx*	Trx	*Drosophila ficusphila*
DFIC009646	*upSET(MLL5)*	-	*Drosophila ficusphila*

GH16622	-	-	*Drosophila grimshawi*
GH14625	*ash1*	Ash	*Drosophila grimshawi*
GH16462	*Blimp-1*	PRDM	*Drosophila grimshawi*
GH21540	*egg*	Suv	*Drosophila grimshawi*
GH16544	*Ez*	Ez	*Drosophila grimshawi*
GH24232	*G9a*	Suv	*Drosophila grimshawi*
GH17704	*Hmt4-20*	-	*Drosophila grimshawi*
GH19166	*Mes-4*	Ash	*Drosophila grimshawi*
GH22347	*Set1*	Trx	*Drosophila grimshawi*
GH16034	*Set2*	Ash	*Drosophila grimshawi*
GH17478	*Set8*	-	*Drosophila grimshawi*
GH12451	*setd*	SETD	*Drosophila grimshawi*
GH21957	*Smyd4-1*	SMYD	*Drosophila grimshawi*
GH16068	*Smyd4-2*	SMYD	*Drosophila grimshawi*
GH21386	*Smyd4-3*	SMYD	*Drosophila grimshawi*
GH20862	*Smyd4-4*	SMYD	*Drosophila grimshawi*
GH18189	*Smyd5*	SMYD	*Drosophila grimshawi*
GH19808	*SmydA-1*	SmydA	*Drosophila grimshawi*
GH16955	*SmydA-2*	SmydA	*Drosophila grimshawi*
GH13590	*SmydA-3*	SmydA	*Drosophila grimshawi*
GH17917	*SmydA-4*	SmydA	*Drosophila grimshawi*
GH20950	*SmydA-5*	SmydA	*Drosophila grimshawi*
GH22837	*SmydA-6*	SmydA	*Drosophila grimshawi*
GH12314	*SmydA-8*	SmydA	*Drosophila grimshawi*
GH17754	*SmydA-9*	SmydA	*Drosophila grimshawi*
GH18750	*Su(var)3-9*	Suv	*Drosophila grimshawi*
GH17848	*trr*	Trx	*Drosophila grimshawi*
GH19675	*trx*	Trx	*Drosophila grimshawi*
GH16924	*upSET(MLL5)*	-	*Drosophila grimshawi*
DKIK011252	*ash1*	Ash	*Drosophila kikkawai*
DKIK012019	*Blimp-1*	PRDM	*Drosophila kikkawai*
DKIK008447	*egg*	Suv	*Drosophila kikkawai*
DKIK007571	*Ez*	Ez	*Drosophila kikkawai*
DKIK003877	*G9a*	Suv	*Drosophila kikkawai*
DKIK003556	*Hmt4-20*	-	*Drosophila kikkawai*
DKIK002285	*Mes-4*	Ash	*Drosophila kikkawai*
DKIK013623	*Set1*	Trx	*Drosophila kikkawai*
DKIK005841	*Set2*	Ash	*Drosophila kikkawai*
DKIK002038	*Set8*	-	*Drosophila kikkawai*
DKIK003896	*setd*	SETD	*Drosophila kikkawai*
DKIK000112	*Smyd4-1*	SMYD	*Drosophila kikkawai*
DKIK005683	*Smyd4-2*	SMYD	*Drosophila kikkawai*
DKIK000033	*Smyd4-3*	SMYD	*Drosophila kikkawai*
DKIK003085	*Smyd4-4*	SMYD	*Drosophila kikkawai*

DKIK011716	*Smyd5*	SMYD	*Drosophila kikkawai*
DKIK008438	*SmydA-1*	SmydA	*Drosophila kikkawai*
DKIK012860	*SmydA-2*	SmydA	*Drosophila kikkawai*
DKIK000670	*SmydA-3*	SmydA	*Drosophila kikkawai*
DKIK011193	*SmydA-4*	SmydA	*Drosophila kikkawai*
DKIK009956	*SmydA-5*	SmydA	*Drosophila kikkawai*
DKIK004100	*SmydA-8*	SmydA	*Drosophila kikkawai*
DKIK004389	*SmydA-9*	SmydA	*Drosophila kikkawai*
DKIK002140	*Su(var)3-9*	Suv	*Drosophila kikkawai*
DKIK010545	*suv*	Suv	*Drosophila kikkawai*
DKIK005234	*trr*	Trx	*Drosophila kikkawai*
DKIK009928	*trx*	Trx	*Drosophila kikkawai*
DKIK001048	*upSET(MLL5)*	-	*Drosophila kikkawai*
CG8887	*ash1*	Ash	*Drosophila melanogaster*
CG5249	*Blimp-1*	PRDM	*Drosophila melanogaster*
CG12196	*egg*	Suv	*Drosophila melanogaster*
CG6502	*Ez*	Ez	*Drosophila melanogaster*
CG2995	*G9a*	Suv	*Drosophila melanogaster*
CG13363	*Hmt4-20*	-	*Drosophila melanogaster*
CG4976	*Mes-4*	Ash	*Drosophila melanogaster*
CG40351	*Set1*	Trx	*Drosophila melanogaster*
CG1716	*Set2*	Ash	*Drosophila melanogaster*
CG3307	*Set8*	-	*Drosophila melanogaster*
CG32732	*setd*	SETD	*Drosophila melanogaster*
CG1868	*Smyd4-1*	SMYD	*Drosophila melanogaster*
CG14122	*Smyd4-2*	SMYD	*Drosophila melanogaster*
CG7759	*Smyd4-3*	SMYD	*Drosophila melanogaster*
CG8378	*Smyd4-4*	SMYD	*Drosophila melanogaster*
CG3353	*Smyd5*	SMYD	*Drosophila melanogaster*
CG8503	*SmydA-1*	SmydA	*Drosophila melanogaster*
CG18136	*SmydA-2*	SmydA	*Drosophila melanogaster*
CG43129	*SmydA-3*	SmydA	*Drosophila melanogaster*
CG11160	*SmydA-4*	SmydA	*Drosophila melanogaster*
CG14590	*SmydA-5*	SmydA	*Drosophila melanogaster*
CG9642	*SmydA-6*	SmydA	*Drosophila melanogaster*
CG9640	*SmydA-7*	SmydA	*Drosophila melanogaster*
CG33548	*SmydA-8*	SmydA	*Drosophila melanogaster*
CG12119	*SmydA-9*	SmydA	*Drosophila melanogaster*
CG43664	*Su(var)3-9*	Suv	*Drosophila melanogaster*
CG4565	*suv*	Suv	*Drosophila melanogaster*
CG3848	*trr*	Trx	*Drosophila melanogaster*
CG8651	*trx*	Trx	*Drosophila melanogaster*
CG9007	*upSET(MLL5)*	-	*Drosophila melanogaster*
XP_017135172.1	*ash1*	Ash	*Drosophila miranda*

XP_017147643.1	*egg*	Suv	*Drosophila miranda*
XP_017135503.1	*Ez*	Ez	*Drosophila miranda*
XP_017156014.1	*Hmt4-20*	-	*Drosophila miranda*
XP_017141202.1	*Mes-4*	Ash	*Drosophila miranda*
XP_017140974.1	*Set1*	Trx	*Drosophila miranda*
XP_017142962.1	*Set2*	Ash	*Drosophila miranda*
XP_017141104.1	*Set8*	-	*Drosophila miranda*
XP_017152710.1	*setd*	SETD	*Drosophila miranda*
XP_017145755.1	*Smyd4-1*	SMYD	*Drosophila miranda*
XP_017137452.1	*Smyd4-2*	SMYD	*Drosophila miranda*
XP_017150457.1	*Smyd4-3*	SMYD	*Drosophila miranda*
XP_017147620.1	*Smyd4-4*	SMYD	*Drosophila miranda*
XP_017141239.1	*Smyd5*	SMYD	*Drosophila miranda*
XP_017146532.1	*SmydA-1*	SmydA	*Drosophila miranda*
XP_017134743.1	*SmydA-2*	SmydA	*Drosophila miranda*
XP_017156586.1	*SmydA-4*	SmydA	*Drosophila miranda*
XP_017149995.1	*SmydA-5*	SmydA	*Drosophila miranda*
XP_017147812.1	*SmydA-6*	SmydA	*Drosophila miranda*
XP_017148482.1	*SmydA-8*	SmydA	*Drosophila miranda*
XP_017148473.1	*SmydA-8*	SmydA	*Drosophila miranda*
XP_017138378.1	*SmydA-9*	SmydA	*Drosophila miranda*
XP_017140697.1	*Su(var)3-9*	Suv	*Drosophila miranda*
XP_017139499.1	*suv*	Suv	*Drosophila miranda*
XP_017145180.1	*trr*	Trx	*Drosophila miranda*
XP_017141807.1	*trx*	Trx	*Drosophila miranda*
XP_017135809.1	*upSET(MLL5)*	-	*Drosophila miranda*
GI12469	-	-	*Drosophila mojavensis*
GI13513	*ash1*	Ash	*Drosophila mojavensis*
GI19513	*egg*	Suv	*Drosophila mojavensis*
GI13833	*Ez*	Ez	*Drosophila mojavensis*
GI21426	*G9a*	Suv	*Drosophila mojavensis*
GI16112	*Hmt4-20*	-	*Drosophila mojavensis*
GI23270	*Mes-4*	Ash	*Drosophila mojavensis*
GI12297	*Set2*	Ash	*Drosophila mojavensis*
GI15146	*Set2*	Ash	*Drosophila mojavensis*
GI22805	*Set8*	-	*Drosophila mojavensis*
GI21676	*setd*	SETD	*Drosophila mojavensis*
GI19663	*Smyd4-1*	SMYD	*Drosophila mojavensis*
GI12333	*Smyd4-2*	SMYD	*Drosophila mojavensis*
GI18563	*Smyd4-3*	SMYD	*Drosophila mojavensis*
GI20970	*Smyd4-4*	SMYD	*Drosophila mojavensis*
GI10143	*Smyd5*	SMYD	*Drosophila mojavensis*
GI19093	*SmydA-1*	SmydA	*Drosophila mojavensis*
GI11814	*SmydA-2*	SmydA	*Drosophila mojavensis*

GI23580	*SmydA-3*	SmydA	*Drosophila mojavensis*
GI16059	*SmydA-4*	SmydA	*Drosophila mojavensis*
GI21287	*SmydA-5*	SmydA	*Drosophila mojavensis*
GI21216	*SmydA-6*	SmydA	*Drosophila mojavensis*
GI21214	*SmydA-7*	SmydA	*Drosophila mojavensis*
GI21459	*SmydA-8*	SmydA	*Drosophila mojavensis*
GI21458	*SmydA-8*	SmydA	*Drosophila mojavensis*
GI15088	*SmydA-9*	SmydA	*Drosophila mojavensis*
GI24667	*Su(var)3-9*	Suv	*Drosophila mojavensis*
GI21761	*trr*	Trx	*Drosophila mojavensis*
GI24532	*trr*	Trx	*Drosophila mojavensis*
GI13679	*upSET(MLL5)*	-	*Drosophila mojavensis*
GL20883	*ash1*	Ash	*Drosophila persimilis*
GL12308	*Blimp-1*	PRDM	*Drosophila persimilis*
GL17699	*egg*	Suv	*Drosophila persimilis*
GL17700	*egg*	Suv	*Drosophila persimilis*
GL15590	*Ez*	Ez	*Drosophila persimilis*
GL13363	*G9a*	Suv	*Drosophila persimilis*
GL13360	*Hmt4-20*	-	*Drosophila persimilis*
GL23881	*Mes-4*	Ash	*Drosophila persimilis*
GL12290	*Set1*	Trx	*Drosophila persimilis*
GL27000	*Set2*	Ash	*Drosophila persimilis*
GL25818	*Set2*	Ash	*Drosophila persimilis*
GL23503	*Set8*	-	*Drosophila persimilis*
GL13316	*setd*	SETD	*Drosophila persimilis*
GL20110	*Smyd4-1*	SMYD	*Drosophila persimilis*
GL24960	*Smyd4-2*	SMYD	*Drosophila persimilis*
GL11690	*Smyd4-3*	SMYD	*Drosophila persimilis*
GL16991	*Smyd4-4*	SMYD	*Drosophila persimilis*
GL12277	*Smyd5*	SMYD	*Drosophila persimilis*
GL10122	*SmydA-1*	SmydA	*Drosophila persimilis*
GL21959	*SmydA-2*	SmydA	*Drosophila persimilis*
GL19122	*SmydA-3*	SmydA	*Drosophila persimilis*
GL18252	*SmydA-4*	SmydA	*Drosophila persimilis*
GL11805	*SmydA-5*	SmydA	*Drosophila persimilis*
GL17552	*SmydA-6*	SmydA	*Drosophila persimilis*
GL13396	*SmydA-8*	SmydA	*Drosophila persimilis*
GL26796	*SmydA-9*	SmydA	*Drosophila persimilis*
GL12073	*Su(var)3-9*	Suv	*Drosophila persimilis*
GL21921	*suv*	Suv	*Drosophila persimilis*
GL18348	*trr*	Trx	*Drosophila persimilis*
GL23289	*trx*	Trx	*Drosophila persimilis*
GL17957	*upSET(MLL5)*	-	*Drosophila persimilis*
GA21391	*ash1*	Ash	*Drosophila pseudoobscura*

GA23469	*Blimp-1*	PRDM	*Drosophila pseudoobscura*
GA24879	*egg*	Suv	*Drosophila pseudoobscura*
GA19644	*Ez*	Ez	*Drosophila pseudoobscura*
GA15565	*G9a*	Suv	*Drosophila pseudoobscura*
GA22871	*Hmt4-20*	-	*Drosophila pseudoobscura*
GA18567	*Mes-4*	Ash	*Drosophila pseudoobscura*
GA14357	*Mes-4*	Ash	*Drosophila pseudoobscura*
GA26260	*Set1*	Trx	*Drosophila pseudoobscura*
GA25456	*Set2*	Ash	*Drosophila pseudoobscura*
GA17259	*Set8*	-	*Drosophila pseudoobscura*
GA17108	*setd*	SETD	*Drosophila pseudoobscura*
GA24934	*Smyd4-1*	SMYD	*Drosophila pseudoobscura*
GA12774	*Smyd4-2*	SMYD	*Drosophila pseudoobscura*
GA20567	*Smyd4-3*	SMYD	*Drosophila pseudoobscura*
GA21031	*Smyd4-4*	SMYD	*Drosophila pseudoobscura*
GA17400	*Smyd5*	SMYD	*Drosophila pseudoobscura*
GA25085	*SmydA-1*	SmydA	*Drosophila pseudoobscura*
GA14810	*SmydA-2*	SmydA	*Drosophila pseudoobscura*
GA14319	*SmydA-3*	SmydA	*Drosophila pseudoobscura*
GA10806	*SmydA-4*	SmydA	*Drosophila pseudoobscura*
GA13100	*SmydA-5*	SmydA	*Drosophila pseudoobscura*
GA21935	*SmydA-6*	SmydA	*Drosophila pseudoobscura*
GA22855	*SmydA-8*	SmydA	*Drosophila pseudoobscura*
GA11413	*SmydA-9*	SmydA	*Drosophila pseudoobscura*
GA19622	*Su(var)3-9*	Suv	*Drosophila pseudoobscura*
GA18262	*suv*	Suv	*Drosophila pseudoobscura*
GA17728	*trr*	Trx	*Drosophila pseudoobscura*
GA27210	*trx*	Trx	*Drosophila pseudoobscura*
GA21472	*upSET(MLL5)*	-	*Drosophila pseudoobscura*
DRHO008775	*ash1*	Ash	*Drosophila rhopaloa*
DRHO004459	*Blimp-1*	PRDM	*Drosophila rhopaloa*
DRHO003622	*egg*	Suv	*Drosophila rhopaloa*
DRHO001312	*Ez*	Ez	*Drosophila rhopaloa*
DRHO000257	*G9a*	Suv	*Drosophila rhopaloa*
DRHO000247	*Hmt4-20*	-	*Drosophila rhopaloa*
DRHO001671	*Mes-4*	Ash	*Drosophila rhopaloa*
DRHO003718	*Set2*	Ash	*Drosophila rhopaloa*
DRHO013383	*Set8*	-	*Drosophila rhopaloa*
DRHO002745	*setd*	SETD	*Drosophila rhopaloa*
DRHO001540	*Smyd4-1*	SMYD	*Drosophila rhopaloa*
DRHO007167	*Smyd4-2*	SMYD	*Drosophila rhopaloa*
DRHO003666	*Smyd4-3*	SMYD	*Drosophila rhopaloa*
DRHO002503	*Smyd4-4*	SMYD	*Drosophila rhopaloa*
DRHO005712	*Smyd5*	SMYD	*Drosophila rhopaloa*

DRHO009589	*SmydA-3*	SmydA	*Drosophila rhopaloa*
DRHO004823	*SmydA-4*	SmydA	*Drosophila rhopaloa*
DRHO000187	*SmydA-8*	SmydA	*Drosophila rhopaloa*
DRHO000188	*SmydA-8*	SmydA	*Drosophila rhopaloa*
DRHO012492	*SmydA-9*	SmydA	*Drosophila rhopaloa*
DRHO012570	*Su(var)3-9*	Suv	*Drosophila rhopaloa*
DRHO012691	*Su(var)3-9*	Suv	*Drosophila rhopaloa*
DRHO009481	*suv*	Suv	*Drosophila rhopaloa*
DRHO000294	*trr*	Trx	*Drosophila rhopaloa*
DRHO009111	*trx*	Trx	*Drosophila rhopaloa*
DRHO006710	*upSET(MLL5)*	-	*Drosophila rhopaloa*
GM17444	*ash1*	Ash	*Drosophila sechellia*
GM14713	*Blimp-1*	PRDM	*Drosophila sechellia*
GM11794	*egg*	Suv	*Drosophila sechellia*
GM24804	*Ez*	Ez	*Drosophila sechellia*
GM19051	*G9a*	Suv	*Drosophila sechellia*
GM19029	*Hmt4-20*	-	*Drosophila sechellia*
GM13547	*Mes-4*	Ash	*Drosophila sechellia*
GM18767	*Set1*	Trx	*Drosophila sechellia*
GM11634	*Set2*	Ash	*Drosophila sechellia*
GM24161	*Set8*	-	*Drosophila sechellia*
GM17489	*setd*	SETD	*Drosophila sechellia*
GM20595	*Smyd4-1*	SMYD	*Drosophila sechellia*
GM24661	*Smyd4-2*	SMYD	*Drosophila sechellia*
GM21288	*Smyd4-3*	SMYD	*Drosophila sechellia*
GM21333	*Smyd4-4*	SMYD	*Drosophila sechellia*
GM15092	*Smyd5*	SMYD	*Drosophila sechellia*
GM21522	*SmydA-1*	SmydA	*Drosophila sechellia*
GM14901	*SmydA-2*	SmydA	*Drosophila sechellia*
GM18755	*SmydA-3*	SmydA	*Drosophila sechellia*
GM11450	*SmydA-4*	SmydA	*Drosophila sechellia*
GM16483	*SmydA-5*	SmydA	*Drosophila sechellia*
GM21751	*SmydA-6*	SmydA	*Drosophila sechellia*
GM18906	*SmydA-8*	SmydA	*Drosophila sechellia*
GM11659	*SmydA-9*	SmydA	*Drosophila sechellia*
GM25792	*Su(var)3-9*	Suv	*Drosophila sechellia*
GM23905	*suv*	Suv	*Drosophila sechellia*
GM18944	*trr*	Trx	*Drosophila sechellia*
GM25445	*upSET(MLL5)*	-	*Drosophila sechellia*
GD12268	*ash1*	Ash	*Drosophila simulans*
GD12857	*Ez*	Ez	*Drosophila simulans*
GD16494	*G9a*	Suv	*Drosophila simulans*
GD16466	*Hmt4-20*	-	*Drosophila simulans*
GD18047	*Mes-4*	Ash	*Drosophila simulans*

GD11928	*Set1*	Trx	*Drosophila simulans*
GD17127	*Set2*	Ash	*Drosophila simulans*
GD18957	*Set8*	-	*Drosophila simulans*
GD16174	*setd*	SETD	*Drosophila simulans*
GD10068	*Smyd4-1*	SMYD	*Drosophila simulans*
GD12724	*Smyd4-2*	SMYD	*Drosophila simulans*
GD10796	*Smyd4-3*	SMYD	*Drosophila simulans*
GD10841	*Smyd4-4*	SMYD	*Drosophila simulans*
GD19999	*Smyd5*	SMYD	*Drosophila simulans*
GD12310	*SmydA-2*	SmydA	*Drosophila simulans*
GD23739	*SmydA-3*	SmydA	*Drosophila simulans*
GD16997	*SmydA-4*	SmydA	*Drosophila simulans*
GD10336	*SmydA-5*	SmydA	*Drosophila simulans*
GD11242	*SmydA-6*	SmydA	*Drosophila simulans*
GD16076	*SmydA-9*	SmydA	*Drosophila simulans*
GD20368	*Su(var)3-9*	Suv	*Drosophila simulans*
GD18718	*suv*	Suv	*Drosophila simulans*
GD16408	*trr*	Trx	*Drosophila simulans*
GD20433	*trx*	Trx	*Drosophila simulans*
GD14471	*upSET(MLL5)*	-	*Drosophila simulans*
XP_016931063.1	*ash1*	Ash	*Drosophila suzukii*
XP_016932019.1	*Blimp-1*	PRDM	*Drosophila suzukii*
XP_016930363.1	*egg*	Suv	*Drosophila suzukii*
XP_016934515.1	*Ez*	Ez	*Drosophila suzukii*
XP_016939221.1	*G9a*	Suv	*Drosophila suzukii*
XP_016939119.1	*Hmt4-20*	-	*Drosophila suzukii*
XP_016940034.1	*Mes-4*	Ash	*Drosophila suzukii*
XP_016940252.1	*Set1*	Trx	*Drosophila suzukii*
XP_016939483.1	*Set1*	Trx	*Drosophila suzukii*
XP_016923155.1	*Set2*	Ash	*Drosophila suzukii*
XP_016940022.1	*Set8*	-	*Drosophila suzukii*
XP_016939300.1	*setd*	SETD	*Drosophila suzukii*
XP_016928575.1	*Smyd4-1*	SMYD	*Drosophila suzukii*
XP_016933469.1	*Smyd4-2*	SMYD	*Drosophila suzukii*
XP_016929914.1	*Smyd4-3*	SMYD	*Drosophila suzukii*
XP_016930447.1	*Smyd4-4*	SMYD	*Drosophila suzukii*
XP_016923380.1	*Smyd5*	SMYD	*Drosophila suzukii*
XP_016927879.1	*SmydA-1*	SmydA	*Drosophila suzukii*
XP_016938466.1	*SmydA-2*	SmydA	*Drosophila suzukii*
XP_016945453.1	*SmydA-3*	SmydA	*Drosophila suzukii*
XP_016931479.1	*SmydA-5*	SmydA	*Drosophila suzukii*
XP_016928680.1	*SmydA-6*	SmydA	*Drosophila suzukii*
XP_016938707.1	*SmydA-8*	SmydA	*Drosophila suzukii*
XP_016938911.1	*SmydA-8*	SmydA	*Drosophila suzukii*

XP_016936678.1	*SmydA-9*	SmydA	*Drosophila suzukii*
XP_016923509.1	*Su(var)3-9*	Suv	*Drosophila suzukii*
XP_016941346.1	*suv*	Suv	*Drosophila suzukii*
XP_016939208.1	*trr*	Trx	*Drosophila suzukii*
XP_016938534.1	*trx*	Trx	*Drosophila suzukii*
DTAK011320	*ash1*	Ash	*Drosophila takahashii*
DTAK002609	*Blimp-1*	PRDM	*Drosophila takahashii*
DTAK004246	*egg*	Suv	*Drosophila takahashii*
DTAK008728	*Ez*	Ez	*Drosophila takahashii*
DTAK007517	*G9a*	Suv	*Drosophila takahashii*
DTAK006501	*Hmt4-20*	-	*Drosophila takahashii*
DTAK001799	*Mes-4*	Ash	*Drosophila takahashii*
DTAK012585	*Set1*	Trx	*Drosophila takahashii*
DTAK009746	*Set2*	Ash	*Drosophila takahashii*
DTAK005184	*Set8*	-	*Drosophila takahashii*
DTAK004856	*setd*	SETD	*Drosophila takahashii*
DTAK001101	*Smyd4-1*	SMYD	*Drosophila takahashii*
DTAK009537	*Smyd4-3*	SMYD	*Drosophila takahashii*
DTAK009631	*Smyd4-4*	SMYD	*Drosophila takahashii*
DTAK003239	*Smyd5*	SMYD	*Drosophila takahashii*
DTAK009409	*SmydA-2*	SmydA	*Drosophila takahashii*
DTAK008828	*SmydA-3*	SmydA	*Drosophila takahashii*
DTAK000988	*SmydA-4*	SmydA	*Drosophila takahashii*
DTAK010973	*SmydA-9*	SmydA	*Drosophila takahashii*
DTAK001868	*Su(var)3-9*	Suv	*Drosophila takahashii*
DTAK003541	*suv*	Suv	*Drosophila takahashii*
DTAK000910	*trr*	Trx	*Drosophila takahashii*
DTAK000774	*trx*	Trx	*Drosophila takahashii*
DTAK004471	*upSET(MLL5)*	-	*Drosophila takahashii*
GJ14055	-	-	*Drosophila virilis*
GJ11874	*ash1*	Ash	*Drosophila virilis*
GJ13412	*Blimp-1*	PRDM	*Drosophila virilis*
GJ21082	*egg*	Suv	*Drosophila virilis*
GJ11699	*Ez*	Ez	*Drosophila virilis*
GJ16443	*G9a*	Suv	*Drosophila virilis*
GJ15764	*Hmt4-20*	-	*Drosophila virilis*
GJ10771	*Mes-4*	Ash	*Drosophila virilis*
GJ23622	*Set1*	Trx	*Drosophila virilis*
GJ13235	*Set2*	Ash	*Drosophila virilis*
GJ19006	*Set2*	Ash	*Drosophila virilis*
GJ22807	*Set8*	-	*Drosophila virilis*
GJ16703	*setd*	SETD	*Drosophila virilis*
GJ15028	*Smyd4-1*	SMYD	*Drosophila virilis*
GJ12224	*Smyd4-2*	SMYD	*Drosophila virilis*

GJ20356	*Smyd4-3*	SMYD	*Drosophila virilis*
GJ20689	*Smyd4-4*	SMYD	*Drosophila virilis*
GJ23360	*Smyd5*	SMYD	*Drosophila virilis*
GJ20068	*SmydA-1*	SmydA	*Drosophila virilis*
GJ13517	*SmydA-2*	SmydA	*Drosophila virilis*
GJ20777	*SmydA-3*	SmydA	*Drosophila virilis*
GJ15632	*SmydA-4*	SmydA	*Drosophila virilis*
GJ20889	*SmydA-5*	SmydA	*Drosophila virilis*
GJ20818	*SmydA-6*	SmydA	*Drosophila virilis*
GJ16480	*SmydA-8*	SmydA	*Drosophila virilis*
GJ18896	*SmydA-9*	SmydA	*Drosophila virilis*
GJ24041	*Su(var)3-9*	Suv	*Drosophila virilis*
GJ10588	*suv*	Suv	*Drosophila virilis*
GJ19853	*trr*	Trx	*Drosophila virilis*
GJ19139	*trr*	Trx	*Drosophila virilis*
trx	*trx*	Trx	*Drosophila virilis*
GJ14017	*upSET(MLL5)*	-	*Drosophila virilis*
GK20252	*ash1*	Ash	*Drosophila willistoni*
GK17303	*Blimp-1*	PRDM	*Drosophila willistoni*
GK21652	*egg*	Suv	*Drosophila willistoni*
GK12358	*Ez*	Ez	*Drosophila willistoni*
GK24998	*G9a*	Suv	*Drosophila willistoni*
GK16179	*Hmt4-20*	-	*Drosophila willistoni*
GK14418	*Mes-4*	Ash	*Drosophila willistoni*
GK12911	*Set1*	Trx	*Drosophila willistoni*
GK11134	*Set1*	Trx	*Drosophila willistoni*
GK25076	*Set2*	Ash	*Drosophila willistoni*
GK10941	*Set8*	-	*Drosophila willistoni*
GK16171	*setd*	SETD	*Drosophila willistoni*
GK21490	*Smyd4-1*	SMYD	*Drosophila willistoni*
GK20590	*Smyd4-2*	SMYD	*Drosophila willistoni*
GK19653	*Smyd4-4*	SMYD	*Drosophila willistoni*
GK12748	*Smyd5*	SMYD	*Drosophila willistoni*
GK20779	*SmydA-1*	SmydA	*Drosophila willistoni*
GK12548	*SmydA-2*	SmydA	*Drosophila willistoni*
GK19981	*SmydA-4*	SmydA	*Drosophila willistoni*
GK21998	*SmydA-5*	SmydA	*Drosophila willistoni*
GK17864	*SmydA-6*	SmydA	*Drosophila willistoni*
GK21977	*SmydA-7*	SmydA	*Drosophila willistoni*
GK23578	*SmydA-8*	SmydA	*Drosophila willistoni*
GK25543	*SmydA-9*	SmydA	*Drosophila willistoni*
GK12089	*Su(var)3-9*	Suv	*Drosophila willistoni*
GK25046	*Su(var)3-9*	Suv	*Drosophila willistoni*
GK13904	*suv*	Suv	*Drosophila willistoni*

GK16377	*trr*	Trx	*Drosophila willistoni*
GK10891	*trx*	Trx	*Drosophila willistoni*
GK16792	*upSET(MLL5)*	-	*Drosophila willistoni*
GE22484	*ash1*	Ash	*Drosophila yakuba*
GE21501	*Blimp-1*	PRDM	*Drosophila yakuba*
GE11418	*egg*	Suv	*Drosophila yakuba*
GE20265	*Ez*	Ez	*Drosophila yakuba*
GE16600	*G9a*	Suv	*Drosophila yakuba*
GE16574	*Hmt4-20*	-	*Drosophila yakuba*
GE10528	*Mes-4*	Ash	*Drosophila yakuba*
GE25383	*Set1*	Trx	*Drosophila yakuba*
GE17076	*Set2*	Ash	*Drosophila yakuba*
GE24231	*Set8*	-	*Drosophila yakuba*
GE17555	*setd*	SETD	*Drosophila yakuba*
GE22225	*Smyd4-1*	SMYD	*Drosophila yakuba*
GE20125	*Smyd4-2*	SMYD	*Drosophila yakuba*
GE12361	*Smyd4-3*	SMYD	*Drosophila yakuba*
GE12406	*Smyd4-4*	SMYD	*Drosophila yakuba*
GE25007	*Smyd5*	SMYD	*Drosophila yakuba*
GE13567	*SmydA-1*	SmydA	*Drosophila yakuba*
GE22530	*SmydA-2*	SmydA	*Drosophila yakuba*
GE18493	*SmydA-3*	SmydA	*Drosophila yakuba*
GE15901	*SmydA-4*	SmydA	*Drosophila yakuba*
GE19428	*SmydA-5*	SmydA	*Drosophila yakuba*
GE11640	*SmydA-6*	SmydA	*Drosophila yakuba*
GE16967	*SmydA-8*	SmydA	*Drosophila yakuba*
GE15465	*SmydA-9*	SmydA	*Drosophila yakuba*
GE26397	*Su(var)3-9*	Suv	*Drosophila yakuba*
GE26056	*suv*	Suv	*Drosophila yakuba*
GE16999	*trr*	Trx	*Drosophila yakuba*
GE26460	*trx*	Trx	*Drosophila yakuba*
GE21994	*upSET(MLL5)*	-	*Drosophila yakuba*
Dnov03720	*ash1*	Ash	*Dufourea novaeangliae*
Dnov07772	*Blimp-1*	PRDM	*Dufourea novaeangliae*
Dnov01361	*egg*	Suv	*Dufourea novaeangliae*
Dnov12361	*Ez*	Ez	*Dufourea novaeangliae*
Dnov03119	*G9a*	Suv	*Dufourea novaeangliae*
Dnov12237	*Hmt4-20*	-	*Dufourea novaeangliae*
Dnov12368	*Mes-4*	Ash	*Dufourea novaeangliae*
Dnov10243	*Set1*	Trx	*Dufourea novaeangliae*
Dnov07842	*Set2*	Ash	*Dufourea novaeangliae*
Dnov04181	*Set8*	-	*Dufourea novaeangliae*
Dnov08929	*setd*	SETD	*Dufourea novaeangliae*
Dnov08926	*setd*	SETD	*Dufourea novaeangliae*

Dnov11007	*Smyd4-2*	SMYD	*Dufourea novaeangliae*
Dnov01731	*Smyd4-2*	SMYD	*Dufourea novaeangliae*
Dnov00419	*Smyd4-3*	SMYD	*Dufourea novaeangliae*
Dnov09445	*Smyd4-3*	SMYD	*Dufourea novaeangliae*
Dnov10364	*Smyd4-3*	SMYD	*Dufourea novaeangliae*
Dnov04775	*Smyd4-4*	SMYD	*Dufourea novaeangliae*
Dnov01975	*Smyd5*	SMYD	*Dufourea novaeangliae*
Dnov11385	*SmydA-1*	SmydA	*Dufourea novaeangliae*
Dnov05910	*SmydA-3*	SmydA	*Dufourea novaeangliae*
Dnov02443	*SmydA-5*	SmydA	*Dufourea novaeangliae*
Dnov05916	*SmydA-8*	SmydA	*Dufourea novaeangliae*
Dnov12047	*Su(var)3-9*	Suv	*Dufourea novaeangliae*
Dnov03047	*suv*	Suv	*Dufourea novaeangliae*
Dnov12588	*trr*	Trx	*Dufourea novaeangliae*
Dnov10256	*trx*	Trx	*Dufourea novaeangliae*
Dnov11710	*upSET(MLL5)*	-	*Dufourea novaeangliae*
EDAN016298	-	-	*Ephemera danica*
EDAN016560	-	-	*Ephemera danica*
EDAN017729	*Blimp-1*	PRDM	*Ephemera danica*
EDAN002594	*Ez*	Ez	*Ephemera danica*
EDAN015366	*G9a*	Suv	*Ephemera danica*
EDAN002811	*Hmt4-20*	-	*Ephemera danica*
EDAN010512	*Mes-4*	Ash	*Ephemera danica*
EDAN000752	*Set1*	Trx	*Ephemera danica*
EDAN006371	*Set2*	Ash	*Ephemera danica*
EDAN007195	*Set8*	-	*Ephemera danica*
EDAN019105	*Set8*	-	*Ephemera danica*
EDAN010686	*setd*	SETD	*Ephemera danica*
EDAN010505	*Smyd4-2*	SMYD	*Ephemera danica*
EDAN000998	*Smyd4-2*	SMYD	*Ephemera danica*
EDAN015875	*Smyd4-2*	SMYD	*Ephemera danica*
EDAN012149	*Smyd4-3*	SMYD	*Ephemera danica*
EDAN007659	*Smyd4-3*	SMYD	*Ephemera danica*
EDAN007660	*Smyd4-3*	SMYD	*Ephemera danica*
EDAN008466	*Smyd4-3*	SMYD	*Ephemera danica*
EDAN008467	*Smyd4-3*	SMYD	*Ephemera danica*
EDAN011068	*Smyd4-3*	SMYD	*Ephemera danica*
EDAN012148	*Smyd4-3*	SMYD	*Ephemera danica*
EDAN013890	*Smyd4-3*	SMYD	*Ephemera danica*
EDAN015693	*Smyd4-3*	SMYD	*Ephemera danica*
EDAN003791	*Smyd4-4*	SMYD	*Ephemera danica*
EDAN007661	*Smyd4-4*	SMYD	*Ephemera danica*
EDAN010840	*Smyd4-4*	SMYD	*Ephemera danica*
EDAN013130	*Smyd4-4*	SMYD	*Ephemera danica*

EDAN016991	*Smyd4-4*	SMYD	*Ephemera danica*
EDAN004206	*Smyd5*	SMYD	*Ephemera danica*
EDAN006746	*SmydA-1*	SmydA	*Ephemera danica*
EDAN007589	*SmydA-2*	SmydA	*Ephemera danica*
EDAN015154	*SmydA-2*	SmydA	*Ephemera danica*
EDAN006745	*SmydA-4*	SmydA	*Ephemera danica*
EDAN004899	*SmydA-4*	SmydA	*Ephemera danica*
EDAN008547	*SmydA-5*	SmydA	*Ephemera danica*
EDAN006919	*trx*	Trx	*Ephemera danica*
EDAN009703	*upSET(MLL5)*	-	*Ephemera danica*
Emex05073	*ash1*	Ash	*Eufriesea mexicana*
Emex04823	*Blimp-1*	PRDM	*Eufriesea mexicana*
Emex06112	*egg*	Suv	*Eufriesea mexicana*
Emex01995	*Ez*	Ez	*Eufriesea mexicana*
Emex06893	*G9a*	Suv	*Eufriesea mexicana*
Emex01515	*Hmt4-20*	-	*Eufriesea mexicana*
Emex01990	*Mes-4*	Ash	*Eufriesea mexicana*
Emex12982	*Set1*	Trx	*Eufriesea mexicana*
Emex04264	*Set2*	Ash	*Eufriesea mexicana*
Emex10641	*Set8*	-	*Eufriesea mexicana*
Emex03503	*setd*	SETD	*Eufriesea mexicana*
Emex03505	*setd*	SETD	*Eufriesea mexicana*
Emex09003	*Smyd4-2*	SMYD	*Eufriesea mexicana*
Emex07171	*Smyd4-3*	SMYD	*Eufriesea mexicana*
Emex01964	*Smyd4-3*	SMYD	*Eufriesea mexicana*
Emex02218	*Smyd4-3*	SMYD	*Eufriesea mexicana*
Emex04615	*Smyd4-4*	SMYD	*Eufriesea mexicana*
Emex11447	*Smyd5*	SMYD	*Eufriesea mexicana*
Emex11626	*SmydA-1*	SmydA	*Eufriesea mexicana*
Emex12315	*SmydA-3*	SmydA	*Eufriesea mexicana*
Emex12316	*SmydA-4*	SmydA	*Eufriesea mexicana*
Emex03889	*SmydA-5*	SmydA	*Eufriesea mexicana*
Emex07597	*SmydA-8*	SmydA	*Eufriesea mexicana*
Emex07120	*Su(var)3-9*	Suv	*Eufriesea mexicana*
Emex06408	*suv*	Suv	*Eufriesea mexicana*
Emex12886	*trr*	Trx	*Eufriesea mexicana*
Emex06429	*trx*	Trx	*Eufriesea mexicana*
Emex10451	*upSET(MLL5)*	-	*Eufriesea mexicana*
EAFF003172	-	-	*Eurytemora affinis*
EAFF012068	-	-	*Eurytemora affinis*
EAFF020546	-	-	*Eurytemora affinis*
EAFF024985	-	-	*Eurytemora affinis*
EAFF026995	-	-	*Eurytemora affinis*
EAFF001510	-	-	*Eurytemora affinis*

EAFF004620	-	-	*Eurytemora affinis*
EAFF014203	-	-	*Eurytemora affinis*
EAFF022901	-	-	*Eurytemora affinis*
EAFF023714	-	-	*Eurytemora affinis*
EAFF024293	-	-	*Eurytemora affinis*
EAFF028510	-	-	*Eurytemora affinis*
EAFF026430	*ash1*	Ash	*Eurytemora affinis*
EAFF004919	*egg*	Suv	*Eurytemora affinis*
EAFF026919	*Ez*	Ez	*Eurytemora affinis*
EAFF023801	*Ez*	Ez	*Eurytemora affinis*
EAFF019469	*G9a*	Suv	*Eurytemora affinis*
EAFF005171	*Hmt4-20*	-	*Eurytemora affinis*
EAFF015538	*Mes-4*	Ash	*Eurytemora affinis*
EAFF001212	*Set1*	Trx	*Eurytemora affinis*
EAFF023546	*Set2*	Ash	*Eurytemora affinis*
EAFF001410	*Set8*	-	*Eurytemora affinis*
EAFF023406	*Set8*	-	*Eurytemora affinis*
EAFF006688	*setd*	SETD	*Eurytemora affinis*
EAFF026654	*Smyd3*	SMYD	*Eurytemora affinis*
EAFF010233	*Smyd4-2*	SMYD	*Eurytemora affinis*
EAFF016439	*Smyd4-3*	SMYD	*Eurytemora affinis*
EAFF004118	*Smyd4-3*	SMYD	*Eurytemora affinis*
EAFF006548	*Smyd4-3*	SMYD	*Eurytemora affinis*
EAFF009472	*Smyd4-3*	SMYD	*Eurytemora affinis*
EAFF011278	*Smyd4-3*	SMYD	*Eurytemora affinis*
EAFF011550	*Smyd4-3*	SMYD	*Eurytemora affinis*
EAFF011786	*Smyd4-3*	SMYD	*Eurytemora affinis*
EAFF012755	*Smyd4-3*	SMYD	*Eurytemora affinis*
EAFF017677	*Smyd4-3*	SMYD	*Eurytemora affinis*
EAFF018167	*Smyd4-3*	SMYD	*Eurytemora affinis*
EAFF019388	*Smyd4-3*	SMYD	*Eurytemora affinis*
EAFF021221	*Smyd4-3*	SMYD	*Eurytemora affinis*
EAFF022204	*Smyd4-3*	SMYD	*Eurytemora affinis*
EAFF023364	*Smyd4-3*	SMYD	*Eurytemora affinis*
EAFF025830	*Smyd4-3*	SMYD	*Eurytemora affinis*
EAFF026138	*Smyd4-3*	SMYD	*Eurytemora affinis*
EAFF018754	*Smyd5*	SMYD	*Eurytemora affinis*
EAFF001987	*SmydA-1*	SmydA	*Eurytemora affinis*
EAFF009709	*SmydA-1*	SmydA	*Eurytemora affinis*
EAFF027622	*SmydA-1*	SmydA	*Eurytemora affinis*
EAFF010234	*SmydA-2*	SmydA	*Eurytemora affinis*
EAFF013875	*SmydA-2*	SmydA	*Eurytemora affinis*
EAFF018214	*SmydA-2*	SmydA	*Eurytemora affinis*
EAFF024128	*SmydA-3*	SmydA	*Eurytemora affinis*

EAFF025544	*SmydA-3*	SmydA	*Eurytemora affinis*
EAFF016441	*SmydA-4*	SmydA	*Eurytemora affinis*
EAFF014843	*SmydA-4*	SmydA	*Eurytemora affinis*
EAFF025365	*SmydA-4*	SmydA	*Eurytemora affinis*
EAFF026939	*SmydA-4*	SmydA	*Eurytemora affinis*
EAFF009720	*SmydA-8*	SmydA	*Eurytemora affinis*
EAFF018143	*SmydA-9*	SmydA	*Eurytemora affinis*
EAFF024909	*SmydA-9*	SmydA	*Eurytemora affinis*
EAFF023098	*Su(var)3-9*	Suv	*Eurytemora affinis*
EAFF017106	*trr*	Trx	*Eurytemora affinis*
EAFF025797	*trr*	Trx	*Eurytemora affinis*
EAFF024134	*trx*	Trx	*Eurytemora affinis*
XP_011299370.1	-	-	*Fopius arisanus*
XP_011314430.1	*ash1*	Ash	*Fopius arisanus*
XP_011300368.1	*Blimp-1*	PRDM	*Fopius arisanus*
XP_011305877.1	*egg*	Suv	*Fopius arisanus*
XP_011301065.1	*Ez*	Ez	*Fopius arisanus*
XP_011300240.1	*G9a*	Suv	*Fopius arisanus*
XP_011297760.1	*Hmt4-20*	-	*Fopius arisanus*
XP_011300581.1	*Mes-4*	Ash	*Fopius arisanus*
XP_011304506.1	*Set2*	Ash	*Fopius arisanus*
XP_011297550.1	*Set8*	-	*Fopius arisanus*
XP_011312838.1	*setd*	SETD	*Fopius arisanus*
XP_011312104.1	*Smyd4-1*	SMYD	*Fopius arisanus*
XP_011307080.1	*Smyd4-2*	SMYD	*Fopius arisanus*
XP_011300905.1	*Smyd4-2*	SMYD	*Fopius arisanus*
XP_011314323.1	*Smyd4-3*	SMYD	*Fopius arisanus*
XP_011304562.1	*Smyd4-3*	SMYD	*Fopius arisanus*
XP_011311122.1	*Smyd4-3*	SMYD	*Fopius arisanus*
XP_011297539.1	*Smyd4-4*	SMYD	*Fopius arisanus*
XP_011304229.1	*Smyd4-4*	SMYD	*Fopius arisanus*
XP_011304682.1	*Smyd5*	SMYD	*Fopius arisanus*
XP_011312778.1	*SmydA-1*	SmydA	*Fopius arisanus*
XP_011302347.1	*SmydA-3*	SmydA	*Fopius arisanus*
XP_011307379.1	*SmydA-5*	SmydA	*Fopius arisanus*
XP_011302346.1	*SmydA-6*	SmydA	*Fopius arisanus*
XP_011306058.1	*SmydA-8*	SmydA	*Fopius arisanus*
XP_011309925.1	*Su(var)3-9*	Suv	*Fopius arisanus*
XP_011306771.1	*suv*	Suv	*Fopius arisanus*
XP_011298309.1	*trx*	Trx	*Fopius arisanus*
XP_011297554.1	*upSET(MLL5)*	-	*Fopius arisanus*
FOCC003770	-	-	*Frankliniella occidentalis*
FOCC010071	-	-	*Frankliniella occidentalis*
FOCC010072	-	-	*Frankliniella occidentalis*

FOCC010076	-	-	*Frankliniella occidentalis*
FOCC010077	-	-	*Frankliniella occidentalis*
FOCC010073	-	-	*Frankliniella occidentalis*
FOCC013086	-	-	*Frankliniella occidentalis*
FOCC012844	-	-	*Frankliniella occidentalis*
FOCC007955	*ash1*	Ash	*Frankliniella occidentalis*
FOCC002283	*Blimp-1*	PRDM	*Frankliniella occidentalis*
FOCC006171	*Blimp-1*	PRDM	*Frankliniella occidentalis*
FOCC008985	*Blimp-1*	PRDM	*Frankliniella occidentalis*
FOCC010349	*Blimp-1*	PRDM	*Frankliniella occidentalis*
FOCC010364	*Blimp-1*	PRDM	*Frankliniella occidentalis*
FOCC011749	*Blimp-1*	PRDM	*Frankliniella occidentalis*
FOCC013507	*Blimp-1*	PRDM	*Frankliniella occidentalis*
FOCC016863	*Blimp-1*	PRDM	*Frankliniella occidentalis*
FOCC008815	*egg*	Suv	*Frankliniella occidentalis*
FOCC008607	*Ez*	Ez	*Frankliniella occidentalis*
FOCC016801	*G9a*	Suv	*Frankliniella occidentalis*
FOCC002018	*Hmt4-20*	-	*Frankliniella occidentalis*
FOCC001774	*Mes-4*	Ash	*Frankliniella occidentalis*
FOCC010613	*Set1*	Trx	*Frankliniella occidentalis*
FOCC009751	*Set2*	Ash	*Frankliniella occidentalis*
FOCC009082	*Set8*	-	*Frankliniella occidentalis*
FOCC015943	*Set8*	-	*Frankliniella occidentalis*
FOCC016014	*Set8*	-	*Frankliniella occidentalis*
FOCC016329	*Set8*	-	*Frankliniella occidentalis*
FOCC001761	*setd*	SETD	*Frankliniella occidentalis*
FOCC001759	*setd*	SETD	*Frankliniella occidentalis*
FOCC017034	*Smyd4-2*	SMYD	*Frankliniella occidentalis*
FOCC016997	*Smyd4-2*	SMYD	*Frankliniella occidentalis*
FOCC005340	*Smyd4-3*	SMYD	*Frankliniella occidentalis*
FOCC005014	*Smyd4-3*	SMYD	*Frankliniella occidentalis*
FOCC007055	*Smyd4-3*	SMYD	*Frankliniella occidentalis*
FOCC009401	*Smyd5*	SMYD	*Frankliniella occidentalis*
FOCC010914	*SmydA-1*	SmydA	*Frankliniella occidentalis*
FOCC013277	*SmydA-2*	SmydA	*Frankliniella occidentalis*
FOCC013364	*SmydA-2*	SmydA	*Frankliniella occidentalis*
FOCC016039	*SmydA-2*	SmydA	*Frankliniella occidentalis*
FOCC016968	*SmydA-3*	SmydA	*Frankliniella occidentalis*
FOCC011484	*SmydA-4*	SmydA	*Frankliniella occidentalis*
FOCC017528	*SmydA-8*	SmydA	*Frankliniella occidentalis*
FOCC002677	*Su(var)3-9*	Suv	*Frankliniella occidentalis*
FOCC005471	*Su(var)3-9*	Suv	*Frankliniella occidentalis*
FOCC017059	*suv*	Suv	*Frankliniella occidentalis*
FOCC010246	*trr*	Trx	*Frankliniella occidentalis*

FOCC004505	*trx*	Trx	*Frankliniella occidentalis*
FOCC008579	*upSET(MLL5)*	-	*Frankliniella occidentalis*
GBUE009708	*ash1*	Ash	*Gerris buenoi*
GBUE018405	*Blimp-1*	PRDM	*Gerris buenoi*
GBUE008804	*egg*	Suv	*Gerris buenoi*
GBUE017226	*Ez*	Ez	*Gerris buenoi*
GBUE005309	*G9a*	Suv	*Gerris buenoi*
GBUE006374	*Hmt4-20*	-	*Gerris buenoi*
GBUE012764	*Mes-4*	Ash	*Gerris buenoi*
GBUE019960	*Set1*	Trx	*Gerris buenoi*
GBUE000965	*Set2*	Ash	*Gerris buenoi*
GBUE013626	*Set8*	-	*Gerris buenoi*
GBUE010864	*setd*	SETD	*Gerris buenoi*
GBUE006756	*Smyd3*	SMYD	*Gerris buenoi*
GBUE017035	*Smyd4-1*	SMYD	*Gerris buenoi*
GBUE018953	*Smyd4-2*	SMYD	*Gerris buenoi*
GBUE003862	*Smyd4-3*	SMYD	*Gerris buenoi*
GBUE009762	*Smyd4-3*	SMYD	*Gerris buenoi*
GBUE004495	*Smyd5*	SMYD	*Gerris buenoi*
GBUE000004	*SmydA-1*	SmydA	*Gerris buenoi*
GBUE000005	*SmydA-4*	SmydA	*Gerris buenoi*
GBUE013250	*SmydA-5*	SmydA	*Gerris buenoi*
GBUE017346	*SmydA-8*	SmydA	*Gerris buenoi*
GBUE017347	*SmydA-9*	SmydA	*Gerris buenoi*
GBUE007007	*Su(var)3-9*	Suv	*Gerris buenoi*
GBUE002656	*trr*	Trx	*Gerris buenoi*
GBUE010721	*trx*	Trx	*Gerris buenoi*
GBUE006188	*upSET(MLL5)*	-	*Gerris buenoi*
GAUT000965	-	-	*Glossina austeni*
GAUT039134	-	-	*Glossina austeni*
GAUT003569	*ash1*	Ash	*Glossina austeni*
GAUT037360	*Blimp-1*	PRDM	*Glossina austeni*
GAUT051764	*Ez*	Ez	*Glossina austeni*
GAUT014335	*G9a*	Suv	*Glossina austeni*
GAUT021127	*Hmt4-20*	-	*Glossina austeni*
GAUT007110	*Mes-4*	Ash	*Glossina austeni*
GAUT010050	*Set1*	Trx	*Glossina austeni*
GAUT042724	*Set2*	Ash	*Glossina austeni*
GAUT051725	*Set8*	-	*Glossina austeni*
GAUT008299	*setd*	SETD	*Glossina austeni*
GAUT003063	*Smyd4-1*	SMYD	*Glossina austeni*
GAUT015030	*Smyd4-2*	SMYD	*Glossina austeni*
GAUT006445	*Smyd4-3*	SMYD	*Glossina austeni*
GAUT005982	*Smyd5*	SMYD	*Glossina austeni*

GAUT028521	*SmydA-1*	SmydA	*Glossina austeni*
GAUT003878	*SmydA-2*	SmydA	*Glossina austeni*
GAUT012548	*SmydA-3*	SmydA	*Glossina austeni*
GAUT050092	*SmydA-4*	SmydA	*Glossina austeni*
GAUT023068	*SmydA-5*	SmydA	*Glossina austeni*
GAUT015085	*SmydA-6*	SmydA	*Glossina austeni*
GAUT020408	*SmydA-6*	SmydA	*Glossina austeni*
GAUT015280	*SmydA-8*	SmydA	*Glossina austeni*
GAUT015283	*SmydA-8*	SmydA	*Glossina austeni*
GAUT015281	*SmydA-9*	SmydA	*Glossina austeni*
GAUT002773	*suv*	Suv	*Glossina austeni*
GAUT039876	*trr*	Trx	*Glossina austeni*
GAUT028766	*trx*	Trx	*Glossina austeni*
GBRI006832	-	-	*Glossina brevipalpis*
GBRI008909	-	-	*Glossina brevipalpis*
GBRI030513	*ash1*	Ash	*Glossina brevipalpis*
GBRI006374	*Blimp-1*	PRDM	*Glossina brevipalpis*
GBRI044332	*egg*	Suv	*Glossina brevipalpis*
GBRI031107	*Ez*	Ez	*Glossina brevipalpis*
GBRI045079	*G9a*	Suv	*Glossina brevipalpis*
GBRI014412	*Hmt4-20*	-	*Glossina brevipalpis*
GBRI036913	*Mes-4*	Ash	*Glossina brevipalpis*
GBRI009791	*Set1*	Trx	*Glossina brevipalpis*
GBRI021201	*Set2*	Ash	*Glossina brevipalpis*
GBRI044844	*Set2*	Ash	*Glossina brevipalpis*
GBRI021014	*Set8*	-	*Glossina brevipalpis*
GBRI007875	*setd*	SETD	*Glossina brevipalpis*
GBRI009726	*Smyd4-1*	SMYD	*Glossina brevipalpis*
GBRI029322	*Smyd4-2*	SMYD	*Glossina brevipalpis*
GBRI026790	*Smyd4-3*	SMYD	*Glossina brevipalpis*
GBRI029821	*Smyd5*	SMYD	*Glossina brevipalpis*
GBRI025958	*SmydA-1*	SmydA	*Glossina brevipalpis*
GBRI012014	*SmydA-2*	SmydA	*Glossina brevipalpis*
GBRI026624	*SmydA-3*	SmydA	*Glossina brevipalpis*
GBRI033680	*SmydA-4*	SmydA	*Glossina brevipalpis*
GBRI005440	*SmydA-6*	SmydA	*Glossina brevipalpis*
GBRI043141	*SmydA-6*	SmydA	*Glossina brevipalpis*
GBRI044001	*SmydA-8*	SmydA	*Glossina brevipalpis*
GBRI044006	*SmydA-8*	SmydA	*Glossina brevipalpis*
GBRI044002	*SmydA-9*	SmydA	*Glossina brevipalpis*
GBRI043534	*Su(var)3-9*	Suv	*Glossina brevipalpis*
GBRI014352	*Su(var)3-9*	Suv	*Glossina brevipalpis*
GBRI031809	*suv*	Suv	*Glossina brevipalpis*
GBRI003992	*trr*	Trx	*Glossina brevipalpis*

GBRI019428	*trx*	Trx	*Glossina brevipalpis*
GBRI030568	*upSET(MLL5)*	-	*Glossina brevipalpis*
GFUI032853	-	-	*Glossina fuscipes*
GFUI022615	-	-	*Glossina fuscipes*
GFUI025617	*ash1*	Ash	*Glossina fuscipes*
GFUI020100	*Blimp-1*	PRDM	*Glossina fuscipes*
GFUI017483	*egg*	Suv	*Glossina fuscipes*
GFUI014956	*G9a*	Suv	*Glossina fuscipes*
GFUI051362	*Hmt4-20*	-	*Glossina fuscipes*
GFUI009308	*Mes-4*	Ash	*Glossina fuscipes*
GFUI010890	*Set1*	Trx	*Glossina fuscipes*
GFUI024474	*Set2*	Ash	*Glossina fuscipes*
GFUI035687	*Set8*	-	*Glossina fuscipes*
GFUI020620	*setd*	SETD	*Glossina fuscipes*
GFUI046686	*Smyd4-1*	SMYD	*Glossina fuscipes*
GFUI031085	*Smyd4-1*	SMYD	*Glossina fuscipes*
GFUI002338	*Smyd4-2*	SMYD	*Glossina fuscipes*
GFUI050271	*Smyd4-3*	SMYD	*Glossina fuscipes*
GFUI000464	*Smyd5*	SMYD	*Glossina fuscipes*
GFUI017459	*SmydA-1*	SmydA	*Glossina fuscipes*
GFUI044864	*SmydA-2*	SmydA	*Glossina fuscipes*
GFUI023273	*SmydA-3*	SmydA	*Glossina fuscipes*
GFUI051070	*SmydA-4*	SmydA	*Glossina fuscipes*
GFUI011622	*SmydA-5*	SmydA	*Glossina fuscipes*
GFUI043484	*SmydA-6*	SmydA	*Glossina fuscipes*
GFUI026980	*SmydA-6*	SmydA	*Glossina fuscipes*
GFUI013391	*SmydA-8*	SmydA	*Glossina fuscipes*
GFUI013397	*SmydA-8*	SmydA	*Glossina fuscipes*
GFUI013396	*SmydA-9*	SmydA	*Glossina fuscipes*
GFUI002253	*Su(var)3-9*	Suv	*Glossina fuscipes*
GFUI033386	*suv*	Suv	*Glossina fuscipes*
GFUI052279	*suv*	Suv	*Glossina fuscipes*
GFUI003837	*trr*	Trx	*Glossina fuscipes*
GFUI033201	*trr*	Trx	*Glossina fuscipes*
GFUI040605	*trx*	Trx	*Glossina fuscipes*
GFUI009588	*upSET(MLL5)*	-	*Glossina fuscipes*
GMOY002463	-	-	*Glossina morsitans*
GMOY005928	*ash1*	Ash	*Glossina morsitans*
GMOY001750	*Blimp-1*	PRDM	*Glossina morsitans*
GMOY012045	*egg*	Suv	*Glossina morsitans*
GMOY001127	*Ez*	Ez	*Glossina morsitans*
GMOY006982	*G9a*	Suv	*Glossina morsitans*
GMOY000051	*Hmt4-20*	-	*Glossina morsitans*
GMOY006253	*Mes-4*	Ash	*Glossina morsitans*

GMOY007151	*Set1*	Trx	*Glossina morsitans*
GMOY010695	*Set2*	Ash	*Glossina morsitans*
GMOY005450	*Set8*	-	*Glossina morsitans*
GMOY005462	*setd*	SETD	*Glossina morsitans*
GMOY000548	*Smyd4-1*	SMYD	*Glossina morsitans*
GMOY003979	*Smyd4-2*	SMYD	*Glossina morsitans*
GMOY007161	*Smyd4-3*	SMYD	*Glossina morsitans*
GMOY003547	*Smyd5*	SMYD	*Glossina morsitans*
GMOY007823	*SmydA-2*	SmydA	*Glossina morsitans*
GMOY003089	*SmydA-3*	SmydA	*Glossina morsitans*
GMOY001698	*SmydA-4*	SmydA	*Glossina morsitans*
GMOY009115	*SmydA-5*	SmydA	*Glossina morsitans*
GMOY007184	*SmydA-6*	SmydA	*Glossina morsitans*
GMOY011379	*SmydA-6*	SmydA	*Glossina morsitans*
GMOY004217	*SmydA-8*	SmydA	*Glossina morsitans*
GMOY004216	*SmydA-9*	SmydA	*Glossina morsitans*
GMOY011471	*Su(var)3-9*	Suv	*Glossina morsitans*
GMOY009260	*Su(var)3-9*	Suv	*Glossina morsitans*
GMOY004169	*trr*	Trx	*Glossina morsitans*
GMOY002816	*trx*	Trx	*Glossina morsitans*
GMOY004407	*upSET(MLL5)*	-	*Glossina morsitans*
GPAI006159	-	-	*Glossina pallidipes*
GPAI017683	*ash1*	Ash	*Glossina pallidipes*
GPAI007592	*Blimp-1*	PRDM	*Glossina pallidipes*
GPAI043842	*egg*	Suv	*Glossina pallidipes*
GPAI005269	*G9a*	Suv	*Glossina pallidipes*
GPAI046219	*Hmt4-20*	-	*Glossina pallidipes*
GPAI007879	*Mes-4*	Ash	*Glossina pallidipes*
GPAI039142	*Set1*	Trx	*Glossina pallidipes*
GPAI036866	*Set2*	Ash	*Glossina pallidipes*
GPAI045113	*Set8*	-	*Glossina pallidipes*
GPAI040696	*setd*	SETD	*Glossina pallidipes*
GPAI045330	*Smyd4-1*	SMYD	*Glossina pallidipes*
GPAI013502	*Smyd4-2*	SMYD	*Glossina pallidipes*
GPAI038323	*Smyd4-3*	SMYD	*Glossina pallidipes*
GPAI032096	*Smyd4-4*	SMYD	*Glossina pallidipes*
GPAI010418	*Smyd5*	SMYD	*Glossina pallidipes*
GPAI049087	*SmydA-1*	SmydA	*Glossina pallidipes*
GPAI025288	*SmydA-2*	SmydA	*Glossina pallidipes*
GPAI033687	*SmydA-3*	SmydA	*Glossina pallidipes*
GPAI031397	*SmydA-4*	SmydA	*Glossina pallidipes*
GPAI044832	*SmydA-5*	SmydA	*Glossina pallidipes*
GPAI015004	*SmydA-6*	SmydA	*Glossina pallidipes*
GPAI040524	*SmydA-6*	SmydA	*Glossina pallidipes*

GPAI009323	*SmydA-8*	SmydA	*Glossina pallidipes*
GPAI009327	*SmydA-8*	SmydA	*Glossina pallidipes*
GPAI009335	*SmydA-9*	SmydA	*Glossina pallidipes*
GPAI047623	*Su(var)3-9*	Suv	*Glossina pallidipes*
GPAI035612	*suv*	Suv	*Glossina pallidipes*
GPAI026359	*trr*	Trx	*Glossina pallidipes*
GPAI030239	*trx*	Trx	*Glossina pallidipes*
GPAI017716	*upSET(MLL5)*	-	*Glossina pallidipes*
GPPI011201	-	-	*Glossina palpalis*
GPPI022844	-	-	*Glossina palpalis*
GPPI035533	*ash1*	Ash	*Glossina palpalis*
GPPI031332	*Blimp-1*	PRDM	*Glossina palpalis*
GPPI038391	*egg*	Suv	*Glossina palpalis*
GPPI023005	*Ez*	Ez	*Glossina palpalis*
GPPI020075	*G9a*	Suv	*Glossina palpalis*
GPPI027364	*Mes-4*	Ash	*Glossina palpalis*
GPPI023174	*Set1*	Trx	*Glossina palpalis*
GPPI024774	*Set2*	Ash	*Glossina palpalis*
GPPI020621	*Set8*	-	*Glossina palpalis*
GPPI042244	*setd*	SETD	*Glossina palpalis*
GPPI046802	*Smyd4-1*	SMYD	*Glossina palpalis*
GPPI040565	*Smyd4-1*	SMYD	*Glossina palpalis*
GPPI039669	*Smyd4-2*	SMYD	*Glossina palpalis*
GPPI031007	*Smyd4-3*	SMYD	*Glossina palpalis*
GPPI035949	*Smyd5*	SMYD	*Glossina palpalis*
GPPI001551	*SmydA-1*	SmydA	*Glossina palpalis*
GPPI020866	*SmydA-2*	SmydA	*Glossina palpalis*
GPPI003369	*SmydA-3*	SmydA	*Glossina palpalis*
GPPI012558	*SmydA-4*	SmydA	*Glossina palpalis*
GPPI016194	*SmydA-5*	SmydA	*Glossina palpalis*
GPPI041869	*SmydA-6*	SmydA	*Glossina palpalis*
GPPI044611	*SmydA-6*	SmydA	*Glossina palpalis*
GPPI000369	*SmydA-8*	SmydA	*Glossina palpalis*
GPPI001276	*SmydA-8*	SmydA	*Glossina palpalis*
GPPI051160	*SmydA-9*	SmydA	*Glossina palpalis*
GPPI028098	*Su(var)3-9*	Suv	*Glossina palpalis*
GPPI047825	*suv*	Suv	*Glossina palpalis*
GPPI037952	*trr*	Trx	*Glossina palpalis*
GPPI040878	*trx*	Trx	*Glossina palpalis*
Hlab06559	*ash1*	Ash	*Habropoda laboriosa*
Hlab06779	*Blimp-1*	PRDM	*Habropoda laboriosa*
Hlab05635	*egg*	Suv	*Habropoda laboriosa*
Hlab13037	*Ez*	Ez	*Habropoda laboriosa*
Hlab12868	*G9a*	Suv	*Habropoda laboriosa*

Hlab11520	*Hmt4-20*	-	*Habropoda laboriosa*
Hlab01507	*Mes-4*	Ash	*Habropoda laboriosa*
Hlab06030	*Set1*	Trx	*Habropoda laboriosa*
Hlab07675	*Set2*	Ash	*Habropoda laboriosa*
Hlab10749	*Set8*	-	*Habropoda laboriosa*
Hlab08772	*setd*	SETD	*Habropoda laboriosa*
Hlab08770	*setd*	SETD	*Habropoda laboriosa*
Hlab10869	*Smyd4-2*	SMYD	*Habropoda laboriosa*
Hlab03265	*Smyd4-2*	SMYD	*Habropoda laboriosa*
Hlab02978	*Smyd4-3*	SMYD	*Habropoda laboriosa*
Hlab06401	*Smyd4-3*	SMYD	*Habropoda laboriosa*
Hlab10576	*Smyd4-3*	SMYD	*Habropoda laboriosa*
Hlab03516	*Smyd4-4*	SMYD	*Habropoda laboriosa*
Hlab08038	*Smyd4-4*	SMYD	*Habropoda laboriosa*
Hlab11342	*Smyd5*	SMYD	*Habropoda laboriosa*
Hlab11165	*SmydA-1*	SmydA	*Habropoda laboriosa*
Hlab13506	*SmydA-3*	SmydA	*Habropoda laboriosa*
Hlab13511	*SmydA-4*	SmydA	*Habropoda laboriosa*
Hlab02530	*SmydA-5*	SmydA	*Habropoda laboriosa*
Hlab11466	*SmydA-8*	SmydA	*Habropoda laboriosa*
Hlab05757	*Su(var)3-9*	Suv	*Habropoda laboriosa*
Hlab05767	*suv*	Suv	*Habropoda laboriosa*
Hlab13473	*trr*	Trx	*Habropoda laboriosa*
Hlab13391	*trx*	Trx	*Habropoda laboriosa*
Hlab11190	*upSET(MLL5)*	-	*Habropoda laboriosa*
HHAL006761	-	-	*Halyomorpha halys*
HHAL002221	*Blimp-1*	PRDM	*Halyomorpha halys*
HHAL006241	*egg*	Suv	*Halyomorpha halys*
HHAL006240	*egg*	Suv	*Halyomorpha halys*
HHAL005772	*Ez*	Ez	*Halyomorpha halys*
HHAL006963	*G9a*	Suv	*Halyomorpha halys*
HHAL009894	*Hmt4-20*	-	*Halyomorpha halys*
HHAL000815	*Mes-4*	Ash	*Halyomorpha halys*
HHAL005878	*Set1*	Trx	*Halyomorpha halys*
HHAL006642	*Set2*	Ash	*Halyomorpha halys*
HHAL003401	*Set8*	-	*Halyomorpha halys*
HHAL007456	*setd*	SETD	*Halyomorpha halys*
HHAL009003	*Smyd3*	SMYD	*Halyomorpha halys*
HHAL000090	*Smyd4-3*	SMYD	*Halyomorpha halys*
HHAL004593	*Smyd4-3*	SMYD	*Halyomorpha halys*
HHAL003302	*Smyd4-4*	SMYD	*Halyomorpha halys*
HHAL001411	*Smyd5*	SMYD	*Halyomorpha halys*
HHAL000853	*SmydA-1*	SmydA	*Halyomorpha halys*
HHAL003743	*SmydA-3*	SmydA	*Halyomorpha halys*

HHAL000852	*SmydA-4*	SmydA	*Halyomorpha halys*
HHAL007047	*SmydA-5*	SmydA	*Halyomorpha halys*
HHAL002785	*SmydA-8*	SmydA	*Halyomorpha halys*
HHAL008217	*Su(var)3-9*	Suv	*Halyomorpha halys*
HHAL007301	*suv*	Suv	*Halyomorpha halys*
HHAL009337	*trr*	Trx	*Halyomorpha halys*
HHAL002662	*trx*	Trx	*Halyomorpha halys*
Hsal_10489	-	-	*Harpegnathos saltator*
Hsal_10054	*ash1*	Ash	*Harpegnathos saltator*
Hsal_05330	*Ez*	Ez	*Harpegnathos saltator*
Hsal_12111	*G9a*	Suv	*Harpegnathos saltator*
Hsal_07893	*Hmt4-20*	-	*Harpegnathos saltator*
Hsal_01244	*Set1*	Trx	*Harpegnathos saltator*
Hsal_05593	*Set8*	-	*Harpegnathos saltator*
Hsal_06751	*setd*	SETD	*Harpegnathos saltator*
Hsal_06753	*setd*	SETD	*Harpegnathos saltator*
Hsal_07051	*Smyd4-2*	SMYD	*Harpegnathos saltator*
Hsal_11656	*Smyd4-2*	SMYD	*Harpegnathos saltator*
Hsal_08142	*Smyd4-3*	SMYD	*Harpegnathos saltator*
Hsal_00516	*Smyd4-3*	SMYD	*Harpegnathos saltator*
Hsal_10487	*Smyd4-3*	SMYD	*Harpegnathos saltator*
Hsal_10488	*Smyd4-3*	SMYD	*Harpegnathos saltator*
Hsal_10252	*Smyd4-4*	SMYD	*Harpegnathos saltator*
Hsal_14332	*Smyd5*	SMYD	*Harpegnathos saltator*
Hsal_03432	*SmydA-3*	SmydA	*Harpegnathos saltator*
Hsal_03431	*SmydA-4*	SmydA	*Harpegnathos saltator*
Hsal_08938	*SmydA-5*	SmydA	*Harpegnathos saltator*
Hsal_14941	*SmydA-8*	SmydA	*Harpegnathos saltator*
Hsal_14066	*suv*	Suv	*Harpegnathos saltator*
Hsal_08754	*upSET(MLL5)*	-	*Harpegnathos saltator*
HMEL007072	*Blimp-1*	PRDM	*Heliconius melpomene*
HMEL014564	*egg*	Suv	*Heliconius melpomene*
HMEL005664	*Ez*	Ez	*Heliconius melpomene*
HMEL015424	*G9a*	Suv	*Heliconius melpomene*
HMEL017733	*Hmt4-20*	-	*Heliconius melpomene*
HMEL016949	*Mes-4*	Ash	*Heliconius melpomene*
HMEL006851	*Set1*	Trx	*Heliconius melpomene*
HMEL017250	*Set2*	Ash	*Heliconius melpomene*
HMEL006860	*Set8*	-	*Heliconius melpomene*
HMEL015094	*Smyd3*	SMYD	*Heliconius melpomene*
HMEL007274	*Smyd4-1*	SMYD	*Heliconius melpomene*
HMEL010330	*Smyd4-3*	SMYD	*Heliconius melpomene*
HMEL003425	*Smyd4-4*	SMYD	*Heliconius melpomene*
HMEL016221	*Smyd5*	SMYD	*Heliconius melpomene*

HMEL008018	*SmydA-2*	SmydA	*Heliconius melpomene*
HMEL005185	*SmydA-4*	SmydA	*Heliconius melpomene*
HMEL017192	*SmydA-8*	SmydA	*Heliconius melpomene*
HMEL006496	*Su(var)3-9*	Suv	*Heliconius melpomene*
HMEL003313	*suv*	Suv	*Heliconius melpomene*
HMEL004761	*trr*	Trx	*Heliconius melpomene*
HMEL013536	*trx*	Trx	*Heliconius melpomene*
HMEL016751	*upSET(MLL5)*	-	*Heliconius melpomene*
HVIT000975	*Blimp-1*	PRDM	*Homalodisca vitripennis*
HVIT015263	*Blimp-1*	PRDM	*Homalodisca vitripennis*
HVIT026928	*egg*	Suv	*Homalodisca vitripennis*
HVIT032230	*egg*	Suv	*Homalodisca vitripennis*
HVIT002875	*Ez*	Ez	*Homalodisca vitripennis*
HVIT004712	*G9a*	Suv	*Homalodisca vitripennis*
HVIT021329	*Hmt4-20*	-	*Homalodisca vitripennis*
HVIT010716	*Mes-4*	Ash	*Homalodisca vitripennis*
HVIT010088	*Set1*	Trx	*Homalodisca vitripennis*
HVIT015244	*Set1*	Trx	*Homalodisca vitripennis*
HVIT015536	*Set2*	Ash	*Homalodisca vitripennis*
HVIT016239	*Set8*	-	*Homalodisca vitripennis*
HVIT026895	*setd*	SETD	*Homalodisca vitripennis*
HVIT026896	*setd*	SETD	*Homalodisca vitripennis*
HVIT001096	*Smyd4-2*	SMYD	*Homalodisca vitripennis*
HVIT000155	*Smyd4-3*	SMYD	*Homalodisca vitripennis*
HVIT007220	*Smyd4-3*	SMYD	*Homalodisca vitripennis*
HVIT022921	*Smyd4-3*	SMYD	*Homalodisca vitripennis*
HVIT003655	*Smyd5*	SMYD	*Homalodisca vitripennis*
HVIT005436	*SmydA-1*	SmydA	*Homalodisca vitripennis*
HVIT004981	*SmydA-2*	SmydA	*Homalodisca vitripennis*
HVIT019774	*SmydA-2*	SmydA	*Homalodisca vitripennis*
HVIT005437	*SmydA-4*	SmydA	*Homalodisca vitripennis*
HVIT004968	*SmydA-5*	SmydA	*Homalodisca vitripennis*
HVIT021581	*SmydA-8*	SmydA	*Homalodisca vitripennis*
HVIT015553	*Su(var)3-9*	Suv	*Homalodisca vitripennis*
HVIT018813	*suv*	Suv	*Homalodisca vitripennis*
HVIT011515	*trr*	Trx	*Homalodisca vitripennis*
HVIT018408	*trx*	Trx	*Homalodisca vitripennis*
HAZT011949	*ash1*	Ash	*Hyalella azteca*
HAZT009026	*Blimp-1*	PRDM	*Hyalella azteca*
HAZT001775	*egg*	Suv	*Hyalella azteca*
HAZT005646	*Ez*	Ez	*Hyalella azteca*
HAZT000211	*Ez*	Ez	*Hyalella azteca*
HAZT012700	*G9a*	Suv	*Hyalella azteca*
HAZT002495	*Hmt4-20*	-	*Hyalella azteca*

HAZT012332	*Mes-4*	Ash	*Hyalella azteca*
HAZT002193	*Set1*	Trx	*Hyalella azteca*
HAZT001741	*Set2*	Ash	*Hyalella azteca*
HAZT003710	*Set8*	-	*Hyalella azteca*
HAZT003709	*Set8*	-	*Hyalella azteca*
HAZT005237	*Smyd4-3*	SMYD	*Hyalella azteca*
HAZT011103	*SmydA-2*	SmydA	*Hyalella azteca*
HAZT002301	*SmydA-2*	SmydA	*Hyalella azteca*
HAZT002302	*SmydA-2*	SmydA	*Hyalella azteca*
HAZT011434	*Su(var)3-9*	Suv	*Hyalella azteca*
HAZT011295	*trr*	Trx	*Hyalella azteca*
HAZT000729	*trx*	Trx	*Hyalella azteca*
HAZT011847	*trx*	Trx	*Hyalella azteca*
HAZT004622	*upSET(MLL5)*	-	*Hyalella azteca*
ISCW020125	-	-	*Ixodes scapularis*
ISCW005314	*ash1*	Ash	*Ixodes scapularis*
ISCW001711	*egg*	Suv	*Ixodes scapularis*
ISCW005550	*egg*	Suv	*Ixodes scapularis*
ISCW020151	*Ez*	Ez	*Ixodes scapularis*
ISCW019618	*G9a*	Suv	*Ixodes scapularis*
ISCW004542	*Mes-4*	Ash	*Ixodes scapularis*
ISCW009292	*Set1*	Trx	*Ixodes scapularis*
ISCW013039	*Set2*	Ash	*Ixodes scapularis*
ISCW020034	*Set8*	-	*Ixodes scapularis*
ISCW011867	*setd*	SETD	*Ixodes scapularis*
ISCW000409	*Smyd4-3*	SMYD	*Ixodes scapularis*
ISCW000706	*Smyd5*	SMYD	*Ixodes scapularis*
ISCW021420	*suv*	Suv	*Ixodes scapularis*
ISCW020985	*trx*	Trx	*Ixodes scapularis*
ISCW020685	*trx*	Trx	*Ixodes scapularis*
ISCW020048	*upSET(MLL5)*	-	*Ixodes scapularis*
LFUL019513	-	-	*Ladona fulva*
LFUL005820	*Blimp-1*	PRDM	*Ladona fulva*
LFUL018789	*Blimp-1*	PRDM	*Ladona fulva*
LFUL002871	*egg*	Suv	*Ladona fulva*
LFUL002215	*Ez*	Ez	*Ladona fulva*
LFUL012693	*G9a*	Suv	*Ladona fulva*
LFUL015879	*Hmt4-20*	-	*Ladona fulva*
LFUL016378	*Mes-4*	Ash	*Ladona fulva*
LFUL015175	*Set1*	Trx	*Ladona fulva*
LFUL000349	*Set2*	Ash	*Ladona fulva*
LFUL016302	*Set8*	-	*Ladona fulva*
LFUL004830	*setd*	SETD	*Ladona fulva*
LFUL004829	*setd*	SETD	*Ladona fulva*

LFUL017126	*Smyd4-2*	SMYD	*Ladona fulva*
LFUL005942	*Smyd4-3*	SMYD	*Ladona fulva*
LFUL003129	*Smyd5*	SMYD	*Ladona fulva*
LFUL012204	*SmydA-1*	SmydA	*Ladona fulva*
LFUL010707	*SmydA-2*	SmydA	*Ladona fulva*
LFUL017916	*SmydA-4*	SmydA	*Ladona fulva*
LFUL012672	*SmydA-4*	SmydA	*Ladona fulva*
LFUL010708	*SmydA-5*	SmydA	*Ladona fulva*
LFUL013113	*suv*	Suv	*Ladona fulva*
LFUL012798	*trr*	Trx	*Ladona fulva*
LFUL001593	*trx*	Trx	*Ladona fulva*
LFUL007711	*upSET(MLL5)*	-	*Ladona fulva*
Lalb_02950	*ash1*	Ash	*Lasioglossum albipes*
Lalb_08394	*Blimp-1*	PRDM	*Lasioglossum albipes*
Lalb_06607	*egg*	Suv	*Lasioglossum albipes*
Lalb_11002	*Ez*	Ez	*Lasioglossum albipes*
Lalb_10338	*G9a*	Suv	*Lasioglossum albipes*
Lalb_12269	*Hmt4-20*	-	*Lasioglossum albipes*
Lalb_07870	*Mes-4*	Ash	*Lasioglossum albipes*
Lalb_10938	*Set1*	Trx	*Lasioglossum albipes*
Lalb_03409	*Set2*	Ash	*Lasioglossum albipes*
Lalb_03480	*Set8*	-	*Lasioglossum albipes*
Lalb_08206	*setd*	SETD	*Lasioglossum albipes*
Lalb_03770	*Smyd4-1*	SMYD	*Lasioglossum albipes*
Lalb_06589	*Smyd4-2*	SMYD	*Lasioglossum albipes*
Lalb_08174	*Smyd4-2*	SMYD	*Lasioglossum albipes*
Lalb_11136	*Smyd4-2*	SMYD	*Lasioglossum albipes*
Lalb_10295	*Smyd4-3*	SMYD	*Lasioglossum albipes*
Lalb_13863	*Smyd4-3*	SMYD	*Lasioglossum albipes*
Lalb_10247	*Smyd4-4*	SMYD	*Lasioglossum albipes*
Lalb_03197	*Smyd4-4*	SMYD	*Lasioglossum albipes*
Lalb_10800	*Smyd5*	SMYD	*Lasioglossum albipes*
Lalb_11488	*SmydA-1*	SmydA	*Lasioglossum albipes*
Lalb_00738	*SmydA-3*	SmydA	*Lasioglossum albipes*
Lalb_00739	*SmydA-4*	SmydA	*Lasioglossum albipes*
Lalb_03057	*SmydA-5*	SmydA	*Lasioglossum albipes*
Lalb_06677	*SmydA-8*	SmydA	*Lasioglossum albipes*
Lalb_06260	*suv*	Suv	*Lasioglossum albipes*
Lalb_11951	*trr*	Trx	*Lasioglossum albipes*
Lalb_07949	*trx*	Trx	*Lasioglossum albipes*
Lalb_00628	*upSET(MLL5)*	-	*Lasioglossum albipes*
LHES001197	*Blimp-1*	PRDM	*Latrodectus hesperus*
LHES003642	*Blimp-1*	PRDM	*Latrodectus hesperus*
LHES003277	*egg*	Suv	*Latrodectus hesperus*

LHES001690	*Ez*	Ez	*Latrodectus hesperus*
LHES004109	*G9a*	Suv	*Latrodectus hesperus*
LHES000806	*Hmt4-20*	-	*Latrodectus hesperus*
LHES001458	*Hmt4-20*	-	*Latrodectus hesperus*
LHES011056	*Mes-4*	Ash	*Latrodectus hesperus*
LHES001912	*Mes-4*	Ash	*Latrodectus hesperus*
LHES003432	*Set1*	Trx	*Latrodectus hesperus*
LHES006360	*Set2*	Ash	*Latrodectus hesperus*
LHES006058	*Set8*	-	*Latrodectus hesperus*
LHES011089	*setd*	SETD	*Latrodectus hesperus*
LHES005367	*Smyd5*	SMYD	*Latrodectus hesperus*
LHES002320	*Su(var)3-9*	Suv	*Latrodectus hesperus*
LHES014148	*suv*	Suv	*Latrodectus hesperus*
LHES010899	*trr*	Trx	*Latrodectus hesperus*
LHES007730	*trx*	Trx	*Latrodectus hesperus*
LDEC014806	-	-	*Leptinotarsa decemlineata*
LDEC016729	*ash1*	Ash	*Leptinotarsa decemlineata*
LDEC016433	*Blimp-1*	PRDM	*Leptinotarsa decemlineata*
LDEC019415	*egg*	Suv	*Leptinotarsa decemlineata*
LDEC019416	*egg*	Suv	*Leptinotarsa decemlineata*
LDEC016953	*Ez*	Ez	*Leptinotarsa decemlineata*
LDEC018674	*G9a*	Suv	*Leptinotarsa decemlineata*
LDEC000081	*Hmt4-20*	-	*Leptinotarsa decemlineata*
LDEC003201	*Set1*	Trx	*Leptinotarsa decemlineata*
LDEC014818	*Set2*	Ash	*Leptinotarsa decemlineata*
LDEC002537	*Set8*	-	*Leptinotarsa decemlineata*
LDEC006090	*setd*	SETD	*Leptinotarsa decemlineata*
LDEC003561	*Smyd3*	SMYD	*Leptinotarsa decemlineata*
LDEC011103	*Smyd4-1*	SMYD	*Leptinotarsa decemlineata*
LDEC001055	*Smyd4-3*	SMYD	*Leptinotarsa decemlineata*
LDEC003781	*Smyd4-3*	SMYD	*Leptinotarsa decemlineata*
LDEC004316	*Smyd4-3*	SMYD	*Leptinotarsa decemlineata*
LDEC011170	*Smyd4-3*	SMYD	*Leptinotarsa decemlineata*
LDEC015452	*Smyd4-3*	SMYD	*Leptinotarsa decemlineata*
LDEC018848	*Smyd4-4*	SMYD	*Leptinotarsa decemlineata*
LDEC018236	*Smyd4-4*	SMYD	*Leptinotarsa decemlineata*
LDEC014032	*Smyd5*	SMYD	*Leptinotarsa decemlineata*
LDEC023571	*SmydA-1*	SmydA	*Leptinotarsa decemlineata*
LDEC004931	*SmydA-3*	SmydA	*Leptinotarsa decemlineata*
LDEC015232	*SmydA-4*	SmydA	*Leptinotarsa decemlineata*
LDEC009677	*SmydA-5*	SmydA	*Leptinotarsa decemlineata*
LDEC012304	*SmydA-6*	SmydA	*Leptinotarsa decemlineata*
LDEC001146	*SmydA-8*	SmydA	*Leptinotarsa decemlineata*
LDEC013064	*Su(var)3-9*	Suv	*Leptinotarsa decemlineata*

LDEC007312	*suv*	Suv	*Leptinotarsa decemlineata*
LDEC013318	*trr*	Trx	*Leptinotarsa decemlineata*
LDEC011089	*trx*	Trx	*Leptinotarsa decemlineata*
lac992.17	*ash1*	Ash	*Lerema accius*
lac899.18	*Blimp-1*	PRDM	*Lerema accius*
lac346.12	*Blimp-1*	PRDM	*Lerema accius*
lac54.25	*egg*	Suv	*Lerema accius*
lac157.11	*Ez*	Ez	*Lerema accius*
lac615.13	*G9a*	Suv	*Lerema accius*
lac161926.6	*Hmt4-20*	-	*Lerema accius*
lac7082.4	*Mes-4*	Ash	*Lerema accius*
lac1066.30	*Set1*	Trx	*Lerema accius*
lac393.2	*Set2*	Ash	*Lerema accius*
lac677.30	*Set8*	-	*Lerema accius*
lac251.21	*setd*	SETD	*Lerema accius*
lac203.8	*Smyd3*	SMYD	*Lerema accius*
lac2058.2	*Smyd4-3*	SMYD	*Lerema accius*
lac160671.79	*Smyd4-4*	SMYD	*Lerema accius*
lac930.2	*Smyd5*	SMYD	*Lerema accius*
lac2099.9	*SmydA-1*	SmydA	*Lerema accius*
lac380.5	*SmydA-2*	SmydA	*Lerema accius*
lac2099.8	*SmydA-4*	SmydA	*Lerema accius*
lac380.4	*SmydA-5*	SmydA	*Lerema accius*
lac380.31	*SmydA-6*	SmydA	*Lerema accius*
lac289.26	*SmydA-8*	SmydA	*Lerema accius*
lac289.25	*SmydA-8*	SmydA	*Lerema accius*
lac751.34	*Su(var)3-9*	Suv	*Lerema accius*
lac90767.1	*trr*	Trx	*Lerema accius*
lac34.38	*trx*	Trx	*Lerema accius*
lac643.37	*upSET(MLL5)*	-	*Lerema accius*
LLUN005950	*egg*	Suv	*Limnephilus lunatus*
LLUN005250	*Ez*	Ez	*Limnephilus lunatus*
LLUN012587	*G9a*	Suv	*Limnephilus lunatus*
LLUN003118	*Hmt4-20*	-	*Limnephilus lunatus*
LLUN009442	*Set1*	Trx	*Limnephilus lunatus*
LLUN006029	*Set8*	-	*Limnephilus lunatus*
LLUN012732	*setd*	SETD	*Limnephilus lunatus*
LLUN011247	*Smyd4-2*	SMYD	*Limnephilus lunatus*
LLUN006391	*Smyd4-4*	SMYD	*Limnephilus lunatus*
LLUN002435	*Smyd5*	SMYD	*Limnephilus lunatus*
LLUN007945	*SmydA-2*	SmydA	*Limnephilus lunatus*
LLUN003965	*SmydA-4*	SmydA	*Limnephilus lunatus*
LLUN002737	*SmydA-4*	SmydA	*Limnephilus lunatus*
LLUN012350	*Su(var)3-9*	Suv	*Limnephilus lunatus*

LLUN008584	*trr*	Trx	*Limnephilus lunatus*
LLUN012029	*upSET(MLL5)*	-	*Limnephilus lunatus*
XP_013774094.1	-	-	*Limulus polyphemus*
XP_013779563.1	*ash1*	Ash	*Limulus polyphemus*
XP_013780438.1	*Blimp-1*	PRDM	*Limulus polyphemus*
XP_013782728.1	*Blimp-1*	PRDM	*Limulus polyphemus*
XP_013791986.1	*Blimp-1*	PRDM	*Limulus polyphemus*
XP_013794286.1	*Blimp-1*	PRDM	*Limulus polyphemus*
XP_013774016.1	*egg*	Suv	*Limulus polyphemus*
XP_013777692.1	*Ez*	Ez	*Limulus polyphemus*
XP_013777216.1	*Ez*	Ez	*Limulus polyphemus*
XP_013793296.1	*G9a*	Suv	*Limulus polyphemus*
XP_013792649.1	*G9a*	Suv	*Limulus polyphemus*
XP_013780359.1	*Hmt4-20*	-	*Limulus polyphemus*
XP_013777413.1	*Hmt4-20*	-	*Limulus polyphemus*
XP_013784275.1	*Mes-4*	Ash	*Limulus polyphemus*
XP_013775697.1	*Mes-4*	Ash	*Limulus polyphemus*
XP_013780692.1	*Set1*	Trx	*Limulus polyphemus*
XP_013773324.1	*Set2*	Ash	*Limulus polyphemus*
XP_013788393.1	*Set8*	-	*Limulus polyphemus*
XP_013782031.1	*setd*	SETD	*Limulus polyphemus*
XP_013777393.1	*Smyd4-3*	SMYD	*Limulus polyphemus*
XP_013788365.1	*Smyd5*	SMYD	*Limulus polyphemus*
XP_013772199.1	*Su(var)3-9*	Suv	*Limulus polyphemus*
XP_013782805.1	*suv*	Suv	*Limulus polyphemus*
XP_013784687.1	*trr*	Trx	*Limulus polyphemus*
XP_013779655.1	*trr*	Trx	*Limulus polyphemus*
XP_013772143.1	*trx*	Trx	*Limulus polyphemus*
XP_013792816.1	*upSET(MLL5)*	-	*Limulus polyphemus*
XP_013791181.1	*upSET(MLL5)*	-	*Limulus polyphemus*
LH22354	-	-	*Linepithema humile*
LH15972	*ash1*	Ash	*Linepithema humile*
LH13683	*Blimp-1*	PRDM	*Linepithema humile*
LH15981	*egg*	Suv	*Linepithema humile*
LH16500	*Ez*	Ez	*Linepithema humile*
LH21466	*G9a*	Suv	*Linepithema humile*
LH18409	*Hmt4-20*	-	*Linepithema humile*
LH12300	*Mes-4*	Ash	*Linepithema humile*
LH17913	*Set1*	Trx	*Linepithema humile*
LH24846	*Set2*	Ash	*Linepithema humile*
LH18657	*Set8*	-	*Linepithema humile*
LH19541	*setd*	SETD	*Linepithema humile*
LH19539	*setd*	SETD	*Linepithema humile*
LH15882	*Smyd4-2*	SMYD	*Linepithema humile*

LH18872	*Smyd4-3*	SMYD	*Linepithema humile*
LH16275	*Smyd4-3*	SMYD	*Linepithema humile*
LH24578	*Smyd4-3*	SMYD	*Linepithema humile*
LH24608	*Smyd4-3*	SMYD	*Linepithema humile*
LH15269	*Smyd4-4*	SMYD	*Linepithema humile*
LH12468	*Smyd5*	SMYD	*Linepithema humile*
LH25024	*SmydA-3*	SmydA	*Linepithema humile*
LH25025	*SmydA-4*	SmydA	*Linepithema humile*
LH16369	*SmydA-5*	SmydA	*Linepithema humile*
LH21230	*SmydA-8*	SmydA	*Linepithema humile*
LH17627	*Su(var)3-9*	Suv	*Linepithema humile*
LH14306	*suv*	Suv	*Linepithema humile*
LH18978	*trr*	Trx	*Linepithema humile*
LH13352	*trx*	Trx	*Linepithema humile*
LH15211	*upSET(MLL5)*	-	*Linepithema humile*
LOCMI00787	-	-	*Locusta miratoria*
LOCMI09053	*ash1*	Ash	*Locusta miratoria*
LOCMI03971	*Blimp-1*	PRDM	*Locusta miratoria*
LOCMI07997	*egg*	Suv	*Locusta miratoria*
LOCMI08045	*Ez*	Ez	*Locusta miratoria*
LOCMI03117	*G9a*	Suv	*Locusta miratoria*
LOCMI10213	*Hmt4-20*	-	*Locusta miratoria*
locmi_mes_4	*Mes-4*	Ash	*Locusta miratoria*
LOCMI05868	*Set1*	Trx	*Locusta miratoria*
LOCMI16575	*Set2*	Ash	*Locusta miratoria*
LOCMI08077	*Set8*	-	*Locusta miratoria*
LOCMI03802	*setd*	SETD	*Locusta miratoria*
LOCMI09401	*Smyd3*	SMYD	*Locusta miratoria*
LOCMI09977	*Smyd4-1*	SMYD	*Locusta miratoria*
LOCMI09668	*Smyd4-3*	SMYD	*Locusta miratoria*
LOCMI08571	*Smyd4-3*	SMYD	*Locusta miratoria*
LOCMI03856	*Smyd4-4*	SMYD	*Locusta miratoria*
LOCMI01890	*Smyd5*	SMYD	*Locusta miratoria*
LOCMI00956	*SmydA-1*	SmydA	*Locusta miratoria*
locmi_smydA_2	*SmydA-2*	SmydA	*Locusta miratoria*
LOCMI13222	*SmydA-4*	SmydA	*Locusta miratoria*
LOCMI04115	*SmydA-5*	SmydA	*Locusta miratoria*
LOCMI02722	*SmydA-6*	SmydA	*Locusta miratoria*
LOCMI13608	*SmydA-8*	SmydA	*Locusta miratoria*
locmi_suv	*Su(var)3-9*	Suv	*Locusta miratoria*
LOCMI02447	*suv*	Suv	*Locusta miratoria*
LOCMI06593	*trr*	Trx	*Locusta miratoria*
LOCMI08240	*trx*	Trx	*Locusta miratoria*
LOCMI04755	*upSET(MLL5)*	-	*Locusta miratoria*

LREC013115	-	-	*Loxosceles reclusa*
LREC012179	*Blimp-1*	PRDM	*Loxosceles reclusa*
LREC005103	*Mes-4*	Ash	*Loxosceles reclusa*
LREC018244	*Mes-4*	Ash	*Loxosceles reclusa*
LREC012138	*Set2*	Ash	*Loxosceles reclusa*
LREC002897	*Set2*	Ash	*Loxosceles reclusa*
LREC020476	*Set8*	-	*Loxosceles reclusa*
LREC020590	*Su(var)3-9*	Suv	*Loxosceles reclusa*
LREC001799	*trx*	Trx	*Loxosceles reclusa*
LCUP000283	-	-	*Lucilia cuprina*
LCUP018392	*ash1*	Ash	*Lucilia cuprina*
LCUP008191	*Blimp-1*	PRDM	*Lucilia cuprina*
LCUP015817	*egg*	Suv	*Lucilia cuprina*
LCUP005952	*Ez*	Ez	*Lucilia cuprina*
LCUP011706	*G9a*	Suv	*Lucilia cuprina*
LCUP009707	*Hmt4-20*	-	*Lucilia cuprina*
LCUP000697	*Mes-4*	Ash	*Lucilia cuprina*
LCUP011687	*Set1*	Trx	*Lucilia cuprina*
LCUP013762	*Set2*	Ash	*Lucilia cuprina*
LCUP000915	*Set8*	-	*Lucilia cuprina*
LCUP004880	*setd*	SETD	*Lucilia cuprina*
LCUP001038	*Smyd4-1*	SMYD	*Lucilia cuprina*
LCUP007217	*Smyd4-2*	SMYD	*Lucilia cuprina*
LCUP012337	*Smyd4-3*	SMYD	*Lucilia cuprina*
LCUP010325	*Smyd4-4*	SMYD	*Lucilia cuprina*
LCUP002371	*Smyd5*	SMYD	*Lucilia cuprina*
LCUP008487	*SmydA-1*	SmydA	*Lucilia cuprina*
LCUP001566	*SmydA-2*	SmydA	*Lucilia cuprina*
LCUP002115	*SmydA-3*	SmydA	*Lucilia cuprina*
LCUP004708	*SmydA-4*	SmydA	*Lucilia cuprina*
LCUP007602	*SmydA-5*	SmydA	*Lucilia cuprina*
LCUP002665	*SmydA-8*	SmydA	*Lucilia cuprina*
LCUP002666	*SmydA-8*	SmydA	*Lucilia cuprina*
LCUP002667	*SmydA-9*	SmydA	*Lucilia cuprina*
LCUP000300	*suv*	Suv	*Lucilia cuprina*
LCUP001960	*trr*	Trx	*Lucilia cuprina*
LCUP010760	*trx*	Trx	*Lucilia cuprina*
LCUP018340	*upSET(MLL5)*	-	*Lucilia cuprina*
LLOJ001507	-	-	*Lutzomyia longipalpis*
LLOJ006340	-	-	*Lutzomyia longipalpis*
LLOJ009331	-	-	*Lutzomyia longipalpis*
LLOJ004268	*Blimp-1*	PRDM	*Lutzomyia longipalpis*
LLOJ009396	*egg*	Suv	*Lutzomyia longipalpis*
LLOJ006291	*Ez*	Ez	*Lutzomyia longipalpis*

LLOJ002505	*G9a*	Suv	*Lutzomyia longipalpis*
LLOJ001900	*Hmt4-20*	-	*Lutzomyia longipalpis*
LLOJ007114	*Mes-4*	Ash	*Lutzomyia longipalpis*
LLOJ007115	*Mes-4*	Ash	*Lutzomyia longipalpis*
LLOJ004596	*Set2*	Ash	*Lutzomyia longipalpis*
LLOJ002416	*setd*	SETD	*Lutzomyia longipalpis*
LLOJ007749	*Smyd4-3*	SMYD	*Lutzomyia longipalpis*
LLOJ005009	*Smyd4-4*	SMYD	*Lutzomyia longipalpis*
LLOJ005920	*Smyd4-4*	SMYD	*Lutzomyia longipalpis*
LLOJ005921	*Smyd4-4*	SMYD	*Lutzomyia longipalpis*
LLOJ009604	*Smyd4-4*	SMYD	*Lutzomyia longipalpis*
LLOJ004165	*Smyd5*	SMYD	*Lutzomyia longipalpis*
LLOJ003243	*SmydA-1*	SmydA	*Lutzomyia longipalpis*
LLOJ002820	*SmydA-2*	SmydA	*Lutzomyia longipalpis*
LLOJ008798	*SmydA-3*	SmydA	*Lutzomyia longipalpis*
LLOJ000942	*SmydA-4*	SmydA	*Lutzomyia longipalpis*
LLOJ007771	*SmydA-8*	SmydA	*Lutzomyia longipalpis*
LLOJ000788	*Su(var)3-9*	Suv	*Lutzomyia longipalpis*
LLOJ000371	*trr*	Trx	*Lutzomyia longipalpis*
LLOJ006848	*trx*	Trx	*Lutzomyia longipalpis*
LLOJ007512	*upSET(MLL5)*	-	*Lutzomyia longipalpis*
Msex2.11575-RA	*ash1*	Ash	*Manduca sexta*
Msex2.11575-RB	*ash1*	Ash	*Manduca sexta*
Msex2.11575-RC	*ash1*	Ash	*Manduca sexta*
Msex2.11575-RD	*ash1*	Ash	*Manduca sexta*
Msex2.11575-RE	*ash1*	Ash	*Manduca sexta*
Msex2.06346-RA	*Blimp-1*	PRDM	*Manduca sexta*
Msex2.06346-RB	*Blimp-1*	PRDM	*Manduca sexta*
Msex2.06346-RC	*Blimp-1*	PRDM	*Manduca sexta*
Msex2.06346-RD	*Blimp-1*	PRDM	*Manduca sexta*
Msex2.06346-RE	*Blimp-1*	PRDM	*Manduca sexta*
Msex2.06346-RF	*Blimp-1*	PRDM	*Manduca sexta*
Msex2.10962-RA	*Blimp-1*	PRDM	*Manduca sexta*
Msex2.10437-RA	*egg*	Suv	*Manduca sexta*
Msex2.12341-RA	*Ez*	Ez	*Manduca sexta*
Msex2.12341-RB	*Ez*	Ez	*Manduca sexta*
Msex2.10252-RC	*G9a*	Suv	*Manduca sexta*
Msex2.10252-RA	*G9a*	Suv	*Manduca sexta*
Msex2.10252-RB	*G9a*	Suv	*Manduca sexta*
Msex2.06794-RA	*Hmt4-20*	-	*Manduca sexta*
Msex2.06794-RB	*Hmt4-20*	-	*Manduca sexta*
Msex2.03620-RA	*Set1*	Trx	*Manduca sexta*
Msex2.03620-RB	*Set1*	Trx	*Manduca sexta*
Msex2.05433-RA	*Set2*	Ash	*Manduca sexta*

Msex2.05433-RB	*Set2*	Ash	*Manduca sexta*
Msex2.03628-RA	*Set8*	-	*Manduca sexta*
Msex2.05202-RA	*setd*	SETD	*Manduca sexta*
Msex2.11948-RA	*setd*	SETD	*Manduca sexta*
Msex2.14358-RA	*Smyd4-3*	SMYD	*Manduca sexta*
Msex2.03698-RA	*Smyd4-3*	SMYD	*Manduca sexta*
Msex2.07730-RA	*Smyd4-4*	SMYD	*Manduca sexta*
Msex2.03357-RA	*Smyd4-4*	SMYD	*Manduca sexta*
Msex2.03357-RB	*Smyd4-4*	SMYD	*Manduca sexta*
Msex2.07382-RA	*Smyd5*	SMYD	*Manduca sexta*
Msex2.03393-RA	*SmydA-1*	SmydA	*Manduca sexta*
Msex2.06019-RB	*SmydA-2*	SmydA	*Manduca sexta*
Msex2.06019-RA	*SmydA-2*	SmydA	*Manduca sexta*
Msex2.06043-RA	*SmydA-3*	SmydA	*Manduca sexta*
Msex2.03394-RC	*SmydA-4*	SmydA	*Manduca sexta*
Msex2.03394-RA	*SmydA-4*	SmydA	*Manduca sexta*
Msex2.03394-RB	*SmydA-4*	SmydA	*Manduca sexta*
Msex2.03394-RD	*SmydA-4*	SmydA	*Manduca sexta*
Msex2.05529-RB	*SmydA-5*	SmydA	*Manduca sexta*
Msex2.05529-RA	*SmydA-5*	SmydA	*Manduca sexta*
Msex2.06047-RA	*SmydA-6*	SmydA	*Manduca sexta*
Msex2.09238-RA	*SmydA-8*	SmydA	*Manduca sexta*
Msex2.09239-RA	*SmydA-8*	SmydA	*Manduca sexta*
Msex2.07042-RA	*suv*	Suv	*Manduca sexta*
Msex2.08650-RA	*trr*	Trx	*Manduca sexta*
Msex2.08650-RB	*trr*	Trx	*Manduca sexta*
Msex2.08650-RC	*trr*	Trx	*Manduca sexta*
Msex2.11099-RA	*upSET(MLL5)*	-	*Manduca sexta*
Msex2.11099-RB	*upSET(MLL5)*	-	*Manduca sexta*
Msex2.11099-RC	*upSET(MLL5)*	-	*Manduca sexta*
Msex2.11099-RD	*upSET(MLL5)*	-	*Manduca sexta*
Msex2.11099-RE	*upSET(MLL5)*	-	*Manduca sexta*
Msex2.11099-RF	*upSET(MLL5)*	-	*Manduca sexta*
Msex2.11099-RG	*upSET(MLL5)*	-	*Manduca sexta*
Msex2.11099-RH	*upSET(MLL5)*	-	*Manduca sexta*
Msex2.11099-RI	*upSET(MLL5)*	-	*Manduca sexta*
Msex2.11099-RJ	*upSET(MLL5)*	-	*Manduca sexta*
Msex2.11099-RK	*upSET(MLL5)*	-	*Manduca sexta*
Msex2.11099-RL	*upSET(MLL5)*	-	*Manduca sexta*
Msex2.11099-RM	*upSET(MLL5)*	-	*Manduca sexta*
MDES004780	-	-	*Mayetiola destructor*
MDES015892	-	-	*Mayetiola destructor*
MDES017515	-	-	*Mayetiola destructor*
MDES017724	-	-	*Mayetiola destructor*

MDES018075	-	-	*Mayetiola destructor*
MDES018152	-	-	*Mayetiola destructor*
MDES018517	-	-	*Mayetiola destructor*
MDES018884	-	-	*Mayetiola destructor*
MDES019051	-	-	*Mayetiola destructor*
MDES019196	-	-	*Mayetiola destructor*
MDES019384	-	-	*Mayetiola destructor*
MDES019442	-	-	*Mayetiola destructor*
MDES019564	-	-	*Mayetiola destructor*
MDES019629	-	-	*Mayetiola destructor*
MDES019647	-	-	*Mayetiola destructor*
MDES020064	-	-	*Mayetiola destructor*
MDES005927	-	-	*Mayetiola destructor*
MDES009322	*ash1*	Ash	*Mayetiola destructor*
MDES013293	*ash1*	Ash	*Mayetiola destructor*
MDES015171	*ash1*	Ash	*Mayetiola destructor*
MDES019720	*ash1*	Ash	*Mayetiola destructor*
MDES000762	*Blimp-1*	PRDM	*Mayetiola destructor*
MDES004660	*egg*	Suv	*Mayetiola destructor*
MDES010541	*egg*	Suv	*Mayetiola destructor*
MDES013164	*egg*	Suv	*Mayetiola destructor*
MDES010774	*Ez*	Ez	*Mayetiola destructor*
MDES012126	*G9a*	Suv	*Mayetiola destructor*
MDES003560	*Hmt4-20*	-	*Mayetiola destructor*
MDES006259	*Mes-4*	Ash	*Mayetiola destructor*
MDES009331	*Mes-4*	Ash	*Mayetiola destructor*
MDES019977	*Mes-4*	Ash	*Mayetiola destructor*
MDES003857	*Set1*	Trx	*Mayetiola destructor*
MDES005832	*Set2*	Ash	*Mayetiola destructor*
MDES015000	*Set2*	Ash	*Mayetiola destructor*
MDES008970	*Set8*	-	*Mayetiola destructor*
MDES008973	*Set8*	-	*Mayetiola destructor*
MDES012871	*Smyd4-1*	SMYD	*Mayetiola destructor*
MDES012900	*Smyd4-2*	SMYD	*Mayetiola destructor*
MDES010788	*Smyd4-4*	SMYD	*Mayetiola destructor*
MDES009713	*Smyd4-4*	SMYD	*Mayetiola destructor*
MDES010008	*Smyd4-4*	SMYD	*Mayetiola destructor*
MDES017360	*Smyd4-4*	SMYD	*Mayetiola destructor*
MDES017361	*Smyd4-4*	SMYD	*Mayetiola destructor*
MDES017362	*Smyd4-4*	SMYD	*Mayetiola destructor*
MDES017432	*Smyd4-4*	SMYD	*Mayetiola destructor*
MDES017433	*Smyd4-4*	SMYD	*Mayetiola destructor*
MDES017455	*Smyd4-4*	SMYD	*Mayetiola destructor*
MDES017824	*Smyd4-4*	SMYD	*Mayetiola destructor*

MDES019529	*Smyd5*	SMYD	*Mayetiola destructor*
MDES013492	*SmydA-1*	SmydA	*Mayetiola destructor*
MDES009703	*SmydA-2*	SmydA	*Mayetiola destructor*
MDES011038	*SmydA-3*	SmydA	*Mayetiola destructor*
MDES010088	*SmydA-5*	SmydA	*Mayetiola destructor*
MDES002338	*Su(var)3-9*	Suv	*Mayetiola destructor*
MDES002499	*suv*	Suv	*Mayetiola destructor*
MDES004136	*trr*	Trx	*Mayetiola destructor*
MDES018256	*trr*	Trx	*Mayetiola destructor*
MDES005703	*trx*	Trx	*Mayetiola destructor*
MDES011632	*upSET(MLL5)*	-	*Mayetiola destructor*
Mrot04594	*ash1*	Ash	*Megachile rotundata*
Mrot00136	*Blimp-1*	PRDM	*Megachile rotundata*
Mrot01289	*egg*	Suv	*Megachile rotundata*
Mrot10765	*Ez*	Ez	*Megachile rotundata*
Mrot00982	*G9a*	Suv	*Megachile rotundata*
Mrot02943	*Hmt4-20*	-	*Megachile rotundata*
Mrot10786	*Mes-4*	Ash	*Megachile rotundata*
Mrot01457	*Set1*	Trx	*Megachile rotundata*
Mrot08140	*Set2*	Ash	*Megachile rotundata*
Mrot05350	*Set8*	-	*Megachile rotundata*
Mrot10030	*setd*	SETD	*Megachile rotundata*
Mrot10032	*setd*	SETD	*Megachile rotundata*
Mrot01630	*Smyd4-2*	SMYD	*Megachile rotundata*
Mrot11105	*Smyd4-2*	SMYD	*Megachile rotundata*
Mrot03129	*Smyd4-3*	SMYD	*Megachile rotundata*
Mrot01502	*Smyd4-3*	SMYD	*Megachile rotundata*
Mrot04731	*Smyd4-4*	SMYD	*Megachile rotundata*
Mrot11920	*Smyd4-4*	SMYD	*Megachile rotundata*
Mrot08726	*Smyd5*	SMYD	*Megachile rotundata*
Mrot07238	*SmydA-1*	SmydA	*Megachile rotundata*
Mrot12858	*SmydA-3*	SmydA	*Megachile rotundata*
Mrot12857	*SmydA-4*	SmydA	*Megachile rotundata*
Mrot04012	*SmydA-5*	SmydA	*Megachile rotundata*
Mrot02009	*SmydA-8*	SmydA	*Megachile rotundata*
Mrot02583	*suv*	Suv	*Megachile rotundata*
Mrot12156	*trr*	Trx	*Megachile rotundata*
Mrot09154	*trx*	Trx	*Megachile rotundata*
Mrot02560	*upSET(MLL5)*	-	*Megachile rotundata*
MESCA001248	*ash1*	Ash	*Megaselia scalaris*
MESCA007133	*Blimp-1*	PRDM	*Megaselia scalaris*
MESCA001701	*Hmt4-20*	-	*Megaselia scalaris*
MESCA006230	*Mes-4*	Ash	*Megaselia scalaris*
MESCA006340	*Mes-4*	Ash	*Megaselia scalaris*

MESCA010216	*Set1*	Trx	*Megaselia scalaris*
MESCA005153	*Smyd4-3*	SMYD	*Megaselia scalaris*
MESCA007382	*Smyd4-4*	SMYD	*Megaselia scalaris*
MESCA003377	*SmydA-3*	SmydA	*Megaselia scalaris*
MESCA004004	*SmydA-6*	SmydA	*Megaselia scalaris*
MESCA001047	*SmydA-8*	SmydA	*Megaselia scalaris*
MESCA006589	*SmydA-9*	SmydA	*Megaselia scalaris*
MESCA004542	*Su(var)3-9*	Suv	*Megaselia scalaris*
MESCA005035	*suv*	Suv	*Megaselia scalaris*
Mqua05919	-	-	*Melipona quadrifasciata*
Mqua07221	*ash1*	Ash	*Melipona quadrifasciata*
Mqua05825	*Blimp-1*	PRDM	*Melipona quadrifasciata*
Mqua00462	*egg*	Suv	*Melipona quadrifasciata*
Mqua13259	*Ez*	Ez	*Melipona quadrifasciata*
Mqua10048	*G9a*	Suv	*Melipona quadrifasciata*
Mqua05000	*Hmt4-20*	-	*Melipona quadrifasciata*
Mqua13274	*Mes-4*	Ash	*Melipona quadrifasciata*
Mqua05445	*Set1*	Trx	*Melipona quadrifasciata*
Mqua08311	*Set2*	Ash	*Melipona quadrifasciata*
Mqua06865	*Set8*	-	*Melipona quadrifasciata*
Mqua05331	*setd*	SETD	*Melipona quadrifasciata*
Mqua05329	*setd*	SETD	*Melipona quadrifasciata*
Mqua10376	*Smyd4-1*	SMYD	*Melipona quadrifasciata*
Mqua09547	*Smyd4-2*	SMYD	*Melipona quadrifasciata*
Mqua13705	*Smyd4-2*	SMYD	*Melipona quadrifasciata*
Mqua06430	*Smyd4-3*	SMYD	*Melipona quadrifasciata*
Mqua02865	*Smyd4-4*	SMYD	*Melipona quadrifasciata*
Mqua07821	*Smyd4-4*	SMYD	*Melipona quadrifasciata*
Mqua11608	*Smyd5*	SMYD	*Melipona quadrifasciata*
Mqua01976	*SmydA-3*	SmydA	*Melipona quadrifasciata*
Mqua01975	*SmydA-4*	SmydA	*Melipona quadrifasciata*
Mqua10068	*SmydA-5*	SmydA	*Melipona quadrifasciata*
Mqua01943	*SmydA-8*	SmydA	*Melipona quadrifasciata*
Mqua12688	*Su(var)3-9*	Suv	*Melipona quadrifasciata*
Mqua10266	*suv*	Suv	*Melipona quadrifasciata*
Mqua14950	*trr*	Trx	*Melipona quadrifasciata*
Mqua15195	*trx*	Trx	*Melipona quadrifasciata*
Mqua06846	*upSET(MLL5)*	-	*Melipona quadrifasciata*
MCINX005952-PA	*ash1*	Ash	*Melitaea cinxia*
MCINX009345-PA	*Blimp-1*	PRDM	*Melitaea cinxia*
MCINX010108-PA	*Hmt4-20*	-	*Melitaea cinxia*
MCINX004631-PA	*Mes-4*	Ash	*Melitaea cinxia*
MCINX015030-PA	*Set2*	Ash	*Melitaea cinxia*
MCINX011554-PA	*Set8*	-	*Melitaea cinxia*

MCINX010627-PA	*setd*	SETD	*Melitaea cinxia*
MCINX003745-PA	*Smyd3*	SMYD	*Melitaea cinxia*
MCINX008955-PA	*Smyd4-2*	SMYD	*Melitaea cinxia*
MCINX016501-PA	*Smyd4-3*	SMYD	*Melitaea cinxia*
MCINX000039-PA	*Smyd4-4*	SMYD	*Melitaea cinxia*
MCINX006664-PA	*Smyd5*	SMYD	*Melitaea cinxia*
MCINX002625-PA	*SmydA-1*	SmydA	*Melitaea cinxia*
MCINX012410-PA	*SmydA-2*	SmydA	*Melitaea cinxia*
MCINX003723-PA	*SmydA-3*	SmydA	*Melitaea cinxia*
MCINX012412-PA	*SmydA-5*	SmydA	*Melitaea cinxia*
MCINX004394-PA	*SmydA-8*	SmydA	*Melitaea cinxia*
MCINX012060-PA	*SmydA-8*	SmydA	*Melitaea cinxia*
MCINX002150-PA	*Su(var)3-9*	Suv	*Melitaea cinxia*
XP_003744440.1	-	-	*Metaseiulus occidentalis*
XP_003738192.1	*Blimp-1*	PRDM	*Metaseiulus occidentalis*
XP_003747828.1	*egg*	Suv	*Metaseiulus occidentalis*
XP_003748456.1	*Ez*	Ez	*Metaseiulus occidentalis*
XP_003743205.1	*G9a*	Suv	*Metaseiulus occidentalis*
XP_003745640.1	*Hmt4-20*	-	*Metaseiulus occidentalis*
XP_003743600.1	*Mes-4*	Ash	*Metaseiulus occidentalis*
XP_003737286.1	*Set1*	Trx	*Metaseiulus occidentalis*
XP_003740543.1	*Set8*	-	*Metaseiulus occidentalis*
XP_003745694.1	*setd*	SETD	*Metaseiulus occidentalis*
XP_003742261.1	*Smyd4-3*	SMYD	*Metaseiulus occidentalis*
XP_003748305.1	*Su(var)3-9*	Suv	*Metaseiulus occidentalis*
XP_003747129.1	*Su(var)3-9*	Suv	*Metaseiulus occidentalis*
XP_003747204.1	*Su(var)3-9*	Suv	*Metaseiulus occidentalis*
XP_003740087.1	*trx*	Trx	*Metaseiulus occidentalis*
XP_003741200.1	*trx*	Trx	*Metaseiulus occidentalis*
XP_008551336.2	-	-	*Microplitis demolitor*
XP_008543689.1	*ash1*	Ash	*Microplitis demolitor*
XP_008543891.1	*Blimp-1*	PRDM	*Microplitis demolitor*
XP_008556889.1	*egg*	Suv	*Microplitis demolitor*
XP_008556241.1	*Ez*	Ez	*Microplitis demolitor*
XP_008556240.1	*Ez*	Ez	*Microplitis demolitor*
XP_008550724.1	*G9a*	Suv	*Microplitis demolitor*
XP_008550676.2	*Hmt4-20*	-	*Microplitis demolitor*
XP_008558386.1	*Mes-4*	Ash	*Microplitis demolitor*
XP_014297501.1	*Set1*	Trx	*Microplitis demolitor*
XP_008547281.1	*Set2*	Ash	*Microplitis demolitor*
XP_008557189.1	*Set8*	-	*Microplitis demolitor*
XP_008554714.2	*setd*	SETD	*Microplitis demolitor*
XP_014300365.1	*Smyd4-1*	SMYD	*Microplitis demolitor*
XP_008547557.1	*Smyd4-2*	SMYD	*Microplitis demolitor*

XP_008556773.1	*Smyd4-2*	SMYD	*Microplitis demolitor*
XP_008560866.2	*Smyd4-2*	SMYD	*Microplitis demolitor*
XP_014299276.1	*Smyd4-2*	SMYD	*Microplitis demolitor*
XP_008547074.1	*Smyd4-3*	SMYD	*Microplitis demolitor*
XP_008544157.1	*Smyd4-3*	SMYD	*Microplitis demolitor*
XP_008544405.1	*Smyd4-3*	SMYD	*Microplitis demolitor*
XP_008551110.1	*Smyd4-3*	SMYD	*Microplitis demolitor*
XP_008551286.1	*Smyd4-3*	SMYD	*Microplitis demolitor*
XP_008552149.1	*Smyd4-3*	SMYD	*Microplitis demolitor*
XP_008552152.1	*Smyd4-3*	SMYD	*Microplitis demolitor*
XP_008556789.1	*Smyd4-3*	SMYD	*Microplitis demolitor*
XP_008560103.1	*Smyd4-4*	SMYD	*Microplitis demolitor*
XP_008556774.1	*Smyd4-4*	SMYD	*Microplitis demolitor*
XP_008558969.1	*Smyd4-4*	SMYD	*Microplitis demolitor*
XP_014299567.1	*Smyd4-4*	SMYD	*Microplitis demolitor*
XP_008546011.1	*Smyd5*	SMYD	*Microplitis demolitor*
XP_008559572.1	*SmydA-1*	SmydA	*Microplitis demolitor*
XP_008547232.1	*SmydA-3*	SmydA	*Microplitis demolitor*
XP_008547423.1	*SmydA-4*	SmydA	*Microplitis demolitor*
XP_008552514.1	*SmydA-5*	SmydA	*Microplitis demolitor*
XP_014297848.1	*Su(var)3-9*	Suv	*Microplitis demolitor*
XP_008558116.1	*suv*	Suv	*Microplitis demolitor*
XP_014296916.1	*trr*	Trx	*Microplitis demolitor*
XP_014295406.1	*trx*	Trx	*Microplitis demolitor*
XP_014300525.1	*upSET(MLL5)*	-	*Microplitis demolitor*
XP_012522270.1	-	-	*Monomorium pharaonis*
XP_012539904.1	*Blimp-1*	PRDM	*Monomorium pharaonis*
XP_012533484.1	*Ez*	Ez	*Monomorium pharaonis*
XP_012531062.1	*G9a*	Suv	*Monomorium pharaonis*
XP_012525264.1	*Hmt4-20*	-	*Monomorium pharaonis*
XP_012537168.1	*Mes-4*	Ash	*Monomorium pharaonis*
XP_012542824.1	*Set1*	Trx	*Monomorium pharaonis*
XP_012537071.1	*Set2*	Ash	*Monomorium pharaonis*
XP_012525334.1	*setd*	SETD	*Monomorium pharaonis*
XP_012526872.1	*Smyd4-1*	SMYD	*Monomorium pharaonis*
XP_012521783.1	*Smyd4-2*	SMYD	*Monomorium pharaonis*
XP_012540827.1	*Smyd4-2*	SMYD	*Monomorium pharaonis*
XP_012543048.1	*Smyd4-3*	SMYD	*Monomorium pharaonis*
XP_012522849.1	*Smyd4-4*	SMYD	*Monomorium pharaonis*
XP_012529035.1	*Smyd5*	SMYD	*Monomorium pharaonis*
XP_012537742.1	*SmydA-3*	SmydA	*Monomorium pharaonis*
XP_012541306.1	*SmydA-5*	SmydA	*Monomorium pharaonis*
XP_012532946.1	*Su(var)3-9*	Suv	*Monomorium pharaonis*
XP_012536533.1	*suv*	Suv	*Monomorium pharaonis*

XP_012530896.1	*trx*	Trx	*Monomorium pharaonis*
XP_012541438.1	*upSET(MLL5)*	-	*Monomorium pharaonis*
MDOA006797	*ash1*	Ash	*Musca domestica*
MDOA009229	*egg*	Suv	*Musca domestica*
MDOA008795	*Ez*	Ez	*Musca domestica*
MDOA000093	*G9a*	Suv	*Musca domestica*
MDOA011066	*Hmt4-20*	-	*Musca domestica*
MDOA002588	*Mes-4*	Ash	*Musca domestica*
MDOA004753	*Set1*	Trx	*Musca domestica*
MDOA011328	*Set2*	Ash	*Musca domestica*
MDOA007714	*Set8*	-	*Musca domestica*
MDOA006911	*setd*	SETD	*Musca domestica*
MDOA000682	*Smyd4-1*	SMYD	*Musca domestica*
MDOA007016	*Smyd4-2*	SMYD	*Musca domestica*
MDOA015400	*Smyd4-3*	SMYD	*Musca domestica*
MDOA012031	*Smyd4-4*	SMYD	*Musca domestica*
MDOA014801	*Smyd5*	SMYD	*Musca domestica*
MDOA009538	*SmydA-1*	SmydA	*Musca domestica*
MDOA004951	*SmydA-2*	SmydA	*Musca domestica*
MDOA011361	*SmydA-3*	SmydA	*Musca domestica*
MDOA009762	*SmydA-4*	SmydA	*Musca domestica*
MDOA006356	*SmydA-5*	SmydA	*Musca domestica*
MDOA009169	*SmydA-6*	SmydA	*Musca domestica*
MDOA001694	*SmydA-6*	SmydA	*Musca domestica*
MDOA013751	*SmydA-6*	SmydA	*Musca domestica*
MDOA000486	*SmydA-7*	SmydA	*Musca domestica*
MDOA000184	*SmydA-8*	SmydA	*Musca domestica*
MDOA004711	*SmydA-8*	SmydA	*Musca domestica*
MDOA006415	*SmydA-8*	SmydA	*Musca domestica*
MDOA008409	*SmydA-8*	SmydA	*Musca domestica*
MDOA012054	*SmydA-8*	SmydA	*Musca domestica*
MDOA015493	*SmydA-8*	SmydA	*Musca domestica*
MDOA007214	*SmydA-9*	SmydA	*Musca domestica*
MDOA006177	*SmydA-9*	SmydA	*Musca domestica*
MDOA008584	*Su(var)3-9*	Suv	*Musca domestica*
MDOA014040	*trr*	Trx	*Musca domestica*
MDOA009761	*trx*	Trx	*Musca domestica*
MDOA013044	*upSET(MLL5)*	-	*Musca domestica*
NV11654	-	-	*Nasonia vitripennis*
NV14044	-	-	*Nasonia vitripennis*
NV14087	-	-	*Nasonia vitripennis*
NV17278	-	-	*Nasonia vitripennis*
NV17530	-	-	*Nasonia vitripennis*
NV19191	-	-	*Nasonia vitripennis*

NV22581	-	-	*Nasonia vitripennis*
NV25539	-	-	*Nasonia vitripennis*
NV25558	-	-	*Nasonia vitripennis*
NV14047	*ash1*	Ash	*Nasonia vitripennis*
NV12817	*Blimp-1*	PRDM	*Nasonia vitripennis*
NV10069	*egg*	Suv	*Nasonia vitripennis*
NV18919	*Ez*	Ez	*Nasonia vitripennis*
NV11612	*G9a*	Suv	*Nasonia vitripennis*
NV18604	*Hmt4-20*	-	*Nasonia vitripennis*
NV18533	*Hmt4-20*	-	*Nasonia vitripennis*
NV11023	*Mes-4*	Ash	*Nasonia vitripennis*
NV15594	*Set1*	Trx	*Nasonia vitripennis*
NV11717	*Set2*	Ash	*Nasonia vitripennis*
NV11709	*Set8*	-	*Nasonia vitripennis*
NV11710	*Set8*	-	*Nasonia vitripennis*
NV18081	*Set8*	-	*Nasonia vitripennis*
NV22706	*Set8*	-	*Nasonia vitripennis*
NV25438	*Set8*	-	*Nasonia vitripennis*
NV26123	*Set8*	-	*Nasonia vitripennis*
NV10907	*setd*	SETD	*Nasonia vitripennis*
NV21718	*Smyd4-2*	SMYD	*Nasonia vitripennis*
NV19127	*Smyd4-3*	SMYD	*Nasonia vitripennis*
NV10465	*Smyd4-3*	SMYD	*Nasonia vitripennis*
NV11407	*Smyd4-3*	SMYD	*Nasonia vitripennis*
NV13931	*Smyd4-3*	SMYD	*Nasonia vitripennis*
NV18759	*Smyd4-3*	SMYD	*Nasonia vitripennis*
NV22774	*Smyd4-3*	SMYD	*Nasonia vitripennis*
NV18064	*Smyd4-4*	SMYD	*Nasonia vitripennis*
NV10223	*Smyd4-4*	SMYD	*Nasonia vitripennis*
NV12278	*Smyd4-4*	SMYD	*Nasonia vitripennis*
NV13215	*Smyd4-4*	SMYD	*Nasonia vitripennis*
NV14061	*Smyd4-4*	SMYD	*Nasonia vitripennis*
NV21824	*Smyd4-4*	SMYD	*Nasonia vitripennis*
NV22744	*Smyd4-4*	SMYD	*Nasonia vitripennis*
NV23325	*Smyd4-4*	SMYD	*Nasonia vitripennis*
NV23933	*Smyd4-4*	SMYD	*Nasonia vitripennis*
NV18013	*Smyd5*	SMYD	*Nasonia vitripennis*
NV14832	*SmydA-1*	SmydA	*Nasonia vitripennis*
NV11805	*SmydA-3*	SmydA	*Nasonia vitripennis*
NV13135	*SmydA-5*	SmydA	*Nasonia vitripennis*
NV11804	*SmydA-6*	SmydA	*Nasonia vitripennis*
NV24437	*SmydA-8*	SmydA	*Nasonia vitripennis*
NV17813	*Su(var)3-9*	Suv	*Nasonia vitripennis*
NV16420	*suv*	Suv	*Nasonia vitripennis*

NV15493	*trr*	Trx	*Nasonia vitripennis*
NV16583	*upSET(MLL5)*	-	*Nasonia vitripennis*
NLU012366.1	-	-	*Nilaparvata lugens*
NLU025978.1	*Blimp-1*	PRDM	*Nilaparvata lugens*
NLU002390.1	*Blimp-1*	PRDM	*Nilaparvata lugens*
NLU012796.1	*Blimp-1*	PRDM	*Nilaparvata lugens*
NLU014118.1	*Blimp-1*	PRDM	*Nilaparvata lugens*
NLU016192.1	*Blimp-1*	PRDM	*Nilaparvata lugens*
NLU018902.1	*Blimp-1*	PRDM	*Nilaparvata lugens*
NLU016222.1	*egg*	Suv	*Nilaparvata lugens*
NLU021947.1	*egg*	Suv	*Nilaparvata lugens*
NLU024424.3	*Ez*	Ez	*Nilaparvata lugens*
NLU019099.2	*G9a*	Suv	*Nilaparvata lugens*
NLU018930.1	*G9a*	Suv	*Nilaparvata lugens*
NLU012978.1	*Hmt4-20*	-	*Nilaparvata lugens*
NLU027254.1	*Mes-4*	Ash	*Nilaparvata lugens*
NLU002794.2	*Set1*	Trx	*Nilaparvata lugens*
NLU010322.1	*Set1*	Trx	*Nilaparvata lugens*
NLU022938.1	*Set2*	Ash	*Nilaparvata lugens*
NLU016891.1	*Set8*	-	*Nilaparvata lugens*
NLU023473.1	*setd*	SETD	*Nilaparvata lugens*
NLU028562.1	*Smyd4-1*	SMYD	*Nilaparvata lugens*
NLU009524.1	*Smyd4-3*	SMYD	*Nilaparvata lugens*
NLU015176.1	*Smyd4-3*	SMYD	*Nilaparvata lugens*
NLU014703.1	*Smyd5*	SMYD	*Nilaparvata lugens*
NLU003997.1	*SmydA-2*	SmydA	*Nilaparvata lugens*
NLU013240.1	*SmydA-3*	SmydA	*Nilaparvata lugens*
NLU005116.1	*SmydA-4*	SmydA	*Nilaparvata lugens*
NLU008274.1	*SmydA-6*	SmydA	*Nilaparvata lugens*
NLU028809.1	*trr*	Trx	*Nilaparvata lugens*
NLU017111.1	*trx*	Trx	*Nilaparvata lugens*
OFAS004228	*egg*	Suv	*Oncopeltus fasciatus*
OFAS004227	*egg*	Suv	*Oncopeltus fasciatus*
OFAS002597	*Ez*	Ez	*Oncopeltus fasciatus*
OFAS007451	*G9a*	Suv	*Oncopeltus fasciatus*
OFAS005156	*Hmt4-20*	-	*Oncopeltus fasciatus*
OFAS010169	*Mes-4*	Ash	*Oncopeltus fasciatus*
OFAS013257	*Set1*	Trx	*Oncopeltus fasciatus*
OFAS017996	*Set1*	Trx	*Oncopeltus fasciatus*
OFAS003387	*Set2*	Ash	*Oncopeltus fasciatus*
OFAS000414	*Set8*	-	*Oncopeltus fasciatus*
OFAS015992	*setd*	SETD	*Oncopeltus fasciatus*
OFAS002440	*Smyd3*	SMYD	*Oncopeltus fasciatus*
OFAS001027	*Smyd4-2*	SMYD	*Oncopeltus fasciatus*

OFAS009133	*Smyd4-3*	SMYD	*Oncopeltus fasciatus*
OFAS000512	*Smyd4-3*	SMYD	*Oncopeltus fasciatus*
OFAS016549	*Smyd4-4*	SMYD	*Oncopeltus fasciatus*
OFAS013600	*Smyd5*	SMYD	*Oncopeltus fasciatus*
OFAS003209	*SmydA-3*	SmydA	*Oncopeltus fasciatus*
OFAS000133	*SmydA-5*	SmydA	*Oncopeltus fasciatus*
OFAS008714	*SmydA-8*	SmydA	*Oncopeltus fasciatus*
OFAS016410	*Su(var)3-9*	Suv	*Oncopeltus fasciatus*
OFAS015321	*suv*	Suv	*Oncopeltus fasciatus*
OFAS000131	*trr*	Trx	*Oncopeltus fasciatus*
OFAS006231	*trx*	Trx	*Oncopeltus fasciatus*
OFAS006758	*upSET(MLL5)*	-	*Oncopeltus fasciatus*
OFAS002811	*upSET(MLL5)*	-	*Oncopeltus fasciatus*
OTAU016833	*ash1*	Ash	*Onthophagus taurus*
OTAU005915	*Blimp-1*	PRDM	*Onthophagus taurus*
OTAU013038	*egg*	Suv	*Onthophagus taurus*
OTAU006929	*Ez*	Ez	*Onthophagus taurus*
OTAU016220	*G9a*	Suv	*Onthophagus taurus*
OTAU010040	*Hmt4-20*	-	*Onthophagus taurus*
OTAU009395	*Mes-4*	Ash	*Onthophagus taurus*
OTAU015711	*Set1*	Trx	*Onthophagus taurus*
OTAU003183	*Set2*	Ash	*Onthophagus taurus*
OTAU007446	*Set8*	-	*Onthophagus taurus*
OTAU011533	*setd*	SETD	*Onthophagus taurus*
OTAU007040	*Smyd4-1*	SMYD	*Onthophagus taurus*
OTAU008673	*Smyd4-2*	SMYD	*Onthophagus taurus*
OTAU001249	*Smyd4-3*	SMYD	*Onthophagus taurus*
OTAU000114	*Smyd5*	SMYD	*Onthophagus taurus*
OTAU005073	*SmydA-1*	SmydA	*Onthophagus taurus*
OTAU009777	*SmydA-2*	SmydA	*Onthophagus taurus*
OTAU010523	*SmydA-3*	SmydA	*Onthophagus taurus*
OTAU005071	*SmydA-4*	SmydA	*Onthophagus taurus*
OTAU011495	*SmydA-4*	SmydA	*Onthophagus taurus*
OTAU005068	*SmydA-5*	SmydA	*Onthophagus taurus*
OTAU002653	*SmydA-8*	SmydA	*Onthophagus taurus*
OTAU013106	*Su(var)3-9*	Suv	*Onthophagus taurus*
OTAU008228	*suv*	Suv	*Onthophagus taurus*
OTAU006134	*trr*	Trx	*Onthophagus taurus*
OTAU008921	*trx*	Trx	*Onthophagus taurus*
OTAU000670	*upSET(MLL5)*	-	*Onthophagus taurus*
ODN02559.1	-	-	*Orchesella cincta*
ODN06200.1	-	-	*Orchesella cincta*
ODM99115.1	-	-	*Orchesella cincta*
ODN00252.1	-	-	*Orchesella cincta*

ODN01047.1	-	-	*Orchesella cincta*
ODN06313.1	-	-	*Orchesella cincta*
ODM98315.1	*ash1*	Ash	*Orchesella cincta*
ODM87160.1	*ash1*	Ash	*Orchesella cincta*
ODM87234.1	*Blimp-1*	PRDM	*Orchesella cincta*
ODM97156.1	*egg*	Suv	*Orchesella cincta*
ODM86873.1	*egg*	Suv	*Orchesella cincta*
ODM88374.1	*egg*	Suv	*Orchesella cincta*
ODM89038.1	*egg*	Suv	*Orchesella cincta*
ODM90642.1	*egg*	Suv	*Orchesella cincta*
ODM91289.1	*egg*	Suv	*Orchesella cincta*
ODM97270.1	*egg*	Suv	*Orchesella cincta*
ODM99504.1	*egg*	Suv	*Orchesella cincta*
ODN05360.1	*Ez*	Ez	*Orchesella cincta*
ODM92914.1	*Ez*	Ez	*Orchesella cincta*
ODN00488.1	*Ez*	Ez	*Orchesella cincta*
ODN01184.1	*Ez*	Ez	*Orchesella cincta*
ODN02335.1	*Ez*	Ez	*Orchesella cincta*
ODM87354.1	*G9a*	Suv	*Orchesella cincta*
ODM98111.1	*G9a*	Suv	*Orchesella cincta*
ODN00570.1	*Hmt4-20*	-	*Orchesella cincta*
ODM88966.1	*Hmt4-20*	-	*Orchesella cincta*
ODN05117.1	*Hmt4-20*	-	*Orchesella cincta*
ODM92236.1	*Mes-4*	Ash	*Orchesella cincta*
ODN04926.1	*Set1*	Trx	*Orchesella cincta*
ODN01969.1	*Set1*	Trx	*Orchesella cincta*
ODM96183.1	*Set2*	Ash	*Orchesella cincta*
ODN04218.1	*Set8*	-	*Orchesella cincta*
ODM90018.1	*Set8*	-	*Orchesella cincta*
ODM94229.1	*setd*	SETD	*Orchesella cincta*
ODM99569.1	*Smyd4-2*	SMYD	*Orchesella cincta*
ODM95186.1	*Smyd4-2*	SMYD	*Orchesella cincta*
ODM97274.1	*Smyd4-2*	SMYD	*Orchesella cincta*
ODN03065.1	*Smyd4-3*	SMYD	*Orchesella cincta*
ODN03685.1	*Smyd4-3*	SMYD	*Orchesella cincta*
ODN05092.1	*Smyd4-3*	SMYD	*Orchesella cincta*
ODM91701.1	*Smyd4-4*	SMYD	*Orchesella cincta*
ODN00025.1	*Smyd5*	SMYD	*Orchesella cincta*
ODM97856.1	*SmydA-1*	SmydA	*Orchesella cincta*
ODM97009.1	*SmydA-1*	SmydA	*Orchesella cincta*
ODM96368.1	*SmydA-2*	SmydA	*Orchesella cincta*
ODN02580.1	*SmydA-2*	SmydA	*Orchesella cincta*
ODN04487.1	*SmydA-2*	SmydA	*Orchesella cincta*
ODM91338.1	*SmydA-3*	SmydA	*Orchesella cincta*

ODM98666.1	*SmydA-4*	SmydA	*Orchesella cincta*
ODM87448.1	*SmydA-4*	SmydA	*Orchesella cincta*
ODM90870.1	*SmydA-4*	SmydA	*Orchesella cincta*
ODM95333.1	*SmydA-4*	SmydA	*Orchesella cincta*
ODM99451.1	*SmydA-4*	SmydA	*Orchesella cincta*
ODN02118.1	*SmydA-4*	SmydA	*Orchesella cincta*
ODN02948.1	*SmydA-4*	SmydA	*Orchesella cincta*
ODM90778.1	*SmydA-5*	SmydA	*Orchesella cincta*
ODM93675.1	*SmydA-5*	SmydA	*Orchesella cincta*
ODM97389.1	*Su(var)3-9*	Suv	*Orchesella cincta*
ODM88213.1	*Su(var)3-9*	Suv	*Orchesella cincta*
ODM88636.1	*Su(var)3-9*	Suv	*Orchesella cincta*
ODM88637.1	*Su(var)3-9*	Suv	*Orchesella cincta*
ODM96262.1	*Su(var)3-9*	Suv	*Orchesella cincta*
ODM98559.1	*Su(var)3-9*	Suv	*Orchesella cincta*
ODM99256.1	*Su(var)3-9*	Suv	*Orchesella cincta*
ODN04495.1	*Su(var)3-9*	Suv	*Orchesella cincta*
ODN06241.1	*Su(var)3-9*	Suv	*Orchesella cincta*
ODN00513.1	*trr*	Trx	*Orchesella cincta*
ODN04290.1	*trx*	Trx	*Orchesella cincta*
ODM95869.1	*upSET(MLL5)*	-	*Orchesella cincta*
OABI008652	-	-	*Orussus abietinus*
OABI010009	*ash1*	Ash	*Orussus abietinus*
OABI000954	*Blimp-1*	PRDM	*Orussus abietinus*
OABI000487	*egg*	Suv	*Orussus abietinus*
OABI009712	*Ez*	Ez	*Orussus abietinus*
OABI010588	*G9a*	Suv	*Orussus abietinus*
OABI008210	*Hmt4-20*	-	*Orussus abietinus*
OABI008285	*Set1*	Trx	*Orussus abietinus*
OABI008237	*Set2*	Ash	*Orussus abietinus*
OABI000345	*Set8*	-	*Orussus abietinus*
OABI006484	*setd*	SETD	*Orussus abietinus*
OABI006487	*setd*	SETD	*Orussus abietinus*
OABI007310	*Smyd3*	SMYD	*Orussus abietinus*
OABI005724	*Smyd4-1*	SMYD	*Orussus abietinus*
OABI001704	*Smyd4-3*	SMYD	*Orussus abietinus*
OABI010928	*Smyd4-4*	SMYD	*Orussus abietinus*
OABI003492	*Smyd4-4*	SMYD	*Orussus abietinus*
OABI007980	*Smyd4-4*	SMYD	*Orussus abietinus*
OABI007981	*Smyd4-4*	SMYD	*Orussus abietinus*
OABI007993	*Smyd4-4*	SMYD	*Orussus abietinus*
OABI007995	*Smyd4-4*	SMYD	*Orussus abietinus*
OABI008791	*Smyd4-4*	SMYD	*Orussus abietinus*
OABI002192	*Smyd5*	SMYD	*Orussus abietinus*

OABI008617	*SmydA-3*	SmydA	*Orussus abietinus*
OABI001072	*SmydA-5*	SmydA	*Orussus abietinus*
OABI001784	*SmydA-8*	SmydA	*Orussus abietinus*
OABI000427	*suv*	Suv	*Orussus abietinus*
OABI010801	*trr*	Trx	*Orussus abietinus*
OABI000651	*trx*	Trx	*Orussus abietinus*
OABI004271	*upSET(MLL5)*	-	*Orussus abietinus*
KRT79956.1	-	-	*Oryctes borbonicus*
KRT83921.1	*ash1*	Ash	*Oryctes borbonicus*
KRT86642.1	*Blimp-1*	PRDM	*Oryctes borbonicus*
KRT84106.1	*egg*	Suv	*Oryctes borbonicus*
KRT84107.1	*egg*	Suv	*Oryctes borbonicus*
KRT78790.1	*G9a*	Suv	*Oryctes borbonicus*
KRT81536.1	*Hmt4-20*	-	*Oryctes borbonicus*
KRT86397.1	*Mes-4*	Ash	*Oryctes borbonicus*
KRT81143.1	*Set1*	Trx	*Oryctes borbonicus*
KRT79337.1	*Set2*	Ash	*Oryctes borbonicus*
KRT86230.1	*Set8*	-	*Oryctes borbonicus*
KRT82960.1	*setd*	SETD	*Oryctes borbonicus*
KRT79564.1	*Smyd3*	SMYD	*Oryctes borbonicus*
KRT82312.1	*Smyd4-2*	SMYD	*Oryctes borbonicus*
KRT79853.1	*Smyd4-3*	SMYD	*Oryctes borbonicus*
KRT78451.1	*Smyd4-3*	SMYD	*Oryctes borbonicus*
KRT85673.1	*Smyd4-4*	SMYD	*Oryctes borbonicus*
KRT80014.1	*Smyd5*	SMYD	*Oryctes borbonicus*
KRT84042.1	*SmydA-1*	SmydA	*Oryctes borbonicus*
KRT82251.1	*SmydA-3*	SmydA	*Oryctes borbonicus*
KRT79705.1	*SmydA-4*	SmydA	*Oryctes borbonicus*
KRT80077.1	*SmydA-4*	SmydA	*Oryctes borbonicus*
KRT84745.1	*SmydA-5*	SmydA	*Oryctes borbonicus*
KRT80385.1	*upSET(MLL5)*	-	*Oryctes borbonicus*
PVEN003514	-	-	*Pachypsylla venusta*
PVEN008929	-	-	*Pachypsylla venusta*
PVEN003699	*ash1*	Ash	*Pachypsylla venusta*
PVEN006306	*Blimp-1*	PRDM	*Pachypsylla venusta*
PVEN009898	*Blimp-1*	PRDM	*Pachypsylla venusta*
PVEN005308	*egg*	Suv	*Pachypsylla venusta*
PVEN003345	*Ez*	Ez	*Pachypsylla venusta*
PVEN007112	*Hmt4-20*	-	*Pachypsylla venusta*
PVEN009344	*Set2*	Ash	*Pachypsylla venusta*
PVEN005377	*Set8*	-	*Pachypsylla venusta*
PVEN004854	*setd*	SETD	*Pachypsylla venusta*
PVEN004408	*Smyd4-3*	SMYD	*Pachypsylla venusta*
PVEN001191	*SmydA-2*	SmydA	*Pachypsylla venusta*

PVEN012478	*SmydA-3*	SmydA	*Pachypsylla venusta*
PVEN008317	*SmydA-5*	SmydA	*Pachypsylla venusta*
PVEN004358	*SmydA-8*	SmydA	*Pachypsylla venusta*
PVEN001009	*Su(var)3-9*	Suv	*Pachypsylla venusta*
PVEN003916	*Su(var)3-9*	Suv	*Pachypsylla venusta*
PVEN007921	*trx*	Trx	*Pachypsylla venusta*
PVEN007586	*upSET(MLL5)*	-	*Pachypsylla venusta*
PpolytesGene0010193	-	-	*Papilio polytes*
PpolytesGene0003344	*ash1*	Ash	*Papilio polytes*
PpolytesGene0002656	*Blimp-1*	PRDM	*Papilio polytes*
PpolytesGene0001401	*egg*	Suv	*Papilio polytes*
PpolytesGene0007131	*Hmt4-20*	-	*Papilio polytes*
PpolytesGene0001743	*Mes-4*	Ash	*Papilio polytes*
PpolytesGene0009812	*Set1*	Trx	*Papilio polytes*
PpolytesGene0006463	*Set2*	Ash	*Papilio polytes*
PpolytesGene0009737	*Set8*	-	*Papilio polytes*
PpolytesGene0003757	*setd*	SETD	*Papilio polytes*
PpolytesGene0011948	*Smyd4-1*	SMYD	*Papilio polytes*
PpolytesGene0010418	*Smyd4-2*	SMYD	*Papilio polytes*
PpolytesGene0000390	*Smyd4-3*	SMYD	*Papilio polytes*
PpolytesGene0002992	*Smyd4-4*	SMYD	*Papilio polytes*
PpolytesGene0009103	*Smyd4-4*	SMYD	*Papilio polytes*
PpolytesGene0002500	*Smyd5*	SMYD	*Papilio polytes*
PpolytesGene0009185	*SmydA-1*	SmydA	*Papilio polytes*
PpolytesGene0009252	*SmydA-2*	SmydA	*Papilio polytes*
PpolytesGene0009269	*SmydA-3*	SmydA	*Papilio polytes*
PpolytesGene0009184	*SmydA-4*	SmydA	*Papilio polytes*
PpolytesGene0009251	*SmydA-5*	SmydA	*Papilio polytes*
PpolytesGene0009274	*SmydA-7*	SmydA	*Papilio polytes*
PpolytesGene0007698	*SmydA-8*	SmydA	*Papilio polytes*
PpolytesGene0008985	*suv*	Suv	*Papilio polytes*
PpolytesGene0003883	*trx*	Trx	*Papilio polytes*
PpolytesGene0007426	*upSET(MLL5)*	-	*Papilio polytes*
PxuthusGene0005385	*ash1*	Ash	*Papilio xuthus*
PxuthusGene0000342	*Blimp-1*	PRDM	*Papilio xuthus*
PxuthusGene0001006	*egg*	Suv	*Papilio xuthus*
PxuthusGene0003790	*Ez*	Ez	*Papilio xuthus*
PxuthusGene0007252	*G9a*	Suv	*Papilio xuthus*
PxuthusGene0001837	*Hmt4-20*	-	*Papilio xuthus*
PxuthusGene0007920	*Mes-4*	Ash	*Papilio xuthus*
PxuthusGene0006641	*Set1*	Trx	*Papilio xuthus*
PxuthusGene0006285	*Set2*	Ash	*Papilio xuthus*
PxuthusGene0006529	*Set8*	-	*Papilio xuthus*
PxuthusGene0003668	*setd*	SETD	*Papilio xuthus*

PxuthusGene0012605	*Smyd4-1*	SMYD	*Papilio xuthus*
PxuthusGene0009507	*Smyd4-2*	SMYD	*Papilio xuthus*
PxuthusGene0007183	*Smyd4-3*	SMYD	*Papilio xuthus*
PxuthusGene0011921	*Smyd4-4*	SMYD	*Papilio xuthus*
PxuthusGene0008942	*Smyd4-4*	SMYD	*Papilio xuthus*
PxuthusGene0003431	*Smyd5*	SMYD	*Papilio xuthus*
PxuthusGene0011724	*SmydA-2*	SmydA	*Papilio xuthus*
PxuthusGene0011744	*SmydA-3*	SmydA	*Papilio xuthus*
PxuthusGene0011655	*SmydA-4*	SmydA	*Papilio xuthus*
PxuthusGene0011723	*SmydA-5*	SmydA	*Papilio xuthus*
PxuthusGene0011749	*SmydA-6*	SmydA	*Papilio xuthus*
PxuthusGene0006228	*SmydA-8*	SmydA	*Papilio xuthus*
PxuthusGene0003277	*Su(var)3-9*	Suv	*Papilio xuthus*
PxuthusGene0006868	*suv*	Suv	*Papilio xuthus*
PxuthusGene0008105	*trr*	Trx	*Papilio xuthus*
PxuthusGene0000539	*upSET(MLL5)*	-	*Papilio xuthus*
PTEP002912	-	-	*Parasteatoda tepidariorum*
PTEP002422	*ash1*	Ash	*Parasteatoda tepidariorum*
PTEP023170	*Blimp-1*	PRDM	*Parasteatoda tepidariorum*
PTEP012295	*Blimp-1*	PRDM	*Parasteatoda tepidariorum*
PTEP012300	*Blimp-1*	PRDM	*Parasteatoda tepidariorum*
PTEP012301	*Blimp-1*	PRDM	*Parasteatoda tepidariorum*
PTEP019733	*Blimp-1*	PRDM	*Parasteatoda tepidariorum*
PTEP020724	*Blimp-1*	PRDM	*Parasteatoda tepidariorum*
PTEP020725	*Blimp-1*	PRDM	*Parasteatoda tepidariorum*
PTEP020733	*Blimp-1*	PRDM	*Parasteatoda tepidariorum*
PTEP028424	*Blimp-1*	PRDM	*Parasteatoda tepidariorum*
PTEP028799	*Blimp-1*	PRDM	*Parasteatoda tepidariorum*
PTEP010963	*egg*	Suv	*Parasteatoda tepidariorum*
PTEP025196	*Ez*	Ez	*Parasteatoda tepidariorum*
PTEP000605	*G9a*	Suv	*Parasteatoda tepidariorum*
PTEP004339	*G9a*	Suv	*Parasteatoda tepidariorum*
PTEP013699	*Hmt4-20*	-	*Parasteatoda tepidariorum*
PTEP015414	*Mes-4*	Ash	*Parasteatoda tepidariorum*
PTEP000233	*Mes-4*	Ash	*Parasteatoda tepidariorum*
PTEP017281	*Set1*	Trx	*Parasteatoda tepidariorum*
PTEP003651	*Set2*	Ash	*Parasteatoda tepidariorum*
PTEP012349	*Set2*	Ash	*Parasteatoda tepidariorum*
PTEP022231	*Set8*	-	*Parasteatoda tepidariorum*
PTEP010279	*setd*	SETD	*Parasteatoda tepidariorum*
PTEP006874	*Smyd3*	SMYD	*Parasteatoda tepidariorum*
PTEP017494	*Su(var)3-9*	Suv	*Parasteatoda tepidariorum*
PTEP012357	*suv*	Suv	*Parasteatoda tepidariorum*
PTEP020401	*trr*	Trx	*Parasteatoda tepidariorum*

PTEP006665	*trx*	Trx	*Parasteatoda tepidariorum*
PHUM191320	-	-	*Pediculus humanus*
PHUM244720	*ash1*	Ash	*Pediculus humanus*
PHUM607020	*Blimp-1*	PRDM	*Pediculus humanus*
PHUM415050	*egg*	Suv	*Pediculus humanus*
PHUM294210	*Ez*	Ez	*Pediculus humanus*
PHUM447810	*G9a*	Suv	*Pediculus humanus*
PHUM503290	*Hmt4-20*	-	*Pediculus humanus*
PHUM433670	*Mes-4*	Ash	*Pediculus humanus*
PHUM240750	*Set1*	Trx	*Pediculus humanus*
PHUM463920	*Set2*	Ash	*Pediculus humanus*
PHUM315980	*Set8*	-	*Pediculus humanus*
PHUM131480	*setd*	SETD	*Pediculus humanus*
PHUM489090	*Smyd3*	SMYD	*Pediculus humanus*
PHUM212930	*Smyd4-3*	SMYD	*Pediculus humanus*
PHUM132280	*Smyd4-3*	SMYD	*Pediculus humanus*
PHUM126850	*Smyd4-4*	SMYD	*Pediculus humanus*
PHUM105860	*Smyd5*	SMYD	*Pediculus humanus*
PHUM105850	*Smyd5*	SMYD	*Pediculus humanus*
PHUM128680	*SmydA-2*	SmydA	*Pediculus humanus*
PHUM472490	*SmydA-3*	SmydA	*Pediculus humanus*
PHUM331980	*SmydA-4*	SmydA	*Pediculus humanus*
PHUM472480	*SmydA-4*	SmydA	*Pediculus humanus*
PHUM128690	*SmydA-5*	SmydA	*Pediculus humanus*
PHUM423590	*Su(var)3-9*	Suv	*Pediculus humanus*
PHUM451270	*suv*	Suv	*Pediculus humanus*
PHUM411800	*trr*	Trx	*Pediculus humanus*
PHUM079870	*trx*	Trx	*Pediculus humanus*
PHUM494030	*upSET(MLL5)*	-	*Pediculus humanus*
PPAI004765	*ash1*	Ash	*Phlebotomus papatasi*
PPAI009638	*egg*	Suv	*Phlebotomus papatasi*
PPAI008768	*Ez*	Ez	*Phlebotomus papatasi*
PPAI004354	*G9a*	Suv	*Phlebotomus papatasi*
PPAI004950	*Mes-4*	Ash	*Phlebotomus papatasi*
PPAI008303	*Set1*	Trx	*Phlebotomus papatasi*
PPAI003533	*Set2*	Ash	*Phlebotomus papatasi*
PPAI008613	*Set2*	Ash	*Phlebotomus papatasi*
PPAI006291	*Set8*	-	*Phlebotomus papatasi*
PPAI009538	*setd*	SETD	*Phlebotomus papatasi*
PPAI011158	*Smyd3*	SMYD	*Phlebotomus papatasi*
PPAI003744	*Smyd4-1*	SMYD	*Phlebotomus papatasi*
PPAI000197	*Smyd4-2*	SMYD	*Phlebotomus papatasi*
PPAI003136	*Smyd4-3*	SMYD	*Phlebotomus papatasi*
PPAI005666	*Smyd5*	SMYD	*Phlebotomus papatasi*

PPAI003492	*SmydA-1*	SmydA	*Phlebotomus papatasi*
PPAI002804	*SmydA-2*	SmydA	*Phlebotomus papatasi*
PPAI003320	*SmydA-3*	SmydA	*Phlebotomus papatasi*
PPAI003491	*SmydA-4*	SmydA	*Phlebotomus papatasi*
PPAI002546	*SmydA-8*	SmydA	*Phlebotomus papatasi*
PPAI008878	*suv*	Suv	*Phlebotomus papatasi*
PPAI005537	*trr*	Trx	*Phlebotomus papatasi*
PPAI007688	*trx*	Trx	*Phlebotomus papatasi*
Px003149.1	-	-	*Plutella xylostella*
Px012028.1	-	-	*Plutella xylostella*
Px012304.1	*ash1*	Ash	*Plutella xylostella*
Px011637.1	*Blimp-1*	PRDM	*Plutella xylostella*
Px010030.1	*egg*	Suv	*Plutella xylostella*
Px012884.1	*egg*	Suv	*Plutella xylostella*
Px012980.1	*G9a*	Suv	*Plutella xylostella*
Px002764.1	*G9a*	Suv	*Plutella xylostella*
Px008524.1	*Mes-4*	Ash	*Plutella xylostella*
Px008525.1	*Mes-4*	Ash	*Plutella xylostella*
Px013558.1	*Set1*	Trx	*Plutella xylostella*
Px014783.1	*Set1*	Trx	*Plutella xylostella*
Px003495.1	*Set2*	Ash	*Plutella xylostella*
Px013563.1	*Set8*	-	*Plutella xylostella*
Px002036.1	*setd*	SETD	*Plutella xylostella*
Px002038.1	*setd*	SETD	*Plutella xylostella*
Px006948.1	*setd*	SETD	*Plutella xylostella*
Px006949.1	*setd*	SETD	*Plutella xylostella*
Px001474.1	*Smyd4-1*	SMYD	*Plutella xylostella*
Px002177.2	*Smyd4-2*	SMYD	*Plutella xylostella*
Px008749.1	*Smyd4-3*	SMYD	*Plutella xylostella*
Px012029.1	*Smyd4-4*	SMYD	*Plutella xylostella*
Px009260.1	*Smyd4-4*	SMYD	*Plutella xylostella*
Px001029.1	*SmydA-1*	SmydA	*Plutella xylostella*
Px015362.1	*SmydA-1*	SmydA	*Plutella xylostella*
Px007787.2	*SmydA-2*	SmydA	*Plutella xylostella*
Px007644.1	*SmydA-3*	SmydA	*Plutella xylostella*
Px015361.3	*SmydA-4*	SmydA	*Plutella xylostella*
Px003408.1	*SmydA-4*	SmydA	*Plutella xylostella*
Px007788.1	*SmydA-5*	SmydA	*Plutella xylostella*
Px007648.2	*SmydA-6*	SmydA	*Plutella xylostella*
Px014557.1	*SmydA-8*	SmydA	*Plutella xylostella*
Px011535.1	*Su(var)3-9*	Suv	*Plutella xylostella*
Px000378.1	*trr*	Trx	*Plutella xylostella*
Px000383.1	*trr*	Trx	*Plutella xylostella*
Px009181.1	*trr*	Trx	*Plutella xylostella*

Px015073.1	*trx*	Trx	*Plutella xylostella*
Px014006.5	*upSET(MLL5)*	-	*Plutella xylostella*
PB19591	-	-	*Pogonomyrmex barbatus*
PB21545	*egg*	Suv	*Pogonomyrmex barbatus*
PB17647	*Ez*	Ez	*Pogonomyrmex barbatus*
PB16229	*G9a*	Suv	*Pogonomyrmex barbatus*
PB19450	*Hmt4-20*	-	*Pogonomyrmex barbatus*
PB21188	*Mes-4*	Ash	*Pogonomyrmex barbatus*
PB20646	*Set1*	Trx	*Pogonomyrmex barbatus*
PB15902	*Set2*	Ash	*Pogonomyrmex barbatus*
PB18656	*Set8*	-	*Pogonomyrmex barbatus*
PB17917	*setd*	SETD	*Pogonomyrmex barbatus*
PB17920	*setd*	SETD	*Pogonomyrmex barbatus*
PB17474	*Smyd4-1*	SMYD	*Pogonomyrmex barbatus*
PB19875	*Smyd4-2*	SMYD	*Pogonomyrmex barbatus*
PB11858	*Smyd4-3*	SMYD	*Pogonomyrmex barbatus*
PB22652	*Smyd4-3*	SMYD	*Pogonomyrmex barbatus*
PB12085	*Smyd5*	SMYD	*Pogonomyrmex barbatus*
PB21709	*SmydA-3*	SmydA	*Pogonomyrmex barbatus*
PB21710	*SmydA-4*	SmydA	*Pogonomyrmex barbatus*
PB18400	*SmydA-5*	SmydA	*Pogonomyrmex barbatus*
PB25817	*SmydA-8*	SmydA	*Pogonomyrmex barbatus*
PB17547	*Su(var)3-9*	Suv	*Pogonomyrmex barbatus*
PB16645	*suv*	Suv	*Pogonomyrmex barbatus*
PB12022	*trr*	Trx	*Pogonomyrmex barbatus*
PB13115	*trx*	Trx	*Pogonomyrmex barbatus*
PB20519	*upSET(MLL5)*	-	*Pogonomyrmex barbatus*
XP_015191195.1	*Blimp-1*	PRDM	*Polistes dominula*
XP_015185231.1	*egg*	Suv	*Polistes dominula*
XP_015189397.1	*Ez*	Ez	*Polistes dominula*
XP_015177385.1	*G9a*	Suv	*Polistes dominula*
XP_015178802.1	*Hmt4-20*	-	*Polistes dominula*
XP_015186803.1	*Set2*	Ash	*Polistes dominula*
XP_015188928.1	*Set8*	-	*Polistes dominula*
XP_015176838.1	*setd*	SETD	*Polistes dominula*
XP_015181174.1	*Smyd4-2*	SMYD	*Polistes dominula*
XP_015176876.1	*Smyd4-2*	SMYD	*Polistes dominula*
XP_015187287.1	*Smyd4-3*	SMYD	*Polistes dominula*
XP_015175405.1	*Smyd4-3*	SMYD	*Polistes dominula*
XP_015177541.1	*Smyd4-3*	SMYD	*Polistes dominula*
XP_015185089.1	*Smyd5*	SMYD	*Polistes dominula*
XP_015179499.1	*SmydA-3*	SmydA	*Polistes dominula*
XP_015175183.1	*SmydA-4*	SmydA	*Polistes dominula*
XP_015178450.1	*SmydA-5*	SmydA	*Polistes dominula*

XP_015174257.1	*SmydA-8*	SmydA	*Polistes dominula*
XP_015174103.1	*Su(var)3-9*	Suv	*Polistes dominula*
XP_015190270.1	*suv*	Suv	*Polistes dominula*
XP_015177420.1	*trr*	Trx	*Polistes dominula*
XP_015187057.1	*trx*	Trx	*Polistes dominula*
RPRC001120	*ash1*	Ash	*Rhodnius prolixus*
RPRC009085	*Ez*	Ez	*Rhodnius prolixus*
RPRC003115	*G9a*	Suv	*Rhodnius prolixus*
RPRC002749	*Hmt4-20*	-	*Rhodnius prolixus*
RPRC001493	*Set1*	Trx	*Rhodnius prolixus*
RPRC001422	*Set2*	Ash	*Rhodnius prolixus*
RPRC006378	*Set8*	-	*Rhodnius prolixus*
RPRC001600	*setd*	SETD	*Rhodnius prolixus*
RPRC003722	*Smyd3*	SMYD	*Rhodnius prolixus*
RPRC005705	*Smyd4-2*	SMYD	*Rhodnius prolixus*
RPRC010144	*Smyd4-3*	SMYD	*Rhodnius prolixus*
RPRC012281	*Smyd4-3*	SMYD	*Rhodnius prolixus*
RPRC014880	*Smyd4-4*	SMYD	*Rhodnius prolixus*
RPRC003079	*Smyd5*	SMYD	*Rhodnius prolixus*
RPRC010307	*SmydA-1*	SmydA	*Rhodnius prolixus*
RPRC008683	*SmydA-3*	SmydA	*Rhodnius prolixus*
RPRC010311	*SmydA-4*	SmydA	*Rhodnius prolixus*
RPRC001060	*SmydA-5*	SmydA	*Rhodnius prolixus*
RPRC013812	*suv*	Suv	*Rhodnius prolixus*
RPRC003178	*trr*	Trx	*Rhodnius prolixus*
KPM10847.1	*ash1*	Ash	*Sarcoptes scabiei*
KPM10339.1	*Blimp-1*	PRDM	*Sarcoptes scabiei*
KPM04871.1	*egg*	Suv	*Sarcoptes scabiei*
KPM09905.1	*Ez*	Ez	*Sarcoptes scabiei*
KPM04996.1	*Hmt4-20*	-	*Sarcoptes scabiei*
KPM03216.1	*Mes-4*	Ash	*Sarcoptes scabiei*
KPM04444.1	*Set1*	Trx	*Sarcoptes scabiei*
KPM10660.1	*Set2*	Ash	*Sarcoptes scabiei*
KPM10949.1	*Set8*	-	*Sarcoptes scabiei*
KPM04695.1	*Smyd3*	SMYD	*Sarcoptes scabiei*
KPM05877.1	*Smyd3*	SMYD	*Sarcoptes scabiei*
KPM11104.1	*Smyd3*	SMYD	*Sarcoptes scabiei*
KPM06712.1	*Smyd4-3*	SMYD	*Sarcoptes scabiei*
KPM06989.1	*Smyd4-3*	SMYD	*Sarcoptes scabiei*
KPM03385.1	*Smyd5*	SMYD	*Sarcoptes scabiei*
KPM05617.1	*Smyd5*	SMYD	*Sarcoptes scabiei*
KPL93604.1	*trr*	Trx	*Sarcoptes scabiei*
SI2.2.0_12919	-	-	*Solenopsis invicta*
SI2.2.0_07690	*ash1*	Ash	*Solenopsis invicta*

SI2.2.0_00981	*egg*	Suv	*Solenopsis invicta*
SI2.2.0_09656	*Ez*	Ez	*Solenopsis invicta*
SI2.2.0_06678	*Mes-4*	Ash	*Solenopsis invicta*
SI2.2.0_14637	*Set1*	Trx	*Solenopsis invicta*
SI2.2.0_04653	*Set2*	Ash	*Solenopsis invicta*
SI2.2.0_02694	*Set8*	-	*Solenopsis invicta*
SI2.2.0_15998	*setd*	SETD	*Solenopsis invicta*
SI2.2.0_02250	*Smyd4-3*	SMYD	*Solenopsis invicta*
SI2.2.0_10758	*Smyd5*	SMYD	*Solenopsis invicta*
SI2.2.0_08812	*SmydA-3*	SmydA	*Solenopsis invicta*
SI2.2.0_10209	*SmydA-4*	SmydA	*Solenopsis invicta*
SI2.2.0_07869	*SmydA-5*	SmydA	*Solenopsis invicta*
SI2.2.0_09986	*SmydA-8*	SmydA	*Solenopsis invicta*
SI2.2.0_12284	*Su(var)3-9*	Suv	*Solenopsis invicta*
SI2.2.0_00751	*suv*	Suv	*Solenopsis invicta*
SI2.2.0_09282	*trr*	Trx	*Solenopsis invicta*
SI2.2.0_07789	*trx*	Trx	*Solenopsis invicta*
SI2.2.0_04884	*upSET(MLL5)*	-	*Solenopsis invicta*
KFM72222.1	-	-	*Stegodyphus mimosarum*
KFM81063.1	-	-	*Stegodyphus mimosarum*
KFM82051.1	-	-	*Stegodyphus mimosarum*
KFM74571.1	*ash1*	Ash	*Stegodyphus mimosarum*
KFM78153.1	*Blimp-1*	PRDM	*Stegodyphus mimosarum*
KFM76516.1	*Blimp-1*	PRDM	*Stegodyphus mimosarum*
KFM77550.1	*egg*	Suv	*Stegodyphus mimosarum*
KFM67407.1	*Ez*	Ez	*Stegodyphus mimosarum*
KFM59010.1	*G9a*	Suv	*Stegodyphus mimosarum*
KFM73887.1	*G9a*	Suv	*Stegodyphus mimosarum*
KFM79732.1	*Hmt4-20*	-	*Stegodyphus mimosarum*
KFM70139.1	*Hmt4-20*	-	*Stegodyphus mimosarum*
KFM62539.1	*Mes-4*	Ash	*Stegodyphus mimosarum*
KFM77664.1	*Set1*	Trx	*Stegodyphus mimosarum*
KFM70004.1	*Set2*	Ash	*Stegodyphus mimosarum*
KFM72722.1	*Set8*	-	*Stegodyphus mimosarum*
KFM79046.1	*setd*	SETD	*Stegodyphus mimosarum*
KFM79258.1	*Smyd3*	SMYD	*Stegodyphus mimosarum*
KFM73274.1	*Smyd4-3*	SMYD	*Stegodyphus mimosarum*
KFM65223.1	*Smyd5*	SMYD	*Stegodyphus mimosarum*
KFM59720.1	*suv*	Suv	*Stegodyphus mimosarum*
KFM63416.1	*trr*	Trx	*Stegodyphus mimosarum*
SCAU010542	-	-	*Stomoxys calcitrans*
SCAU008949	-	-	*Stomoxys calcitrans*
SCAU008374	*ash1*	Ash	*Stomoxys calcitrans*
SCAU008763	*Blimp-1*	PRDM	*Stomoxys calcitrans*

SCAU004069	*egg*	Suv	*Stomoxys calcitrans*
SCAU005606	*Ez*	Ez	*Stomoxys calcitrans*
SCAU001322	*G9a*	Suv	*Stomoxys calcitrans*
SCAU006850	*Hmt4-20*	-	*Stomoxys calcitrans*
SCAU008248	*Mes-4*	Ash	*Stomoxys calcitrans*
SCAU012005	*Set1*	Trx	*Stomoxys calcitrans*
SCAU003593	*Set2*	Ash	*Stomoxys calcitrans*
SCAU009186	*Set8*	-	*Stomoxys calcitrans*
SCAU014524	*setd*	SETD	*Stomoxys calcitrans*
SCAU006212	*Smyd4-1*	SMYD	*Stomoxys calcitrans*
SCAU000793	*Smyd4-2*	SMYD	*Stomoxys calcitrans*
SCAU016375	*Smyd4-3*	SMYD	*Stomoxys calcitrans*
SCAU005169	*Smyd4-4*	SMYD	*Stomoxys calcitrans*
SCAU008975	*Smyd5*	SMYD	*Stomoxys calcitrans*
SCAU007742	*SmydA-1*	SmydA	*Stomoxys calcitrans*
SCAU015663	*SmydA-2*	SmydA	*Stomoxys calcitrans*
SCAU008859	*SmydA-3*	SmydA	*Stomoxys calcitrans*
SCAU003400	*SmydA-4*	SmydA	*Stomoxys calcitrans*
SCAU005082	*SmydA-5*	SmydA	*Stomoxys calcitrans*
SCAU000935	*SmydA-6*	SmydA	*Stomoxys calcitrans*
SCAU010581	*SmydA-6*	SmydA	*Stomoxys calcitrans*
SCAU011677	*SmydA-6*	SmydA	*Stomoxys calcitrans*
SCAU004225	*SmydA-7*	SmydA	*Stomoxys calcitrans*
SCAU010775	*SmydA-8*	SmydA	*Stomoxys calcitrans*
SCAU006646	*SmydA-9*	SmydA	*Stomoxys calcitrans*
SCAU007743	*trr*	Trx	*Stomoxys calcitrans*
SCAU008744	*trx*	Trx	*Stomoxys calcitrans*
SCAU009601	*upSET(MLL5)*	-	*Stomoxys calcitrans*
03g02610	*ash1*	Ash	*Tetranychus urticae*
15g00080	*Blimp-1*	PRDM	*Tetranychus urticae*
03g02220	*egg*	Suv	*Tetranychus urticae*
07g01000	*egg*	Suv	*Tetranychus urticae*
28g01660	*egg*	Suv	*Tetranychus urticae*
05g08610	*Ez*	Ez	*Tetranychus urticae*
15g02370	*Ez*	Ez	*Tetranychus urticae*
02g11280	*Hmt4-20*	-	*Tetranychus urticae*
02g03670	*Mes-4*	Ash	*Tetranychus urticae*
07g03190	*Set1*	Trx	*Tetranychus urticae*
15g00050	*Set2*	Ash	*Tetranychus urticae*
20g02320	*Set8*	-	*Tetranychus urticae*
20g02380	*Set8*	-	*Tetranychus urticae*
07g04000	*setd*	SETD	*Tetranychus urticae*
12g03090	*Smyd3*	SMYD	*Tetranychus urticae*
02g04130	*Smyd4-2*	SMYD	*Tetranychus urticae*

04g03960	*Smyd4-2*	SMYD	*Tetranychus urticae*
06g06280	*Smyd4-2*	SMYD	*Tetranychus urticae*
06g06270	*Smyd4-3*	SMYD	*Tetranychus urticae*
08g00440	*Smyd4-3*	SMYD	*Tetranychus urticae*
02g06270	*Smyd4-4*	SMYD	*Tetranychus urticae*
04g08120	*Su(var)3-9*	Suv	*Tetranychus urticae*
17g01640	*trr*	Trx	*Tetranychus urticae*
TCALIF_00296	-	-	*Tigriopus californicus*
TCALIF_00673	-	-	*Tigriopus californicus*
TCALIF_00901	-	-	*Tigriopus californicus*
TCALIF_06487	-	-	*Tigriopus californicus*
TCALIF_07919	-	-	*Tigriopus californicus*
TCALIF_08703	-	-	*Tigriopus californicus*
TCALIF_09504	-	-	*Tigriopus californicus*
TCALIF_10816	-	-	*Tigriopus californicus*
TCALIF_13437	-	-	*Tigriopus californicus*
TCALIF_04842	-	-	*Tigriopus californicus*
TCALIF_08427	-	-	*Tigriopus californicus*
TCALIF_08822	-	-	*Tigriopus californicus*
TCALIF_11522	-	-	*Tigriopus californicus*
TCALIF_02232	-	-	*Tigriopus californicus*
TCALIF_03221	-	-	*Tigriopus californicus*
TCALIF_03641	-	-	*Tigriopus californicus*
TCALIF_03794	-	-	*Tigriopus californicus*
TCALIF_05025	-	-	*Tigriopus californicus*
TCALIF_08162	-	-	*Tigriopus californicus*
TCALIF_11823	-	-	*Tigriopus californicus*
TCALIF_12339	-	-	*Tigriopus californicus*
TCALIF_12461	-	-	*Tigriopus californicus*
TCALIF_12483	-	-	*Tigriopus californicus*
TCALIF_03449	*ash1*	Ash	*Tigriopus californicus*
TCALIF_10212	*Blimp-1*	PRDM	*Tigriopus californicus*
TCALIF_09221	*egg*	Suv	*Tigriopus californicus*
TCALIF_01325	*Ez*	Ez	*Tigriopus californicus*
TCALIF_11585	*G9a*	Suv	*Tigriopus californicus*
TCALIF_11332	*Hmt4-20*	-	*Tigriopus californicus*
TCALIF_05036	*Mes-4*	Ash	*Tigriopus californicus*
TCALIF_08908	*Set1*	Trx	*Tigriopus californicus*
TCALIF_02122	*Set2*	Ash	*Tigriopus californicus*
TCALIF_01201	*Set8*	-	*Tigriopus californicus*
TCALIF_09968	*Set8*	-	*Tigriopus californicus*
TCALIF_04636	*Smyd3*	SMYD	*Tigriopus californicus*
TCALIF_08718	*Smyd4-2*	SMYD	*Tigriopus californicus*
TCALIF_01669	*Smyd4-2*	SMYD	*Tigriopus californicus*

TCALIF_02034	*Smyd4-2*	SMYD	*Tigriopus californicus*
TCALIF_08951	*Smyd4-3*	SMYD	*Tigriopus californicus*
TCALIF_00745	*Smyd4-3*	SMYD	*Tigriopus californicus*
TCALIF_03792	*Smyd4-3*	SMYD	*Tigriopus californicus*
TCALIF_06026	*Smyd4-3*	SMYD	*Tigriopus californicus*
TCALIF_07236	*Smyd4-3*	SMYD	*Tigriopus californicus*
TCALIF_07774	*Smyd4-3*	SMYD	*Tigriopus californicus*
TCALIF_09013	*Smyd4-3*	SMYD	*Tigriopus californicus*
TCALIF_10785	*Smyd4-3*	SMYD	*Tigriopus californicus*
TCALIF_11285	*Smyd4-3*	SMYD	*Tigriopus californicus*
TCALIF_00173	*Smyd5*	SMYD	*Tigriopus californicus*
TCALIF_05643	*SmydA-1*	SmydA	*Tigriopus californicus*
TCALIF_02745	*SmydA-2*	SmydA	*Tigriopus californicus*
TCALIF_03170	*SmydA-2*	SmydA	*Tigriopus californicus*
TCALIF_04193	*SmydA-2*	SmydA	*Tigriopus californicus*
TCALIF_06488	*SmydA-2*	SmydA	*Tigriopus californicus*
TCALIF_06636	*SmydA-2*	SmydA	*Tigriopus californicus*
TCALIF_09550	*SmydA-2*	SmydA	*Tigriopus californicus*
TCALIF_09913	*SmydA-2*	SmydA	*Tigriopus californicus*
TCALIF_10822	*SmydA-2*	SmydA	*Tigriopus californicus*
TCALIF_01118	*SmydA-3*	SmydA	*Tigriopus californicus*
TCALIF_06674	*SmydA-3*	SmydA	*Tigriopus californicus*
TCALIF_07921	*SmydA-3*	SmydA	*Tigriopus californicus*
TCALIF_09525	*SmydA-3*	SmydA	*Tigriopus californicus*
TCALIF_07312	*SmydA-4*	SmydA	*Tigriopus californicus*
TCALIF_00957	*SmydA-4*	SmydA	*Tigriopus californicus*
TCALIF_01525	*SmydA-4*	SmydA	*Tigriopus californicus*
TCALIF_02597	*SmydA-4*	SmydA	*Tigriopus californicus*
TCALIF_03700	*SmydA-4*	SmydA	*Tigriopus californicus*
TCALIF_03986	*SmydA-4*	SmydA	*Tigriopus californicus*
TCALIF_05703	*SmydA-4*	SmydA	*Tigriopus californicus*
TCALIF_06000	*SmydA-4*	SmydA	*Tigriopus californicus*
TCALIF_06628	*SmydA-4*	SmydA	*Tigriopus californicus*
TCALIF_07034	*SmydA-4*	SmydA	*Tigriopus californicus*
TCALIF_07920	*SmydA-4*	SmydA	*Tigriopus californicus*
TCALIF_10848	*SmydA-9*	SmydA	*Tigriopus californicus*
TCALIF_10306	*Su(var)3-9*	Suv	*Tigriopus californicus*
TCALIF_03934	*suv*	Suv	*Tigriopus californicus*
TCALIF_02488	*trr*	Trx	*Tigriopus californicus*
TCALIF_01684	*trx*	Trx	*Tigriopus californicus*
TCALIF_13010	*upSET(MLL5)*	-	*Tigriopus californicus*
TIMCR00014387	*ash1*	Ash	*Timema cristinae*
TIMCR00022157	*Blimp-1*	PRDM	*Timema cristinae*
TIMCR00001066	*egg*	Suv	*Timema cristinae*

TIMCR00017665	*Ez*	Ez	*Timema cristinae*
TIMCR00006076	*G9a*	Suv	*Timema cristinae*
TIMCR00001220	*Hmt4-20*	-	*Timema cristinae*
TIMCR00027233	*Mes-4*	Ash	*Timema cristinae*
TIMCR00003489	*Set1*	Trx	*Timema cristinae*
TIMCR00000548	*Set2*	Ash	*Timema cristinae*
TIMCR00039149	*Set8*	-	*Timema cristinae*
TIMCR00019708	*setd*	SETD	*Timema cristinae*
TIMCR00010103	*Smyd4-2*	SMYD	*Timema cristinae*
TIMCR00035004	*Smyd4-3*	SMYD	*Timema cristinae*
TIMCR00030298	*Smyd5*	SMYD	*Timema cristinae*
TIMCR00013643	*SmydA-1*	SmydA	*Timema cristinae*
TIMCR00013644	*SmydA-1*	SmydA	*Timema cristinae*
TIMCR00015986	*SmydA-2*	SmydA	*Timema cristinae*
TIMCR00022379	*SmydA-2*	SmydA	*Timema cristinae*
TIMCR00026766	*Su(var)3-9*	Suv	*Timema cristinae*
TIMCR00012042	*Su(var)3-9*	Suv	*Timema cristinae*
TIMCR00023913	*suv*	Suv	*Timema cristinae*
TIMCR00023447	*trr*	Trx	*Timema cristinae*
TIMCR00035528	*trx*	Trx	*Timema cristinae*
TC013422	-	-	*Tribolium castaneum*
TC005321	-	-	*Tribolium castaneum*
TC000311	*ash1*	Ash	*Tribolium castaneum*
TC014741	*Blimp-1*	PRDM	*Tribolium castaneum*
TC006043	*egg*	Suv	*Tribolium castaneum*
TC003094	*Ez*	Ez	*Tribolium castaneum*
TC011127	*G9a*	Suv	*Tribolium castaneum*
TC008337	*Hmt4-20*	-	*Tribolium castaneum*
TC012700	*Mes-4*	Ash	*Tribolium castaneum*
TC015826	*Set1*	Trx	*Tribolium castaneum*
TC014310	*Set2*	Ash	*Tribolium castaneum*
TC015766	*Set8*	-	*Tribolium castaneum*
TC007289	*setd*	SETD	*Tribolium castaneum*
TC007288	*setd*	SETD	*Tribolium castaneum*
TC010888	*Smyd4-3*	SMYD	*Tribolium castaneum*
TC003856	*Smyd4-3*	SMYD	*Tribolium castaneum*
TC005323	*Smyd4-4*	SMYD	*Tribolium castaneum*
TC004406	*Smyd4-4*	SMYD	*Tribolium castaneum*
TC005322	*Smyd4-4*	SMYD	*Tribolium castaneum*
TC000380	*Smyd5*	SMYD	*Tribolium castaneum*
TC005992	*SmydA-1*	SmydA	*Tribolium castaneum*
TC006485	*SmydA-2*	SmydA	*Tribolium castaneum*
TC006182	*SmydA-3*	SmydA	*Tribolium castaneum*
TC006183	*SmydA-3*	SmydA	*Tribolium castaneum*

TC006184	*SmydA-3*	SmydA	*Tribolium castaneum*
TC006159	*SmydA-4*	SmydA	*Tribolium castaneum*
TC005638	*SmydA-5*	SmydA	*Tribolium castaneum*
TC005637	*SmydA-5*	SmydA	*Tribolium castaneum*
TC005968	*SmydA-6*	SmydA	*Tribolium castaneum*
TC014347	*SmydA-8*	SmydA	*Tribolium castaneum*
TC001103	*Su(var)3-9*	Suv	*Tribolium castaneum*
TC000606	*trr*	Trx	*Tribolium castaneum*
TC004768	*trx*	Trx	*Tribolium castaneum*
TC030632	*upSET(MLL5)*	-	*Tribolium castaneum*
TPRE001038	-	-	*Trichogramma pretiosum*
TPRE002399	-	-	*Trichogramma pretiosum*
TPRE003198	-	-	*Trichogramma pretiosum*
TPRE003985	-	-	*Trichogramma pretiosum*
TPRE005031	-	-	*Trichogramma pretiosum*
TPRE007067	-	-	*Trichogramma pretiosum*
TPRE007373	-	-	*Trichogramma pretiosum*
TPRE009604	-	-	*Trichogramma pretiosum*
TPRE009654	-	-	*Trichogramma pretiosum*
TPRE009657	-	-	*Trichogramma pretiosum*
TPRE009686	-	-	*Trichogramma pretiosum*
TPRE010458	-	-	*Trichogramma pretiosum*
TPRE010584	-	-	*Trichogramma pretiosum*
TPRE012931	-	-	*Trichogramma pretiosum*
TPRE013037	-	-	*Trichogramma pretiosum*
TPRE013039	-	-	*Trichogramma pretiosum*
TPRE013040	-	-	*Trichogramma pretiosum*
TPRE003982	*ash1*	Ash	*Trichogramma pretiosum*
TPRE005359	*Blimp-1*	PRDM	*Trichogramma pretiosum*
TPRE006558	*egg*	Suv	*Trichogramma pretiosum*
TPRE001904	*Ez*	Ez	*Trichogramma pretiosum*
TPRE004428	*G9a*	Suv	*Trichogramma pretiosum*
TPRE003399	*Hmt4-20*	-	*Trichogramma pretiosum*
TPRE012907	*Hmt4-20*	-	*Trichogramma pretiosum*
TPRE013035	*Hmt4-20*	-	*Trichogramma pretiosum*
TPRE000203	*Mes-4*	Ash	*Trichogramma pretiosum*
TPRE011489	*Set2*	Ash	*Trichogramma pretiosum*
TPRE011483	*Set8*	-	*Trichogramma pretiosum*
TPRE011484	*Set8*	-	*Trichogramma pretiosum*
TPRE011983	*setd*	SETD	*Trichogramma pretiosum*
TPRE012280	*Smyd4-2*	SMYD	*Trichogramma pretiosum*
TPRE000898	*Smyd4-3*	SMYD	*Trichogramma pretiosum*
TPRE002943	*Smyd4-3*	SMYD	*Trichogramma pretiosum*
TPRE007294	*Smyd4-3*	SMYD	*Trichogramma pretiosum*

TPRE011373	*Smyd4-3*	SMYD	*Trichogramma pretiosum*
TPRE001932	*Smyd4-4*	SMYD	*Trichogramma pretiosum*
TPRE000669	*Smyd4-4*	SMYD	*Trichogramma pretiosum*
TPRE003984	*Smyd4-4*	SMYD	*Trichogramma pretiosum*
TPRE005034	*Smyd4-4*	SMYD	*Trichogramma pretiosum*
TPRE006227	*Smyd4-4*	SMYD	*Trichogramma pretiosum*
TPRE008172	*Smyd4-4*	SMYD	*Trichogramma pretiosum*
TPRE008286	*Smyd4-4*	SMYD	*Trichogramma pretiosum*
TPRE009534	*Smyd4-4*	SMYD	*Trichogramma pretiosum*
TPRE009537	*Smyd4-4*	SMYD	*Trichogramma pretiosum*
TPRE001074	*Smyd5*	SMYD	*Trichogramma pretiosum*
TPRE002558	*SmydA-1*	SmydA	*Trichogramma pretiosum*
TPRE010256	*SmydA-3*	SmydA	*Trichogramma pretiosum*
TPRE011498	*SmydA-8*	SmydA	*Trichogramma pretiosum*
TPRE012312	*Su(var)3-9*	Suv	*Trichogramma pretiosum*
TPRE001049	*suv*	Suv	*Trichogramma pretiosum*
TPRE001671	*trr*	Trx	*Trichogramma pretiosum*
TPRE012706	*upSET(MLL5)*	-	*Trichogramma pretiosum*
XP_011869561.1	*ash1*	Ash	*Vollenhovia emeryi*
XP_011875591.1	*Blimp-1*	PRDM	*Vollenhovia emeryi*
XP_011866930.1	*egg*	Suv	*Vollenhovia emeryi*
XP_011862500.1	*Ez*	Ez	*Vollenhovia emeryi*
XP_011879623.1	*G9a*	Suv	*Vollenhovia emeryi*
XP_011882170.1	*Mes-4*	Ash	*Vollenhovia emeryi*
XP_011878102.1	*Set1*	Trx	*Vollenhovia emeryi*
XP_011883473.1	*Set2*	Ash	*Vollenhovia emeryi*
XP_011865567.1	*Set8*	-	*Vollenhovia emeryi*
XP_011881916.1	*setd*	SETD	*Vollenhovia emeryi*
XP_011865219.1	*Smyd4-2*	SMYD	*Vollenhovia emeryi*
XP_011883193.1	*Smyd4-3*	SMYD	*Vollenhovia emeryi*
XP_011864998.1	*Smyd4-3*	SMYD	*Vollenhovia emeryi*
XP_011865460.1	*Smyd4-3*	SMYD	*Vollenhovia emeryi*
XP_011876680.1	*Smyd4-4*	SMYD	*Vollenhovia emeryi*
XP_011882395.1	*Smyd5*	SMYD	*Vollenhovia emeryi*
XP_011868637.1	*SmydA-4*	SmydA	*Vollenhovia emeryi*
XP_011872217.1	*Su(var)3-9*	Suv	*Vollenhovia emeryi*
XP_011881406.1	*suv*	Suv	*Vollenhovia emeryi*
XP_011878029.1	*trx*	Trx	*Vollenhovia emeryi*
XP_011881364.1	*upSET(MLL5)*	-	*Vollenhovia emeryi*
XP_011689252.1	*ash1*	Ash	*Wasmannia auropunctata*
XP_011704139.1	*Blimp-1*	PRDM	*Wasmannia auropunctata*
XP_011685467.1	*egg*	Suv	*Wasmannia auropunctata*
XP_011685538.1	*Ez*	Ez	*Wasmannia auropunctata*
XP_011688056.1	*G9a*	Suv	*Wasmannia auropunctata*

XP_011700605.1	*Hmt4-20*	-	*Wasmannia auropunctata*
XP_011707267.1	*Mes-4*	Ash	*Wasmannia auropunctata*
XP_011690355.1	*Set1*	Trx	*Wasmannia auropunctata*
XP_011704258.1	*Set2*	Ash	*Wasmannia auropunctata*
XP_011684970.1	*Set8*	-	*Wasmannia auropunctata*
XP_011684969.1	*Set8*	-	*Wasmannia auropunctata*
XP_011689690.1	*setd*	SETD	*Wasmannia auropunctata*
XP_011685059.1	*Smyd4-2*	SMYD	*Wasmannia auropunctata*
XP_011701164.1	*Smyd4-2*	SMYD	*Wasmannia auropunctata*
XP_011692065.1	*Smyd4-3*	SMYD	*Wasmannia auropunctata*
XP_011707114.1	*Smyd4-3*	SMYD	*Wasmannia auropunctata*
XP_011696067.1	*Smyd4-4*	SMYD	*Wasmannia auropunctata*
XP_011703831.1	*Smyd4-4*	SMYD	*Wasmannia auropunctata*
XP_011686678.1	*Smyd5*	SMYD	*Wasmannia auropunctata*
XP_011703429.1	*SmydA-3*	SmydA	*Wasmannia auropunctata*
XP_011707824.1	*SmydA-5*	SmydA	*Wasmannia auropunctata*
XP_011685806.1	*suv*	Suv	*Wasmannia auropunctata*
XP_011692049.1	*trr*	Trx	*Wasmannia auropunctata*
XP_011703242.1	*trx*	Trx	*Wasmannia auropunctata*
XP_011685852.1	*upSET(MLL5)*	-	*Wasmannia auropunctata*
ZNEV_16755	*ash1*	Ash	*Zootermopsis nevadensis*
ZNEV_08706	*Blimp-1*	PRDM	*Zootermopsis nevadensis*
ZNEV_08064	*Blimp-1*	PRDM	*Zootermopsis nevadensis*
ZNEV_15214	*egg*	Suv	*Zootermopsis nevadensis*
ZNEV_02258	*Ez*	Ez	*Zootermopsis nevadensis*
ZNEV_05631	*G9a*	Suv	*Zootermopsis nevadensis*
ZNEV_05656	*Hmt4-20*	-	*Zootermopsis nevadensis*
ZNEV_03918	*Set1*	Trx	*Zootermopsis nevadensis*
ZNEV_02205	*Set2*	Ash	*Zootermopsis nevadensis*
ZNEV_05607	*Set8*	-	*Zootermopsis nevadensis*
ZNEV_12254	*setd*	SETD	*Zootermopsis nevadensis*
ZNEV_05448	*Smyd4-2*	SMYD	*Zootermopsis nevadensis*
ZNEV_08753	*Smyd4-2*	SMYD	*Zootermopsis nevadensis*
ZNEV_00619	*Smyd4-3*	SMYD	*Zootermopsis nevadensis*
ZNEV_01984	*Smyd4-3*	SMYD	*Zootermopsis nevadensis*
ZNEV_06317	*Smyd4-3*	SMYD	*Zootermopsis nevadensis*
ZNEV_01118	*Smyd5*	SMYD	*Zootermopsis nevadensis*
ZNEV_04915	*SmydA-1*	SmydA	*Zootermopsis nevadensis*
ZNEV_06338	*SmydA-2*	SmydA	*Zootermopsis nevadensis*
ZNEV_00680	*SmydA-3*	SmydA	*Zootermopsis nevadensis*
ZNEV_04914	*SmydA-4*	SmydA	*Zootermopsis nevadensis*
ZNEV_06339	*SmydA-5*	SmydA	*Zootermopsis nevadensis*
ZNEV_00677	*SmydA-6*	SmydA	*Zootermopsis nevadensis*
ZNEV_07333	*SmydA-9*	SmydA	*Zootermopsis nevadensis*

ZNEV_00097	*Su(var)3-9*	Suv	*Zootermopsis nevadensis*
ZNEV_04699	*suv*	Suv	*Zootermopsis nevadensis*
ZNEV_09224	*trr*	Trx	*Zootermopsis nevadensis*
ZNEV_03032	*trx*	Trx	*Zootermopsis nevadensis*
ZNEV_06854	*upSET(MLL5)*	-	*Zootermopsis nevadensis*

Supplementary Table S3. Summary of *SET* genes in the 147 arthropod genomes.

Order	Species	SMYD	SETD	PRDM	Ash	Suv	Trx	Ez	Others	Total
Acariformes	*Sarcoptes scabiei*	7	0	1	3	1	2	1	2	17
Acariformes	*Tetranychus urticae*	7	1	1	3	4	2	2	3	23
Parasitiformes	*Ixodes scapularis*	2	1	0	3	4	3	1	3	17
Parasitiformes	*Metaseiulus occidentalis*	1	1	1	1	5	3	1	3	16
Xiphosura	*Limulus polyphemus*	2	1	4	4	5	4	2	6	28
Scorpiones	*Centruroides exilicauda*	2	1	3	3	6	3	1	2	21
Araneae	*Latrodectus hesperus*	1	1	2	3	4	3	1	3	18
Araneae	*Loxosceles reclusa*	0	0(1)	1	4	1	1	0(1)	2	11
Araneae	*Parasteatoda tepidariorum*	1	1	10	5	5	3	1	3	29
Araneae	*Stegodyphus mimosarum*	3	1	2	3	4	2	1	6	22
Amphipoda	*Hyalella azteca*	1	0	1	3	3	4	2	7	21
Calanoida	*Eurytemora affinis*	19	1	0	3	3	4	2	30	62
Cladocera	*Daphnia magna*	7	1	1	3	4	3	1	11	31
Cladocera	*Daphnia pulex*	6	1	2	3	5	3	2	10	32
Harpacticoida	*Tigriopus californicus*	14	0(1)	1	3	4	3	1	52	79
Collembola	*Orchesella cincta*	8	1	1	4	19	4	5	27	69
Diplura	*Catajapyx aquilonaris*	5	1	1	5	8	1	1	8	30
Phthiraptera	*Pediculus humanus*	6	1	1	3	4	3	1	9	28
Blattodea	*Blattella germanica*	4	2	2	4	3	2	0(1)	7	25
Coleoptera	*Agrilus planipennis*	4	1	2	3	3	3	1	9	26
Coleoptera	*Anoplophora glabripennis*	7	1	2	3	3	3	2	7	28
Coleoptera	*Dendroctonus ponderosae*	5	1	1	3	3	3	1	12	29
Coleoptera	*Leptinotarsa decemlineata*	10	1	1	2	5	3	1	9	32
Coleoptera	*Onthophagus taurus*	4	1	1	3	4	3	1	10	27
Coleoptera	*Oryctes borbonicus*	6	1	1	3	3	1	0(1)	9	25
Coleoptera	*Tribolium castaneum*	6	2	1	3	3	3	1	15	34
Diptera	*Aedes aegypti*	11	0(1)	2	3	2	3	2	11	35
Diptera	*Aedes albopictus*	12	0(1)	2	4	3	4	1	12	39
Diptera	*Anopheles albimanus*	7	1	1	3	3	3	1	11	30
Diptera	*Anopheles arabiensis*	15	1	1	3	3	3	1	10	37
Diptera	*Anopheles atroparvus*	6	1	2	3	2	3	1	8	26
Diptera	*Anopheles christyi*	8	1	1	3	3	3	1	9	29
Diptera	*Anopheles coluzzii*	15	1	1	3	3	3	1	8	35
Diptera	*Anopheles culicifacies*	6	1	1	3	3	3	1	10	28
Diptera	*Anopheles darlingi*	7	0(1)	1	3	3	3	0(1)	11	30

Diptera	*Anopheles dirus*	11	0(1)	1	3	3	3	1	9	32
Diptera	*Anopheles epiroticus*	8	1	1	3	3	3	1	9	29
Diptera	*Anopheles farauti*	5	1	1	3	3	3	1	7	24
Diptera	*Anopheles funestus*	8	0(1)	1	3	3	3	1	9	29
Diptera	*Anopheles gambiae*	19	1	1	3	3	3	1	10	41
Diptera	*Anopheles maculatus*	7	0(1)	2	3	3	3	1	9	29
Diptera	*Anopheles melas*	15	1	1	1	3	3	1	9	34
Diptera	*Anopheles merus*	15	1	1	3	3	2	0(1)	11	36
Diptera	*Anopheles minimus*	9	1	1	3	3	3	1	9	30
Diptera	*Anopheles quadriannulatus*	15	0(1)	1	3	3	3	1	10	37
Diptera	*Anopheles sinensis*	6	1	1	2	2	3	1	4	20
Diptera	*Anopheles stephensi*	8	1	1	3	3	3	1	11	31
Diptera	*Bactrocera cucurbitae*	5	1	1	3	4	3	1	13	31
Diptera	*Bactrocera dorsalis*	4	1	2	4	4	6	2	22	45
Diptera	*Ceratina calcarata*	5	1	0(1)	2	4	3	1	11	28
Diptera	*Ceratitis capitata*	5	1	1	3	3	3	1	14	31
Diptera	*Culex quinquefasciatus*	40	1	1	13	2	9	1	14	81
Diptera	*Drosophila ananassae*	5	0(1)	1	3	4	3	1	12	30
Diptera	*Drosophila biarmipes*	5	1	1	4	4	3	1	9	28
Diptera	*Drosophila bipectinata*	5	1	1	3	4	3	1	10	28
Diptera	*Drosophila elegans*	5	1	1	3	3	3	1	9	26
Diptera	*Drosophila erecta*	5	1	1	3	3	3	1	13	30
Diptera	*Drosophila eugracilis*	4	0(1)	1	3	3	3	1	11	27
Diptera	*Drosophila ficusphila*	5	1	1	3	3	3	1	8	25
Diptera	*Drosophila grimshawi*	5	1	1	3	3	3	1	12	29
Diptera	*Drosophila kikkawai*	5	1	1	3	4	3	1	10	28
Diptera	*Drosophila melanogaster*	5	1	1	3	4	3	1	12	30
Diptera	*Drosophila miranda*	5	1	0(1)	3	3	3	1	11	28
Diptera	*Drosophila mojavensis*	5	1	0	4	3	2	1	14	30
Diptera	*Drosophila persimilis*	5	1	1	4	5	3	1	11	31
Diptera	*Drosophila pseudoobscura*	5	1	1	4	4	3	1	11	30
Diptera	*Drosophila rhopaloa*	5	1	1	3	5	2	1	8	26
Diptera	*Drosophila sechellia*	5	1	1	3	4	2	1	11	28
Diptera	*Drosophila simulans*	5	1	0(1)	3	3	3	1	9	26
Diptera	*Drosophila suzukii*	5	1	1	3	4	4	1	10	29
Diptera	*Drosophila takahashii*	4	1	1	3	4	3	1	7	24
Diptera	*Drosophila virilis*	5	1	1	4	4	4	1	12	32
Diptera	*Drosophila willistoni*	4	1	1	3	5	4	1	11	30
Diptera	*Drosophila yakuba*	5	1	1	3	4	3	1	11	29
Diptera	*Glossina austeni*	4	1	1	3	2	3	1	14	29
Diptera	*Glossina brevipalpis*	4	1	1	4	5	3	1	14	33
Diptera	*Glossina fuscipes*	5	1	1	3	5	4	0(1)	15	35
Diptera	*Glossina morsitans*	4	1	1	3	4	3	1	12	29
Diptera	*Glossina pallidipes*	5	1	1	3	4	3	0(1)	14	32

Diptera	*Glossina palpalis*	5	1	1	3	4	3	1	13	31
Diptera	*Lucilia cuprina*	5	1	1	3	3	3	1	12	29
Diptera	*Lutzomyia longipalpis*	6	1	1	3	3	2	1	10	27
Diptera	*Mayetiola destructor*	13	0(1)	1	9	6	4	1	25	60
Diptera	*Megaselia scalaris*	2	0(1)	1	3	2	1	0(1)	5	16
Diptera	*Musca domestica*	5	1	0(1)	3	3	3	1	20	37
Diptera	*Phlebotomus papatasi*	5	1	0(1)	4	3	3	1	6	24
Diptera	*Stomoxys calcitrans*	5	1	1	3	2	3	1	16	32
Diptera	*Belgica antarctica*	27	2	1	3	5	4	0(1)	12	55
Ephemeroptera	*Ephemera danica*	18	1	1	2(1)	1(1)	2	1	12	40
Hemiptera	*Acyrthosiphon pisum*	14	0(1)	0	2	10	4	1	31	63
Hemiptera	*Cimex lectularius*	4	1	2	3	5	3	1	5	24
Hemiptera	*Diaphorina citri*	3	0(1)	1	4	4	3	2	11	29
Hemiptera	*Gerris buenoi*	6	1	1	3	3	3	1	8	26
Hemiptera	*Halyomorpha halys*	5	1	1	2	5	3	1	8	26
Hemiptera	*Homalodisca vitripennis*	5	2	2	2	5	4	1	8	29
Hemiptera	*Nilaparvata lugens*	4	1	6	2	4	4	1	7	29
Hemiptera	*Oncopeltus fasciatus*	6	1	0(1)	2	5	4	1	7	27
Hemiptera	*Pachypsylla venusta*	1	1	2	2	3	1	1	9	20
Hemiptera	*Rhodnius prolixus*	6	1	0(1)	2	2	2	1	6	21
Hymenoptera	*Acromyrmex echinatior*	7	2	1	3	3	3	1	7	27
Hymenoptera	*Apis dorsata*	6	0(1)	1	3	3	1	1	7	23
Hymenoptera	*Apis florea*	7	1	1	3	3	2	0(1)	8	26
Hymenoptera	*Apis mellifera*	7	1	1	3	4	3	1	9	29
Hymenoptera	*Athalia rosae*	7	1	2	2	3	2	1	8	26
Hymenoptera	*Atta cephalotes*	8	1	1	3	4	3	1	7	28
Hymenoptera	*Bombus impatiens*	7	1	1	3	4	3	1	10	30
Hymenoptera	*Bombus terrestris*	8	1	1	3	4	3	1	8	29
Hymenoptera	*Camponotus floridanus*	8	2	1	2	3	2	1	8	27
Hymenoptera	*Cardiocondyla obscurior*	7	2	1	3	4	3	1	10	31
Hymenoptera	*Cephus cinctus*	6	1	1	0(2)	3	2	0(1)	6	22
Hymenoptera	*Cerapachys biroi*	5	1	1	0(2)	3	3	1	6	22
Hymenoptera	*Ceratosolen solmsi*	8	1	1	3	3	2	1	9	28
Hymenoptera	*Copidosoma floridanum*	17	1	1	3	4	2	1	16	45
Hymenoptera	*Dufourea novaeangliae*	7	2	1	3	4	3	1	7	28
Hymenoptera	*Eufriesea mexicana*	6	2	1	3	4	3	1	8	28
Hymenoptera	*Fopius arisanus*	9	1	1	3	4	1	1	9	29
Hymenoptera	*Habropoda laboriosa*	8	2	1	3	4	3	1	8	30
Hymenoptera	*Harpegnathos saltator*	8	2	0	1	2	1	1	8	23
Hymenoptera	*Linepithema humile*	7	2	1	3	4	3	1	8	29
Hymenoptera	*Megachile rotundata*	7	2	1	3	3	3	1	8	28
Hymenoptera	*Melipona quadrifasciata*	7	2	1	3	4	3	1	8	29
Hymenoptera	*Microplitis demolitor*	18	1	1	3	4	3	2	8	40
Hymenoptera	*Monomorium pharaonis*	6	1	1	2	3	2	1	5	21

Hymenoptera	*Nasonia vitripennis*	17	1	1	3	4	2	1	23	52
Hymenoptera	*Orussus abietinus*	11	2	1	2	3	3	1	7	30
Hymenoptera	*Pogonomyrmex barbatus*	5	2	0(1)	2	4	3	1	8	26
Hymenoptera	*Polistes dominula*	6	1	1	1	4	2	1	6	22
Hymenoptera	*Solenopsis invicta*	2	1	0(1)	3	3	3	1	7	21
Hymenoptera	*Trichogramma pretiosum*	15	1	1	3	4	1	1	26	52
Hymenoptera	*Vollenhovia emeryi*	6	1	1	3	4	2	1	3	21
Hymenoptera	*Wasmannia auropunctata*	7	1	1	3	3	3	1	6	25
Hymenoptera	*Lasioglossum albipes*	9	1	1	3	3	3	1	8	29
Isoptera	*Zootermopsis nevadensis*	6	1	2	2	4	3	1	10	29
Lepidoptera	*Bombyx mori*	4	2	1	3	4	3	1	8	26
Lepidoptera	*Danaus plexippus*	5	1	1	3	5	3	1	10	29
Lepidoptera	*Heliconius melpomene*	5	0(1)	1	2	4	3	1	6	23
Lepidoptera	*Papilio polytes*	6	1	1	3	2	2	0(1)	11	27
Lepidoptera	*Papilio xuthus*	6	1	1	3	4	2	1	9	27
Lepidoptera	*Plutella xylostella*	5	4	1	4	5	6	0	13	38
Lepidoptera	*Lerema accius*	4	1	2	3	3	3	1	10	27
Lepidoptera	*Melitaea cinxia*	5	1	1	3	1	0(3)	0(1)	8	23
Lepidoptera	*Manduca sexta*	6	2	7	7	5	5	2	29	63
Odonata	*Ladona fulva*	3	2	2	2(1)	3(1)	3	1	9	27
Orthoptera	*Locusta migratoria*	9	1	1	3	4	3	1	7	29
Phasmatoptera	*Timema cristinae*	3	1	1	3	5	3	1	6	23
Thysanoptera	*Frankliniella occidentalis*	6	2	8	3	5	3	1	21	49
Trichoptera	*Limnephilus lunatus*	3	1	0	0(2)	3	2	1	6	18

Note: The numbers in parenthesis indicates the number of the genes which are not present in the official gene sets. The species selected in the Figure 2A are highlighted in red. Others, arthropod-specific and unclassified *SET* genes

Supplementary Table S4. Primers used in the study.

**Recombinant protein expression**	
L06-SP6-Kozak-F	TATTTAGGTGACACTATAGAACAGACCACCATGGCCAGCAGGGGCTTCAAA
L06-his-R	TTAATGATGATGATGATGATGGTCATCATTTAATTCTGTGGCA
**RNA interference**	
RNAi-GFP-F	CACAAGTTCAGCGTGTCCG
RNAi-GFP-R	GTTCACCTTGATGCCGTTC
RNAi-SmydA-2-F	CGCCCTGGGCCCCAAGATCT
RNAi-SmydA-2-R	CCAGCACGGCGATGCGGAAG
**Probe synthesis**	
FISH-SmydA-2-F	GACCGCCACTCCGACAGCAAGAT
FISH-SmydA-2-R	ACATGCCTGGTTCCAGAACATCA
**qPCR**	
RP49-F (reference)	CCATAGTTGCCTGACTCCC

RP49-R	GATGGAGGCGGATAAAGTTG
qPCR-SmydA-2-F	TACAAGACGCTGCTGACGCT
qPCR-SmydA-2-R	CGCGTTCGTGTCCAAGATG

Note: F, forward primers; R, reverse primers.

Supplementary Figures

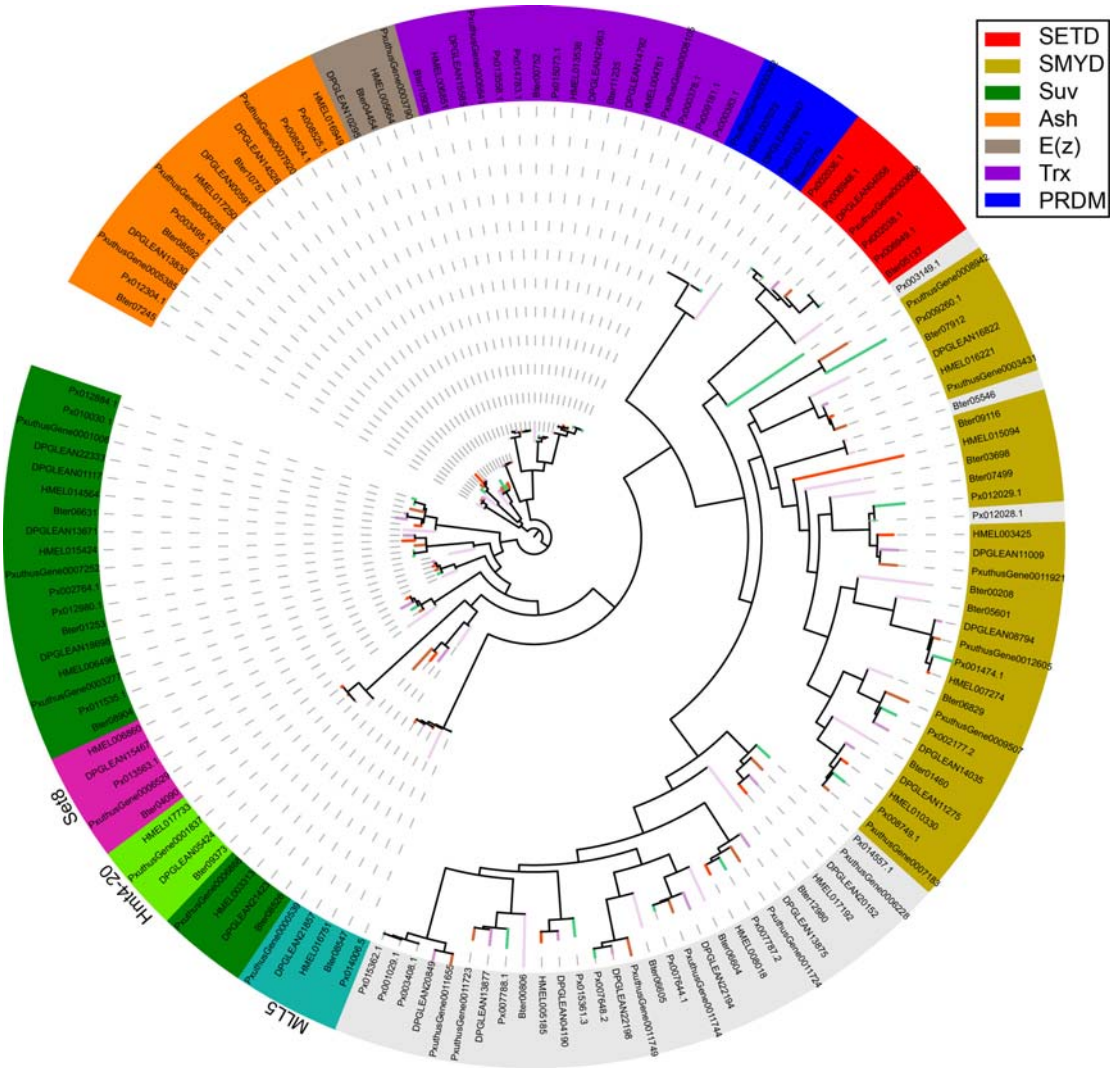

Supplementary Figure S1. Phylogenetic analysis of the *SET* genes in Lepidoptera using Maximum-likelihood inferences with PhyML. The *SET* gene families labeled with different colors are shown in the exterior circle of the phylogenetic tree. The insect species involved are represented with different colors of the external branch.

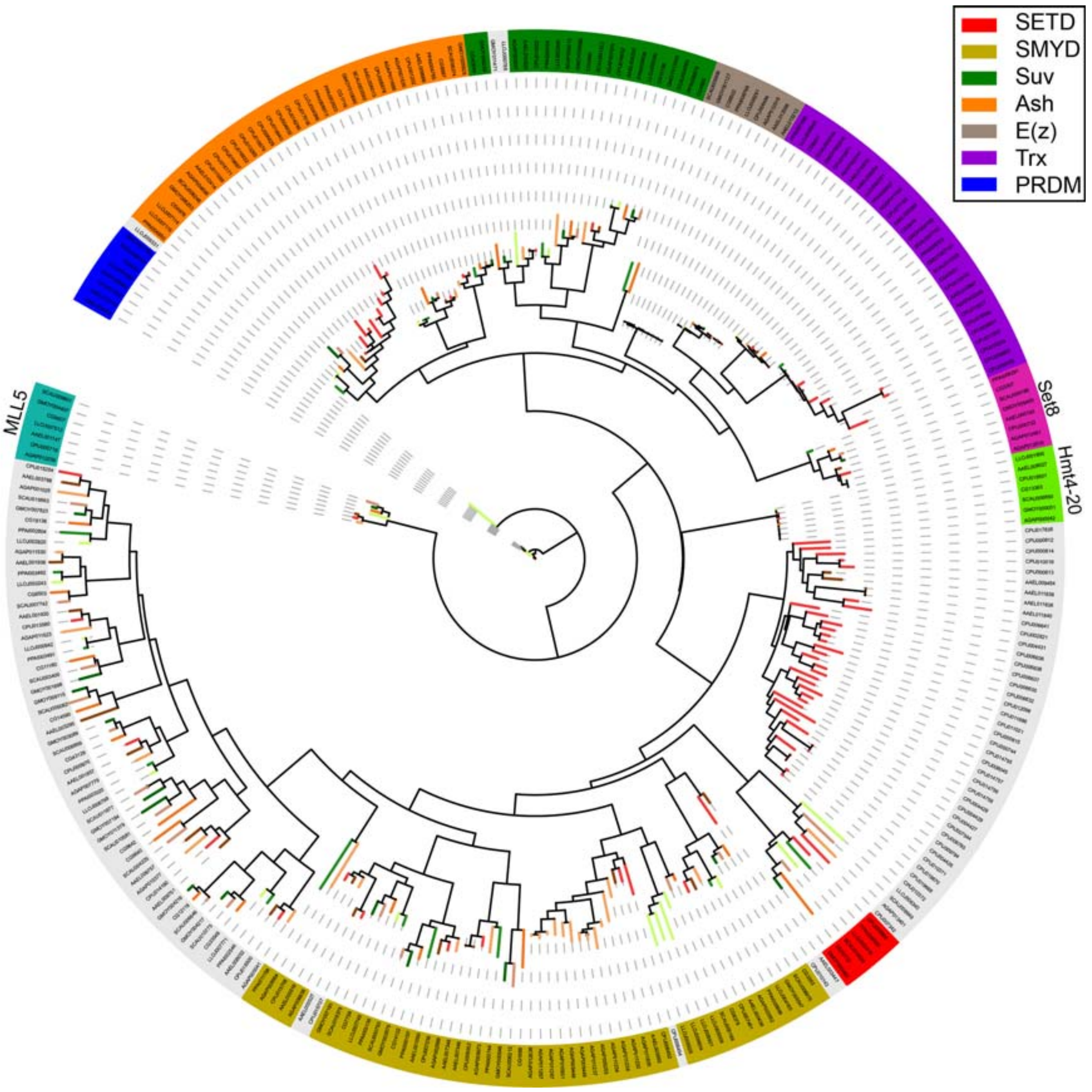

Supplementary Figure S2. Phylogenetic analysis of the *SET* genes in Diptera using Maximum-likelihood inferences with PhyML. The *SET* gene families labeled with different colors are shown in the exterior circle of the phylogenetic tree. The insect species involved are represented with different colors of the external branch. The representative species are selected to improve clarity.

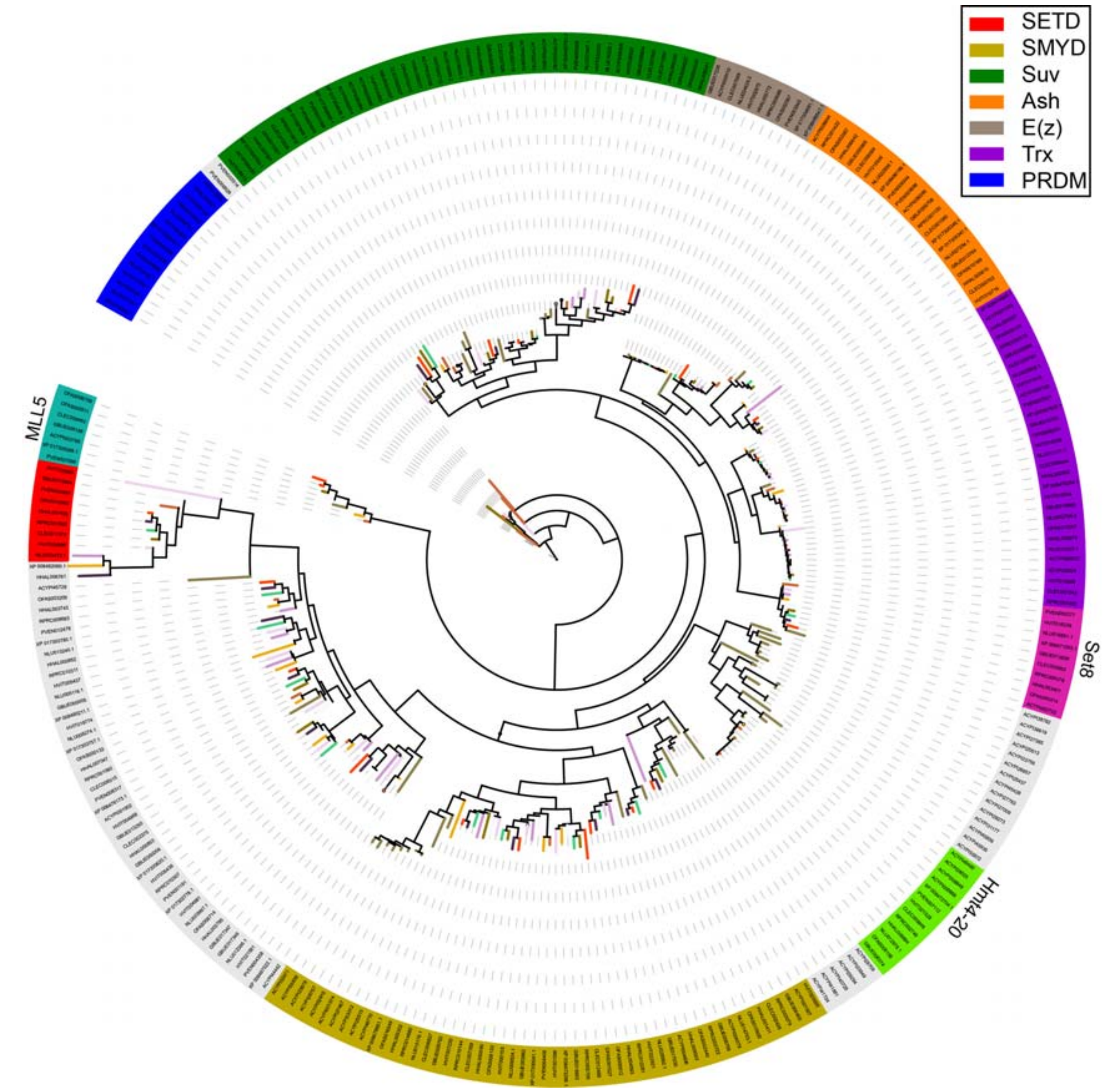

Supplementary Figure S3. Phylogenetic analysis of the *SET* genes in Hemiptera using Maximum-likelihood inferences with PhyML. The *SET* gene families labeled with different colors are shown in the exterior circle of the phylogenetic tree. The insect species involved are represented with different colors of the external branch.

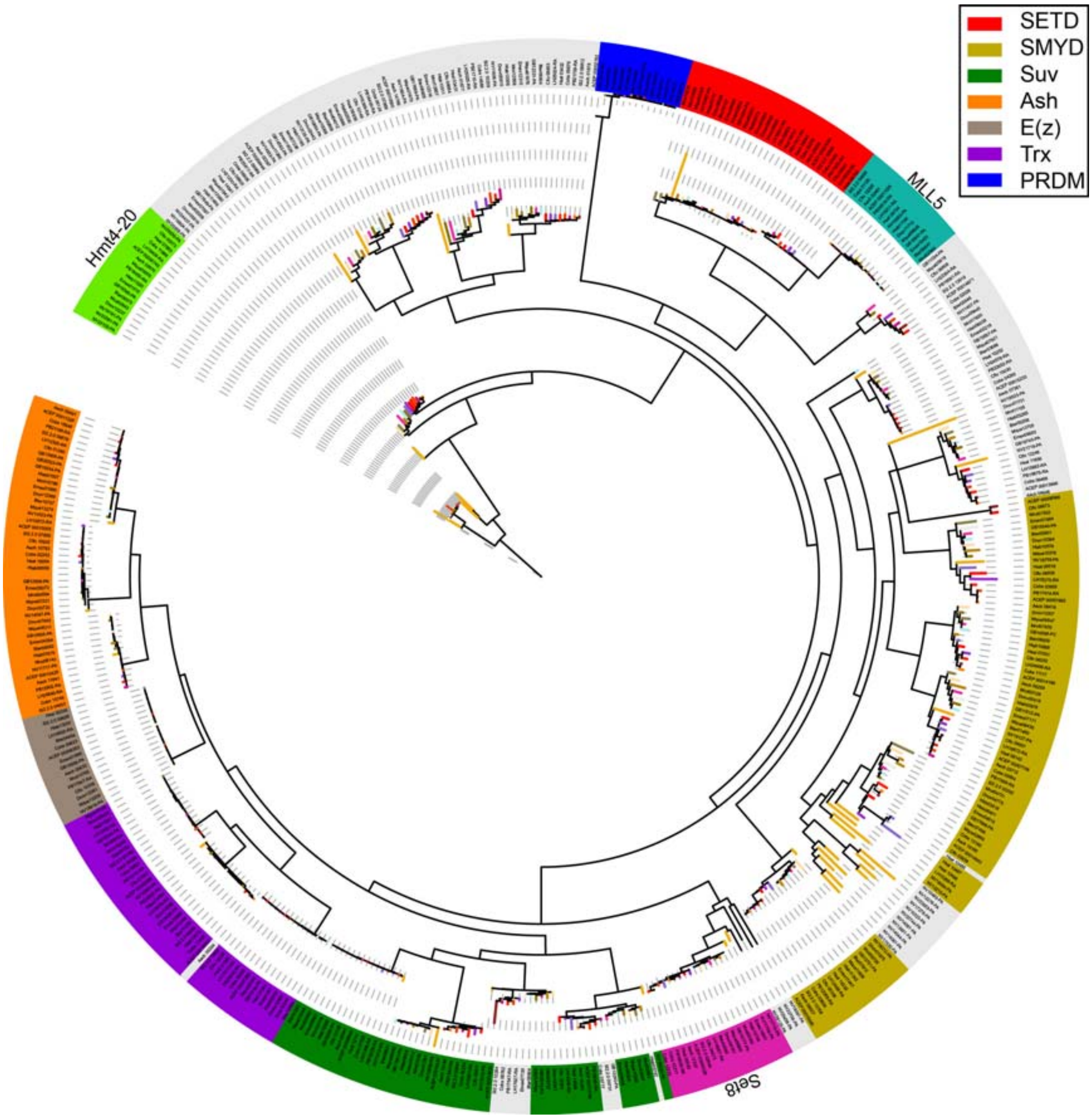

Supplementary Figure S4. Phylogenetic analysis of the *SET* genes in Hymenoptera using Maximum-likelihood inferences with PhyML. The *SET* gene families labeled with different colors are shown in the exterior circle of the phylogenetic tree. The insect species involved are represented with different colors of the external branch. The representative species are selected to improve clarity.

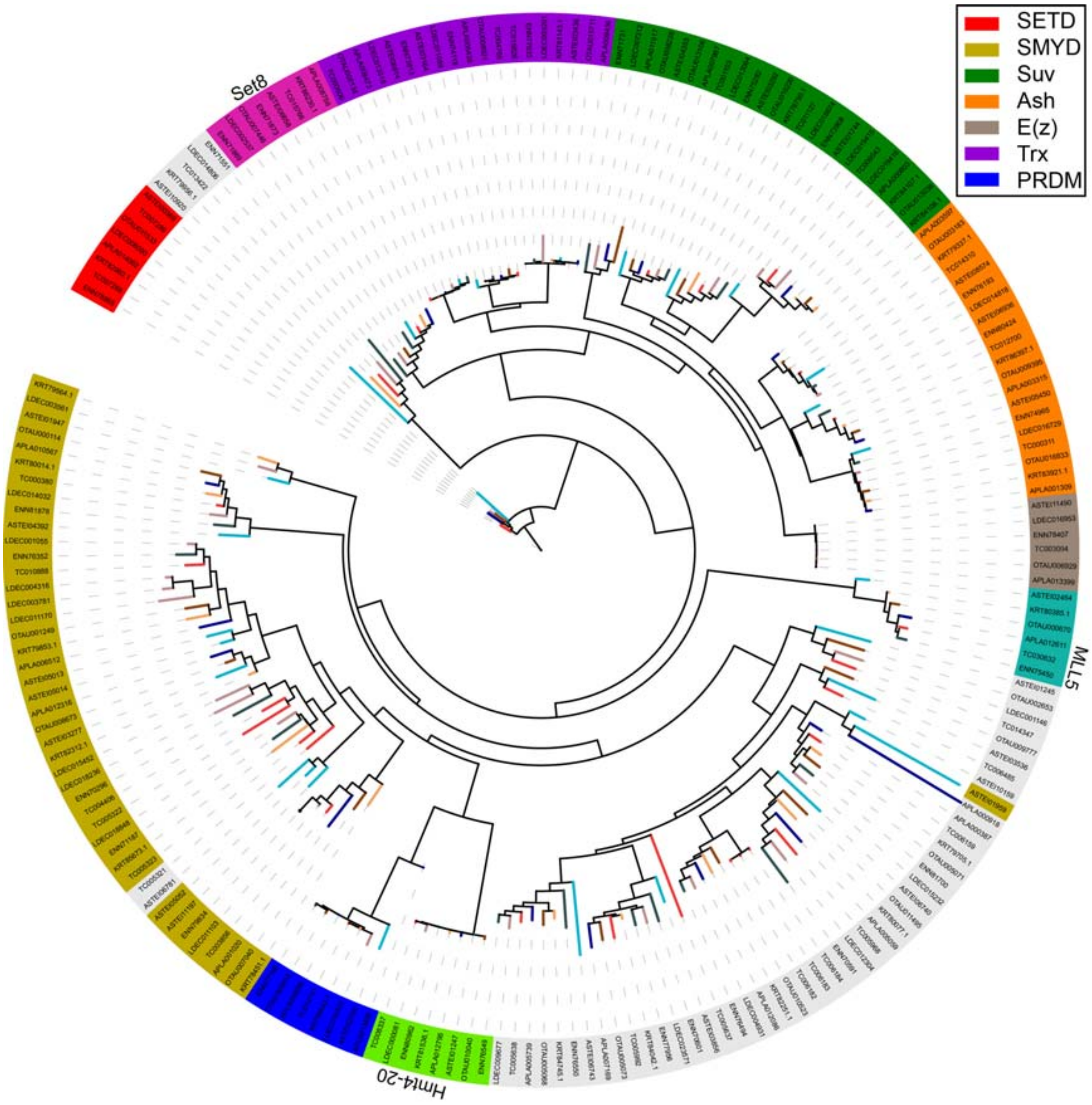

Supplementary Figure S5. Phylogenetic analysis of the *SET* genes in Coleopteran using Maximum-likelihood inferences with PhyML. The *SET* gene families labeled with different colors are shown in the exterior circle of the phylogenetic tree. The insect species involved are represented with different colors of the external branch.

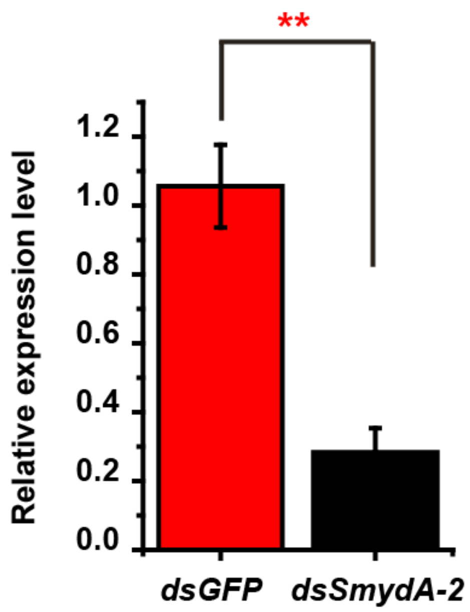

Supplementary Figure S6. Effects of RNA interference of the mRNA expression levels of *SmydA-2* in locust brains. The locusts are injected with double-stranded RNAs into the second ventral segment of the abdomen. Due to the systemic RNA interference in locusts, the brain, which is spatially distant from the abdomen, is used in qPCR assays to guarantee effective expression knockdown. qPCR data are shown as the mean ± SEM (n = 6). **P < 0.01.

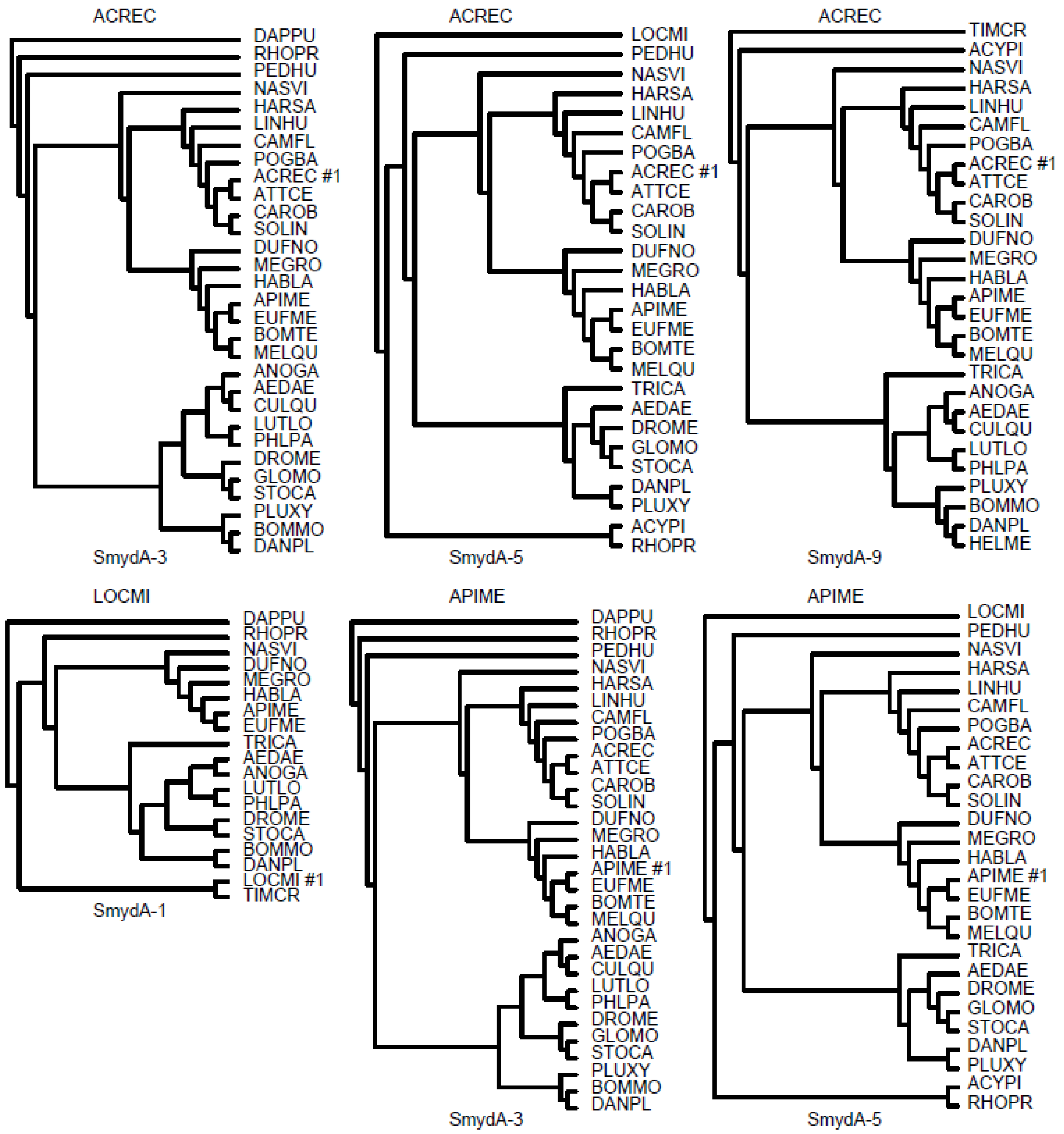

Supplementary Figure S7. Tree topology and branch labeling for tests of selection on *SET* genes. APIME, *Apis mellifera*; ACREC, *Acromyrmex echinatior*; LOCMI, *Locusta migratoria*. Supplementary Table S1 presents the abbreviation of insect species.
