# Supplementary material for: Comparative genomic analysis of SET domain family reveals the origin, expansion, and putative function of the arthropod-specific SmydA genes as histone modifiers in insects
Source: Gigascience. 2017 Apr 22;6(6):1–16. doi: 10.1093/gigascience/gix031 (PMC5459927; doi:10.1093/gigascience/gix031)
Supplement: GIGA-D-16-00127_Revision_1.pdf [file gix031_GIGA-D-16-00127_Revision_1.pdf]

Dear Editor,

Thank you for your organizing the review for our manuscript (by Jiang et al. MS# GIGA-D-16-00127). Also, we appreciate the two reviewer's constructive suggestions and comments which largely improved the quality and presentation of this manuscript. Here, I submitted the revised manuscript and answered all questions raised by the reviewers in a point-by-point manner. We believe that the revised manuscript has been substantially improved in terms of data retrieval and presentation. Please let me know if you have any further question.

Best regards,

Le Kang, Ph.D. and Professor

Institute of Zoology, Chinese Academy of Sciences

Beijing 100101, China

Tel: 86-10-6480-7219

Fax:86-10-6480-7099

E-mail: lkang@ioz.ac.cn

Response to the Reviewers

Explanation: All editorial correspondence from *Gigascience*, including the reviewers' comments, is verbatim in black. Our responses are inserted directly into this text in blue. All changes have been implemented in the final version of the manuscript.

GIGA-D-16-00127

Comparative genomic analysis of SET-domain family reveals the origin, expansion, and putative function of the arthropod-specific SmydA genes as histone modifier in insects

Feng Jiang; Qing Liu; Yanli Wang; Jie Zhang; Huimin Wang; Tianqi Song; Meiling Yang; Xianhui Wang; Le Kang

GigaScience

Dear Prof Kang,

Your manuscript "Comparative genomic analysis of SET-domain family reveals the origin, expansion, and putative function of the arthropod-specific SmydA genes as histone modifier in insects" (GIGA-D-16-00127) has been assessed by our reviewers. We are unable to consider it for publication in its current form, but we would be willing to send a revised manuscript for re-review, if you are able to fully address the comments below.

The reviewers have raised a number of important points (see below), and we can not make a decision on the manuscript unless those comments are fully addressed in a revised manuscript.

In particular, you need to be more precise with respect to your methods and justify better your analysis decisions (e.g. with respect to inclusion/exclusion criteria etc - see the referees' reports).

Response:

We have addressed the reviewer' comments specifically and mention below how the text changed as a consequence.

In addition to the two reports below, we have discussed the paper with another expert adviser, who unfortunately was not able to complete a full report. But from this discussion with a third expert we draw another set of comments that we as ask you to address:

We feel you need to concentrate more on one major question in the introduction. You mention subfunctionalisation, epigenetic protein modification, and evolution of SET domain containing genes. This seems a bit convoluted, and we feel it needs better emphasis. We are also not sure how conclusive the RNAi experiments are in respect to the function of these genes - this will need more details and better justification.

Response:

The primary results show that the evolution novelty of *SET* domain containing genes is linked to the insect phenotypic plasticity by putative histone modification. Therefore, we deleted the following sentences regarding to subfunctionalisation to emphasis the importance of the other two aspects:

"In taxonomically related species, the expansion of conserved gene families through gene duplication is widespread in metazoan genomes [4]. Gene duplication may increase species fitness by subfunctionalization or neofunctionalization [5, 6]. Subfunctionalization results in the symmetric division of the functional capability of the original gene among the duplicated genes [7]. Neofunctionalization allows the original copy to maintain its function and permits the new copy to diverge under relaxed selective constraints or positive selection for a novel function."

Essential genes are often considered as conserved and functionally important, whereas

pseudogenes have been considered to be more dispensable and to have minor influences on survival and phenotype. Knockdown or knockout of essential gene (for example, DNA cytosine-5-methyltransferases in honeybees and histone methyltransferase G9a in mice) expression often result in lethal phenotype. Therefore, the point that no pseudogenization for *SmydA-2* in locusts could be supported by the RNAi experiments in this study. Accordingly, we added the following sentence to achieve a better justification:

“Essential genes are often considered as conserved and functionally important [29], whereas pseudogenes have been considered to be more dispensable and to have minor influences on survival and phenotype.”

References:

Krylov DM, Wolf YI, Rogozin IB, Koonin EV: Gene loss, protein sequence divergence, gene dispensability, expression level, and interactivity are correlated in eukaryotic evolution. *Genome Res* 2003, 13(10):2229-2235.

Miklos GL, Rubin GM: The role of the genome project in determining gene function: insights from model organisms. *Cell* 1996, 86(4):521-529.

Kucharski R, Maleszka J, Foret S, Maleszka R: Nutritional control of reproductive status in honeybees via DNA methylation. *Science* 2008, 319(5871):1827-1830.

Tachibana M, Sugimoto K, Nozaki M, Ueda J, Ohta T, Ohki M, Fukuda M, Takeda N, Niida H, Kato H et al: G9a histone methyltransferase plays a dominant role in euchromatic histone H3 lysine 9 methylation and is essential for early embryogenesis. *Genes & development* 2002, 16(14):1779-1791.

In the main manuscript, please also clarify how gene gain was inferred (Fig. 2) and mention the method used for optimising character changes. The expert who has advised us on the paper also relayed the following comment, that we also ask you to

address:

Response:

We consider only the binary state, presence or absence, of a given *SET* homologous group in any given node. The member number of *SET* homologous group in each species was not considered. Ancestral state reconstruction was implemented in the Mesquite program under maximum likelihood optimization using Markov k-state 1 parameter model in which forward and backward transition rates are equal. After ancestral reconstruction, we measured gain (emergence) and loss events of *SET* homologous group along each branch in the phylogenetic tree. The gain event of *SET* homologous group was defined as the *SET* homologous group was absent at the ancestral nodes of a given node and either of the outgroups.

To improve clarity, we revised the following sentences in the Method section:

"We constructed a character matrix that represents present/absent states for each *SET* homologous group to reconstruct the ancestral states of interior clades. We did not consider member number variation and considered only the binary state, presence or absence, of a given *SET* homologous group in any given node."

"Ancestral state reconstruction was implemented in the Mesquite program (http://mesquiteproject.org/) under maximum likelihood optimization using Markov k-state 1 parameter model. After ancestral reconstruction, we measured emergence and loss events of *SET* homologous group along each branch in the phylogenetic tree. The emergence event of *SET* homologous group was defined as the *SET* homologous group was absent at the ancestral nodes of a given node and either of the outgroups"

"How can we be certain of the absence of these genes in some taxa, given the great divergence and possible lack of similarity to the HMM used to detect the genes in the first place?"

Response:

SET domain, which is necessary for many histone lysine methyltransferases,

possesses a catalytic activity that transfers a methyl group to the amino group of lysine residues of nuclear histones from S-adenosyl-L-methionine. Therefore, the detection of *SET* domain provides an efficient way to identify histone lysine methyltransferase. HMM-based searching performs much better than pairwise methods (for example BLAST), and it is amongst the most successful approaches for detecting REMOTE HOMOLOGY (divergent homologs) between proteins. We detected the *SET* domains of the seven major conserved groups in the arthropod species, which are diverged from each other hundreds of million years ago, suggesting that HMM-based searching is competent for divergent homolog identification. Whether a *SET* domain lacking of sequence similarity (rapid evolving or pseudogenaztion) to known *SET* domain homologs retain histone lysine methylation activities is still an open question, but this is an issue beyond the scope of this study. Hope the matter is satisfactory.

References:

Madera M, Gough J: A comparison of profile hidden Markov model procedures for remote homology detection. *Nucleic Acids Res* 2002, 30(19):4321-4328.

Please also make an effort to present your work shorter, but with greater precision, also with respect to figure legends. (for example Fig. 1:, to cite our adviser: "What are the long lines after each terminal? How are the terminals were chosen, given there are thousands of gene sequences, and how they were categorized (based on the tree or some assignment done by an external analysis?"

Response:

To improve preciseness and clearness, we provided much more detailed descriptions of both the figure legends in the three Figures and the Methods section in the main text. The length of the grey long line after each terminal is directly proportional to the length of the corresponding *SET* gene. As noted in the figure legend of Figure 1, the terminals were chosen based on the following criteria; One representative is elected for each order. They were categorized based on a phylogenetic tree using Bayesian

inference analysis of protein sequences of *SET* genes.

In response, we added the following sentence in the figure legend of Figure 1:

"The length of the grey long line after each terminal is directly proportional to the length of the corresponding *SET* gene."

Please also mention the sources of all original data you use, including accession numbers and, if possible, accession date. Further material that can help reproducibility, such as custom scripts, intermediate results, supplementary data, software outputs etc, can be uploaded to our server GigaDB. You are also welcome to use protocols.io (https://www.protocols.io/) as a convenient way to share methods and protocols.

Response:

Dr, Scott Edmunds, the executive editor of *GigaScience*, asked us to upload all the required files to server GigaDB several days later after the initial submission. Here are the lists for the files we have uploaded to GigaDB. Please see the full email correspondence at the bottom of this document for further information.

1) All the sequences files for SET genes in this study: 1.allSETgeneSequence.tar.gz

2) The alignment based and non-alignment based phylogeny trees and the MAFFT alignment file in the Figure 1: 2.PhylogeneticTreeSETgenes.tar.gz

3) The BUSCO-based "species tree" which is used for phylogeny inference of insect orders in the Figure 2: 3.BUSCOSpeciesTree.tar.gz

4) The MAFFT alignment file for BUSCO-based single-copy genes: 4.BUSCOalignment.tar.gz

5) HMMER output file for SET domain identification: 5.HMMERout.tar.gz

6) PSILC program output file: 6. PSILCoutput.tar.gz

7) InterProScan output file: 7. InterProScanoutput.tar.gz

8) The phylogeny trees include in the supplementary files (in Newick format): 8. PhylogenyTreesinSupplyFiles.tar.gz

9) The improved gene models using transcriptome data: 9.RevisedGeneModels.tar.gz

The reports, together with any other comments, are below. Please also take a moment to check our website at http://giga.edmgr.com/ for any additional comments that were saved as attachments.

If you are able to fully address these points, we would encourage you to submit a revised manuscript to GigaScience. Once you have made the necessary corrections, please submit online at:

http://giga.edmgr.com/

If you have forgotten your username or password please use the "Send Login Details" link to get your login information. For security reasons, your password will be reset.

Please include a point-by-point within the 'Response to Reviewers' box in the submission system. Please ensure you describe additional experiments that were carried out and include a detailed rebuttal of any criticisms or requested revisions that you disagreed with. Please also ensure that your revised manuscript conforms to the journal style, which can be found in the Instructions for Authors on the journal homepage.

The due date for submitting the revised version of your article is 19 Apr 2017.

I look forward to receiving your revised manuscript soon.

Best wishes,

Hans Zauner

GigaScience

www.gigasciencejournal.com

Reviewer reports:

Reviewer #2: This manuscript describes an in-depth analysis of the SET genes in arthropod species, with a particular interest in the Smyd class which includes both widely conserved and arthropod-specific members. It is of special interest that the authors make an effort to combine high throughput bioinformatic analyses with an experimental approach to prove that arthropod-specific Smyd proteins retain histone

modification activity and are differentially expressed in phenotypicaly different individuals of the same species. The work is well suited for the GigaScience journal, but in the opinion of this reviewer some questions should be addressed.

Response:

We really appreciate the positive responses from the reviewer.

1. The introduction gives a description of the molecular function of SET domain-containing proteins as histone modification enzymes, but does not discuss already published data on Smyd gene evolution. Given the special emphasis on Smyd genes, it would be convenient to mention previous publications giving a classification of Smyd genes in vertebrates and invertebrates. The article published by Calpena et al (PlosOne 2015) is of particular relevance, since the authors introduce the main Smyd classes: Smyd3, which includes the vertebrate Smyd1 and 2; Smyd 4, which is expanded in arthropods; Smyd 5; and the arthropod-specific SmydA. Based on this evidence, they introduce some of the nomenclature used in this manuscript (Smyd4-1 to 4, SmydA-1 to 9). The manuscript under review makes a more detailed analysis of Set genes in several arthropod species, but giving due credit to previous work does not diminish the merit of theirs. On the contrary, it provides a framework in which to give a richer discussion of their own results.

Response:

Thanks for this suggestions and we accepted this criticism thoughtfully. In response, we added the following sentences in the revision:

"A recent study has provided a framework for understanding the evolution history of SMYD gene family in representative animal phyla [24]. The phylogenetic results show that the metazoan SMYD genes can be classified in three main classes, *Smyd3*, *Smyd5* and *Smyd4*. Two sub-classes of SMYD genes, namely *Smyd4-4* and *SmydA*, are absent in vertebrates; the former on is insect-specific and the later one is arthropod-specific. Within Chelicerata, we detected *Smyd4-4* in Acariform mites (non-insect arthropods), suggesting our evidence did not support the point that

Smyd4-4 is specific of insects. Since Chelicerata represents an out-group branch for this study, further studies covering more basal branches of arthropod phylogeny are required to ascertain the origin of *Smyd4-4*."

2. In pages 6 and 7 it is described how the sequences were selected and the overall distribution of set genes in arthropods. Bearing in mind that the process of sequence inclusion/exclusion is very complicated in such a diverse family, the authors must discuss how this may have affected their analysis. This discussion is relevant on the light of the apparent contradictions indicated below (points 8-10).

Response:

There are no contradictions and inconsistencies regarding to this issue in the original manuscript. Please see the explanations below for the points 8-10. This issue is not raised by sequence inclusion/exclusion.

We appreciated this good comment that has led us to revisit our thinking of an important but easily neglected point of this study and emphasize the importance of sequence inclusion/exclusion in this study. *SET* domain possesses a catalytic activity that transfers a methyl group to the amino group of lysine residues of nuclear histones from S-adenosyl-L-methionine. Therefore, *SET* domain is necessary for many histone lysine methyltransferases. During the course of evolution, a few cases of conserved genes have lost some core domains, which are crucial for their gene functions. The loss of crucial domain usually abolishes its gene function. For example, the *SET* domain in *Smyd3* could be identified both in vertebrates and in invertebrates. It has been experimentally validated that the human *Smyd3* showed histone H3-K4 methyltransferase activity, in consistent with the presence of *SET* domain in *Smyd3* in human. We failed to detect the *SET* domain in *Smyd3* in all the *Drosophila* species even under a less stringent criterion of e-value cut-off. However, the *SET* domain in *Smyd3* could be identified in a large number of insects and in *Anopheles gambiae*, which is in the same order of *Drosophila*. This indicates that *SET* domain has specifically lost in *Drosophila* species, implying that *Smyd3* in *Drosophila* species

may deprived of histone methylation activity. Indeed, no experimentally evidence for histone methylation capacity for *Smyd3* has been reported in *Drosophila* so far. Because our study focuses on histone lysine methyltransferases which are by means of *SET* domain, the genes lacking *SET* domain (even though the *SET* domain can be detected in their homologs in closely related species) were excluded for further analysis in this study.

To make this point clear to the readers, we added the following sentences in the main text:

"Despite that the *SET* domain can be detected in their homologs in closely related species, the genes lacking *SET* domain were considered as deprived of lysine methylation capacity and were excluded for further analysis."

References:

Hamamoto R, Furukawa Y, Morita M, Iimura Y, Silva FP, Li M, Yagyu R, Nakamura Y: SMYD3 encodes a histone methyltransferase involved in the proliferation of cancer cells. Nat Cell Biol 2004, 6(8):731-740.

3. In pages 7 and 8 and in Figure 1 the phylogeny of Set genes is described. Two methods are used, alignment-based Bayesian and alignment-free, and the authors state that both gave similar topologies. In Figure 1, only the first tree is shown, it would be convenient to show the other phylogeny in a supplementary figure.

Response:

Thanks for this comment. We uploaded both the alignment based and non-alignment based phylogeny trees (in Newick format) in the Figure 1 to the publicly accessible database GigaDB. In addition, we also uploaded the MAFFT alignment file to GigaDB.

4. In Figure 1 there are two color codes, one for domains outside the circle and one for the Set gene classes, but the second one is not mentioned in the figure legend. The black branches must be the arthropod-specific genes. Is that so?

Response:

Yes, the black branches indicate the *SET* genes which cannot be classified into the seven major conserved groups, suggesting their arthropod origin. To improve clarity, we added the following sentences into the figure legend:

"The branch colors of the phylogenetic tress indicate the established SET gene classification which divides *SET* genes into seven major conserved groups, namely: Suv, Ash, Trx, E(z), PRDM, SMYD, and SETD. The *SET* genes labeled in black branches cannot be classified into the seven major conserved groups, suggesting their arthropod origin."

5. At this point it should be mentioned in the main text that this branch corresponds to the already defined SmydA class.

Response:

Thanks for this comment. The *SET* genes labeled in black branches include both the defined *SmydA* genes and the unclassified *SET* genes which show patchy distributed patterns across arthropod species. In order to achieve preciseness, we added the following sentence in the main text:

"Indeed, a large number of these *SET* genes are homologuous to the already defined arthropod-specific *SmydA* genes described in the previous study [28]."

References:

Calpena E, Palau F, Espinos C, Galindo MI: Evolutionary History of the Smyd Gene Family in Metazoans: A Framework to Identify the Orthologs of Human Smyd Genes in Drosophila and Other Animal Species. *PLoS One* 2015, 10(7):e0134106.

6. From line 177 onwards the term "set homologous group" is used, but it is not

defined. My guess is that the 19 homologous groups mentioned in line 185 are the ones in Figure 1B, taking Smyd4-1 to Smyd4-4 as one group. The authors should define clearly what they call a set homologous group and identify them with a reference to a figure or a table. In addition I would suggest reorganising Figure 2 by swapping panels A and B.

Response:

Yes, your guess is right. The grouping of the *SET* genes was inferred using the OrthoMCL software, which is based on a scalable method for constructing orthologous groups across multiple eukaryotic taxa. As suggested, we revised the following sentence and re-organized Figure 2 by swapping panels A and B.

"A character matrix that represents the present/absent states for each *SET* homologous group (a OrthoMCL-based homolog set including both putative orthologs and paralogs) was constructed to infer the ancestral states of interior nodes along with the species tree using the Mesquite program."

References:

Li L, Stoeckert CJ, Jr., Roos DS: OrthoMCL: identification of ortholog groups for eukaryotic genomes. *Genome Res* 2003, 13(9):2178-2189.

7. More information should be given regarding Figure 2. The bars to the right in panel A are not explained in the figure legend. Panel B is based on selected species within each order, but it is not mentioned which are these species. I suggest highlighting them on Supplementary Table S3.

Response:

We agreed with this comment, and accordingly we revised our manuscript as follows.

We added the following explanation for the bars on the right in the panel A in the figure legend of Figure 2B (original Figure 2A):

"The bars indicate the number ranges of *SET* homologous groups in each order."

We added the following sentence in the table note of the Supplementary Table S3: "The species selected in the Figure 2A are highlighted in red."

8. As a result of the distribution reflected in Figure 3B it is mentioned in line 233 that SmydA genes are absent in all Chelicerata. But in the Supplementary table S3 it is indicated that chelicerates have arthropod-specific genes in range of 2-6 copies. Please explain this discrepancy and ensure that the information reflected for these and other species is accurate.

Response:

Many thanks for this comment. The point that *SmydA* genes are absent in all Chelicerata could not be reflected in Figure 3B. Figure 2B (see Partial Figure 2B below) clearly indicates the absence of *SmydA* genes in Chelicerata. To improve clarity, adding "as shown in Figure 2B" to this sentence makes it clear to the readers that the point regarding to *SmydA* genes in Chelicerata is reflected in Figure 2B.

Partial Figure 2B (Figure 2A in the revision):

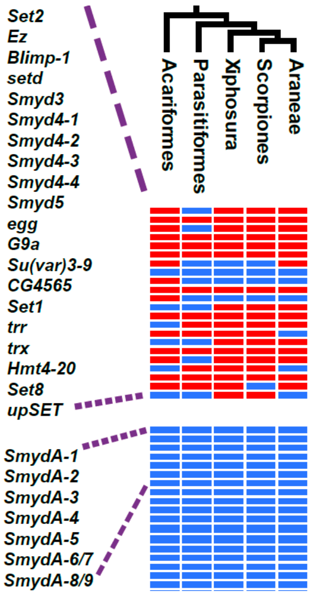

It is our fault that we did not detect that the column name had not been updated. In the the draft version of the manuscript, there were two columns, namely arthropod-specific and unclassified *SET* genes, respectively. In the original submission, we combined these two columns into a single column. Let's take

Tetranychus urticae (Chelicerata) as example. A total of 23 *SET* genes is present in *Tetranychus urticae* (see the partial Supplementary Table S3 below), and twenty of them are belong to the seven major conserved groups. Because 02g11280 (*Hmt4-20*), 20g02320 (*Set8*) and 20g02380 (*Set8*) could not be classified into the seven major conserved groups (see the partial Supplementary Table S2 below), these three genes were considered as unclassified *SET* genes. Therefore, *SmydA* is absent in *Tetranychus urticae*. We apologize for the carelessness. In response, "AS" was substituted to "Others", and the footnote in Supplementary Table S3 was corrected as follows:

"Others, arthropod-specific and unclassified *SET* genes."

Partial Supplementary Table S3:

Supplementary Table S3. Summary of *SET* genes in the 147 arthropod genomes.

Order	Species	SMYD	SETD	PRDM	Ash	Suv	Trx	Ez	AS	Total
Acariformes	*Sarcoptes scabiei*	7	0	1	3	1	2	1	2	17
Acariformes	*Tetranychus urticae*	7	1	1	3	4	2	2	3	23

Partial Supplementary Table S2:

03g02610	*ash1*	Ash	*Tetranychus urticae*
15g00080	*Blimp-1*	PRDM	*Tetranychus urticae*
03g02220	*egg*	Suv	*Tetranychus urticae*
07g01000	*egg*	Suv	*Tetranychus urticae*
28g01660	*egg*	Suv	*Tetranychus urticae*
05g08610	*Ez*	Ez	*Tetranychus urticae*
15g02370	*Ez*	Ez	*Tetranychus urticae*
02g11280	*Hmt4-20*	-	*Tetranychus urticae*
02g03670	*Mes-4*	Ash	*Tetranychus urticae*
07g03190	*Set1*	Trx	*Tetranychus urticae*
15g00050	*Set2*	Ash	*Tetranychus urticae*
20g02320	*Set8*	-	*Tetranychus urticae*
20g02380	*Set8*	-	*Tetranychus urticae*
07g04000	*setd*	SETD	*Tetranychus urticae*
12g03090	*Smyd3*	SMYD	*Tetranychus urticae*
02g04130	*Smyd4-2*	SMYD	*Tetranychus urticae*
04g03960	*Smyd4-2*	SMYD	*Tetranychus urticae*
06g06280	*Smyd4-2*	SMYD	*Tetranychus urticae*
06g06270	*Smyd4-3*	SMYD	*Tetranychus urticae*
08g00440	*Smyd4-3*	SMYD	*Tetranychus urticae*
02g06270	*Smyd4-4*	SMYD	*Tetranychus urticae*
04g08120	*Su(var)3-9*	Suv	*Tetranychus urticae*
17g01640	*trr*	Trx	*Tetranychus urticae*

9. Calpena et al describe a sub-class within Smyd4, Smyd4-4, which is specific of insects. The authors should discuss if their evidence supports this.

Response:

We detected *Smyd4-4* in the non-insect arthropods, suggesting our evidence did not support the point that *Smyd4-4* is specific of insects.

10. The tree in Figure 3B shows a clear segregation between the SmydA and Smyd4 groups, but it does not include Smyd3 or Smyd5. Smyd3 is also highlighted as absent from diptera in Figure 2B, despite the fact that there is a Smyd3 gene defined in Drosophila, which again reinforces the need to discuss the criteria for sequence inclusion (point 2) and the selection of the representative species (point 7). Are these the same representative species selected in Figure 2B?

Response:

Many thanks for this comment and the conscientious reading of our manuscript by the reviewer. We are VERY embarrassed. We thought we had been very circumspect in manuscript preparation, but evidently we were not sufficiently circumspect. In fact, we included *Smyd3* or *Smyd5* in Figure 3B. There is a branch named *Smyd4-5* (*Smyd4-5* has never existed throughout the manuscript and the supplementary file at all) in the Figure 3B in the original submission. Actually, *Smyd4-5* is a typo for *Smyd5*. Since the *SET* domains in *Smyd3* are only detected in a limited number of species studied (See the seventh line of the matrix in Figure 2A), the *Smyd3* genes from a few species constitute the minor branch closing to *Smyd5*. Again, it is entirely our fault and we apologize. We explained the criteria for sequence inclusion in the point 2 above and included the names of the representative species in the figure legend of Figure 2B.

The Figure 3B in the original submission:

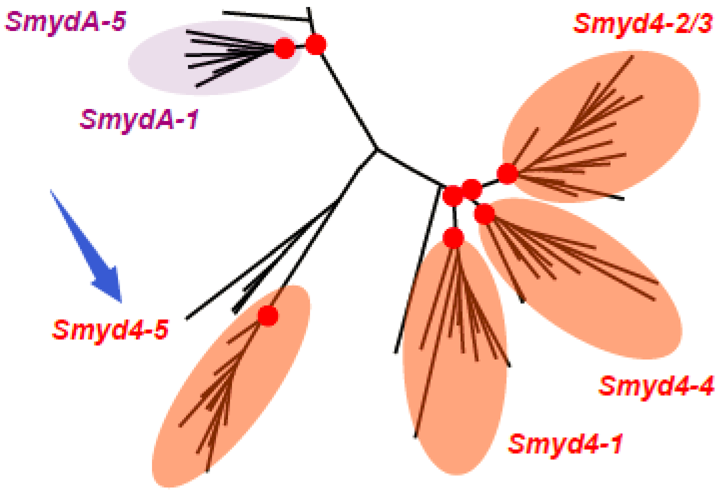

The corrected Figure 3B in the revision:

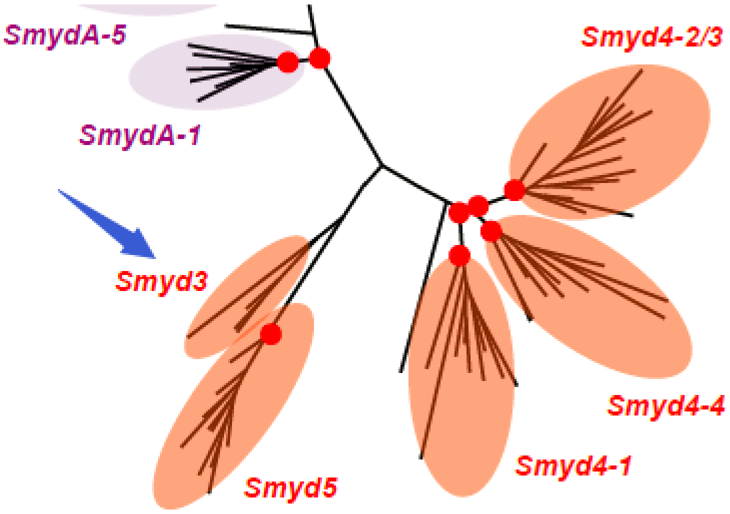

11. Unless the reader is an expert in entomology, it is difficult to identify the four species only by the body shapes. Species names should be mentioned in the legend.

Response:

We added the following sentence in the figure legend of Figure 3C:

"Shown from top to bottom are *Drosophila melanogaster*, *Anopheles gambiae*, *Tribolium castaneum* and *Apis mellifera*."

Reviewer 1:

Jiang et al. performed a thorough phylogenetic analysis of arthropod SET-domain containing genes and identified an arthropod-specific SET gene family (SmydA).

They showed that the latter gene family is under strong purifying selection, and complemented their bioinformatic analyses with experimental data showing that a.o. members of the SmydA gene family are essential for insect survival. This is a nice and interesting study but, in my opinion, the manuscript has one major flaw, namely a very concise, in some cases unclear, Material &Methods. Once the issues mentioned below have been addressed, I consider this manuscript acceptable for publication in GigaScience.

Response:

We thank for the reviewer's positive responses for our study.

Line 348: what was the extent of fold change for SET genes? Were the SET genes among the most highly overexpressed genes in the comparison between alternative phenotypes?

Response:

We used |log2FC| > 0.585 (log2 fold change, corresponds to 1.5 fold change) in the DE analysis. As shown in the following figure, the extent of log2 fold change for the *SET* genes ranges from -10.4 to 9.2.

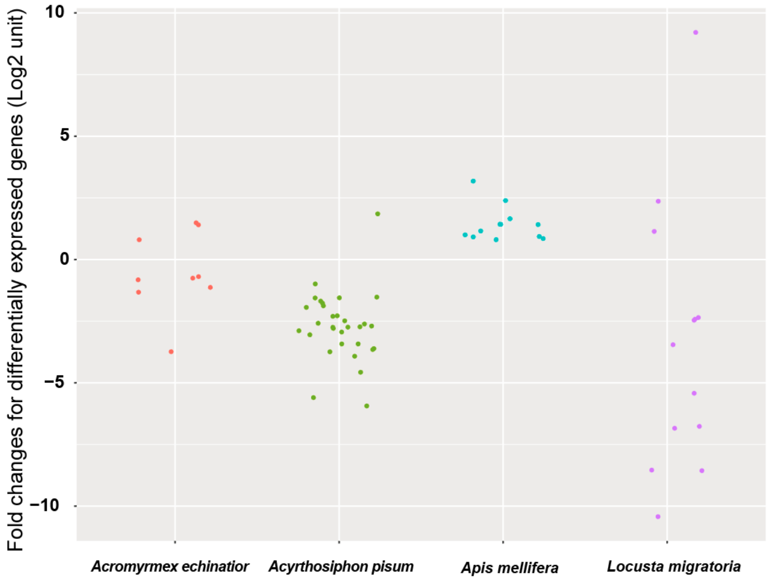

No, the *SET* genes were not among the most highly overexpressed genes in the

comparison between alternative phenotypes. We sorted the DE genes in an ascending order according to their expression levels. To show the expression levels of the *SET* genes in a global view, we computed the percentile ranks of each *SET* genes and plotted the distribution of the expression percentile ranks using a dot plot. The distribution of expression percentile ranks indicated the *SET* genes in DE lists showed a broad range of expression levels.

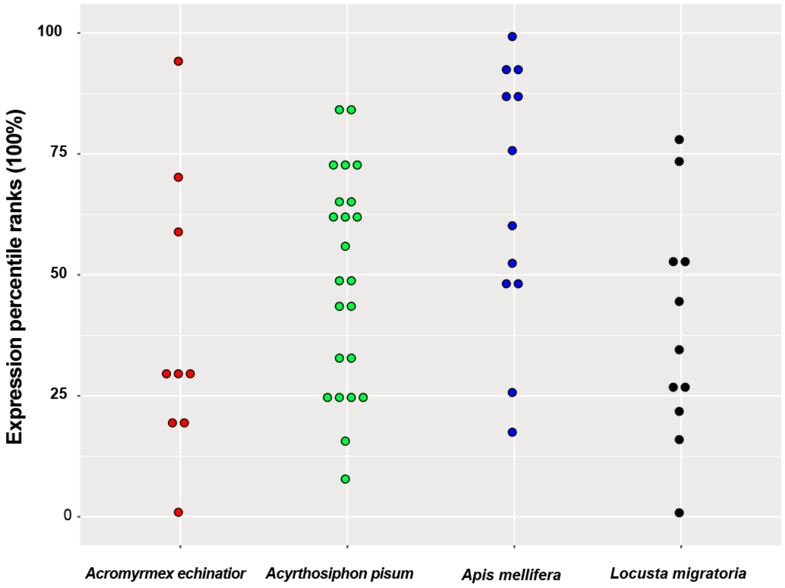

Line 499: which E-value cutoff was used in the HMMER search for SET genes?

Response:

To improve clarity, we revised the following sentence:

"The hidden Markov model-based HMMER program was used to identify the *SET* domain containing proteins using PF00856 in the Pfam database with a conditional E-value cutoff of 1e-5 [43, 44]."

Table S2: Next to accession numbers, authors should provide a fasta-file containing the 4,498 SET gene sequences that were used for this study. This way an interested reader does not need to browse different genome portals to collect data. In addition, the study is not dependent on a website that might not be available/working in the future (see also Minor corrections).

Response:

We agreed with this comment. In response to the editor's requests, we uploaded all the *SET* gene sequences to GigaDB, a database serving as a official repository to host data associated with articles in *GigaScience*. Please see the full email correspondence at the bottom of this document for further information.

Figure 1: author should include a phylogenetic tree (as a supplementary file) with the accession numbers of the sequences (or alternatively upload the alignment to a data repository like e.g. Dryad)

Response:

Thanks for this comment. In response to the editor's requests, we uploaded both the alignment based and non-alignment based phylogeny trees (the accession numbers are included in the Newick trees) to the publicly accessible database GigaDB. In addition, we also uploaded the MAFFT alignment file to GigaDB.

Line 504-505: GO analysis description is very concise/unclear. Authors should provide more details. e.g. be more specific about InterPro databases and models that were used.

Response:

Thanks for this comment. We divided all the *SET* genes into a large number of small subsets of sequences. These subsets of sequences were scanned against InterPro's signatures using the InterProScan version 5.13-52.0 program simultaneously on a Linux cluster server. The member database binaries and models include TIGRFAM, ProDom, Panther, SMART, PrositePatterns, SuperFamily, PRINTS, Gene3d, PIRSF, PfamA and PrositeProfiles. The Gene ontology terms for each *SET* gene were assigned according to the InterPro's signatures. The InterProScan output files have been uploaded to GigaDB. Accordingly, we added the InterProScan (GeneWise and PSILC as well) program version and the following sentence into the Method section: "The member database binaries and models include TIGRFAM, ProDom, Panther, SMART, PrositePatterns, SuperFamily, PRINTS, Gene3d, PIRSF, PfamA and PrositeProfiles."

Line 512: what settings were used with MAFFT?

Response:

To improve clarity, we revised the following sentence:

"Multiple alignments were generated using the MAFFT alignment software with default parameters."

line 514: which version of ProtTest software was used?

Response:

We revised the following sentence to make a clear statement for the ProtTest software version used in this study.

"According to the Akaike information criterion, the model of molecular evolution with the best fit to the data was determined by using the ProtTest 3.4.2 software [49]."

Line 519: authors should provide more details about the feature frequency profile method (parameters etc…)

Response:

To improve clarity, we revised the following sentence:

"The alignment-free and distance-based methods for phylogenetic tree building were implemented by means of the feature frequency profile method with the FFP version 3.19 suite (http://sourceforge.net/projects/ffp-phylogeny/), utilizing the FFPaa program for amino acid sequences with a word length of L = 5. The FFPboot program was used for bootstrap analysis of the tree generated for 100 replicates."

Line 528: the authors are very concise regarding the single copy orthologous gene family phylogenetic analysis, authors should provide more details (method?, provide alignment as a supplementary file).

Response:

Thanks for this comment. Using BUSCO analysis, genes sets were classified into the four categories, namely completed, duplicated, fragmented and missing, respectively.

Only the completed BUSCO genes (single-copy ortholog) were used for further species tree construction. A neighbor-joining phylogenetic tree was constructed from amino acid sequences of single-copy orthologs using Phylip version 3.69 package. The bootstrap values, calculated from 100 replicates using the seqboot, protdist, neighbor and consense programs of Phylip version 3.69 package. As requested by the executive editor, we uploaded both the BUSCO-based "species tree" and the MAFFT alignment file for BUSCO-based single-copy genes to GigaDB. In response, we revised the following sentences in the Method section:

"Single-copy orthologous gene families were inferred from the benchmarking universal single-copy ortholog BUSCO gene sets from each species [51]. The resulting 527 single-copy orthologous (completed genes in BUSCO) gene families were used to construct the neighbor-joining species tree, which is consistent with the phylogenomic tree recently inferred from transcriptome data [18]. The neighbor-joining species tree was constructed from amino acid sequences of single-copy orthologs using Phylip version 3.69 package. The bootstrap values, calculated from 100 replicates using the seqboot, protdist, neighbor and consense programs of Phylip package."

Line 538: authors should provide genome assembly versions of the different insect species

Response:

We added the following sentence in the Method section:

"(genome assembly version: v2.4 for *L. migratoria*, v1.0 for *A. pisum*, Amel_2.0 for *A. mellifera* and Aech_v2.0 for *A. echinatior*, respectively)"

Line 539-542: author should provide parameters for the Tophat2 mapping. What version of Tophat 2 was used? What version of HTSeq and EdgeR was used?

Response:

The versions are 2.0.14 for Tophat2, 0.6.1 for HTSeq and 3.8.0 for edgeR,

respectively. We added the versions for these three programs in the Method section.

line 542: what was the FC cutoff used for the DE analysis? In addition authors should provide a list of DE genes for all comparisons as a Supplementary Table

Response:

We used |log2FC| > 0.585 (log2 fold change, corresponds to 1.5 fold change) in the DE analysis. We uploaded the lists of DE genes for the four insects to GigaDB.

line 548: …and then backtranslated…; with what kind of software/script was the backtranslation done?

Response:

A basic command line is required for this task. We put the sequences (the aligned protein sequences in the first column and the corresponding nucleotide sequences in the second column) into a file, and type the following command for backtranslation:

```
cat filename | perl -ne ' my ($aa,$nt) = split /\t/; my $j = 0;for (my $i = 0;$i < length($aa) - 1;$i++){ my $amiac = substr($aa,$i,1); my $bases = "";if ($amiac !~ /\-/) { $bases = substr($nt,$j,3); $j+=3;} else { $bases = "---";} print "$bases";}'
```

Line 546-547/: how was the SmydA-2 gene picked up, which primers/PCR protocol were used?

Response:

We added the PCR protocol in the Method section and provided the primers in the Supplementary Table S4.

“The PCR parameters were a preincubation 94 °C for 5 min, followd by 30 cycles of 94 °C for 10 sec, 58 °C for 30 sec,72 °C for 30 sec, and a final extension at 72 °C for 10min.”

Line 559-560: very concise description of recombinant protein expression? Which

primers were used to pick-up gene/ligation protocol (restriction enzymes?) into vector etc. Authors should provide more details.

Response:

We provided the more detailed description for the recombinant protein expression and provided the primers in the Supplementary Table S4. Accordingly, we revised the following sentences in the revision:

"The recombinant proteins for *SmydA-2* and the negative controls of translation system were produced using the TNT protein expression system (Promega) following the manufacturer's protocol. In brief, 3 μg PCR-generated DNA templates (Supplementary Table S4) were added to 30 μl TNT master mix, and the translation reactions were incubated at 25 °C for 2 hr. The recombinant proteins were verified by Western blotting using His-tag antibodies."

Line 569: is this a well-established laboratory locust strain? Has this strain been previously described (origin, name?). Authors should provide more details for this strain if possible.

Response:

Yes, the locusts used in this study are from a well-established laboratory locust strain in our lab. This locust strain has been sequenced and described in our previous studies. In response, we revised the following sentence:

"Locusts (the migratory locust, *Locusta migratoria*) were reared in large, well-ventilated cages (40 cm × 40 cm × 40 cm) at a density of 500–1000 insects per container."

References:

Kang L, Chen X, Zhou Y, Liu B, Zheng W, Li R, Wang J, Yu J: The analysis of large-scale gene expression correlated to the phase changes of the migratory locust. *Proc Natl Acad Sci U S A* 2004, 101(51):17611-17615.

Ma Z, Guo W, Guo X, Wang X, Kang L: Modulation of behavioral phase changes of the migratory locust by the catecholamine metabolic pathway. *Proc Natl Acad Sci U S A* 2011, 108(10):3882-3887.

Yang M, Wei Y, Jiang F, Wang Y, Guo X, He J, Kang L: MicroRNA-133 inhibits behavioral aggregation by controlling dopamine synthesis in locusts. *PLoS Genet* 2014, 10(2):e1004206.

Wang X, Fang X, Yang P, Jiang X, Jiang F, Zhao D, Li B, Cui F, Wei J, Ma C et al: The locust genome provides insight into swarm formation and long-distance flight. *Nat Commu*n 2014, 5:2957.

He J, Chen Q, Wei Y, Jiang F, Yang M, Hao S, Guo X, Chen D, Kang L: MicroRNA-276 promotes egg-hatching synchrony by up-regulating brm in locusts. *Proc Natl Acad Sci U S A* 2016, 113(3):584-589.

Line 572: authors should provide primers that were used for dsRNA synthesis

Response:

We provided the primers in the Supplementary Table S4.

Line 578/579: authors should provide more details regarding measuring of SmydA-2 mRNA expression levels (qPCR: primers, amplification protocol, reference genes?...)

Response:

We added the following sentences in the Method section and provided the primers in the Supplementary Table S4.

"The parameters were a pre-incubation 95°C for 10 min, followed by 45 cycles of 95 °C for 10 sec, 58 °C for 20 sec, and a single acquisiton when 72 °C for 20 sec. The ribosomal protein 49 gene was used as reference control, and the quantification was based on the requirement of PCR cycle number (Ct) to cross or exceed the fluorescence intensity level; the $2^{-\Delta\Delta Ct}$ method was used to analyze mRNA expression levels."

Line 580: literature reference for the Kaplan-Meier method?

Response:

We added the literature reference for the Kaplan-Meier method in the Method section.

Line 806: which representative species were used, authors should include a phylogenetic tree (as a supplementary file) with the accession numbers of the sequences (or alternatively upload the alignment to a data repository like e.g. Dryad)

Response:

The 11 representative species were selected from 11 arthropod orders. We have uploaded the alignment file to GigaDB and included the species names in the figure legend of Figure 3B as follows:

"The representative species include *Apis mellifera*, *Daphnia pule*, *Drosophila melanogaster*, *Ixodes scapularis*, *Locusta migratoria*, *Pediculus humanus*, *Plutella xylostella*, *Rhodnius prolixus*, *Tetranychus urticae*, *Timema cristinae* and *Tribolium castaneum*."

Typos/Minor Corrections

line 143: link to SET gene database is not working (see also comment above)

Response:

We checked the running status of the *SET* gene database and we will take a periodic checking to make sure the database is working properly. Alternatively, all the data deposited in our database can be retrieved from the database GigaDB which is maintained by *GigaScience*.

line 151: why would genome-size be correlated with SET-gene number? Is there any precedent in literature. If not, authors should remove this sentence

Response:

We agreed with this comment, and we removed this sentence in the revision.

Line 155: please specify in the manuscript which "representative species" were used for phylogenetic analysis?

Response:

We included the species names in the figure legend of Figure 1 as follows:

"The representative species include *Apis mellifera*, *Daphnia pule*, *Drosophila melanogaster*, *Ixodes scapularis*, *Locusta migratoria*, *Pediculus humanus*, *Plutella xylostella*, *Rhodnius prolixus*, *Tetranychus urticae*, *Timema cristinae* and *Tribolium castaneum*."

Line 266: was present in all…

Response:

Thanks for your elaborative comments. The text has been revised as suggested.

Line 284: replace "were" with "are"

Response:

The text has been revised as suggested.

Line 309: methylation activities

Response:

The text has been revised as suggested.

Line 354: "sensitivities" of DEG number? Authors should rephrase

Response:

We revised the following sentence in the revision:

"the number changes of the DEGs in *SET* genes in the four insects were even more prominent…"

Line 375: remove "as"

Response:

The text has been revised as suggested.

Figure 2B: reformat/resize font so the names of the arthropod specific SET genes can also be shown

Response:

As suggested, we resized the font of Figure 2B to show the names of *SmydA* in the arthropod specific *SET* genes. We did not label the remaining ones in the arthropod specific *SET* genes, because they are randomly emerged and are not well-characterized into a specific gene category.

Previous Responses to Dr. Scott Edmunds, executive editor of GigaScience, in November 10, 2016

From: Feng Jiang jiangf@biols.ac.cn

To: database@gigasciencejournal.com, em@editorialmanager.com

CC: KANG <lkang@ioz.ac.cn>

Dear Dr. Scott Edmunds and Dr. Chris Hunter,

We have uploaded all the required files to GigaDB and provide a point-by-point response below to your previous comments. Please substitute the revised manuscripts (including main-text, supplementary file and Table 1) which are included in the attached files to the corresponding files in our previous submission.

Best regards,

Feng Jiang

On behalf of Prof. Le Kang

Here is the lists for the files we have uploaded to GigaDB.

1) All the sequences files for SET genes in this study: 1.allSETgeneSequence.tar.gz

2) The alignment based and non-alignment based phylogeny trees and the MAFFT alignment file in the Figure 1: 2.PhylogeneticTreeSETgenes.tar.gz

3) The BUSCO-based "species tree" which is used for phylogeny inference of insect orders in the Figure 2: 3.BUSCOSpeciesTree.tar.gz

4) The MAFFT alignment file for BUSCO-based single-copy genes: 4.BUSCOalignment.tar.gz

5) HMMER output file for SET domain identification: 5.HMMERout.tar.gz

6) PSILC program output file: 6. PSILCoutput.tar.gz

7) InterProScan output file: 7. InterProScanoutput.tar.gz

8) The phylogeny trees include in the supplementary files (in Newick format): 8. PhylogenyTreesinSupplyFiles.tar.gz

9) The improved gene models using transcriptome data: 9.RevisedGeneModels.tar.gz

1. For the fasta file of CDS and protein translations, do you have references or accession numbers for how this was put together? This needs to be in a supplemental file if it isn't already.

Response:

All the accession numbers for the SET genes involved in this study were provided in the supplemental Table in our previous submission.

2. Table S1 has some inconsistencies (its hard to check as its pdf rather than CSV file, but for ZNEV you use a DIFFERENT species codename in the table (ZOONE) that needs correcting) and the "Genome Database" column is not very useful because it just gives generic link to the massive archives without exact accessions for the genomes used. And an unstable looking Chinese ftp site. Is this going into the INSDC databases like the SRA?

Response:

A five-letter abbreviation for species name is used throughout the manuscript. For example, ZOONE is an abbreviation for the species name Zootermopsis nevadensis. ZNEV (for example, ZNEV_05631 stands for the G9A gene in ZOONE) is used as

the leading letters for accession number in the official gene sets which are released by the Zootermopsis nevadensis genome sequencing consortium. Therefore, there is no inconsistency in the supplemental Tables.

The “version” column indicates the exact database version involved in this study. In the revision we have provided the extract web path for the databases in the supplementary file.

All the sequences deposited in our web server have been uploaded to GigaDB. This GigaDB database server can provide high-quality and stable services for data retrieval in future.

3. We also will need the data backing up statements like: "Fluorescence in situ hybridization analysis and in vitro methyltransferase activity assays showed that". Also, is this the same data as "Images for fluorescence signals were acquired using an LSM 710 confocal fluorescence microscope (Zeiss)."?

Response:

Yes, this is the same data as "Images for fluorescence signals were acquired using an LSM 710 confocal fluorescence microscope (Zeiss). The data for these two analyses are shown in the Figure 4.

4. You used CEGMA to extract 455 single copy genes, so we need the alignment files for those and the newick trees they generated from them. CEGMA isn't updated, so would be better to replace this with BUSCO.

Response:

In this revision we replace our CEGMA results with the results generated from BUSCO and revised the manuscript accordingly. We have uploaded the alignment files and the newick trees of BUSCO data to GigaDB.

5. Looking at table1.2016102701.xls, how do they make their totals up? for example

if you look at row 12

Diptera Aedes (2) 11-12 1 2 3-4 2-3 3-4

1-2 11-12 34-38

the difference between the range in the total is only 4 yet the differences in the values is 6? i.e. the total should be 34-40 not 34-38. row 13 has an even bigger difference.

Response:

The dash is used to represent the range of SET gene number in each genus. Because the gene numbers for different conserved SET groups are variable, the range of SET gene number could be summed up as the addition of the lower limits to upper limits of gene number in the same genus. The exact gene number for different groups in a species are shown in the supplementary Table 3. As shown in the supplementary Table 3, there are two species in the genus Aedes. The gene numbers for the two species are 11:0(1):2:3:2:3:2:11 and 12:0(1):2:4:3:4:1:12, respectively. The sum of these numbers are 34 (11+0+2+3+2+3+2+11) and 38 (12+0+2+4+3+4+1+12), respectively. The numbers in parenthesis indicates the number of the genes which are not present in the official gene sets. This statement is provided in the table note of the supplementary Table 3. To improve clarity, we add the sentence “The exact gene numbers for different groups in a species are shown in the supplementary Table 3.” in the table note of Table 1 in the revision.

6. There are no alignment files provided anywhere, so we would need the MAFFT output alignments. And tree files for both sorts of the phylogentic analysis (alignment based and non-alignment based), and the "species tree" used (all in Newick or other common tree format).

Response:

We have uploaded these files to GigaDB.

We should also get the following other files a) HMMER* output file; b) the multi-fasta alignments to support the statement "obvious incorrect gene models were improved with transcriptome data"; C) The PSILC program output file, for evidence

of pseudogenes; d)The InterProScan output file.

Response:

We have uploaded these files to GigaDB.

You also need to cite HMMER in the manuscript.

Response:

In the previous submission the HMMER paper was already cited in the second paragraphs of the Materials and Methods section.

From: GigaScience EdOffice

To: Le Kang

Subject: GigaScience, GIGA-D-16-00127 - data queries

CC: database@gigasciencejournal.com

GIGA-D-16-00127

Comparative genomic analysis of SET-domain family reveals the origin, expansion, and putative function of the arthropod-specific SmydA genes as histone modifier in insects

Feng Jiang; Qing Liu; Yanli Wang; Jie Zhang; Huimin Wang; Tianqi Song; Meiling Yang; Xianhui Wang; Le Kang

GigaScience

Dear Le Kang,

Apologies for the slow follow up, but things have been a bit hectic with travels and ICG. We've gone through your paper and have the following questions and requirements for data before this can be sent to review.

1. For the fasta file of CDS and protein translations, do you have references or accession numbers for how this was put together? This needs to be in a supplemental file if it isn't already.

2. Table S1 has some inconsistencies (its hard to check as its pdf rather than CSV file, but for ZNEV you use a DIFFERENT species codename in the table (ZOONE) that needs correcting) and the "Genome Database" column is not very useful because it just gives generic link to the massive archives without exact accessions for the genomes used. And an unstable looking Chinese ftp site. Is this going into the INSDC databases like the SRA?

3. We also will need the data backing up statements like: "Fluorescence in situ hybridization analysis and in vitro methyltransferase activity assays showed that". Also, is this the same data as "Images for fluorescence signals were acquired using an LSM 710 confocal fluorescence microscope (Zeiss)."?

4. You used CEGMA to extract 455 single copy genes, so we need the alignment files for those and the newick trees they generated from them. CEGMA isn't updated, so would be better to replace this with BUSCO.

5. Looking at table1.2016102701.xls, how do they make their totals up? for example if you look at row 12

Diptera Aedes (2) 11-12 1 2 3-4 2-3 3-4 1-2 11-12 34-38

the difference between the range in the total is only 4 yet the differences in the values is 6? i.e. the total should be 34-40 not 34-38. row 13 has an even bigger difference.

6. There are no alignment files provided anywhere, so we would need the MAFFT output alignments. And tree files for both sorts of the phylogentic analysis (alignment

based and non-alignment based), and the "species tree" used (all in Newick or other common tree format).

We should also get the following other files a) HMMER* output file; b) the multi-fasta alignments to support the statement "obvious incorrect gene models were improved with transcriptome data"; C) The PSILC program output file, for evidence of pseudogenes; d)The InterProScan output file.

You also need to cite HMMER in the manuscript.

Please work with our curators (cc'd) to get this (and any other data they highlight) and also send any changes to the manuscript and supplemental files for the paper to us and we will replace them in the submission.

Let us know if you have any questions.

Best wishes,

Scott

Manuscript
Click here to download Manuscript insectsSetDomain_giga_notrack_17021701.doc

Comparative genomic analysis of *SET*-domain family reveals the origin, expansion, and putative function of the arthropod-specific *SmydA* genes as histone modifier in insects

Feng Jiang[1,*], Qing Liu[1,2,*], Yanli Wang[2,3], Jie Zhang[1], Huimin Wang[1], Tianqi Song[3], Meiling Yang[2], Xianhui Wang[2,#], Le Kang[1,2,#]

[1] Beijing Institutes of Life Science, Chinese Academy of Sciences, Beijing, China

[2] State Key Laboratory of Integrated Management of Pest Insects and Rodents, Institute of Zoology, Chinese Academy of Sciences, Beijing, China

[3] Institute of Applied Biology, Shanxi University, Taiyuan, Shanxi, China

*These authors contributed equally to this study.

Corresponding authors:

Le Kang, Ph.D. and Professor

Institute of Zoology, Chinese Academy of Sciences

Beijing 100101, China

Tel: 86-10-6480-7219

Fax: 86-10-6480-7099

E-mail: lkang@ioz.ac.cn

OR

Xianhui, Wang Ph.D. and Professor

Institute of Zoology, Chinese Academy of Sciences

Beijing 100101, China

Tel: 86-10-64807220

Fax: 86-10-6480-7099

E-mail: wangxh@ioz.ac.cn

Evolution of *SET* Genes in Insects

Abstract

The *SET* domain is an evolutionarily conserved motif present in histone lysine methyltransferases, which are important in the regulation of chromatin and gene expression in animals. In this study, we searched for *SET* domain-containing genes (*SET* genes) in all of the 147 arthropod genomes sequenced so far to understand the evolutionary history by which *SET* domain have evolved in insects. Phylogenetic and ancestral state reconstruction analysis revealed an arthropod-specific *SET* gene family, named *SmydA*, which is ancestral to arthropod animals and specifically diversified during insect evolution. Considering that pseudogenization is the most probable fate of the new emerging gene copies, we provided experimental and evolutionary evidence to demonstrate their essential functions. Fluorescence *in situ* hybridization analysis and *in vitro* methyltransferase activity assays showed that the *SmydA-2* gene was transcriptionally active and retained the original histone methylation activity. Expression knockdown by RNA interference significantly increased mortality, implying that the *SmydA* genes may be essential for insect survival. We further showed predominantly strong purifying selection on the *SmydA* gene family and a potential association between the regulation of gene expression and insect phenotypic plasticity by transcriptome analysis. Overall, these data suggest that the *SmydA* gene family retains essential functions that may possibly define novel regulatory pathways in insects. This work provides insights into the roles of lineage-specific domain duplication in insect evolution.

Key words: insects, domain, gene duplication, histone modification.

Background

Protein domains are functional and structural units that are evolutionary well conserved across species [1]. Specific protein domains are often linked to discrete biological function; therefore, the frequent duplication, gain, and loss of protein domains play substantial roles in functional novelty [2]. Domain duplication can be achieved via frequent domain-containing gene family expansion. Thus, the member number of a gene family that contains domains can be expanded, representing a common method by which divergence to domain sequences can lead to the evolutionary novelty of domain-containing genes [3]. Rapid domain diversification in particular lineages is important for the adaptation of lineage-specific ecological specializations [4].

Histones are highly alkaline proteins in cell nuclei that package and order the nuclear DNA into nucleosomes, which are the main components of chromatin. Histone modifications are a major epigenetic regulatory mechanism for phenotypic plasticity in insects. Inhibition of histone deacetylation affects developmental plasticity both in ants (*Camponotus floridanus*) and honeybees (*Apis mellifera*) [5, 6]. Genome-wide profiling of histone modifications revealed an important role of histone H3 lysine 27 acetylation in the caste differentiation of ants [7]. Methylations of histone H3 lysine 27 and histone H3 lysine 36 are more abundant in queen ovaries than in larvae, implying that histone methylation plays a specific role in honey bees [8]. In recent years an increasing number of publications have established histone lysine methylation as a central epigenetic modification in regulation of chromatin and transcription. The *SET* domain, which is observed in many histone lysine methyltransferases, is widely and probably universally distributed in metazoan

species. This protein family typically comprises an approximately 130 amino acid-long *SET* domain, which was identified in the strongest PEV suppressor gene Su(var)3-9, in the Pc-G gene Enhancer of zeste [E(z)] and in the activating trx-G gene Trithorax of *Drosophila* [9]. The *SET* domain possesses a catalytic activity that transfers a methyl group to the amino group of lysine residues of nuclear histones from S-adenosyl-L-methionine. Based on their biochemical characteristics, *SET* domain is capable of catalyzing mono-, di- or tri-methylation of their lysine substrates. *SET* domain-dependent methylation has been identified in a wide range of lysine residues in different histones: K4 (K is the abbreviation for lysine), K9, K27, K36, and K79 in histone H3; K20 in histone H4; K59 in the globular domain of histone H4; and K26 in histone H1B [10]. Methylation of lysine residues in histone proteins is an important post-translational epigenetic event that regulates gene expression by serving as an epigenetic marker for the recruitment of complexes that participate in the organization of chromatin structure [11]. The importance of *SET*-domain containing genes is strongly supported by the involvement of this protein family in diverse biological mechanisms, such as transcriptional activation, transcriptional repression, enhancer function, mRNA splicing and DNA replication [12]. Therefore, expectedly, the regulation of various *SET*-domain containing genes are increasing correlated with diverse epigenetic phenomena which, for example, include epigenetic control in plants, centromeric gene silencing in yeasts, repeat-induced point mutations in fungi, DNA elimination in *Tetrahymena*, germline chromatin silencing in worms and heterochromatin formation in flies [13].

Insects constitute a remarkably diverse group of organisms that make up a vast majority of known species with their importance including biodiversity, agricultural,

and human health concerns. The insect lineage comprises species that are both cosmopolitan distributed and geographically restricted, showing a broad range of adaptation diversity. The evolutionary history of gene families is not confounded by whole-genome duplication, and the major topology of insect species is well resolved [14]. Therefore, the insect lineage offers an excellent model to study domain/gene evolution in the context of gene family dynamics [15-19]. Insect *SET* domain-containing genes (*SET* genes) have been identified in a limited number of representative insect species without complicated analysis [20-22]. The *Smyd* subfamilies of *SET* genes have expanded in a few insects from Diptera and Hymenoptera, and several members of the *Smyd* subfamilies show significant changes in gene expression in response to phenotypic plasticity in ants [23, 24]. However, the evolutionary history of insect *SET* genes remains largely unknown because the *SET* genes from a broad range of insect species have not been combined in a single evolutionary framework. Therefore, a comprehensive study of the origin and diversification of the *SET* gene family in insects is required. Accurate classification of *SET*-domain containing genes can pave the fundamental way to further understanding the epigenetic basis of gene regulation in insects.

In the present study, we aimed to ascertain the origin and diversification of *SET* genes in insects. We searched for *SET* genes in the 130 insect genomes and the 17 arthropod genomes as outgroups. These 130 insect species include both hemimetabolous and holometabolous insects and cover all the insect species for which genome data have been fully available and annotated so far. Our phylogenetic analysis revealed that an important diversification of arthropod-specific *SET* genes, *SmydA*, occurred during insect evolution. Experimental evidence of the important

functions of *SmydA* genes in insects was obtained through fluorescence *in situ* hybridization, *in vitro* methyltransferase activity assay, and survival assay after expression knockdown. Furthermore, we compared the gene expression patterns and examined the selection signatures of *SmydA* genes in the four representative insects exhibiting phenotypic plasticity. These results provide insights into the regulatory roles of lineage-specific domain duplication in insect evolution.

Results

Identification and phylogenetic classification of *SET* genes

We comprehensively searched for *SET* genes in a wide range of sequenced insect species, which included 130 insect species from 14 insect orders (Supplementary Table S1). The *SET* genes were defined by the presence of the *SET* domain as predicted by the HMMER search, and their gene models were manually improved. Seventeen non-insect arthropods were also included to achieve ancestral status along with insect evolution. In total, 4,498 *SET* genes were identified in the 147 arthropod genomes (Supplementary Table S2). The genes showing potential pseudogene signals were removed in these identified *SET* genes. A database webserver (http://159.226.67.242:8080/) has been constructed to select, retrieve, and analyze the data in this study. In insects, the number of *SET* genes found per species ranges from 16 in the scuttle fly *Megaselia scalaris* to 81 in the mosquito *Culex quinquefasciatus* (Table 1 and see Supplementary Table S3 for the full list of summary of *SET* genes in the 147 arthropod genomes). This observation suggests that the size of *SET* genes varies significantly among different insect lineages Although the genome size of the migratory locust *Locusta migratoria* is approximately 30-fold that of the fruit fly

Drosophila melanogaster [25] , the number of *SET* genes in locusts is comparable with that of flies. The specificity of certain substrates is reflected by the classification of *SET* genes, and *SET* genes can be classified into seven major conserved groups, namely: Suv, Ash, Trx, E(z), PRDM, SMYD, and SETD [20]. We performed phylogenetic analysis of the *SET* genes for representative species to obtain insights into the evolution of insect *SET* genes. Multiple sequence alignments of complete proteins could not accurately determine the homologous sites of *SET* genes because of the considerably different sequence lengths and domain architectures of these genes. Thus, alignment-based methods using Bayesian inferences for *SET* domain sequences and alignment-free methods based on feature frequency profiles for complete protein sequences were conducted to infer phylogenetic relationships. The overall tree topologies (Figure 1) inferred using the two methods were generally consistent. Based on the previous nomenclature system [20], the phylogenetic tree topology allows the grouping of insect *SET* genes into seven major conserved groups, generally showing slight fluctuation in the member sizes in each conserved group. The protein domains for each *SET* gene were annotated using the InterProScan package. In general, the *SET* genes in the same conserved group exhibited a similar domain composition, suggesting that the domain architectures support the conserved group classification inferred through the phylogenetic analysis. In addition to the *SET* genes in the conserved groups, a large number of *SET* genes could not be classified into known conserved groups on the basis of the phylogenetic analysis. These unclassified genes act as potential “arthropod-specific” genes. Indeed, a large number of these *SET* genes are homologuous to the already defined arthropod-specific *SmydA* genes described in

the previous study [24]. The lineage-specificity was further verified through reciprocal BLAST search against known *SET* genes of nematodes and humans.

Ancestral states of the *SET* gene family in insects

A character matrix that represents the present/absent states for each *SET* homologous group (a OrthoMCL-based homolog set including both putative orthologs and paralogs) was constructed to infer the ancestral states of interior nodes along with the species tree using the Mesquite program. The ancestral states at different nodes could infer the emergences/losses of the *SET* homologous group that occurred at and above the level of orders (Figure 2). The grouping of *SET* homologous genes for each species was inferred using the OrthoMCL program with the corresponding orthologous *SET* gene in *D. melanogaster*, and the grouping reliability was supported by the phylogenetic analysis (Supplementary Figure S1–S5). The putative ancestral state was composed of 19 *SET* homologous groups present in the last common ancestor (LCA) of the studied arthropod species. Generally, the insect species possessed more *SET* homologous groups than the chelicerata species studied, suggesting that *SET* homologous groups considerably expanded during insect evolution. At the interior clades, novel *SET* homologous groups emerged several times. Only few losses of *SET* homologous groups, such as the loss of *SmydA-3*, were observed at the interior clades. The large fluctuation of *SET* homologous groups in each species indicates that these groups experienced rapid lineage-specific expansion/contraction within insect orders. For example, in Hymenoptera, the number of *SET* homologous groups ranged from 18 (covering 23 *SET* genes) in the jumping ant *Harpegnathos saltator* to 30 (covering 52 *SET* genes) in the parasitoid wasp

Nasonia vitripennis. In Diptera, 13 *SET* homologous groups (covering 14 *SET* genes) were found in *M. scalaris*, and the oriental fruit fly *Bactrocera dorsalis* possessed only 31 *SET* homologous groups (covering 45 *SET* genes). A large number of arthropod specific *SET* homologous groups cannot be classified into the seven major conserved groups, which revealed their origin after the emergence of main arthropod lineages. Nevertheless, at least six of these groups were present among insect species belonging to different orders, indicating their broad conservation in insects (Figure 2A).

SET domains do not just function as an independent unit, as in many proteins it co-occurs with multiple other protein domains to regulate their target specificity and catalysis [12]. We surveyed the gene ontology (GO) classification of proteins by integrating biological knowledge into three hierarchies, namely, biological process, molecular function, and cellular component, to assess the function innovation of domain acquisition globally. The common GO categories included histone lysine methylation (GO:0034968), regulation of transcription (GO:0006355), protein binding (GO:0005515), nucleic acid binding (GO:0003676), and metal ion binding (GO:0046872) (Figure 3A). Partitioning of *SET* gene families between the conserved and arthropod specific groups revealed that GO categories could be shared between the two groups or be assigned exclusively to one group. The GO categories, which were only exclusive in the arthropod specific groups, included RNA methyltransferase activity (GO:0008173), metallocarboxypeptidase activity (GO:0004181), lysozyme activity (GO:0003796), homophilic cell adhesion (GO:0007156), sulfotransferase activity (GO:0008146) and so on.

Evolution of *SET* Genes in Insects

Emergence of arthropod lineage-specific *SET* gene families

Pairwise BLAST search against all the *SET* genes indicated that the arthropod specific *SET* genes showed considerable amino acid similarity to the SMYD groups, which contain a conserved core consisting of a *SET* domain and a MYND (Myeloid translocation protein, Nervy, Deaf) zinc finger domain [26]. The arthropod specific *SET* genes also contain the *SET* and MYND domains and were named *SmydA* [24]. We performed the phylogenetic analysis of the SMYD genes through Bayesian inferences. The majority of the SMYD genes could be classified into 11 monophyletic clades, which exhibited similar high Bayesian posterior probability values (Figure 3B). In a global view, these SMYD genes fell into two distinct branches, which correspond with the conserved SMYD and *SmydA* groups. These results could exclude the possibility that the *SmydA* groups have raised from multiple independent gain events by duplications from deeply diverged SMYD genes of insects. Indeed, as shown in Figure 2A, *SmydA* genes were absent from in all Chelicerata species investigated but present in the genomes of crustacean species and insect species, suggesting that *SmydA* genes may have originated prior to the divergence of Crustacea and Insecta. *SmydA-1*, *SmydA-2*, *SmydA-3,* and *SmydA-6* were already present before the split of Crustacea with other insects, showing clues for their ancient duplication events. The strong support for distinct individual lineages of paralogous genes implied that multiple duplications occurred within the order level; the most notable case was the detection of three copies of *SmydA-3* in the red flour beetle *Tribolium castaneum* (Supplementary Table 2). *SmydA-1/SmydA-4* and *SmydA-6* were subjected to additional rounds of duplication in Lepidoptera and Orthoptera, respectively. The genes annotated as *SmydA-8* and *SmydA-9* in *D. melanogaster* previously formed a

238 single clade alone with a high Bayesian posterior probability value (0.99), suggesting
239 a specific duplication event in *Drosophila*. Therefore, the *SmydA* groups differed
240 considerably in the number of genes in each insect order, implying the complexity of
241 their evolutionary histories.

242 To shed light into the evolutionary history of *SmydA* genes, we determined the
243 location and gene order of *SmydA* genes in the four holometabolous species with
244 available chromosome-level genome assemblies or genome-scale genetic linkage
245 maps (Figure 3C). In Diptera, the syntenic gene orders could be inferred from the four
246 ancient *SmydA* genes, namely, *SmydA-1*, *SmydA-2*, *SmydA-3*, and *SmydA-6*, all of
247 which may have been present in the ancestor of insects and crustaceans. An
248 insect-specific *SmydA-9* could be observed in the majority of insect orders, including
249 both hemimetabolous and holometabolous insects. *SmydA-9* showed syntenic
250 conservation with the four ancient genes. This gene order was also conserved when
251 *SmydA* genes in insects distantly related from other insect orders were examined.
252 Almost all of the five synteny-anchoring genes were maintained in both the
253 coleopteran species *T. castaneum* and hymenoptera species *A. mellifera*, with an
254 exception of *SmydA-2* that was missed in *A. mellifera*. In contrast to those in *T.*
255 *castaneum* and *A. mellifera*, the reversed order of *SmydA-3* and *SmydA-6* in Dipteran
256 species implies that an intrachromosome transfer event of genomic segments occurred
257 before the emergence of Diptera. Duplication events could also occur in the early
258 diversification of arthropod species. No orthologous *SmydA-4* gene was detected the
259 chelicerata species, indicating that duplication event contributes to the emergence of
260 *SmydA-4* gene in Pancrustacea species. *SmydA-4* was present in all the
261 hemimetabolous insect orders studied, as well as in the holometabolous insect orders

Lepidoptera, Coleoptera, and Diptera. The absence of *SmydA-4* in all the 32 hymenopteran species suggested that subsequent loss of *SmydA-4* could be traced back to the ancestor of the hymenopteran lineage before the divergence of wasp, ants, and bees. In the SMYD phylogenetic tree, the Bayesian inferences supported the grouping of *SmydA-3*, *SmydA-4*, and *SmydA-6*. Three of the four species exhibited a accordant location of *SmydA-3*/*SmydA-4/SmydA-6* in the syntenic regions. In addition to the old duplication events that categorized the divergent duplicates into distinct *SmydA* subfamilies (e.g., *SmydA-3* and *SmydA-4*), recent duplications within an insect order were also observed. The three copies of *SmydA-3* in *T. castaneum*, which spanned within a 4.2 kb genomic region, were observed in tandem array between the two syntenic genes *SmydA-1* and *SmydA-6*. The closeness in protein sequence and genomic location implies an evolutionary origin of these three copies of *SmydA-3* via local duplication. Overall, our data suggest that the order of *SmydA* genes was conserved over a remarkable wide range of holometabolous insect orders.

Selective pressures acting on *SmydA* genes

Functional differentiations or mutations leading to pseudogene formation are the two major causes for sequence divergence between new duplicates and their orthologous counterpart. Synonymous substitutions are assumed to accumulate at a constant rate; hence, the ratios of nonsynonymous substitution per nonsynonymous site (d_N) to synonymous substitution per synonymous site (d_S) are deemed to be an indicator to measure the relative rates of evolution for protein sequences. The four genes (ACYPI26757 and ACYPI55839 in *Acyrthosiphon pisum*; Px015362.1 and Px001029.1 in *Plutella xylostella*) showing signals of recombination were removed

from the further selection analysis. We estimated a global d_N/d_S ratio (one ratio, model M0) for these *SET* genes to determine whether the *SmydA* genes have been under different selection pressures than the other conserved *SET* genes. The d_N/d_S ratios (ω = d_N/d_S ratio) of *SET* genes varied from low (0.0007, Ez, CG6502) to high (0.1627, *Smyd4-1*, *CG1868*), indicating a variance in the rates of protein evolution on different *SET* genes (Table 2). The ω values among the conserved *SET* genes (excluding the SMYD genes) ranged from 0.0007 to 0.0624 (mean ω = 0.0185). The conserved SMYD and *SmydA* groups showed ω values in the ranges of 0.055–0.1627 (mean ω = 0.1020) and 0.0052–0.1623 (mean ω = 0.0884), respectively. Overall, both the conserved SMYD and *SmydA* (P = 0.0003 and P = 0.0178, Wilcoxon signed-rank tests with Bonferroni correction, respectively) groups exhibited significantly higher ω values than the conserved *SET* genes (Figure 3D). However, the distributions of ω values of the conserved SMYD and *SmydA* groups were statistically indistinguishable (P = 1.0000, Wilcoxon signed-rank tests with Bonferroni correction).

Function approval of *SmydA* genes

We attempted to determine whether the *SmydA* genes retained histone methylation activities to approve the non-pseudogenization process of these genes. We expressed *SmydA-2* as a randomly selected representative and performed *in vitro* histone methylation activity assays using histones as substrates in the migratory locust. As shown in Figure 4A, Western blot analysis detected increased lysine methylation on histone H3 compared with the controls, indicating that *SmydA-2* possesses methyltransferase activity on histones. Similar to that of the other conserved SMYD genes, the methyltransferase activity of *SmydA-2* was also dependent on S-adenosyl

methionine. Fluorescence *in situ* hybridization analysis provided further tissue expression evidence to support the reliability of the *SmydA-2* gene function. Obvious fluorescence signals were observed in the brain and epidermal cells of cuticle in the locusts (Figure 4B). These cells did not show any hybridization signal for the negative controls. The origin and evolution of new emerging genes undergo an increased expression breadth of new duplicated genes over evolutionary time [27, 28]. Thus, we determined the expression levels of the *SmydA-2* gene using quantitative real-time polymerase chain reaction (qPCR) analysis in the different tissues. qPCR data showed that the *SmydA-2* gene was expressed in a broad range of tissues, including brains, testes, ovaries, cuticles, and legs (Figure 4C). The broad expression pattern suggests that the *SmydA-2* gene is less tissue specific and may serve as a functional gene in multiple tissues [28].

Essential genes are often considered as conserved and functionally important [29] , whereas pseudogenes have been considered to be more dispensable and to have minor influences on survival and phenotype. To determine whether the *SmydA-2* gene plays an essential role during development [30], we knocked its expression down by using RNA interferences in the locusts. Compared with the controls, the relative mRNA level of the *SmydA-2* gene decreased by approximately 70% after injecting double-strand RNAs (Supplementary Figure S6). After injection of *dsSmydA-2,* we observed large numbers of dead locusts, which did not display obvious defect phenotype. As shown in Figure 4D, Kaplan–Meier survival estimates indicate that injection of locusts with *dsSmydA-2* significantly increased mortality when compared with the controls (χ^2 = 6.260, df = 1, P = 0.0123, Chi-square tests).

Evolution of *SET* Genes in Insects

Expression and selection analysis of *SmydA* genes in response to phenotypic plasticity

Epigenetic reprogramming that modifies chromatin structure through histone modifiers contributes to orchestrate the generation and maintenance of phenotypic plasticity, which is a key trait for the success of insects. Therefore, we compared the expression patterns of histone-modifier *SET* genes in four representative insects exhibiting phenotypic plasticity, namely, locust density-dependent behavior, aphid seasonal morphs, dietary-mediated interactions of bees and ants. Specially, we performed differential expression analysis between gregarious and solitary locusts, between asexual and sexual morphs in *A. pisum*, between queens and workers in *A. mellifera*, and between large workers and queens in *Acromyrmex echinatior*. In all the four species, a number of differentially expressed genes (DEGs) were detected between the two alternative phenotypes using the criteria of a false discovery rate (FDR)-corrected $P < 0.05$. In terms of DEG number, a large portion of *SET* genes showed significant changes in gene expression (12 in 29, 41%, in *A. mellifera*; 23 in 62, 37%, in *A. pisum*;11 in 29, 38%, in *L. migratoria*; and 10 in 27, 37%, in *A. echinatior*). Compared with that of the DEGs observed at the genome-wide level (DEGs in total), the number changes of the DEGs in *SET* genes in the four insects were even more prominent, emphasizing the important regulatory role of *SET* genes in phenotypic transition (*Ps* < 0.05, Chi-square tests). Overlapping of the differentially expressed *SET* genes derived from the same ortholog could provide a clue of their convergent function in phenotypic transition. We found two *SET* genes, namely, *Set2* and *SmydA-5*, showed significant changes in gene expression simultaneously in three of the four insect species studied.

Evolution of *SET* Genes in Insects

Assuming that a non-pseudogene gene should not be randomly expressed, we compared the expression pattern of the duplication-derived *SmydA* genes to their derived ancestral SMYD genes in response to environment-dependent phenotypic plasticity (Figure 5). The majority of *SET* genes from the conserved SMYD (33 in 34 in total, 97%) and *SmydA* (13 in 17 in total, 76%) groups were expressed in at least one insect. No significant differences (P = 0.749, Chi-Square tests) in the number of expressed genes were observed between the two groups. A number of DEGs were detected in both the conserved SMYD and *SmydA* groups in the four insect species. All the four *SmydA* genes in *A. echinatio*r were also differentially expressed. We also obtained significant results in three of the six *SmydA* genes of *L. migratoria* and in two of the five *SmydA* genes of *A. mellifera* between the two alternative phenotypes. The DEG number in the *SmydA* groups did not show significant deviation from those in the conserved SMYD group in the four insects (*Ps* > 0.2, Fisher's exact tests). This result suggests that the *SmydA* genes might not be randomly expressed and that they did not represent pseudogenes or transcriptional byproducts. Thus, the *SmydA* genes may preserve a regulatory role, indicating the function similarity to their ancestral SMYD genes.

The free ratio model of *SmydA* genes fitted the data significantly better than the one model (model M0) using likelihood ratio tests (*Ps* < 0.001), indicating heterogeneous rates of sequence evolution along the gene tree of *SmydA* genes. Therefore, we tested whether the differentially expressed *SmydA* genes between alternative phenotypes (foreground branches) evolved under different selective pressures than those in the remaining branches (background branch) (Supplementary Figure S7). The branch model was much better supported by the data than the model

M0 for *SmydA-5* in *A. mellifera* and *SmydA-1* in *L. migratoria* (Table 3). Fixing ω = 1 for the foreground branch did not result in an improved fit over the branch model with the unconstrained foreground branch (the null neutral model and the alternative model). This result suggests that the ω values in the external branch were smaller than 1 for *SmydA-3* and *SmydA-5* in *A. mellifera*, *SmydA-1* in *L. migratoria*, and *SmydA-3* in *A. echinatio*r. Only *SmydA-1* in *L. migratoria* exhibited elevated ω values, and a branch-site model allowing heterogeneous ω values across sequences and branches identified four sites (5M, 11K, 93P, and 105C) under positive selection.

Discussion

In this study, the phylogenetic analyses allowed the subdivision of the insect *SET* genes into seven major conserved groups and one arthropod-specific *SmydA* group. We inferred many *SmydA* gene duplication events along insect evolution, suggesting an important diversification of the *SmydA* genes occurred during insect evolutionary processes. With the *SmydA-2* genes in locusts as representatives, the maintenance of essential gene function was confirmed from the experimental evidence of *in vitro* methyltransferase activity, *in situ* mRNA expression, and phenotypes after expression knockdown. Based on the examination of distribution pattern and selection signatures across insects, our data indicated that extensive pseudogenization unlikely occurred for the *SmydA* genes. Finally, the transcriptome analyses of the four insects showed that several *SmydA* genes are involved in insect phenotype plasticity, suggesting that *SmydA* genes contributed novelties for insect adaptive evolution. This data suggests a role of diverged regulatory functions after their duplication in insects.

404 A recent study has provided a framework for understanding the evolution history
405 of SMYD gene family in representative animal phyla [24]. The phylogenetic results
406 show that the metazoan SMYD genes can be classified in three main classes, *Smyd3*,
407 *Smyd5* and *Smyd4*. Two sub-classes of SMYD genes, namely *Smyd4-4* and *SmydA*,
408 are absent in vertebrates; the former on is insect-specific and the later one is
409 arthropod-specific. Within Chelicerata, we detected *Smyd4-4* in Acariform mites
410 (non-insect arthropods), suggesting our evidence did not support the point that
411 *Smyd4-4* is specific of insects. Since Chelicerata represents an out-group branch for
412 this study, further studies covering more basal branches of arthropod phylogeny are
413 required to ascertain the origin of *Smyd4-4*. *SmydA* genes represent a class of
414 arthropod-specific genes that are only present in the LCA of insect species and
415 crustacean species, suggesting their origin after the split of chelicerates from
416 Pancrustacea species. Conservation of five ancient *SmydA* genes in a wide range of
417 species suggests they probably originated from duplication events of conserved
418 SMYD genes predating the diversification of insects. Although a few cases of
419 whole-genome duplication have been documented in chelicerates, evidence that
420 whole-genome duplication occurs widely in arthropod evolution remains lacking [31].
421 Therefore, gene duplication rather than whole-genome duplication possibly leads to
422 the emergence of multiple copies of ancient *SmydA* genes in the LCA of Pancrustacea
423 species. The clear split of conserved SMYD and *SmydA* genes excluded the
424 possibility that multiple independent duplication events from conserved SMYD genes
425 resulted in the current repertoire of *SmydA* genes in insects. This result suggests that
426 the five ancient *SmydA* genes were first produced from a single ancestral gene, which
427 was derived from conserved SMYD genes. The five ancient *SmydA* genes were thus

the source from which insect-specific *SmydA* duplications were subsequently produced in insects. Determining the location and order of multiple gene members at the genomic scale sheds light on the evolutionary history of gene family. The closely linked manner in genomic location suggests that homologous recombination and functional differentiation may be a major force to shape the evolution of *SmydA* genes in insects. For instance, in dipteran and lepidopteran insects, homologous recombination may give rise to *SmydA-6* via the duplication events of *SmydA-3* because *SmydA-3* and *SmydA-6* were in close proximity to each other in both genomic location and phylogenetic trees. The tandem organization of three *SmydA-3* copies in *T. castaneum* may also result from species-specific duplications via homologous recombination. Retrotransposition events may represent another contributing force for generating unlinked *SmydA* genes; these events can also generate intronless retroposed gene copies [32]. However, the retrotransposition events could not be inferred from the presence of signature of intron–exon structure because of the subsequent insertion in deeply diverged duplicates, such as *SmydA-5*. Conserved gene orders between species from Lepidoptera, Coleoptera, and Diptera revealed a high degree of macrosyntenic gene order of the five ancient *SmydA* genes during approximately 348 million years of evolutions splitting these insects [33]. This observation implies strong constraints for preserving the conserved gene order of *SmydA* genes in insects. Currently, whether this macro-syntenic gene order is preserved outside holometabolous insects cannot be determined because chromosome-level genome assemblies or genome-scale genetic linkage maps are not available in hemimetabolous insects. This issue would be addressed when the genome assembly is considerably improved in the future.

Evolution of *SET* Genes in Insects

Selective pressures were significantly weaker for the SMYD genes than for the six conserved groups (Suv, Ash, Trx, E(z), PRDM, and SETD). Compared with the six conserved groups, SMYD genes were the least conserved gene group and, concordantly, the least constrained one. Nevertheless, the ω values of SMYD genes ranged from 0.0052 for *SmydA-2* to 0.1627 for *Smyd4-1*. $\omega << 1$ was consistent with their broad conservation across insects, implying their essential functional roles. This observation suggests that purifying selection is the main force governing the evolution of SMYD genes. The distributions of ω values of the conserved SMYD and *SmydA* gens were statistically indistinguishable, indicating a symmetrical rate of sequence evolution. Thus, purifying selection is subject to the conserved SMYD and *SmydA* genes, but their intensity may be relaxed compared with other *SET* genes. Both the GO analysis and the *in vitro* methyltransferase activity assay suggest that *SmydA* genes, similar to their conserved SMYD ancestors, are sufficient to perform the original function relating to histone methylation [34]. GO ontology analysis implied that the *SmydA* genes have developed to acquire novel functions. These functions were absent in the conserved SMYD genes, indicating that the *SmydA* genes may have undergone functional differentiation. Gene duplications that occurred in specific lineages are important in contributing to lineage-specific adaptive processes [35]. After gene duplication, purifying selection is expected in both gene copies if duplication can confer a selective advantage [36]. By contrast, one of the two copies can evolve either under relaxed purifying selection when no immediate advantage is shown from gene duplication or under positive selection when a new function is acquired via advantageous mutations [37]. Overall, these data suggest that the *SmydA* genes may not represent redundant gene copies that are under pseudogenization.

Evolution of *SET* Genes in Insects

Several members of the SMYD family of histone methyltransferases have undergone a dramatic expansion in the insect lineage [23]. These SMYD genes were identified as caste-specific genes in ants (*Harpegnathos saltator*), suggesting that these histone modifiers play dedicated regulatory roles in insect phenotypic plasticity. However, the biological significance of the differential expressions of these genes remains unknown [38]. Our study further verified the presence of the differential expression patterns of the SMYD genes in the four other insects that also possessed adaptive phenotypic plasticity. Consequently, the understanding of convergent regulatory roles of the SMYD genes in insect phenotypic plasticity was extended. Histone lysine methyltransferase catalyzes methyl group transfer to the amino group of lysine residues of histones by means of the *SET* domain, a domain presented within many proteins that regulate diverse development processes [39]. Histone lysine methylation on specific residues is associated with distinct signatures of gene expression, thereby serving as a chromatin modulator for epigenetic regulation [40]. Future studies should understand how the expanded SMYD gene family can quickly become essential and identify the roles of the duplicated SMYD genes in insects, despite the expectation of redundant functionality at the beginning of new duplicated gene evolution [30].

Materials and Methods

Identification of insect *SET* genes

Genome assemblies and official gene sets of 130 insect species, including 62 dipteran insects, 33 hymenopteran insects, 10 hemipteran insects, 7 coleopteran species, 9 lepidopteran insects, and representatives from Orthoptera, Phthiraptera,

Phasmatoptera, Trichoptera, Thysanoptera, Isoptera, Blattodea, Ephemeroptera and Odonata, were obtained from their respective genome databases (Supplementary Table S1). Among the basal arthropod species, we included 17 arthropod genomes from 10 chelicerate species, five crustacean species and two non-insect hexapod species.

The hidden Markov model-based HMMER program was used to identify the *SET* domain containing proteins using PF00856 in the Pfam database with a conditional E-value cutoff of 1e-5 [41, 42]. Despite that the *SET* domain can be detected in their homologs in closely related species, the genes lacking SET domain were considered as deprived of lysine methylation capacity and were excluded for further analysis. The resulting genes with stop codons or frameshift mutations were subsequently manually checked. The obvious incorrect gene models were improved with transcriptome data through the GeneWise version 2.2.0 program [43]. The PSILC version 1.21 program was used to identify the potential pseudogenes [44]. Gene Ontology (GO) categories were determined via scanning protein sequences against Interpro member databases using various profile-based and hidden Markov models in the InterProScan version 5.13-52.0 package [45]. The member database binaries and models include TIGRFAM, ProDom, Panther, SMART, PrositePatterns, SuperFamily, PRINTS, Gene3d, PIRSF, PfamA and PrositeProfiles.

Phylogenetic analysis, ancestral state reconstructions, and tests for selection

Alignment-based methods using Bayesian inferences for *SET* domain sequences and alignment-free methods based on feature frequency profiles for complete protein sequences were used to infer phylogenetic relationships of *SET* genes across insects.

Multiple alignments were generated using the MAFFT alignment software [46]. According to the Akaike information criterion, the model of molecular evolution with the best fit to the data was determined by using the ProtTest 3.4.2 software [47]. Bayesian reconstruction of phylogeny was conducted using the MrBayes 3.2.1 software for 10,000,000 generations [48]. The first 25% of the trees were discarded as burn-in. The alignment-free and distance-based methods for phylogenetic tree building were implemented by means of the feature frequency profile method with the FFP version 3.19 suite (http://sourceforge.net/projects/ffp-phylogeny/), utilizing the FFPaa program for amino acid sequences with a word length of $L = 5$. The FFPboot program was used for bootstrap analysis of the tree generated for 100 replicates.

We constructed a character matrix that represents present/absent states for each *SET* homologous group to reconstruct the ancestral states of interior clades. We did not consider member number variation and considered only the binary state, presence or absence, of a given *SET* homologous group in any given node. The grouping of the *SET* genes was inferred from the OrthoMCL software with the corresponding orthologous *SET* gene in *D. melanogaster*. Ancestral state reconstruction was implemented in the Mesquite program (http://mesquiteproject.org/) under maximum likelihood optimization using Markov k-state 1 parameter model. After ancestral reconstruction, we measured emergence and loss events of *SET* homologous group along each branch in the phylogenetic tree. The emergence event of *SET* homologous group was defined as the *SET* homologous group was absent at the ancestral nodes of a given node and either of the outgroups This process requires a phylogeny tree of all the species studied. Single-copy orthologous gene families were inferred from the benchmarking universal single-copy ortholog BUSCO gene sets from each species

[49]. The resulting 527 single-copy orthologous (completed genes in BUSCO) gene families were used to construct the neighbor-joining species tree, which is consistent with the phylogenomic tree recently inferred from transcriptome data [14]. The neighbor-joining species tree was constructed from amino acid sequences of single-copy orthologs using Phylip version 3.69 package. The bootstrap values, calculated from 100 replicates using the seqboot, protdist, neighbor and consense programs of Phylip package.

Expression of SMYD family genes in response to phenotypic plasticity

The transcriptome data for gregarious and solitary locusts in *L. migratoria*, asexual and sexual morphs in *A. pisum*, queens and workers in *A. mellifera*, and minor and major workers in *A. echinatior* were retrieved from the NCBI database under accession numbers PRJNA79681, GSE56830, GSE61253, and GSE51576, respectively. The raw reads were preprocessed to remove adapters and low-quality bases using the Trimmomatic software; these reads were then mapped to the genome assembly (genome assembly version: v2.4 for *L. migratoria*, v1.0 for *A. pisum*, Amel_2.0 for *A. mellifera* and Aech_v2.0 for *A. echinatior*, respectively) using the Tophat2 version 2.0.14 software [50, 51]. Raw counts of each gene were calculated and annotated using the HT-seq version 0.6.1 package in Python, and the trimmed mean of M value normalization method was used to normalize raw counts [52]. Differential expression analysis was performed using the edgeR version 3.8.0 package at an FDR cut-off of 0.05 [53].

Function approval of *SmydA-2* genes via experimental evidence

A fluorescence *in situ* analysis of *SmydA-2* was performed on the brains and integuments of locust nymphs. Biotin-labeled antisense and sense probes (Supplementary Table S4) of *SmydA-2* were produced from pGEM-T Easy plasmids (Promega) by using the T7/SP6 RNA transcription system (Roche) following the manufacturer’s protocol. The PCR parameters were a preincubation 94 °C for 5 min, followd by 30 cycles of 94 °C for 10 sec, 58 °C for 30 sec, 72 °C for 30 sec, and a final extension at 72 °C for 10min. The brains and integuments were fixed in 4% paraformaldehyde overnight. The paraffin-embedded slides (5 µm thick) were deparaffinized in xylene, rehydrated with an ethanol gradient, digested with 20 µg/mL proteinase K (Roche) at 37 °C for 15 min, and then incubated with *SmydA-2* probe at 60 °C for 5 min. The slides were hybridized for 7–15 h at 37 °C and washed in 0.2×SSC and 2% BSA at 4 °C for 5 min. The biotin-labeled probes of *SmydA-2* were detected with a streptavidin horseradish peroxidase conjugate and fluorescein tyramide substrate using a TSA kit (Perkin Elmer). Images for fluorescence signals were acquired using an LSM 710 confocal fluorescence microscope (Zeiss).

The recombinant proteins for *SmydA-2* and the negative controls of translation system were produced using the TNT protein expression system (Promega) following the manufacturer’s protocol. In brief, 3 µg PCR-generated DNA templates (Supplementary Table S4) were added to 30 µl TNT master mix, and the translation reactions were incubated at 25 °C for 2 h. The recombinant proteins were verified by Western blotting using His-tag antibodies. For *in vitro* methyltransferase assay, 2 mg of unmodified histone H3 peptides (Sino Biological) were incubated with 1 mg of recombinant protein and 0.1 mM S-adenosyl-methionine (SAM, NEB) in a reaction

buffer containing 50 mM Tris-HCl (pH 8.0), 10% glycerol, 20 mM KCl, 5 mM $MgCl_2$, 1 mM DTT, and 1 mM PMSF at 30 °C for 2 h. The reaction mixtures were subjected to electrophoresis on SDS-PAGE, and the methylation activities were detected in Western blotting using anti-pan methyl lysine antibody (Abcam). Anti-histone H3 (Abcam) was used as endogenous control for protein samples.

Locusts (the migratory locust, *Locusta migratoria*) were reared in large, well-ventilated cages (40 cm × 40 cm × 40 cm) at a density of 500–1000 insects per container. These colonies were reared under a 14:10 light/dark photo regime at 30 °C and were fed fresh wheat seedlings and bran. Double-stranded RNAs of *SmydA-2* and green fluorescent protein (GFP) were prepared using the T7 RiboMAX Express RNAi system (Promega) in accordance with the manufacturer's protocols. Second-instar locusts were injected with double-stranded RNAs in the second ventral segment of the abdomen. Total RNAs were isolated using TRIzol reagent (Thermo Fisher Scientific) and then reverse-transcribed into cDNA using M-MLV reverse transcriptase (Promega). The mRNA levels were quantified using the SYBR Green expression assays on a LightCycler 480 instrument (Roche). The parameters were a pre-incubation 95°C for 10 min, followed by 45 cycles of 95 °C for 10 sec, 58 °C for 20 sec, and a single acquisiton when 72 °C for 20 sec. The ribosomal protein 49 gene was used as reference control, and the quantification was based on the requirement of PCR cycle number to cross or exceed the fluorescence intensity level; the $2^{-\Delta\Delta Ct}$ method was used to analyze mRNA expression levels. Survival data were analyzed using the Kaplan–Meier method [54], and survival curves were compared using log-rank testing for the *dsSmydA-2* and *dsGFP* curves.

Evolution of *SET* Genes in Insects

Signature of selection detected through likelihood ratio tests

Protein sequences of *SET* genes were aligned with the MAFFT alignment software [46] and the back-translated into corresponding nucleotide sequences. Gene conversion was detected using the recombination detection program GENECONV version 1.81a. To assess the contribution of natural selection during the diversification of the *SET* gene family in insects, the ratios of nonsynonymous substitution per nonsynonymous site (d_N) to synonymous substitution per synonymous site (d_S) across the phylogenetic tree of the species were calculated using the software package PAML version 4.48a [55]. The basic model M0 (null model) assumes the ratio $\varpi = d_N/d_S$ is invariable (one-ratio model) among all branches examined, whereas the alternative model allows the ϖ ratio to vary in different tree branches in the phylogenetic tree [56, 57]. Likelihood ratio tests were applied to compare the null and alternative models, which estimated ϖ ratio separately for different branches, assuming a priori and the background branches. A significantly higher likelihood of the alternative model than the null model indicates a better fit to the data, indicating a variation of selective pressures in different tree branches [56, 57].

Declarations

List of abbreviations

SET genes, *SET* domain-containing genes; E(z), Enhancer of zeste; LCA, last common ancestor; GO, gene ontology; MYND, Myeloid translocation protein; qPCR, quantitative real-time polymerase chain reaction; DEGs, differentially expressed

genes; FDR, false discovery rate; SAM, S-adenosyl-methionine; GFP, green fluorescent protein; PP, posterior probability

Ethics approval and consent to participate

All animal procedures were licensed under the Institutional Animal Care and Use Committee of the Institute of Zoology, Chinese Academy of Sciences.

Consent for publication

Not applicable

Competing interests

The authors declare they have no competing interests.

Funding

This research was supported by the following grants: National Natural Science Foundation of China Grants (Nos. 31301915, 31472051, and 31672353) and Strategic Priority Research Program of the Chinese Academy of Sciences (No. XDB11010000).

Authors' contributions

F.J., X.W., and L.K conceived and designed the experiments. F.J. and Q. L analyzed and interpreted the data. F.J., Q. L., Y.W., J.Z., H.W., T.S., and M.Y. performed the experiments. F.J., Q.L., and L.K wrote the paper.

Acknowledgements

The computational resources were provided by the Research Network of Computational Biology and the Supercomputing Center at Beijing Institutes of Life Science, Chinese Academy of Sciences.

Availability of supporting data and materials

The dataset supporting the conclusions of this article is available in http://159.226.67.242:8080/.

References

1. Elofsson A, Sonnhammer EL: **A comparison of sequence and structure protein domain families as a basis for structural genomics**. *Bioinformatics* 1999, **15**(6):480-500.
2. Itoh M, Nacher JC, Kuma K, Goto S, Kanehisa M: **Evolutionary history and functional implications of protein domains and their combinations in eukaryotes**. *Genome Biol* 2007, **8**(6):R121.
3. Sakarya O, Conaco C, Egecioglu O, Solla SA, Oakley TH, Kosik KS: **Evolutionary expansion and specialization of the PDZ domains**. *Mol Biol Evol* 2010, **27**(5):1058-1069.
4. Moore AD, Bornberg-Bauer E: **The dynamics and evolutionary potential of domain loss and emergence**. *Mol Biol Evol* 2012, **29**(2):787-796.
5. Simola DF, Graham RJ, Brady CM, Enzmann BL, Desplan C, Ray A, Zwiebel LJ, Bonasio R, Reinberg D, Liebig J *et al*: **Epigenetic (re)programming of caste-specific behavior in the ant Camponotus floridanus**. *Science* 2016, **351**(6268):aac6633.
6. Spannhoff A, Kim YK, Raynal NJ, Gharibyan V, Su MB, Zhou YY, Li J, Castellano S, Sbardella G, Issa JP *et al*: **Histone deacetylase inhibitor activity in royal jelly might facilitate caste switching in bees**. *EMBO Rep* 2011, **12**(3):238-243.
7. Simola DF, Ye C, Mutti NS, Dolezal K, Bonasio R, Liebig J, Reinberg D, Berger SL: **A chromatin link to caste identity in the carpenter ant Camponotus floridanus**. *Genome Res* 2013, **23**(3):486-496.
8. Dickman MJ, Kucharski R, Maleszka R, Hurd PJ: **Extensive histone post-translational modification in honey bees**. *Insect Biochem Mol Biol* 2013, **43**(2):125-137.
9. Jenuwein T, Laible G, Dorn R, Reuter G: **SET domain proteins modulate chromatin domains in eu- and heterochromatin**. *Cell Mol Life Sci* 1998, **54**(1):80-93.

10. Dillon SC, Zhang X, Trievel RC, Cheng X: **The SET-domain protein superfamily: protein lysine methyltransferases**. *Genome Biol* 2005, **6**(8):227.
11. Boros IM: **Histone modification in Drosophila**. *Brief Funct Genomics* 2012, **11**(4):319-331.
12. Herz HM, Garruss A, Shilatifard A: **SET for life: biochemical activities and biological functions of SET domain-containing proteins**. *Trends Biochem Sci* 2013, **38**(12):621-639.
13. Jenuwein T: **The epigenetic magic of histone lysine methylation**. *FEBS J* 2006, **273**(14):3121-3135.
14. Misof B, Liu S, Meusemann K, Peters RS, Donath A, Mayer C, Frandsen PB, Ware J, Flouri T, Beutel RG *et al*: **Phylogenomics resolves the timing and pattern of insect evolution**. *Science* 2014, **346**(6210):763-767.
15. Ferguson LC, Green J, Surridge A, Jiggins CD: **Evolution of the insect yellow gene family**. *Mol Biol Evol* 2011, **28**(1):257-272.
16. Helmkampf M, Cash E, Gadau J: **Evolution of the insect desaturase gene family with an emphasis on social Hymenoptera**. *Mol Biol Evol* 2015, **32**(2):456-471.
17. Tanaka K, Diekmann Y, Hazbun A, Hijazi A, Vreede B, Roch F, Sucena E: **Multispecies Analysis of Expression Pattern Diversification in the Recently Expanded Insect Ly6 Gene Family**. *Mol Biol Evol* 2015, **32**(7):1730-1747.
18. Urena E, Pirone L, Chafino S, Perez C, Sutherland JD, Lang V, Rodriguez MS, Lopitz-Otsoa F, Blanco FJ, Barrio R *et al*: **Evolution of SUMO Function and Chain Formation in Insects**. *Mol Biol Evol* 2016, **33**(2):568-584.
19. Benton R: **Multigene Family Evolution: Perspectives from Insect Chemoreceptors**. *Trends Ecol Evol* 2015, **30**(10):590-600.
20. Zhang L, Ma H: **Complex evolutionary history and diverse domain organization of SET proteins suggest divergent regulatory interactions**. *The New phytologist* 2012, **195**(1):248-263.
21. Vidal NM, Grazziotin AL, Iyer LM, Aravind L, Venancio TM: **Transcription factors, chromatin proteins and the diversification of Hemiptera**. *Insect Biochem Mol Biol* 2016, **69**:1-13.
22. Rider SD, Jr., Srinivasan DG, Hilgarth RS: **Chromatin-remodelling proteins of the pea aphid, Acyrthosiphon pisum (Harris)**. *Insect Mol Biol* 2010, **19 Suppl 2**:201-214.
23. Bonasio R, Zhang G, Ye C, Mutti NS, Fang X, Qin N, Donahue G, Yang P, Li Q, Li C *et al*: **Genomic comparison of the ants Camponotus floridanus and Harpegnathos saltator**. *Science* 2010, **329**(5995):1068-1071.
24. Calpena E, Palau F, Espinos C, Galindo MI: **Evolutionary History of the Smyd Gene Family in Metazoans: A Framework to Identify the Orthologs of Human Smyd Genes in Drosophila and Other Animal Species**. *PLoS One* 2015, **10**(7):e0134106.
25. Wang X, Fang X, Yang P, Jiang X, Jiang F, Zhao D, Li B, Cui F, Wei J, Ma C *et al*: **The locust genome provides insight into swarm formation and long-distance flight**. *Nat Commun* 2014, **5**:2957.

26. Thompson EC, Travers AA: **A Drosophila Smyd4 homologue is a muscle-specific transcriptional modulator involved in development**. *PLoS One* 2008, **3**(8):e3008.
27. Kaessmann H: **Origins, evolution, and phenotypic impact of new genes**. *Genome Res* 2010, **20**(10):1313-1326.
28. Assis R, Bachtrog D: **Neofunctionalization of young duplicate genes in Drosophila**. *Proc Natl Acad Sci U S A* 2013, **110**(43):17409-17414.
29. Krylov DM, Wolf YI, Rogozin IB, Koonin EV: **Gene loss, protein sequence divergence, gene dispensability, expression level, and interactivity are correlated in eukaryotic evolution**. *Genome research* 2003, **13**(10):2229-2235.
30. Kemkemer C, Long M: **New genes important for development**. *EMBO Rep* 2014, **15**(5):460-461.
31. Kenny NJ, Chan KW, Nong W, Qu Z, Maeso I, Yip HY, Chan TF, Kwan HS, Holland PW, Chu KH *et al*: **Ancestral whole-genome duplication in the marine chelicerate horseshoe crabs**. *Heredity (Edinb)* 2016, **116**(2):190-199.
32. Kaessmann H, Vinckenbosch N, Long M: **RNA-based gene duplication: mechanistic and evolutionary insights**. *Nat Rev Genet* 2009, **10**(1):19-31.
33. Hedges SB, Dudley J, Kumar S: **TimeTree: a public knowledge-base of divergence times among organisms**. *Bioinformatics* 2006, **22**(23):2971-2972.
34. Tan X, Rotllant J, Li H, De Deyne P, Du SJ: **SmyD1, a histone methyltransferase, is required for myofibril organization and muscle contraction in zebrafish embryos**. *Proc Natl Acad Sci U S A* 2006, **103**(8):2713-2718.
35. Domazet-Loso T, Tautz D: **An evolutionary analysis of orphan genes in Drosophila**. *Genome Res* 2003, **13**(10):2213-2219.
36. Innan H, Kondrashov F: **The evolution of gene duplications: classifying and distinguishing between models**. *Nat Rev Genet* 2010, **11**(2):97-108.
37. Pegueroles C, Laurie S, Alba MM: **Accelerated evolution after gene duplication: a time-dependent process affecting just one copy**. *Mol Biol Evol* 2013, **30**(8):1830-1842.
38. Bonasio R: **The role of chromatin and epigenetics in the polyphenisms of ant castes**. *Brief Funct Genomics* 2014, **13**(3):235-245.
39. Leinhart K, Brown M: **SET/MYND Lysine Methyltransferases Regulate Gene Transcription and Protein Activity**. *Genes (Basel)* 2011, **2**(1):210-218.
40. Sims RJ, 3rd, Nishioka K, Reinberg D: **Histone lysine methylation: a signature for chromatin function**. *Trends Genet* 2003, **19**(11):629-639.
41. Finn RD, Mistry J, Tate J, Coggill P, Heger A, Pollington JE, Gavin OL, Gunasekaran P, Ceric G, Forslund K *et al*: **The Pfam protein families database**. *Nucleic Acids Res* 2010, **38**(Database issue):D211-222.
42. Finn RD, Clements J, Eddy SR: **HMMER web server: interactive sequence similarity searching**. *Nucleic Acids Res* 2011, **39**(Web Server issue):W29-37.
43. Birney E, Clamp M, Durbin R: **GeneWise and Genomewise**. *Genome Res* 2004, **14**(5):988-995.
44. Coin L, Durbin R: **Improved techniques for the identification of pseudogenes**. *Bioinformatics* 2004, **20 Suppl 1**:i94-100.

45. Jones P, Binns D, Chang HY, Fraser M, Li W, McAnulla C, McWilliam H, Maslen J, Mitchell A, Nuka G *et al*: **InterProScan 5: genome-scale protein function classification**. *Bioinformatics* 2014, **30**(9):1236-1240.

46. Katoh K, Asimenos G, Toh H: **Multiple alignment of DNA sequences with MAFFT**. *Methods Mol Biol* 2009, **537**:39-64.

47. Darriba D, Taboada GL, Doallo R, Posada D: **ProtTest 3: fast selection of best-fit models of protein evolution**. *Bioinformatics* 2011, **27**(8):1164-1165.

48. Ronquist F, Teslenko M, van der Mark P, Ayres DL, Darling A, Hohna S, Larget B, Liu L, Suchard MA, Huelsenbeck JP: **MrBayes 3.2: efficient Bayesian phylogenetic inference and model choice across a large model space**. *Syst Biol* 2012, **61**(3):539-542.

49. Simao FA, Waterhouse RM, Ioannidis P, Kriventseva EV, Zdobnov EM: **BUSCO: assessing genome assembly and annotation completeness with single-copy orthologs**. *Bioinformatics* 2015, **31**(19):3210-3212.

50. Bolger AM, Lohse M, Usadel B: **Trimmomatic: a flexible trimmer for Illumina sequence data**. *Bioinformatics* 2014, **30**(15):2114-2120.

51. Trapnell C, Pachter L, Salzberg SL: **TopHat: discovering splice junctions with RNA-Seq**. *Bioinformatics* 2009, **25**(9):1105-1111.

52. Robinson MD, Oshlack A: **A scaling normalization method for differential expression analysis of RNA-seq data**. *Genome Biol* 2010, **11**(3):R25.

53. Robinson MD, McCarthy DJ, Smyth GK: **edgeR: a Bioconductor package for differential expression analysis of digital gene expression data**. *Bioinformatics* 2010, **26**(1):139-140.

54. Kaplan EL, Meier P: **Nonparametric-Estimation from Incomplete Observations**. *J Am Stat Assoc* 1958, **53**(282):457-481.

55. Yang Z: **PAML 4: phylogenetic analysis by maximum likelihood**. *Mol Biol Evol* 2007, **24**(8):1586-1591.

56. Yang Z: **Likelihood ratio tests for detecting positive selection and application to primate lysozyme evolution**. *Mol Biol Evol* 1998, **15**(5):568-573.

57. Yang Z, Nielsen R: **Synonymous and nonsynonymous rate variation in nuclear genes of mammals**. *J Mol Evol* 1998, **46**(4):409-418.

Figures

Figure 1. Phylogenetic analysis of *SET* genes in insects. A phylogeny using Bayesian inference is generated from the domain protein sequence of *SET* genes. One representative is elected for each order. The protein domains, which are labeled with different colors based on the domain type, are shown in the exterior circle of the phylogenetic tree. The length of the grey long line after each terminal is directly

proportional to the length of the corresponding *SET* gene. The branch colors of the phylogenetic tress indicate the established *SET* gene classification which divides *SET* genes into seven major conserved groups, namely: Suv, Ash, Trx, E(z), PRDM, SMYD, and SETD. The *SET* genes labeled in black branches cannot be classified into the seven major conserved groups, suggesting their arthropod origin. The representative species include *Apis mellifera*, *Daphnia pule*, *Drosophila melanogaster*, *Ixodes scapularis*, *Locusta migratoria*, *Pediculus humanus*, *Plutella xylostella*, *Rhodnius prolixus*, *Tetranychus urticae*, *Timema cristinae* and *Tribolium castaneum*.

Figure 2. Diversification of arthropod-specific *SET* genes. (A) Distribution pattern of *SET* genes in arthropod orders. One representative is elected for each order. Red color indicates presence of *SET* genes, and blue color indicates absence of *SET* genes. (B) Inference of ancestral sets of *SET* homologous groups along the evolution of insects. The gains and losses of *SET* homologous groups are indicated in the internal nodes of the phylogenetic tree. The number in parentheses indicates the number of species in each order. The bars indicate the number ranges of *SET* homologous groups in each order.

Figure 3. Evolution of *SmydA* genes in insects. (A) Gene ontology categories of the conserved and arthropod-specific groups of *SET* genes. The gene ontology categories, which are only present in the arthropod-specific group, are highlighted in red. (B) Phylogenetic tree of the SMYD gene family of the representative species selected from each order. The representative species include *Apis mellifera*, *Daphnia pule*,

Drosophila melanogaster, *Ixodes scapularis*, *Locusta migratoria*, *Pediculus humanus*, *Plutella xylostella*, *Rhodnius prolixus*, *Tetranychus urticae*, *Timema cristinae* and *Tribolium castaneum*. The phylogenetic tree is constructed using the Bayesian inference method. The Bayesian posterior probability (PP) values are indicated only for the internal nodes to improve clarity; consequently, the *SET* genes are grouped into different monophyletic clades (SMYD subfamilies). Red and orange circles indicate PP > 90% and PP > 70%, respectively. (C) Conserved syntenies for *SmydA* genes in four holometabolous species. Shown from top to bottom are *Drosophila melanogaster*, *Anopheles gambiae*, *Tribolium castaneum* and *Apis mellifera*. (D) Distributions of ω ($\omega = d_N/d_S$ ratio) values of the conserved SMYD and *SmydA* groups of *SET* genes.

Figure 4. Function approval of *SmydA-2* genes through experimental evidence. (A) *In vitro* methyltransferase assay of histone H3 of *SmydA-2* in locusts. Anti-pan methyl lysine antibody recognizes histone H3 *in vitro* methylated with *SmydA-2*. Anti-histone H3 serves as endogenous control for protein samples. The analyses were carried out in three replicates. **$P < 0.01$. (B) Expression evidence of *SmydA-2* in the brain and cuticle of locusts via fluorescence *in situ* hybridization analysis. Green signals indicate the expression of *SmydA-2* /control, and blue signals indicate nuclear staining with Hoechst. (C) Relative gene expression of *SmydA-2* in the different tissues. mRNA levels are quantified using the SYBR Green expression assays on a LightCycler 480 instrument. The qPCR data are shown as the mean ± SEM ($n = 6$). (D) Survival analysis of the locusts after *SmydA-2* double-strand RNA injection. Data

are analyzed through the Kaplan–Meier survival curve comparison of the *dsSmydA-2* and *dsGFP* groups for three replicates.

Figure 5. Differential expression analysis in insects showing phenotype plasticity. Alternative phenotype includes gregarious and solitary phases in *Locusta migratoria* (LOCMI), asexual and sexual morphs in *Acyrthosiphon pisum* (ACYPI), queens and workers in *Apis mellifera* (APIME), and large workers and queens in *Acromyrmex echinatior* (ACREC).

Tables

Table 1. Summary of *SET* genes in insect genomes.

Table 2. Tests of rate heterogeneity acting on *SET* genes in insects.

Table 3. Signatures of selection acting on differentially expressed *SET* genes in response to phenotypic plasticity.

Supplementary Data

Supplementary Table S1. The arthropod genome data involved in this study.

Supplementary Table S2. *SET* genes in the 147 arthropod genomes.

Supplementary Table S3. Summary of *SET* genes in the 147 arthropod genomes.

Supplementary Table S4. Primers used in the study.

Supplementary Figure S1. Phylogenetic analysis of the *SET* genes in Lepidoptera using Maximum-likelihood inferences with PhyML. The *SET* gene families labeled

with different colors are shown in the exterior circle of the phylogenetic tree. The insect species involved are represented with different colors of the external branch.

Supplementary Figure S2. Phylogenetic analysis of the *SET* genes in Diptera using Maximum-likelihood inferences with PhyML. The *SET* gene families labeled with different colors are shown in the exterior circle of the phylogenetic tree. The insect species involved are represented with different colors of the external branch. The representative species are selected to improve clarity.

Supplementary Figure S3. Phylogenetic analysis of the *SET* genes in Hemiptera using Maximum-likelihood inferences with PhyML. The *SET* gene families labeled with different colors are shown in the exterior circle of the phylogenetic tree. The insect species involved are represented with different colors of the external branch.

Supplementary Figure S4. Phylogenetic analysis of the *SET* genes in Hymenoptera using Maximum-likelihood inferences with PhyML. The *SET* gene families labeled with different colors are shown in the exterior circle of the phylogenetic tree. The insect species involved are represented with different colors of the external branch. The representative species are selected to improve clarity.

Supplementary Figure S5. Phylogenetic analysis of the *SET* genes in Coleopteran using Maximum-likelihood inferences with PhyML. The *SET* gene families labeled with different colors are shown in the exterior circle of the phylogenetic tree. The insect species involved are represented with different colors of the external branch.

Supplementary Figure S6. Effects of RNA interference of the mRNA expression levels of *SmydA-2* in locust brains. The locusts are injected with double-stranded RNAs into the second ventral segment of the abdomen. Due to the systemic RNA interference in locusts, the brain, which is spatially distant from the abdomen, is used

923 in qPCR assays to guarantee effective expression knockdown. qPCR data are shown as the mean ± SEM (n = 6). **P < 0.01.

925 **Supplementary Figure S7. Tree topology and branch labeling for tests of selection on *SET* genes.** APIME, *Apis mellifera*; ACREC, *Acromyrmex echinatior*; LOCMI, *Locusta migratoria*. Supplementary Table S1 presents the abbreviation of insect species.

Table 1

Click here to download Table Table1.17020802.xls

Table 1. Summary of *SET* genes in insect genomes.

Order	Genus	SMYD	SETD	PRDM	Ash	Suv	Trx	Ez	Other	Total
Coleoptera	*Agrilus* (1)	4	1	2	3	3	3	1	9	26
Coleoptera	*Anoplophora* (1)	7	1	2	3	3	3	2	7	28
Coleoptera	*Dendroctonus* (1)	5	1	1	3	3	3	1	12	29
Coleoptera	*Leptinotarsa* (1)	10	1	1	2	5	3	1	9	32
Coleoptera	*Onthophagus* (1)	4	1	1	3	4	3	1	10	27
Coleoptera	*Oryctes* (1)	6	1	1	3	3	1	1	9	25
Coleoptera	*Tribolium* (1)	6	2	1	3	3	3	1	15	34
Phthiraptera	*Pediculus* (1)	6	1	1	3	4	3	1	9	28
Blattodea	*Blattella* (1)	4	2	2	4	3	2	1	7	25
Diptera	*Aedes* (2)	11-12	1	2	3-4	2-3	3-4	1-2	11-12	34-38
Diptera	*Anopheles* (19)	6-19	1	1-2	1-3	2-3	2-3	1	4-11	20-37
Diptera	*Bactrocera* (2)	4-5	1	1-2	3-4	4	3-6	1-2	13-22	31-45
Diptera	*Ceratina* (1)	5	1	1	2	4	3	1	11	28
Diptera	*Ceratitis* (1)	5	1	1	3	3	3	1	14	31
Diptera	*Culex* (1)	40	1	1	13	2	9	1	14	81
Diptera	*Drosophila* (22)	4-5	1	1	3-4	3-5	2-4	1	7-14	24-31
Diptera	*Glossina* (6)	4-5	1	1	3-4	2-5	3-4	1	12-15	29-34
Diptera	*Lucilia* (1)	5	1	1	3	3	3	1	12	29
Diptera	*Lutzomyia* (1)	6	1	1	3	3	2	1	10	27
Diptera	*Mayetiola* (1)	13	1	1	9	6	4	1	25	60
Diptera	*Megaselia* (1)	2	1	1	3	2	1	1	5	16
Diptera	*Musca* (1)	5	1	1	3	3	3	1	20	37
Diptera	*Phlebotomus* (1)	5	1	1	4	3	3	1	6	24
Diptera	*Belgica* (1)	27	2	1	3	5	4	1	12	55
Diptera	*Stomoxys* (1)	5	1	1	3	2	3	1	16	32
Ephemeroptera	*Ephemera* (1)	18	1	1	3	2	2	1	12	40
Hemiptera	*Acyrthosiphon* (1)	14	1	0	2	10	4	1	31	63
Hemiptera	*Cimex* (1)	4	1	2	3	5	3	1	5	24
Hemiptera	*Diaphorina* (1)	3	1	1	4	4	3	2	11	29
Hemiptera	*Gerris* (1)	6	1	1	3	3	3	1	8	26
Hemiptera	*Halyomorpha* (1)	5	1	1	2	5	3	1	8	26
Hemiptera	*Homalodisca* (1)	5	2	2	2	5	4	1	8	29
Hemiptera	*Nilaparvata* (1)	4	1	6	2	4	4	1	7	29
Hemiptera	*Oncopeltus* (1)	6	1	1	2	5	4	1	7	27
Hemiptera	*Pachypsylla* (1)	1	1	2	2	3	1	1	9	20
Hemiptera	*Rhodnius* (1)	6	1	1	2	2	2	1	6	21
Hymenoptera	*Acromyrmex* (1)	7	2	1	3	3	3	1	7	27
Hymenoptera	*Apis* (3)	6-7	1	1	3	3-4	1-3	1	7-9	22-29
Hymenoptera	*Athalia* (1)	7	1	2	2	3	2	1	8	26
Hymenoptera	*Atta* (1)	8	1	1	3	4	3	1	7	28
Hymenoptera	*Bombus* (2)	7-8	1	1	3	4	3	1	8-10	29-30
Hymenoptera	*Camponotus* (1)	8	2	1	2	3	2	1	8	27
Hymenoptera	*Cardiocondyla* (1)	7	2	1	3	4	3	1	10	31
Hymenoptera	*Cephus* (1)	6	1	1	2	3	2	1	6	22
Hymenoptera	*Cerapachys* (1)	5	1	1	2	3	3	1	6	22
Hymenoptera	*Ceratosolen* (1)	8	1	1	3	3	2	1	9	28
Hymenoptera	*Copidosoma* (1)	17	1	1	3	4	2	1	16	45
Hymenoptera	*Dufourea* (1)	7	2	1	3	4	3	1	7	28

Hymenoptera	*Eufriesea* (1)	6	2	1	3	4	3	1	8	28
Hymenoptera	*Fopius* (1)	9	1	1	3	4	1	1	9	29
Hymenoptera	*Habropoda* (1)	8	2	1	3	4	3	1	8	30
Hymenoptera	*Harpegnathos* (1)	8	2	0	1	2	1	1	8	23
Hymenoptera	*Linepithema* (1)	7	2	1	3	4	3	1	8	29
Hymenoptera	*Megachile* (1)	7	2	1	3	3	3	1	8	28
Hymenoptera	*Melipona* (1)	7	2	1	3	4	3	1	8	29
Hymenoptera	*Microplitis* (1)	18	1	1	3	4	3	2	8	40
Hymenoptera	*Monomorium* (1)	6	1	1	2	3	2	1	5	21
Hymenoptera	*Nasonia* (1)	17	1	1	3	4	2	1	23	52
Hymenoptera	*Orussus* (1)	11	2	1	2	3	3	1	7	30
Hymenoptera	*Pogonomyrmex* (1)	5	2	1	2	4	3	1	8	26
Hymenoptera	*Polistes* (1)	6	1	1	1	4	2	1	6	22
Hymenoptera	*Solenopsis* (1)	2	1	1	3	3	3	1	7	21
Hymenoptera	*Trichogramma* (1)	15	1	1	3	4	1	1	26	52
Hymenoptera	*Vollenhovia* (1)	6	1	1	3	4	2	1	3	21
Hymenoptera	*Lasioglossum* (1)	9	1	1	3	3	3	1	8	29
Hymenoptera	*Wasmannia* (1)	7	1	1	3	3	3	1	6	25
Isoptera	*Zootermopsis* (2)	6	1	2	2	4	3	1	10	29
Lepidoptera	*Bombyx* (1)	4	2	1	3	4	3	1	8	26
Lepidoptera	*Danaus* (1)	5	1	1	3	5	3	1	10	29
Lepidoptera	*Heliconius* (1)	5	1	1	2	4	3	1	6	23
Lepidoptera	*Papilio* (2)	6	1	1	3	2-4	2	1	9-11	26-27
Lepidoptera	*Lerema* (1)	4	1	2	3	3	3	1	10	27
Lepidoptera	*Melitaea* (1)	5	1	1	3	1	3	1	8	23
Lepidoptera	*Manduca* (1)	6	2	7	7	5	5	2	29	63
Lepidoptera	*Plutella* (1)	5	4	1	4	5	6	0	13	38
Odonata	*Ladona* (1)	3	2	2	3	4	3	1	9	27
Orthoptera	*Locusta* (1)	9	1	1	3	4	3	1	7	29
Phasmatoptera	*Timema* (1)	3	1	1	3	5	3	1	6	23
Thysanoptera	*Frankliniella* (1)	6	2	8	3	5	3	1	21	49
Trichoptera	*Limnephilus* (1)	3	1	0	2	3	2	1	6	18

[illegible] used to represent the range of *SET* gene number in each genus. The exact gene numbers for different groups in a species are shown in the supplementary Table 3. Other, arthropod-specific and unclassified *SET* genes.

 Click here to download Table Table2.16100701.xls

Table 2. Tests of rate heterogeneity acting on *SET* genes in insects.

	Gene	One Ratio Likelihood	One Ratio ω	Free Ratio Likelihood	df	*P*
SMYD	*Smyd3*	-4833.870633	0.055	-4833.870633	16	<0.001
	Smyd4-1	-17270.85481	0.1627	-17140.2931	58	<0.001
	Smyd4-2	-13187.36796	0.1125	-13112.10598	44	<0.001
	Smyd4-3	-20488.96316	0.1069	-20364.99139	66	<0.001
	Smyd4-4	-15552.36608	0.1112	-15475.97917	44	<0.001
	Smyd5	-21495.43548	0.0633	-21329.01303	64	<0.001
	upSET(MLL5)	-7286.598116	0.0103	-7247.800191	62	0.087
	Set8	-6450.096636	0.0321	-6386.997507	60	<0.001
	Hmt4-20	-3523.660744	0.0079	-3478.339497	56	<0.001
SETD	*SETD*	-9030.115692	0.033	-9009.972504	34	0.212
PRDM	*Blimp-1*	-2679.981724	0.0051	-2664.129882	52	0.988
Ash	*Mes-4*	-5530.425067	0.0163	-5504.225668	56	0.612
	ash1	-4995.315864	0.0122	-4947.987993	60	<0.001
	Set2	-5636.021533	0.0118	-5570.266003	60	<0.001
Suv	*Su(var)3-9*	-4351.473377	0.0212	-4308.872564	32	<0.001
	egg	-15308.27271	0.0624	-15214.54477	54	<0.001
	CG4565	-7168.675146	0.056	-7114.254055	46	<0.001
	G9a	-4641.585219	0.0091	-4604.810574	54	0.040
Trx	*trx*	-3897.22035	0.0031	-3877.624919	58	0.972
	Set1	-3733.003015	0.0026	-3700.07484	60	0.281
	trr	-4549.712	0.0114	-4471.116449	60	<0.001
E(z)	*Ez*	-3368.302419	0.0007	-3355.922925	61	1.000
SMYDA	*SmydA-1*	-10066.85883	0.0904	-9995.276076	34	<0.001
	SmydA-2	-11858.79656	0.0052	-11812.61641	30	<0.001
	SmydA-3	-13902.68842	0.0817	-13842.81154	56	<0.001
	SmydA-4	-9602.742487	0.0254	-9583.599425	26	0.057
	SmydA-5	-13748.76916	0.1179	-13656.26849	50	<0.001
	SmydA-6	-12142.19779	0.1623	-12043.99319	42	<0.001
	SmydA-9	-13258.40628	0.1357	-13193.53611	52	<0.001

Note: Accounting for the unequal genome sequencing efforts between different insect families, we selected one species within each genus to be representative of the genus.

Click here to download Table Table3.16082501.xls

Table 3. Signatures of selection acting on differential expressed *SET* genes in response to phenotypic plasticity.

Model-Parameters	APIME		LOCMI	ACREC		
	SmydA-3	*SmydA-5*	*SmydA-1*	*SmydA-3*	*SmydA-5*	*SmydA-9*
**Basic models**						
M0: ω	0.082	0.118	0.090	0.082	0.118	0.136
**Branch models**						
B0: lnL	-13914.741	-13749.007	-10088.904	-13905.140	-13749.047	-13259.370
B0: ω_0 ($\omega_1 = 1$)	0.077	0.113	0.090	0.081	0.117	0.135
BA: lnL	-13901.138	-13745.405	-10056.182	-13901.922	-13748.719	-13258.338
BA: ω_0, ω_1	0.080, 0.142	0.115, 0.313	0.095, 0.003	0.081, 0.177	0.118, 0.181	0.135, 0.186
**Branch-site models**						
A0: p_{2a} ($\omega_2 = 1$)	0.078	0.059	0.111	0.082	0.155	0.096
AA: p_{2a}', ω_2	0.078, 1.000	0.025, 3.102	0.109, 8.895	0.082, 1.000	0.155,1.000	0.011, 19.742
Positively selected sites (BEB)			5 M 11 K 93 P 105 C			
**LRT, *P***						
M0 versus BA	0.078	0.009	<0.001	0.216	0.752	0.712
BA versus B0	<0.001	0.007	<0.001	0.011	0.418	0.151
A0 versus AA	1.000	0.802	0.022	1.000	1.000	0.082

ω, the ratios of nonsynonymous substitution per nonsynonymous site to synonymous substitution per synonymous site; ω0, ω1, background and foreground ω values, respectively; APIME, *Apis mellifera* ; ACREC, *Acromyrmex echinator* ; LOCMI, *Locusta migratoria.*

Figure 1

Click here to download Figure figure1_20181006.pdf

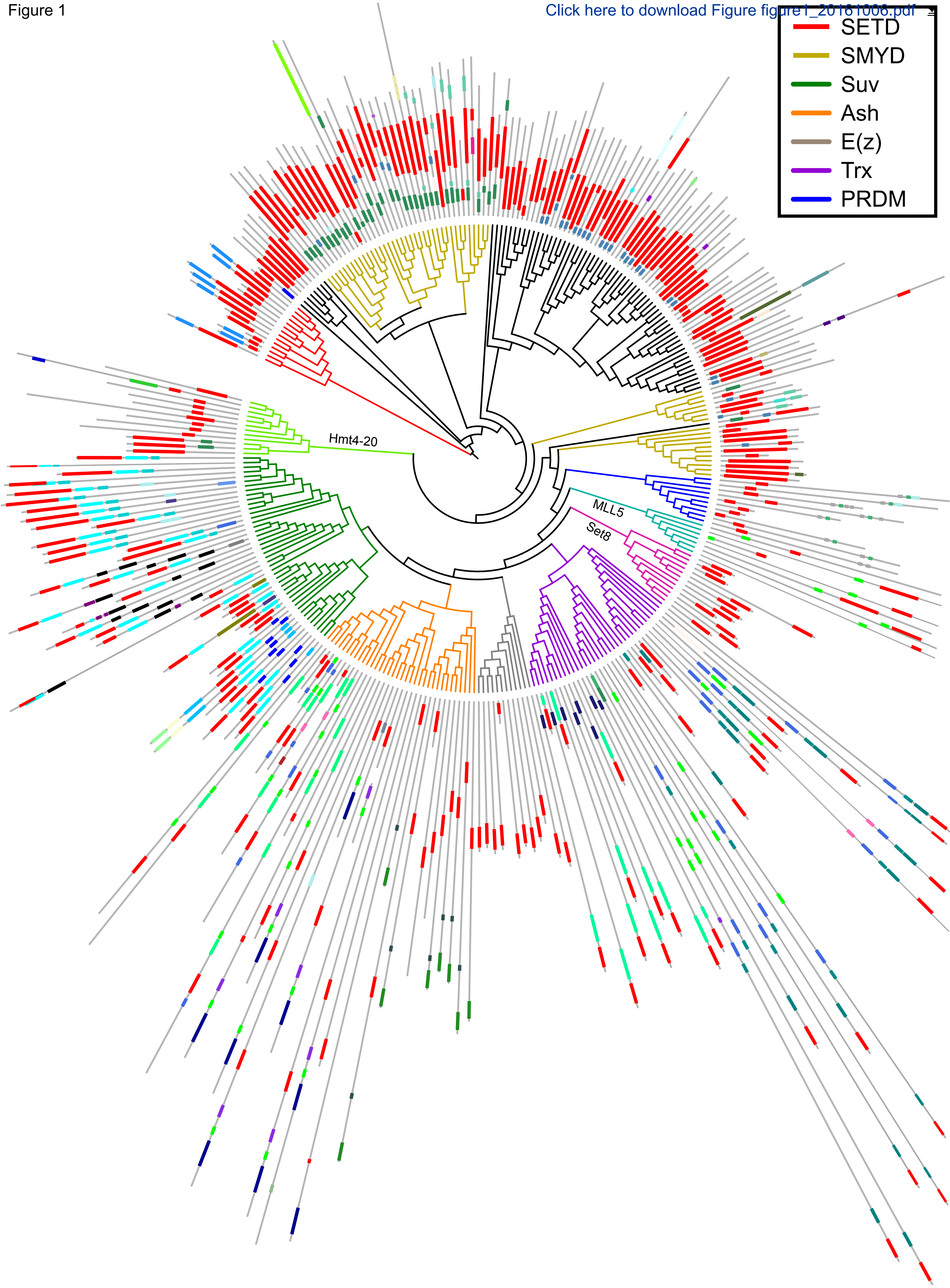

Figure 2

Click here to download Figure figure2_2017020801.pdf

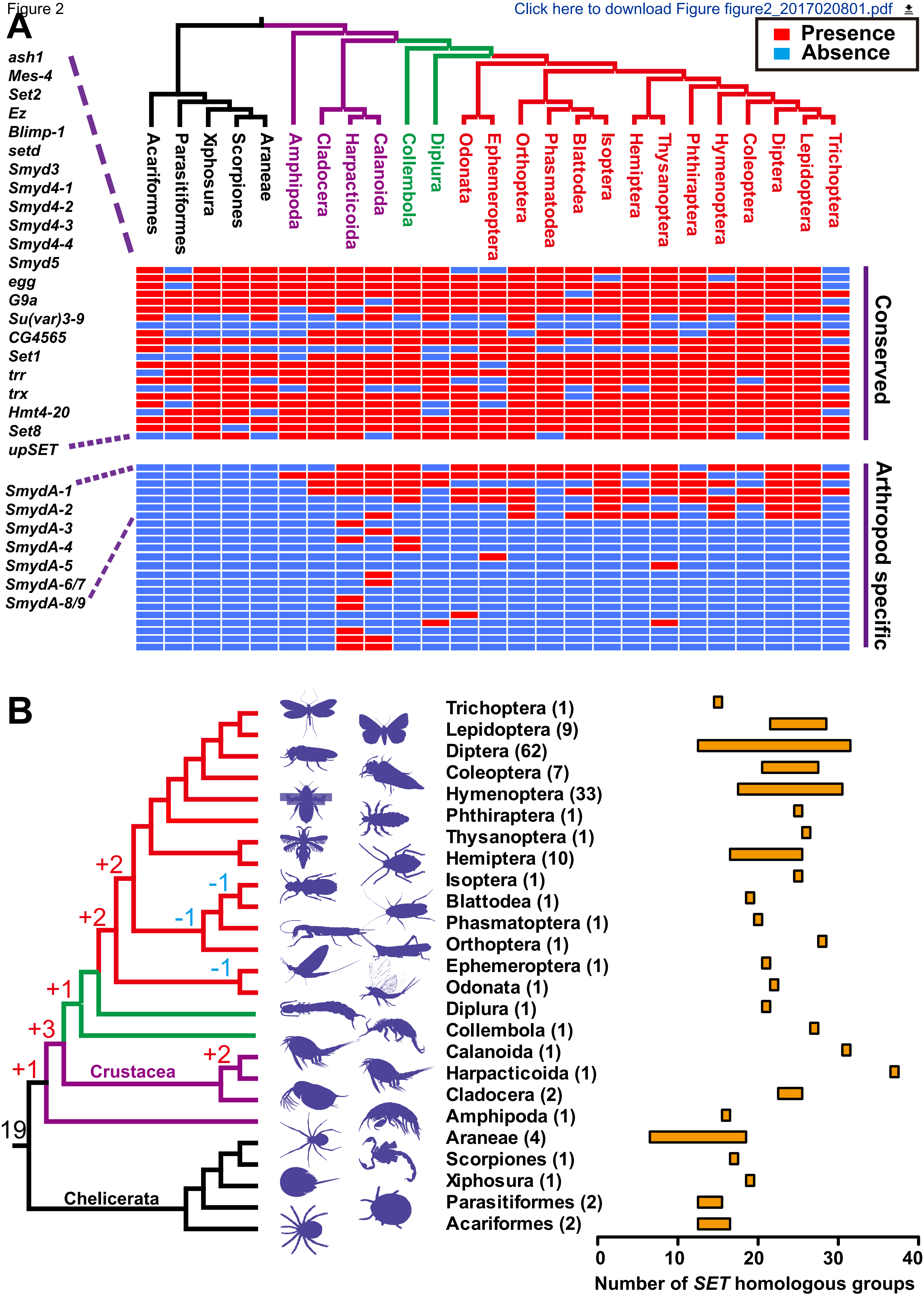

Figure 3

Click here to download Figure figure3_2017020801.pdf

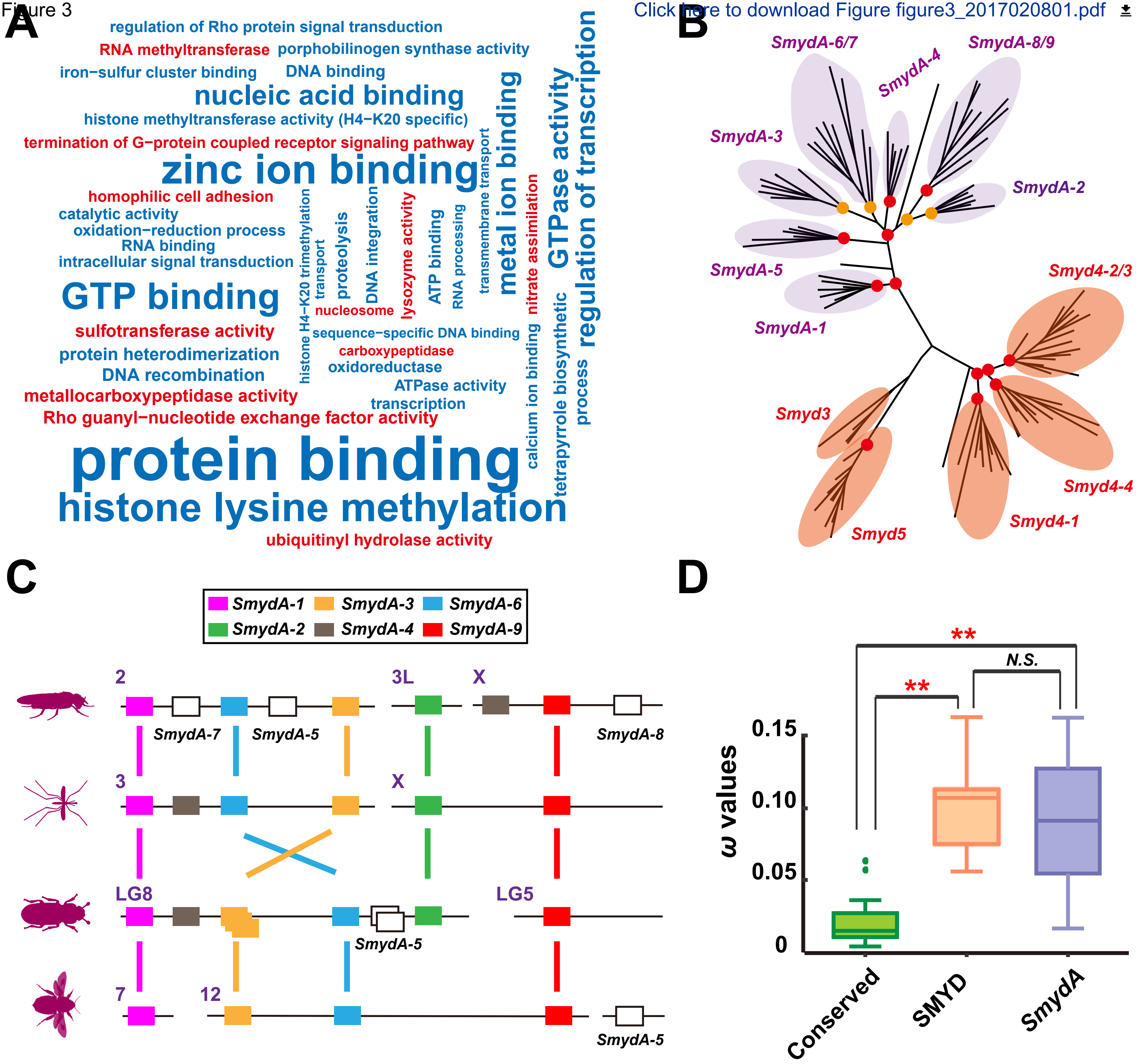

Figure 4
Click here to download Figure figure4_2016102801.pdf

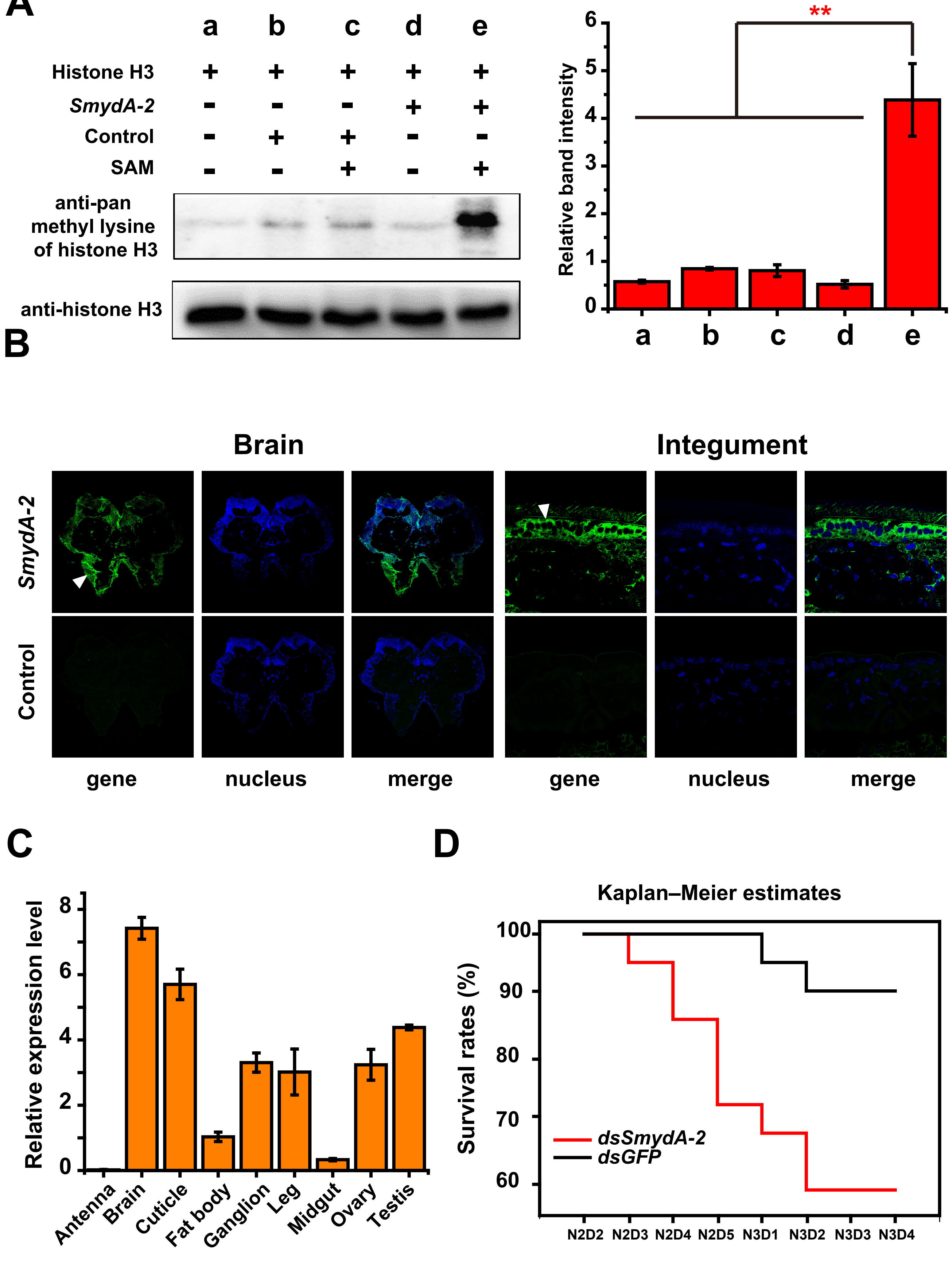

Figure 5

Click here to download Figure figure5_2017020801.pdf

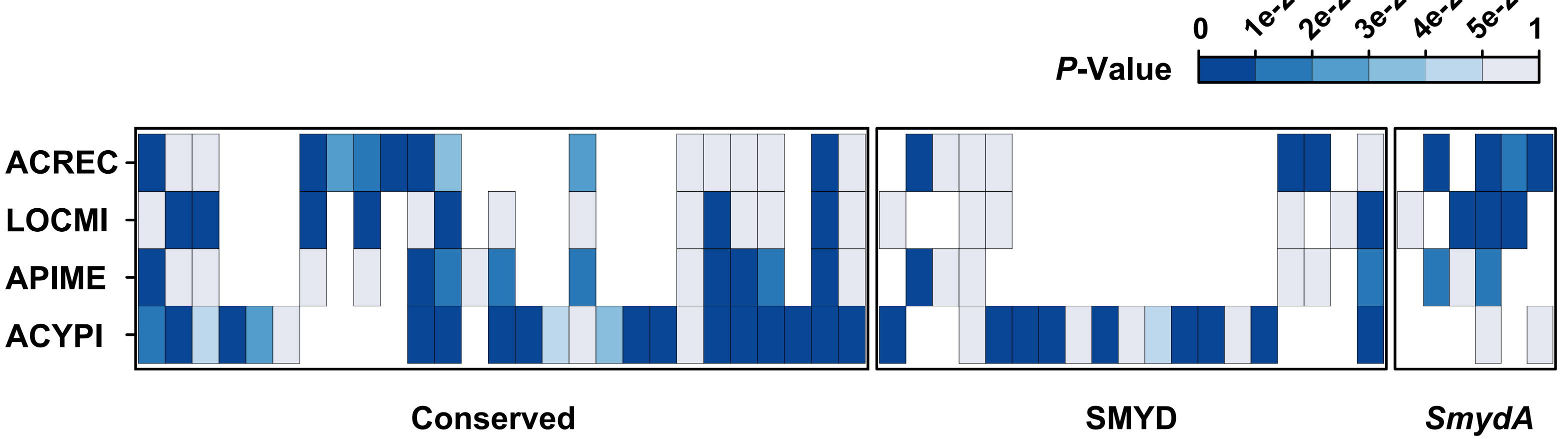

Click here to access/download
Supplementary Material
insectsSetDomain_supply_giga_17021701.pdf
