# Supplementary material for: Comparative genomic analysis of SET domain family reveals the origin, expansion, and putative function of the arthropod-specific SmydA genes as histone modifiers in insects
Source: Gigascience. 2017 Apr 22;6(6):1–16. doi: 10.1093/gigascience/gix031 (PMC5459927; doi:10.1093/gigascience/gix031)
Supplement: Response_to_Reviewer_Comments_revision_1.pdf [file gix031_Response_to_Reviewer_Comments_revision_1.pdf]

Close

We addressed all the editorial concerns.
