# Supplementary material for: Comparative genomic analysis of SET domain family reveals the origin, expansion, and putative function of the arthropod-specific SmydA genes as histone modifiers in insects
Source: Gigascience. 2017 Apr 22;6(6):1–16. doi: 10.1093/gigascience/gix031 (PMC5459927; doi:10.1093/gigascience/gix031)
Supplement: Reviewer_2_Report_(Original_Submission).pdf [file gix031_Reviewer_2_Report_(Original_Submission).pdf]

Reviewer Report

Title: "Comparative genomic analysis of SET-domain family reveals the origin, expansion, and putative function of the arthropod-specific SmydA genes as histone modifiers in insects"

Version: Original Submission **Date:** 12/29/2016

Reviewer name: Máximo Ibo Galindo, Ph.D.

Reviewer Comments to Author:

This manuscript describes an in-depth analysis of the SET genes in arthropod species, with a particular interest in the Smyd class which includes both widely conserved and arthropod-specific members. It is of special interest that the authors make an effort to combine high throughput bioinformatic analyses with an experimental approach to prove that arthropod-specific Smyd proteins retain histone modification activity and are differentially expressed in phenotypicaly different individuals of the same species. The work is well suited for the GigaScience journal, but in the opinion of this reviewer some questions should be addressed.

1. The introduction gives a description of the molecular function of SET domain-containing proteins as histone modification enzymes, but does not discuss already published data on Smyd gene evolution. Given the special emphasis on Smyd genes, it would be convenient to mention previous publications giving a classification of Smyd genes in vertebrates and invertebrates. The article published by Calpena et al (PlosOne 2015) is of particular relevance, since the authors introduce the main Smyd classes: Smyd3, which includes the vertebrate Smyd1 and 2; Smyd 4, which is expanded in arthropods; Smyd 5; and the arthropod-specific SmydA. Based on this evidence, they introduce some of the nomenclature used in this manuscript (Smyd4-1 to 4, SmydA-1 to 9). The manuscript under review makes a more detailed analysis of Set genes in several arthropod species, but giving due credit to previous work does not diminish the merit of theirs. On the contrary, it provides a framework in which to give a richer discussion of their own results.

2. In pages 6 and 7 it is described how the sequences were selected and the overall distribution of set genes in arthropods. Bearing in mind that the process of sequence inclusion/exclusion is very complicated in such a diverse family, the authors must discuss how this may have affected their analysis. This discussion is relevant on the light of the apparent contradictions indicated below (points 8-10).

3. In pages 7 and 8 and in Figure 1 the phylogeny of Set genes is described. Two methods are used, alignment-based Bayesian and alignment-free, and the authors state that both gave similar topologies.

In Figure 1, only the first tree is shown, it would be convenient to show the other phylogeny in a supplementary figure.

4. In Figure 1 there are two color codes, one for domains outside the circle and one for the Set gene classes, but the second one is not mentioned in the figure legend. The black branches must be the arthropod-specific genes. Is that so?

5. At this point it should be mentioned in the main text that this branch corresponds to the already defined SmydA class.

6. From line 177 onwards the term "set homologous group" is used, but it is not defined. My guess is that the 19 homologous groups mentioned in line 185 are the ones in Figure 1B, taking Smyd4-1 to Smyd4-4 as one group. The authors should define clearly what they call a set homologous group and identify them with a reference to a figure or a table. In addition I would suggest reorganising Figure 2 by swapping panels A and B.

7. More information should be given regarding Figure 2. The bars to the right in panel A are not explained in the figure legend. Panel B is based on selected species within each order, but it is not mentioned which are these species. I suggest highlighting them on Supplementary Table S3.

8. As a result of the distribution reflected in Figure 3B it is mentioned in line 233 that SmydA genes are absent in all Chelicerata. But in the Supplementary table S3 it is indicated that chelicerates have arthropod-specific genes in range of 2-6 copies. Please explain this discrepancy and ensure that the information reflected for these and other species is accurate.

9. Calpena et al describe a sub-class within Smyd4, Smyd4-4, which is specific of insects. The authors should discuss if their evidence supports this.

10. The tree in Figure 3B shows a clear segregation between the SmydA and Smyd4 groups, but it does not include Smyd3 or Smyd5. Smyd3 is also highlighted as absent from diptera in Figure 2B, despite the fact that there is a Smyd3 gene defined in Drosophila, which again reinforces the need to discuss the criteria for sequence inclusion (point 2) and the selection of the representative species (point 7). Are these the same representative species selected in Figure 2B?

11. Unless the reader is an expert in entomology, it is difficult to identify the four species only by the body shapes. Species names should be mentioned in the legend.

Methods

Are the methods appropriate to the aims of the study, are they well described, and are necessary controls included? Yes

Conclusions

Are the conclusions adequately supported by the data shown? No

Reporting Standards

Does the manuscript adhere to the journal's guidelines on minimum standards of reporting? Yes

Statistics

Are you able to assess all statistics in the manuscript, including the appropriateness of statistical tests used? No, and I do not feel adequately qualified to assess the statistics.

Quality of Written English

Please indicate the quality of language in the manuscript: Acceptable

Declaration of Competing Interests

Please complete a declaration of competing interests, considering the following questions:

- Have you in the past five years received reimbursements, fees, funding, or salary from an organisation that may in any way gain or lose financially from the publication of this manuscript, either now or in the future?
- Do you hold any stocks or shares in an organisation that may in any way gain or lose financially from the publication of this manuscript, either now or in the future?
- Do you hold or are you currently applying for any patents relating to the content of the manuscript?
- Have you received reimbursements, fees, funding, or salary from an organization that holds or has applied for patents relating to the content of the manuscript?
- Do you have any other financial competing interests?
- Do you have any non-financial competing interests in relation to this paper?

If you can answer no to all of the above, write 'I declare that I have no competing interests' below. If your reply is yes to any, please give details below.

I declare that I have no competing interests

I agree to the open peer review policy of the journal. I understand that my name will be included on my report to the authors and, if the manuscript is accepted for publication, my named report including any attachments I upload will be posted on the website along with the authors' responses. I agree for my report to be made available under an Open Access Creative Commons CC-BY license (http://creativecommons.org/licenses/by/4.0/). I understand that any comments which I do not wish to be included in my named report can be included as confidential comments to the editors, which will not be published.

I agree to the open peer review policy of the journal

To further support our reviewers, we have joined with Publons, where you can gain additional credit to further highlight your hard work (see: https://publons.com/journal/530/gigascience). On publication of this paper, your review will be automatically added to Publons, you can then choose whether or not to claim your Publons credit. I understand this statement.

Yes
