# Supplementary material for: Comparative genomic analysis of SET domain family reveals the origin, expansion, and putative function of the arthropod-specific SmydA genes as histone modifiers in insects
Source: Gigascience. 2017 Apr 22;6(6):1–16. doi: 10.1093/gigascience/gix031 (PMC5459927; doi:10.1093/gigascience/gix031)
Supplement: Reviewer_3_Report_(Original_Submission).pdf [file gix031_Reviewer_3_Report_(Original_Submission).pdf]

Reviewer Report

Title: "Comparative genomic analysis of SET-domain family reveals the origin, expansion, and putative function of the arthropod-specific SmydA genes as histone modifiers in insects"

Version: Original Submission **Date:** 1/6/2017

Reviewer name: Wannes Dermauw

Reviewer Comments to Author:

Jiang et al. performed a thorough phylogenetic analysis of arthropod SET-domain containing genes and identified an arthropod-specific SET gene family (SmydA). They showed that the latter gene family is under strong purifying selection, and complemented their bioinformatic analyses with experimental data showing that a.o. members of the SmydA gene family are essential for insect survival. This is a nice and interesting study but, in my opinion, the manuscript has one major flaw, namely a very concise, in some cases unclear, M&M. Once the issues mentioned below have been addressed, I consider this manuscript acceptable for publication in GigaScience.

Line 348: what was the extent of fold change for SET genes? Were the SET genes among the most highly overexpressed genes in the comparison between alternative phenotypes?

Line 499: which E-value cutoff was used in the HMMER search for SET genes?

Table S2: Next to accession numbers, authors should provide a fasta-file containing the 4,498 SET gene sequences that were used for this study. This way an interested reader does not need to browse different genome portals to collect data. In addition, the study is not dependent on a website that might not be available/working in the future (see also Minor corrections).

Figure 1: author should include a phylogenetic tree (as a supplementary file) with the accession numbers of the sequences (or alternatively upload the alignment to a data repository like e.g. Dryad)

Line 504-505: GO analysis description is very concise/unclear. Authors should provide more details. e.g. be more specific about InterPro databases and models that were used.

Line 512: what settings were used with MAFFT?

line 514: which version of ProtTest software was used?

Line 519: authors should provide more details about the feature frequency profile method (parameters etc…)

Line 528: the authors are very concise regarding the single copy orthologous gene family phylogenetic analysis, authors should provide more details (method?, provide alignment as a supplementary file).

Line 538: authors should provide genome assembly versions of the different insect species

Line 539-542: author should provide parameters for the Tophat2 mapping. What version of Tophat 2 was used? What version of HTSeq and EdgeR was used?

line 542: what was the FC cutoff used for the DE analysis? In addition authors should provide a list of DE genes for all comparisons as a Supplementary Table

line 548: …and then backtranslated…; with what kind of software/script was the backtranslation done?

Line 546-547/: how was the SmydA-2 gene picked up, which primers/PCR protocol were used?

Line 559-560: very concise description of recombinant protein expression? Which primers were used to pick-up gene/ligation protocol (restriction enzymes?) into vector etc. Authors should provide more details.

Line 569: is this a well-established laboratory locust strain? Has this strain been previously described (origin, name?). Authors should provide more details for this strain if possible.

Line 572: authors should provide primers that were used for dsRNA synthesis

Line 578/579: authors should provide more details regarding measuring of SmydA-2 mRNA expression levels (qPCR: primers, amplification protocol, reference genes?...)

Line 580: literature reference for the Kaplan-Meier method?

Line 806: which representative species were used, authors should include a phylogenetic tree (as a supplementary file) with the accession numbers of the sequences (or alternatively upload the alignment to a data repository like e.g. Dryad)

Typos/Minor Corrections

line 143: link to SET gene database is not working (see also comment above)

line 151: why would genome-size be correlated with SET-gene number? Is there any precedent in literature. If not, authors should remove this sentence

Line 155: please specify in the manuscript which "representative species" were used for phylogenetic analysis?

Line 266: was present in all…

Line 284: replace "were" with "are"

Line 309: methylation activities

Line 354: "sensitivities" of DEG number? Authors should rephrase

Line 375: remove "as"

Figure 2B: reformat/resize font so the names of the arthropod specific SET genes can also be shown

Methods

Are the methods appropriate to the aims of the study, are they well described, and are necessary controls included? No

Conclusions

Are the conclusions adequately supported by the data shown? Yes

Reporting Standards

Does the manuscript adhere to the journal's guidelines on [minimum standards of reporting?]() No

Statistics

Are you able to assess all statistics in the manuscript, including the appropriateness of statistical tests used? No, and I do not feel adequately qualified to assess the statistics.

Quality of Written English

Please indicate the quality of language in the manuscript: Needs some language corrections before being published

Declaration of Competing Interests

Please complete a declaration of competing interests, considering the following questions:

- Have you in the past five years received reimbursements, fees, funding, or salary from an organisation that may in any way gain or lose financially from the publication of this manuscript, either now or in the future?
- Do you hold any stocks or shares in an organisation that may in any way gain or lose financially from the publication of this manuscript, either now or in the future?
- Do you hold or are you currently applying for any patents relating to the content of the manuscript?
- Have you received reimbursements, fees, funding, or salary from an organization that holds or has applied for patents relating to the content of the manuscript?

- Do you have any other financial competing interests?
- Do you have any non-financial competing interests in relation to this paper?

If you can answer no to all of the above, write 'I declare that I have no competing interests' below. If your reply is yes to any, please give details below.

I declare that I have no competing interests.

I agree to the open peer review policy of the journal. I understand that my name will be included on my report to the authors and, if the manuscript is accepted for publication, my named report including any attachments I upload will be posted on the website along with the authors' responses. I agree for my report to be made available under an Open Access Creative Commons CC-BY license (http://creativecommons.org/licenses/by/4.0/). I understand that any comments which I do not wish to be included in my named report can be included as confidential comments to the editors, which will not be published.

I agree to the open peer review policy of the journal

To further support our reviewers, we have joined with Publons, where you can gain additional credit to further highlight your hard work (see: https://publons.com/journal/530/gigascience). On publication of this paper, your review will be automatically added to Publons, you can then choose whether or not to claim your Publons credit. I understand this statement.

Yes
